# Structure and ligand binding in the putative anti-microbial peptide transporter protein, YejA

**DOI:** 10.1099/mic.0.001430

**Published:** 2024-02-09

**Authors:** Bryony K. Ackroyd, Eleanor J. Dodson, Javeria Mehboob, Adam A. Dowle, Gavin H. Thomas, Anthony J. Wilkinson

**Affiliations:** ^1^​ York Structural Biology Laboratory and York Biomedical Research Institute, Department of Chemistry, University of York, York YO10 5DD, UK; ^2^​ Department of Biology and York Biomedical Research Institute, University of York, York YO10 5DD, UK; ^3^​ Bioscience Technology Facility, Department of Biology, University of York, York YO10 5DD, UK

**Keywords:** ABC transporter, anti-microbial peptide, crystal structure, mass spectrometry, YejA

## Abstract

YejABEF is an ATP-binding cassette transporter that is implicated in the sensitivity of *Escherichia coli* to anti-microbial peptides, the best-characterized example being microcin C, a peptide-nucleotide antibiotic that targets aspartyl-tRNA synthetase. Here the structure of the extracellular solute binding protein, YejA, has been determined, revealing an oligopeptide-binding protein fold enclosing a ligand-binding pocket larger than those of other peptide-binding proteins of known structure. Prominent electron density in this cavity defines an undecapeptide sequence LGEPRYAFNFN, an observation that is confirmed by mass spectrometry. In the structure, the peptide interactions with the protein are mediated by main chain hydrogen bonds with the exception of Arg5 whose guanidinium side chain makes a set of defining polar interactions with four YejA residues. More detailed characterization of purified recombinant YejA, by a combination of ESI and MALDI-mass spectrometry as well as thermal shift assays, reveals a set of YejA complexes containing overlapping peptides 10–19 residues in length. All contain the sequence LGEPRYAFN. Curiously, these peptides correspond to residues 8–26 of the mature YejA protein, which belong to a unique N-terminal extension that distinguishes YejA from other cluster C oligopeptide binding proteins of known structure. This 35-residue extension is well-ordered and packs across the surface of the protein. The undecapeptide ligand occupies only a fraction of the enclosed pocket volume suggesting the possibility that much larger peptides or peptide conjugates could be accommodated, though thermal shift assays of YejA binding to antimicrobial peptides and peptides unrelated to LGEPRYAFNFN have not provided evidence of binding. While the physiological significance of this ‘auto-binding’ is not clear, the experimental data suggest that it is not an artefact of the crystallization process and that it may have a function in the sensing of periplasmic or membrane stress.

## Introduction

ATP-binding cassette (ABC) peptide transporters are widespread in bacteria and play well-characterized roles in nutrient uptake, cell-wall recycling and cell signalling. Each possesses a complex of four membrane components that effect transport. Two of these are integral membrane proteins that form a channel through which the peptide is translocated and two are ATPases on the cytoplasmic face of the membrane that fuel the transport process allowing accumulation against a steep concentration gradient [1]. The specificity of transporters involved in substrate uptake is determined by a fifth component in the form of a cognate substrate-binding protein (SBP) that captures periplasmic or extracellular peptides in Gram-negative or Gram-positive bacterial cells, respectively [2]. For example, DppA and OppA, that serve as SBPs in the general dipeptide and oligopeptide permeases in *Escherichia coli*, are able to bind hundreds and potentially millions of different short peptides, with peptide length determining the substrate range of the transporters [3, 4]. Some related oligopeptide permeases are served by additional SBPs, which have very narrow specificity, two examples being the murein tripeptide- and tetrapeptide-binding protein, MppA from *E. coli* [5, 6] and the pheromone-binding protein PrgZ of *Enterococcus faecalis* [7]. More rarely narrow peptide specificity is conferred by a complete transporter, for example in the DppBCDE system of *Bacillus subtilis* that binds murein tripeptide [8]. There exist, in addition, numerous peptide transporters whose substrate specificity is less well characterized and/or whose precise function is unknown. The interest here is in one such system, the Yej transporter found in Gram-negative bacteria.

The *yejABEF* operon encodes YejA as the SBP, YejB and YejE as the transmembrane subunits, and YejF as the ATPase driving transmembrane transport. Interestingly, YejF harbours two putative nucleotide-binding domains within a single polypeptide. A clear demonstration that Yej is a peptide transporter came from elegant genetic studies of microcin C resistance in *E. coli* [9–11]. Microcin C is a peptide-nucleotide antibiotic that inhibits translation [9]. It consists of a heptapeptide (fMet-Arg-Thr-Gly-Asn-Ala-Asp) C-terminally linked to a modified adenosine monophosphate through a phosphoramidate bond. Inside sensitive cells, microcin C is processed by a peptide deformylase and one or more peptidases giving rise to a non-hydrolysable aspartyl adenylate that inhibits aspartyl tRNA synthetase [10]. Analysis of a transposon-insertion library led to the discovery of microcin C-resistant mutants with insertions in *yejABEF* with follow-up experiments showing that deletion of any one of the four genes of the *yej* operon conferred resistance to microcin C [9]. Later structure-activity studies showed that YejABEF can transport larger microcin C analogues across the bacterial inner membrane, though the upper chain length was not determined [11]. In the absence of knowledge of the physiological substrates of the transporter, these authors sought to establish the peptide length dependence of YejABEF-mediated uptake in competition assays using microcin C. This led to the tentative conclusion that the Yej transporter handles peptides in the range 7–13 residues [11].

In *Salmonella enterica* subsp. Typhimurium, the *yejABEF* operon has been associated with virulence following the discovery that *yejE* and *yejF* [12, 13] and later the whole *yej* operon [14] are up-regulated following infection of macrophages and epithelial cells. In view of the inferences from sequence analysis that YejABEF is a peptide transporter, the latter authors examined the hypothesis that the *yej* operon confers resistance to anti-microbial peptides (AMPs). In the course of infection of various hosts, *Salmonella* spp. are exposed to a range of AMPs as components of the innate immune response. AMPs are heterogeneous in character but typically comprise 10–50 amino acid residues often enriched for side chains with basic functional groups. They form secondary and sometimes tertiary structures with broad-spectrum activity often disrupting or permeabilizing the membranes of pathogenic bacteria, fungi or enveloped viruses [15]. In *Salmonella*, deletion of *yejF* increased sensitivity to anti-microbial peptides, including human defensins, protamine, melittin and polymyxin B with electron micrographs revealing gross membrane defects in a high proportion of the cells [14]. These deletion mutants were defective in proliferation on the macrophage and epithelial lines tested, moreover they were less virulent in a mouse model of typhoid infection. The mechanism by which Yej confers resistance to AMPs is not known. One hypothesis is that the AMPs are transported away from their site of action on the cell surface and into the bacterial cytoplasm where they are degraded.

A later study in the α-proteobacterium *Brucella melitensis* NI, an intracellular pathogen that causes severe febrile illness in humans, also led to association of the orthologous Yej ABC transporter with resistance to the antimicrobial peptide polymyxin B [16]. Polymyxin B, a cationic cyclic heptapeptide bearing a lipidated tripeptide attachment, is produced by *Bacillus polymyxa*. In *B. melitensis*, the *yej* operon comprises five genes *yejA1A2BEF,* with *yejA1* and *yejA2* encoding two predicted SBPs. Single gene and whole operon deletion mutants were constructed. The whole operon and *yejE* deletion mutants grew normally on defined media but exhibited increased sensitivity to acid stress, increased susceptibility to polymyxin B and a reduced capacity to invade macrophages and persist in the spleens of infected mice. Interestingly, polymyxin B induces expression of the *yej* operon. Curiously, the other single gene deletions did not change the susceptibility to polymyxin B suggesting perhaps that a function of the transporter components that does not require transport is involved. For *yejA1* and *yejA2*, there may be functional redundancy, which was not examined in this study [16].

The solute-binding proteins of ABC transporters in bacteria act as receptors for their cognate uptake system and define its specificity [3]. They bind ligands with moderate to high affinity (*K*_d_ ~1 µM) capturing substrates through a domain closure mechanism that has been likened to a Venus fly-trap. Solute-binding proteins often co-purify with ligands in their enclosed interiors and their crystal structures have in some instances revealed substrate specificity and function that was hitherto unknown [17]. For peptide transporters, similar approaches have helped to define the length preference of the substrates [18–20]. Following, its secretion to the periplasm, mature YejA from *E. coli* is expected to comprise 585 residues. It belongs to the cluster C solute-binding proteins, which are larger in size due to their possession of an extra subdomain that allows them to bind larger substrates [21]. Recently, the structure of YejA from *Sinorhizobium meliloti*, which shares 38 % sequence identity with the *E. coli* orthologue was reported in a complex with copurifying di- and pentapeptides [22]. Among other functionally characterized proteins of known structure and specificity, YejA from *E. coli* has highest sequence identity (23.6 %) to AppA from *Bacillus subtilis*. To explore the substrate specificity of the Yej transporter from *E. coli*, we have determined the crystal structure of its periplasmic solute-binding protein component, YejA and measured ligand binding in thermal shift assays and by mass spectrometry.

## Methods

### Plasmid construction

A DNA fragment encompassing the coding sequence of *yejA* was amplified from *E. coli* K12 genomic DNA by the PCR using the oligonucleotide pair EcYejA-F and EcYejA-R (Table S1). The PCR primers were designed with HiFi specific overhangs for cloning into pETFPP_30, a derivative of pET-22b+. The 1804 base-pair PCR product was purified by PCR Clean up (QIAgen) and mixed with pETFPP_30 vector that had been ‘linearized’ with NdeI and XhoI. HiFi DNA assembly (NEB) was carried out following the supplier’s instructions and the ligation products were introduced into Solopack Gold DH5α competent cells (Agilent). Plasmid recovered from ampicillin-resistant transformants was sequenced (GATC) using the T7 promoter and T7 terminator primers. The verified plasmid pBKA101 encodes a recombinant protein consisting of residues 1–585 of mature *E. coli* YejA fused N-terminally to a methionine residue and C-terminally to a human rhinovirus 3C (HRV 3C) cleavable hexahistidine tag (Table S1).

### Protein production and purification

*Escherichia coli* BL21 (DE3) cells harbouring pBKA101 were grown with shaking as 1 litre cultures in Luria–Bertani broth supplemented with ampicillin (100 µg ml^−1^) at 37 °C to an OD_600_ of 0.4–0.6 before induction of recombinant protein production by addition of isopropyl β-d-thiogalactopyranoside (IPTG) to 1 mM. Cells were grown either for a further 3 h at 37 °C or overnight at 30 °C. Cells were harvested by centrifugation at 5000 r.p.m. for 20 min at 4 °C in an F10S rotor (Thermo Scientific). Cells were resuspended in 35 ml buffer A (50 mM potassium phosphate, 200 mM NaCl, 20 % glycerol, pH 7.8) containing 10 mM imidazole to which phenylmethylsulphonyl fluoride was added to 1 mM prior to sonication (20 kHz for 6 mins, over a period of 30 mins at 4 °C). Cell debris was removed by centrifugation [15 000 r.p.m. for 25 min in an SS34 rotor (Thermo Scientific)] and the supernatant was applied to a nickel-charged HisTrap HP column (GE Healthcare) pre-equilibrated with buffer A containing 40 mM imidazole. The bound protein was eluted in a single step with buffer A containing 500 mM imidazole. The eluate was analysed by polyacrylamide gel electrophoresis (SDS-PAGE) and fractions enriched in a recombinant protein of apparent Mr=65 kDa were combined and dialysed overnight against buffer B (50 mM Tris-HCl pH 8.0, 150 mM NaCl) in the presence of HRV 3C protease to remove the C-terminal hexa-histidine tag. The digestion products were concentrated by pressure membrane filtration (30 000 Da cut off) and subjected to size exclusion chromatography on an S200 16/600 column (Cytiva) equilibrated in buffer B. Recombinant protein-containing fractions were identified by SDS-PAGE, combined and concentrated. The overall yield was approximately 50 mg of purified protein per litre of shaking bacterial culture.

The crystal structure revealed the presence of endogenous ligands in the peptide binding site. We therefore sought, in subsequent preparations, to remove these ligands and obtain unliganded YejA for peptide-binding studies. In these preparations therefore, the Ni-NTA column chromatography purification procedure was modified so that following the binding and washing steps, the column was washed with buffer C (buffer A containing 2 M guanidine hydrochloride), to partially unfold the immobilised protein. After washing with 10 column volumes of buffer C, the concentration of guanidinium hydrochloride was decreased to zero in a series of five steps [23]. The protein was then eluted from the column with 500 mM imidazole as before.

### Size exclusion chromatography with multi-angle laser light scattering (SEC-MALLS)

YejA samples at either 1 mg ml^−1^ or 3 mg ml^−1^ in 20 mM Tris-HCl pH 8.0, 50 mM NaCl were resolved on a Superdex 200 10/300 s column (GE Healthcare) at 0.5 ml min^−1^ with an HPLC system (Shimadzu). Light-scattering data were collected continuously on material eluting from the column using an in-line Wyatt Dawn Heleos LS detector with an inline Wyatt Optilab rEX refractive index detector and an SPD-20A UV detector. Molecular weights were calculated by analysing data with the Wyatt programme ASTRA.

### Circular dichroism (CD) spectroscopy

CD spectra of YejA were collected on a J-810 spectropolarimeter (Jasco) along with the supplied software SpectraManager version 1.53.00 (Jasco). Then, 0.2 mg ml^−1^ (2.92 µM) protein was analysed in 20 mM Tris pH 8.0 and 50 mM NaCl buffer. Spectra were recorded at 20 °C in a 1 mm path-length quartz cuvette (Starna) over the wavelength range 190–260 nm at 200 nm min^−1^ with 0.5 nm pitch.

### Crystallization and structure determination

Protein concentrations were determined with an Epoch Microplate Spectrophotometer using an extinction coefficient at 280 nm calculated from the sequence using Protparam (https://web.expasy.org/protparam/). Vapour diffusion crystallization experiments were set up as sitting drops in 96-well plates using Hydra 96 and Mosquito liquid-handling systems to dispense reservoir and drop solutions, respectively. JCSG, PDB Min and PEG/ION screens were explored. Crystals of YejA were obtained from sitting nanodrops set up with 150 nl of 20 mg ml^−1^ YejA in 20 mM Tris-HCl, 50 mM NaCl pH 8.0, and 150 nl of well solution composed of 0.1 M MES pH 6.5, 12 % w/v polyethylene glycol 20 000. These single crystals were discovered approximately 1 month after the drops were set up and had typical dimensions of 0.25×0.1×0.1 mm^3^.

Single crystals were transferred to a solution of mother containing 30 % glycerol, captured in a fine nylon loop, and cryocooled in liquid nitrogen for data collection at the DIAMOND Light Source on beamline i04-1. Data processing showed the space group to be I222 with cell dimensions *a*=91.4 Å, *b*=105.3 Å, *c*=145.3 Å indicating a single protein chain in the asymmetric unit and a solvent content of 52 %. Data were processed using *xia2* [24] and extended to 1.6 Å spacing and the statistics are shown in Table 1.

**Table 1.** Data collection and processing

Diffraction source	I04, DLS
**Wavelength (Å**)	0.9282
**Temperature (K**)	100
**Detector**	Pilatus 6 M-F
**Crystal-detector distance (mm**)	203.86
**Rotation range per image (°**)	0.1
**Total rotation range (°**)	220
**Space group**	I222
***a, b, c* (Å**)	91.44, 105.27, 145.39
**a, b, g (°**)	90.0, 90.0, 90.0
Resolution range (Å) *	85.26–1.55 (1.59–1.55)
**Total no. of reflections**	844 713 (41,880)
**No. of unique reflections**	101 011 (4,907)
**Completeness (%**)	99.6(99.0)
**Multiplicity**	8.4(8.5)
**I/Σ(I)**	14.1(2.4)
**Rpim †**	0.027(0.253)
**CC_1/2_**	0.997(0.904)
**Overall B factor from Wilson plot (Å^2^**)	15.46

*Values in parentheses correspond to the outer resolution shell.

† *R*_pim_ = ∑*hkl*[1/(*n–*1)]^1/2^∑*i*|*I*_*i*_ –〈*I*〉|/∑*hkl*∑*I*_*i*_ where *I*_*i*_ is the intensity of the *i*th measurement of a reflection with indices *hkl* and 〈*I*〉 is the statistically weighted average reflection intensity.

The structure was solved by molecular replacement in the program molrep [25] using as the search model the coordinate set 4ONY for an otherwise uncharacterized solute-binding protein from *Brucella melitensis* (Uniprot ID C0RL96), representing the closest homologue (32 % sequence identity) in the Protein Data Bank. Searches with the complete molecule did not give a satisfactory solution. Thus, the 4ONY structure was partitioned into two lobes and molecular replacement calculations were performed firstly using residues 1–261 and 545–580. This gave a convincing solution, which was refined using the program refmac [26, 27], fixed, and a second molecular replacement calculation was carried out using residues 262–544. This gave a solution which was subsequently refined in refmac and gaps in the model filled in using buccaneer [28]. Iterative rounds of model building using coot [29] and refinement in refmac5 brought the *R* and *R*_free_ factors down to 19 and 21 % respectively. At this point, a peptide ligand was built into an extended electron density feature, which could not be accounted for by protein atoms. Final refinement statistics are shown in Table 2. The coordinates and structure factor have been deposited in the Protein Data Bank with Accession Code 7ATR.

**Table 2.** Structure refinement

Resolution range (Å)*	85.26–1.55 (1.59–1.55)
Completeness (%)*	99.53(98.89)
σ cutoff	None
No. of reflections, working set*	96 135 (6,890)
No. of reflections, test set*	4874 (349)
Final *R*_cryst_*	0.170(0.237)
Final *R*_free_*	0.185(0.263)
Cruickshank DPI	0.072
No. of non-H atoms	
Protein	4811
Glycerol	18
Ligand	408
Total	5331
R.m.s. deviations	
Bonds (Å)	0.0134
Angles (°)	1.827
Average B factors (Å^2^)	
Protein	21.37
Glycerol	32.2
Ligand	29.3
Water	29.17
Ramachandran plot	
Favoured regions (%)	96.76
Additionally allowed (%)	2.56
Outliers (%)	0.68

*Values in parentheses correspond to the outer resolution shell.

### Electrospray mass spectrometry

Electrospray ionization mass spectrometry was conducted on a Waters LCT Premier XE system with MassLynx 4.1 software. The system was calibrated with sodium formate solution and calibration verified with horse heart myoglobin (16 951.5±1.5 Da). YejA was run at a concentration of 10 mg ml^−1^ in 2 mM Tris-HCl pH 8.0. Samples were prepared in 1 : 1 acetonitrile-water containing 0.1 % formic acid.

In later electrospray ionization mass spectrometry (ESI-MS) data acquisitions, protein in aqueous 1 M ammonium acetate was infused at a flow rate of 3 µl h^−1^ at 3 mg ml^−1^ concentration into a Bruker maXis qTOF mass spectrometer via an electrospray ionization source. Dry gas temperature was 250 °C and the dry gas flow rate was 6 l s^−1^. Spectra were summed over 1 min acquisitions at 0.1 Hz spectral acquisition rate. Ion funnel voltages were adjusted to aid the preservation (100 eV) or separation (200 eV) of gas-phase complexes. Subsequently data were smoothed (0.5 Da, 1 cycle, Gauss) and baseline subtracted (flatness 0.8) before maximum entropy deconvolution to average masses at 10 K resolution.

For MALDI-MS/MS, a 100 µl aliquot of sample was acidified with the addition of 10 µl aqueous 1 % trifluoroacetic acid (TFA) before extracting and desalting peptides using Promega C_18_ ZipTips. Desalted peptides were spotted out onto a MALDI target plate and overlaid with 5 mg ml^−1^ 4-hydroxy-α-cyano-cinnamic acid matrix. Peptides were analysed by MALDI-MS/MS using a Bruker ultraflex III mass spectrometer with the 30 strongest precursors, with a signal-to-noise (S/N) ratio greater than 30, selected for MS/MS fragmentation. Spectra were baseline-subtracted and smoothed (Savitsky-Golay, width 0.15 *m/z*, cycles 4); monoisotopic peak detection used a SNAP averaging algorithm C_4.9384_, N_1.3577_, O_1.4773_, S_0.0417_, H_7.7583_) with a minimum S/N ratio of 5. MS^2^ spectra were searched against the expected sequence of YejA using the Mascot search program with enzyme cleavage set at wild.

### Thermal shift assays

A final concentration of 0.5 mg ml^−1^ (7.3 µM) YejA and 5×SYPRO orange dye (Sigma) was used in each assay in a total volume of 25 µl per well. A Stratagene Mx3005P real-time qPCR instrument (Qiagen) was used with fluorescence excitation at 517 nm and emission measured at 585 nm following 1 °C/30 s temperature increments over the range 25–96 °C. Data analysis was performed using JSTA.

### Bioinformatics

Query protein EcYejA (UniProt ID P33913) was compared with RefSeq select proteins database by using blastp (blast NCBI), set to return up to 5000 sequences from Proteobacteria, which were then reduced to 451 species sequences to have one species per genus. This was supplemented with additional genus relevant for comparisons being made in the study, namely *Sinorhizobium* and *Brucella*. Fasta sequences were aligned using Clustal alignment in Jalview [30] .

## Results and discussion

### Overproduction and purification of recombinant YejA

**Fig. 1.**
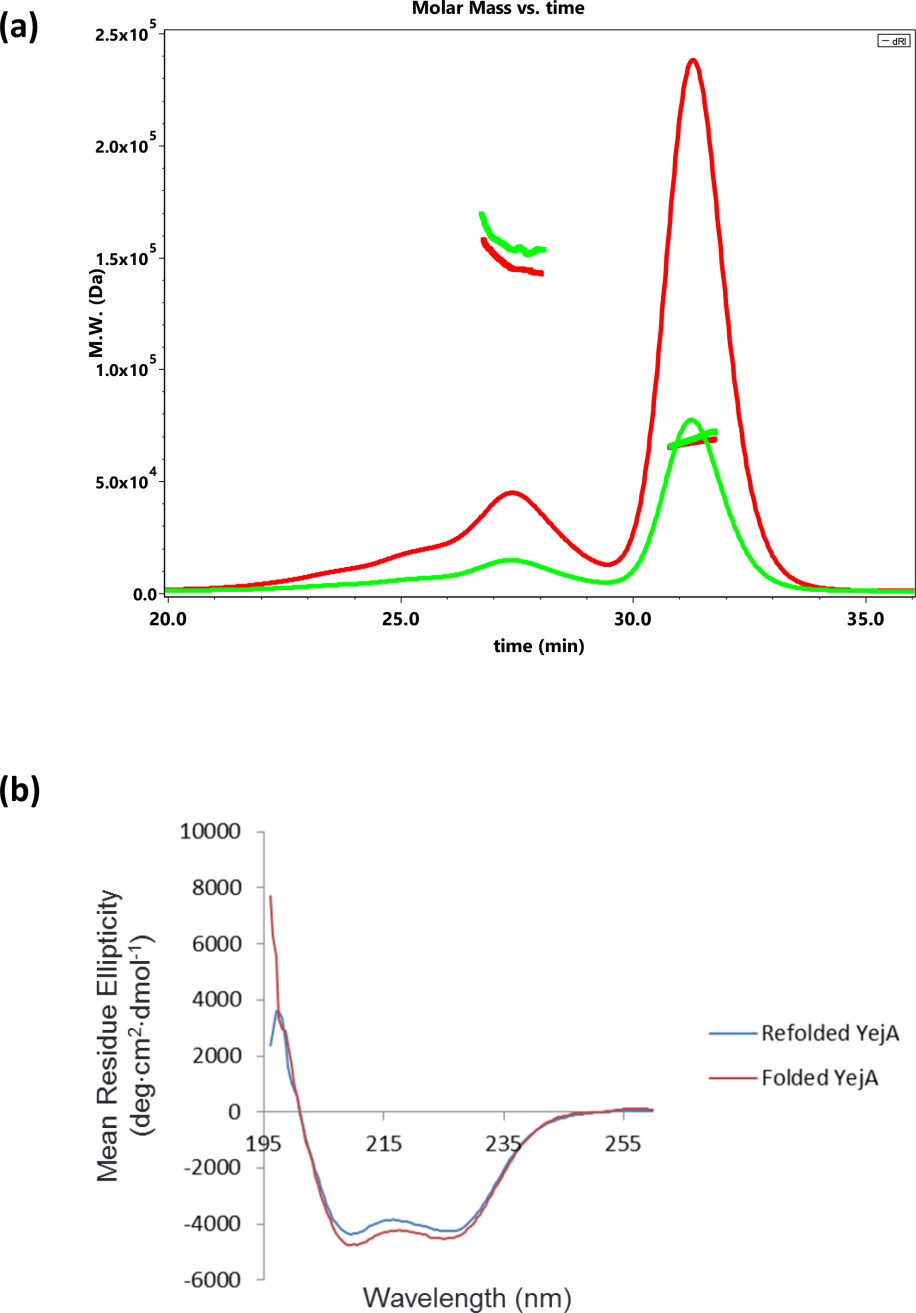
Physical properties of YejA. (a) Molecular mass of YejA measured from SEC-MALLS analysis. In total, 100 µl samples of YejA at 1 mg ml^−1^ (green trace) and 3 mg ml^−1^ (red trace) were loaded onto a Superdex S200 10/300 gel filtration column. The thinner continuous lines trace the refractive index of the eluate as a function of time while the thicker lines indicate the weight average molecular weight of species in the eluate calculated using the light scattering measurements. (b) Circular dichroism spectra of YejA recorded at 20 °C on a Jasco J-810 CD spectrophotometer using a 0.1 cm length quartz cuvette. The red and blue lines represent YejA before and after treatment with 2M guanidinium-HCl and refolding.

YejA is expected to localize to the *E. coli* periplasm following secretion by the Sec system and removal of the N-terminal signal peptide. For soluble recombinant protein production in *E. coli*, we constructed a plasmid directing the production of mature YejA fused C-terminally to an HRV 3C cleavable hexahistidine tag (Table S1). Following cell lysis, affinity chromatography and gel filtration, purified recombinant YejA consists of an N-terminal methionine, residues Gln1 - Glu585 of the mature protein and a vestigial LEVLFQ sequence at the C-terminus produced by HRV 3C protease cleavage. Electrospray ionization mass spectrometry analysis of purified YejA under denaturing conditions gave a mass of 68 383 Da very close to the mass of the fusion protein calculated from its sequence (68 382 kDa). Analysis of purified YejA by size exclusion chromatography and multi-angle laser light scattering (SEC-MALLS) revealed the major species to be a monomer (65 kDa) with evidence for the presence of a minor dimeric species (Fig. 1a). Assessment of the protein quality using circular dichroism revealed a pair of shallow minima in the spectrum across the wavelength range 200–230 nm, indicating the presence of secondary structure consistent with a fully folded protein (Fig. 1b).

### YejA has a typical cluster C fold with an extended and structured N-terminal segment

**Fig. 2.**
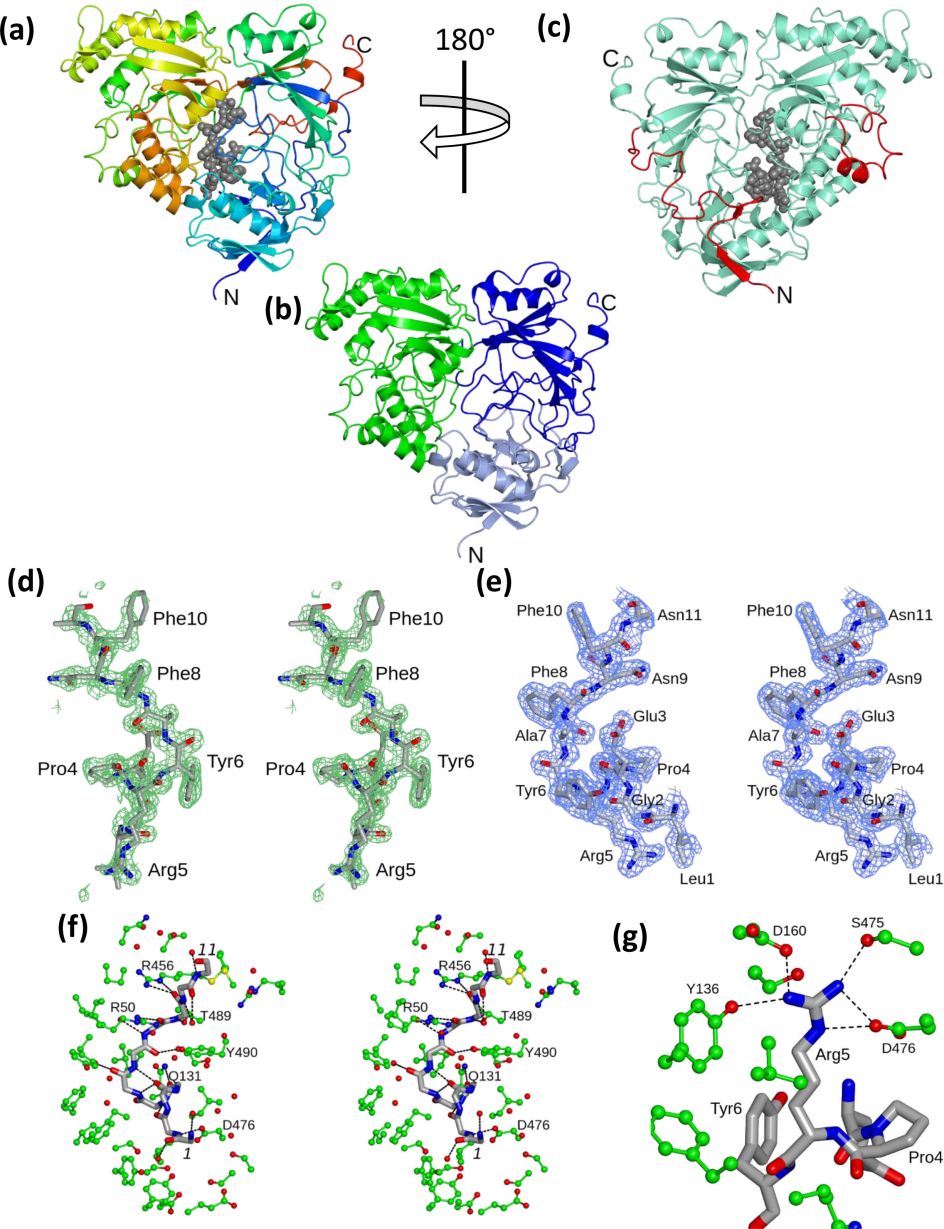
Structure of YejA. (a) Ribbon tracing colour ramped from N (blue) to C (red) termini with the peptide ligand shown as grey spheres. (b) Ribbon tracing of YejA emphasing the lobe/domain structure. Lobe 1 comprises two domains coloured in different shades of blue. Lobe 2 is coloured green. (c) Ribbon trace of the YejA polypeptide drawn in aquamarine with the N-terminal residues 1–35 and residues 343–368 shown in red. (d) Stereo view of the Fo-Fc electron density maps contoured at 3σ and calculated before the ligand was added to the model. The map is displayed on the refined undecapeptide coordinates. (e) 2F_o_-F_c_ electron-density map in the refined model contoured at 0.67σ and displayed on the undecapeptide. (f) Stereo view of the main chain of the undecapeptide ligand displayed together with selected protein side chains and water molecules. Polar interactions are shown as dashed lines. (g) Interactions of Arg5 of the peptide ligand.

Despite extensive efforts, crystals of YejA suitable for structure determination were obtained only on a single occasion. These crystals were grown from a batch of protein that had not undergone the mild unfolding treatment that would remove endogenous ligands. They were discovered in a nanodrop approximately 1 month after the experiment had been set up. A diffraction dataset from these crystals extending to 1.6 Å spacing, collected at the DIAMOND light source, allowed the structure to be solved. The electron-density maps following molecular replacement were of high quality allowing uninterrupted tracing of the polypeptide chain from the N-terminus (Met0) to Ser579 (Fig. 2a). The protein folds to form two lobes comprising residues 1–286 and 540–579 (lobe 1) and residues 287–539 (lobe 2). The segments 282–287 and 540–542, which do not participate in secondary structure and which bridge the lobes are thus likely to form the hinge that would allow inter-lobe movements necessary for ligand entry and exit. Lobe 1 can be partitioned into two domains I and II, which are coloured in different shades of blue in Fig. 2b. Each contains a β-sheet. The chain topology is the same as that seen in other oligopeptide-binding proteins. The closest structure to YejA of *E. coli* in the Protein Data Bank is not surprisingly that of YejA from *S. meliloti* (7Z8E), the two structures superposing with an rmsΔ of 1.67 Å for 543 matching C_α_ atoms. For lobes I and II the rmsΔ values are 1.80 and 1.39 Å for 311 and 236 matching C_α_s, respectively. The next closest matches are the oligopeptide binding protein AppA from *B. subtilis* (1xoc) ahead of a series of other dipeptide-, oligopeptide- and metal chelate binding proteins.

Both the *E. coli* and *S. meliloti* YejA proteins share a common feature in being some 60 amino acid residues longer than other peptide binding proteins. This can be accounted for, in large part, by a 35 residue N-terminal extension and an insertion of 25 residues (343–368), which form a loop on the surface of lobe 2 (Figs 2c, S1 and S2A, available in the online version of this article). Neither of these regions contributes to the expected peptide-binding site, which resides in the interior. The N-terminal segment contributes a fifth strand to the four-stranded β-sheet at the base of domain II before meandering across the surface of domain II and domain I leading into the first strand of the central β-sheet of domain I (Fig. 2c). In other structures of oligopeptide binding proteins, the N- and the C-termini are in close proximity preceding or following strands, which contribute to the β-sheet in domain I. Where N-terminal extensions occur these have variable conformation and a tendency towards disorder. The extensive packing of residues 1–35 onto the surface of the protein with an interface of 1675 Å^2^ and the excellent chemical complementary across this interface suggests this is not the case for YejA and that this is a core part of the fold.

Comparison of the experimentally determined crystal structure of YejA with a model generated using Alphafold [31] shows very good agreement with lobes I and II superimposable with rmsΔ values of 0.84 and 0.81 Å, respectively, for 325 and 255 equivalent atoms. The predicted conformations of the additional N-terminal and insertion segments agree well with the crystal structure. The principal disagreement between the two structures relates to the juxtaposition of the lobes. The rmsΔ following SSM superposition of the complete chains is 3.28 Å for 487 matched atoms. This is to be expected in proteins such as YejA where relative motions of the lobes are an integral component of ligand binding.

### The ligand binding site contains a well-defined undecapeptide

Upon completion of the polypeptide chain tracing, it was evident that there was additional density in the protein interior in what is expected to be the ligand-binding site. SBPs frequently co-purify and co-crystallize with their cognate ligands and this has been used to assign functions to ABC transporters of hitherto unknown specificity [17]. In the case of peptide-binding proteins, which are often broadly specific, the crystals may be expected to contain a broad range of ligands reflecting the relative ligand affinity and the peptide substrates available to the protein. As a result, the electron density is averaged over multiple ligands. For YejA however, the electron-density feature was both extended and well-defined and could be modelled as an undecapeptide that obviously contained the sequence ProArgTyrXxxPheXxxPhe (Fig. 2d). A database search against the *E. coli* genome gave the most likely source of this peptide sequence as residues 15–21 of YejA itself. As a result, the intervening and flanking residues were straightforwardly fitted as residues 12–22 of *Ec*YejA, Leu-Gly-Glu-Pro-Arg-Tyr-Ala-Phe-Asn-Phe-Asn. In the refined structure, the electron density is an excellent match for this sequence though the side chain of Asn11 is not well defined and there is evidence for the presence of peptide extensions at the N-terminus and at the C-terminus (Fig. 2e).

The main chain portion of the undecapeptide forms polar interactions with six residues of YejA. The amide group of Gly2 interacts with the carboxylate of Asp476 (Fig. 2f). The carbonyl group of Pro4 forms a hydrogen bond to the side chain amide –NH_2_ of Gln131. The carbonyl oxygen of Ala7 forms a hydrogen bond with the phenolic hydroxyl of Tyr490 and there follows a series of interactions between, the >C=O of Phe8 and the side chain of Arg50, the >NH of Asn9 and the hydroxyl of Thr489, and the >C=O of Asn9 and the guanidino group of Arg456 (Fig. 2f). The α-amino group of Leu1 forms a polar interaction with a single water molecule as does this residue’s carbonyl group (Fig. 2f). Further interactions of ligand main chain groups with water molecules are made by the amide >NHs of Asn9, Phe10 and Asn11 together with the carbonyl oxygens of Tyr6 and Phe10. The carbonyl of Pro2 makes an intramolecular hydrogen bond to the >NH of Arg5 while further along the chain, the >C=O of Glu3 forms bifurcated hydrogen bonds to the amide groups of ligand residues Tyr6 and Ala7.

There are few strong interactions between the side chains of the ligand and the protein. The striking exception is the side chain of Arginine-5, which makes an extensive set of polar interactions with YejA (Fig. 2g). Its guanidino group forms ion-pairing interactions with the carboxylate side chains of both Asp160 and Asp476, as well as charge dipole interactions with the phenolic hydroxyl of Tyr136 and the hydroxyl of Ser475. The only other side chain hydrogen bonding interaction is formed by the amide of Asn9 and the guanidinium of Arg465.

### YejA encloses a voluminous peptide-binding cavity

**Fig. 3.**
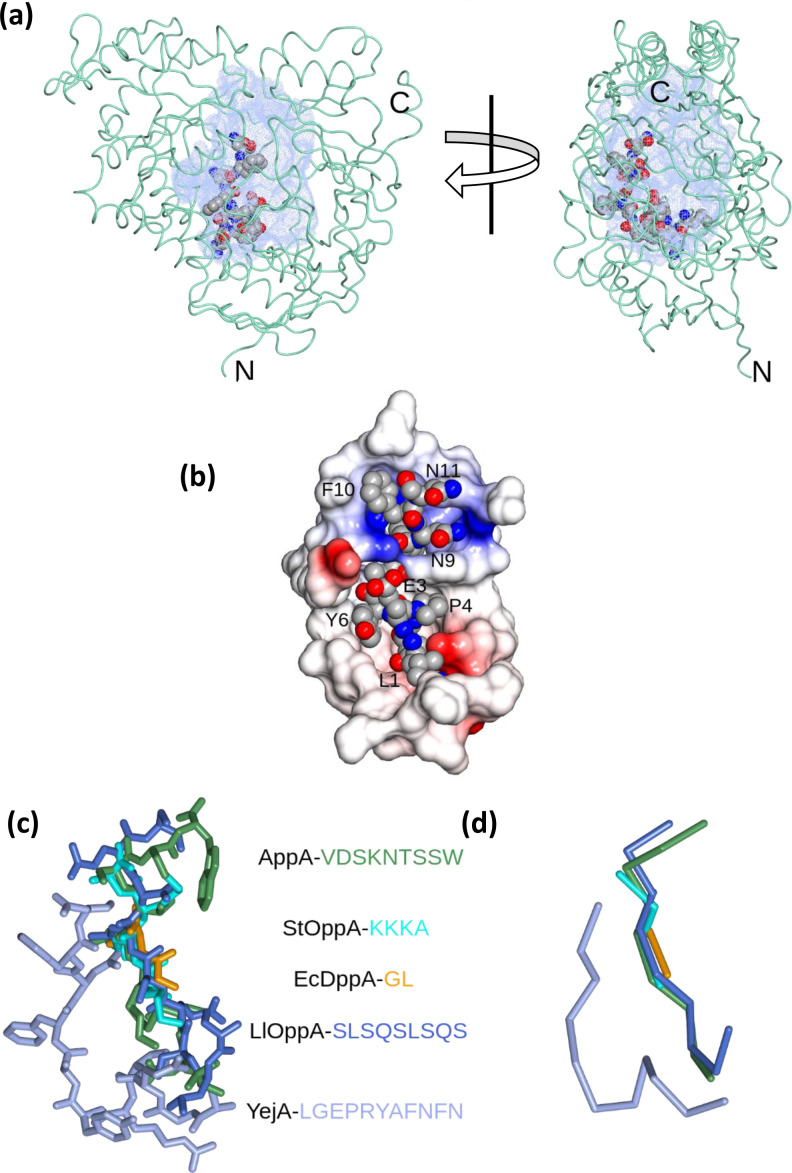
The ligand-binding site in YejA. (a) Approximately orthogonal views of the enclosed cavity in YejA. The interior surface calculated in the programme, rbcavity [34] is displayed as blue dots within the YejA protein represented as a worm tracing and with the undecapeptide ligand shown as atomic spheres. (b) View of the undecapeptide from within the interior cavity, with neighbouring YejA residues displayed as an electrostatic surface. The undeacapeptide is represented as spheres coloured by atom type. (c) All atom and (d) Cα atom representations of Gly-Leu in EcDppA [38] (orange; 1dpp), Lys-Lys-Lys-Ala in StOppA [39] (cyan; 1olc), Val-Asp-Ser-Lys-Asn-Thr-Ser-Ser-Trp in BsAppA [19] (lawn green; 1xoc), Ser-Leu-Ser-Gln-Ser-Leu-Ser-Gln-Ser in LlOppA [32] (light blue; 3ryb), and Leu-Gly-Glu-Pro-Arg-Tyr-Ala-Phe-Asn-Phe-Asn in EcYejA (ice blue). The structures of the proteins were superposed using the SSM Superpose routine implemented in CCP4mg [40].

The undecapeptide ligand is asymmetrically positioned in what is a sizeable interior cavity (Fig. 3a). As a result, despite its enclosed location, the ligand has an extensive surface that is not buried by direct interactions with the protein. The side chains of Leu1, Glu3, Pro4, Tyr6, Asn9 Phe10 and Asn11 are oriented partially or completely towards the cavity (Fig. 3b). At the heart of this face of the peptide is the side chain of Glu3 whose carboxylate makes interactions with three buried water molecules. The structure is more tightly packed around Arg5 as already mentioned, as well as at Gly2 and Ala7 where steric hindrance would restrict the size of the side chain. Finally, Phe8 is snugly bound in a pocket circumscribed by Leu53, Val129, Pro130, Thr489 and Arg 456 and through which there is a narrow opening to the bulk solvent.

The mode of binding of the ligand, LGEPRYAFNFN, in YejA is distinct from the mode of peptide binding in StOppA and EcDppA, where ion-pairing interactions with the ligands’ charged N- and C-termini, and hydrogen bonding to the main chain amide and carbonyl groups, contribute to sequence-independent binding [18]. It has more similarities to peptide binding in OppA from *Lactococcus lactis* (LlOppA), which binds a range of longer peptide ligands [32]. In LlOppA, there are no constraints on the position of the N- and C-termini of the peptide allowing peptides to bind in various ‘*registers*’. Instead there is a side chain binding cavity that exhibits a strong preference for large hydrophobic residues [33]. The absence of defining interactions between YejA and the N- and C-termini of LGEPRYAFNFN and the apparent selection for Arg5 in this peptide may indicate a similar peptide binding strategy. It should be noted however, that whereas peptides bind in an overlapping volume in DppA, the two OppAs and AppA at the core of the protein, the peptide in YejA is significantly displaced (Fig. 3c, d), moreover in YejA, the peptide is in a noticeably less extended conformation with the backbone adopting a curvature. Whereas the convex surface of the peptide makes extensive direct contacts to the protein, the concave surface faces the interior cavity.

The enclosed ligand-binding cavity is strikingly large with the undecapeptide occupying only a fraction of the available volume (Fig. 3a). The interior volume was calculated using the rbcavity routine in rDock as 8253 Å^3^ [34]. This dwarfs the cavities in *Ec*DppA (1434 Å^3^), StOppA (1874 Å^3^), BsAppA (3773 Å^3^) and even exceeds that of LlOppA (5955 Å^3^). The volume of the ligand-binding cavity in these well-characterized peptide-binding proteins correlates with the upper limit on the size of the ligands bound (Table 3). Thus, the volume of the cavity in YejA is consistent with its proposed role in binding larger longer peptides and potentially even small folded proteins that are able to access the periplasm.

**Table 3.** Peptide binding pocket volumes

Protein	PDB ID	Cavity vol. (Å^3^)*	Ligand	Ligand repertoire	Reference
YejA	7atr	8253	LGEPRYAFNFN	Anti-microbial peptides	This work
StOppA	1olc	1874	KKKA	Oligopeptides 2–5	[39]
LlOppA	3ryb	5955	SLSQSLSQS	Oligopeptides 7–35	[33]
EcDppA	1dpp	1434	GL	Dipeptides	[38]
BsAppA	1xoc	3773	VDSKNTSSW	Phr peptides	[19]

*Cavity volumes were calculated using the rbcavity routine in rDock [34].

In YejA from *S. meliloti* (SmYejA), the protein crystallized in complex with a pair of copurifying ligands of unknown origin, a dipeptide (SS) and a pentapeptide (GSDVA) [22]. Compared to the undecapeptide bound in EcYejA, these peptides occupy different volumes within the enclosed cavity. Following least-squares superposition of the respective protein backbones, there is little significant overlap of the ligands (Fig. S2B). As shown in Fig. S3, the peptide ligands in SmYejA occupy volumes that are also distinct from those occupied by peptide ligands in a wider set of SBPs of known structure. In SmYejA, there is extensive hydrogen bonding between the protein and the peptide main chains [22]. Similarly to EcYejA, the cavity in SmYejA appears to have the capacity to bind much larger peptide ligands.

### YejA co-purifies with multiple overlapping self-derived peptides

Following the discovery of electron density defining an undecapeptide in the crystals of YejA, we purified fresh batches of protein and used mass spectrometry to characterize co-purifying ligands in more detail. Firstly, ESI-MS was used to determine the molecular mass of native species in the sample. In these experiments, the ion funnel voltage was set at either 100 eV to preserve gas phase protein-peptide complexes or at 200 eV to favour their dissociation. The mass of bound ligands was inferred from the difference in mass of the liganded (native) and unliganded (dissociated) forms. To identify these peptides, we analysed the samples by MALDI-MS/MS. During these experiments, peptide ligands stripped from the YejA protein were fragmented and product ion masses measured. The data from their MS/MS spectra were matched against a protein database to identify the peptide sequences (Table 4).

**Table 4.** Ligand binding in YejA inferred from mass spectrometry

MALDI-MS/MS mass (Da)^∗^	Ligand	Residues	Molecular mass (Da)†	Identified from native ESI-MS‡
1164.59	VLGEPRYAFN	10	1165.31	
1311.66	VLGEPRYAFNF	11	1312.498	YES
1326.64	LGEPRYAFNFN	11	1327.46	YES
1382.70	FAVLGEPRYAFN	12	1383.57	YES
1425.70	VLGEPRYAFNFN	12	1426.59	YES
1562.76	VLGEPRYAFNFNH	13	1563.74	YES
1633.80	AVLGEPRYAFNFNH	14	1634.81	
1643.81	FAVLGEPRYAFNFN	14	1644.85	
1709.83	VLGEPRYAFNFNHF	14	1710.91	
1780.87	FAVLGEPRYAFNFNH	15	1781.99	
1824.86	VLGEPRYAFNFNHFD	15	1826.00	YES
1987.92	VLGEPRYAFNFNHFDY	16	1989.18	YES
1998.97	AFAVLGEPRYAFNFNHF	17	2000.25	
2058.96	AVLGEPRYAFNFNHFDY	17	2060.26	YES
2277.06	AFAVLGEPRYAFNFNHFDY	19	2278.51	YES

*The masses of peptides present in purified YejA preparations was measured using MALDI-MS/MS with fragmentation of peptides and analysis of the products using the Mascot search tool.

†Computed using the ProtParam tool https://web.expasy.org/protparam/

‡The presence of these ligands bound to YejA was confirmed in a number of cases from differences in mass from the unliganded protein in ESI-MS experiments.

At 200 eV, a major peak at 68 383 Da in the ESI-mass spectrum was observed corresponding to the mass of uncomplexed YejA whose calculated mass is 68 382 Da (Fig. S4A). Three further significant peaks are present with masses 1426, 1824 and 1989 Da greater than this mass indicating the presence of 12-mer, 15-mer and 16-mer peptides with sequences VLGEPRYAFNFN, VLGEPRYAFNFNHFD and VLGEPRYAFNFNHFDY, respectively (Fig. S4A). The presence of these species in the 200 eV spectra, suggests these peptides are tightly bound. At the lower ion cone voltage of 100 eV, little or no uncomplexed YejA protein was observed (Fig. S4B) and the 12-mer (calculated mass 69 808 Da), 15-mer (70 206 Da) and 16-mer (70 370 Da) complexes were again seen with YejA-VLGEPRYAFNFN as the dominant species. Other peaks in this spectrum correspond to YejA in complex with the 11-mer LGEPRYAFNFN (69 693 Da), 17-mer AVLGEPRYAFNFNHFDY (70 441 Da) and the 19-mer AFAVLGEPRYAFNFNHFDY (70 659 Da) (Table 4, Fig. S4B). Remarkably, all of the peptides identified are overlapping fragments spanning residues 8–26 in the N-terminal segment of mature YejA. These observations corroborate the identification of the peptide LGEPRYAFNFN in the crystal structure. Furthermore, the crystal structure suggests that the bound undecapeptide could be extended at both its N- and C-termini into unoccupied cavity volume without steric hindrance.

### YejA binds to exogenous LGEPRYAFNFN

To investigate the undecapeptide:YejA interaction further, we tested a synthetic peptide LGEPRYAFNFN (mass 1327 Da). In a first experiment, we combined YejA and the undecapeptide at a 1 : 1 molar ratio and examined the mixture by ESI-MS. As shown in Fig. S5A and B, the major peak observed in the spectrum at 69 709 Da is an exact match to the calculated mass of the YejA:LGEPRYAFNFN complex.

**Fig. 4.**
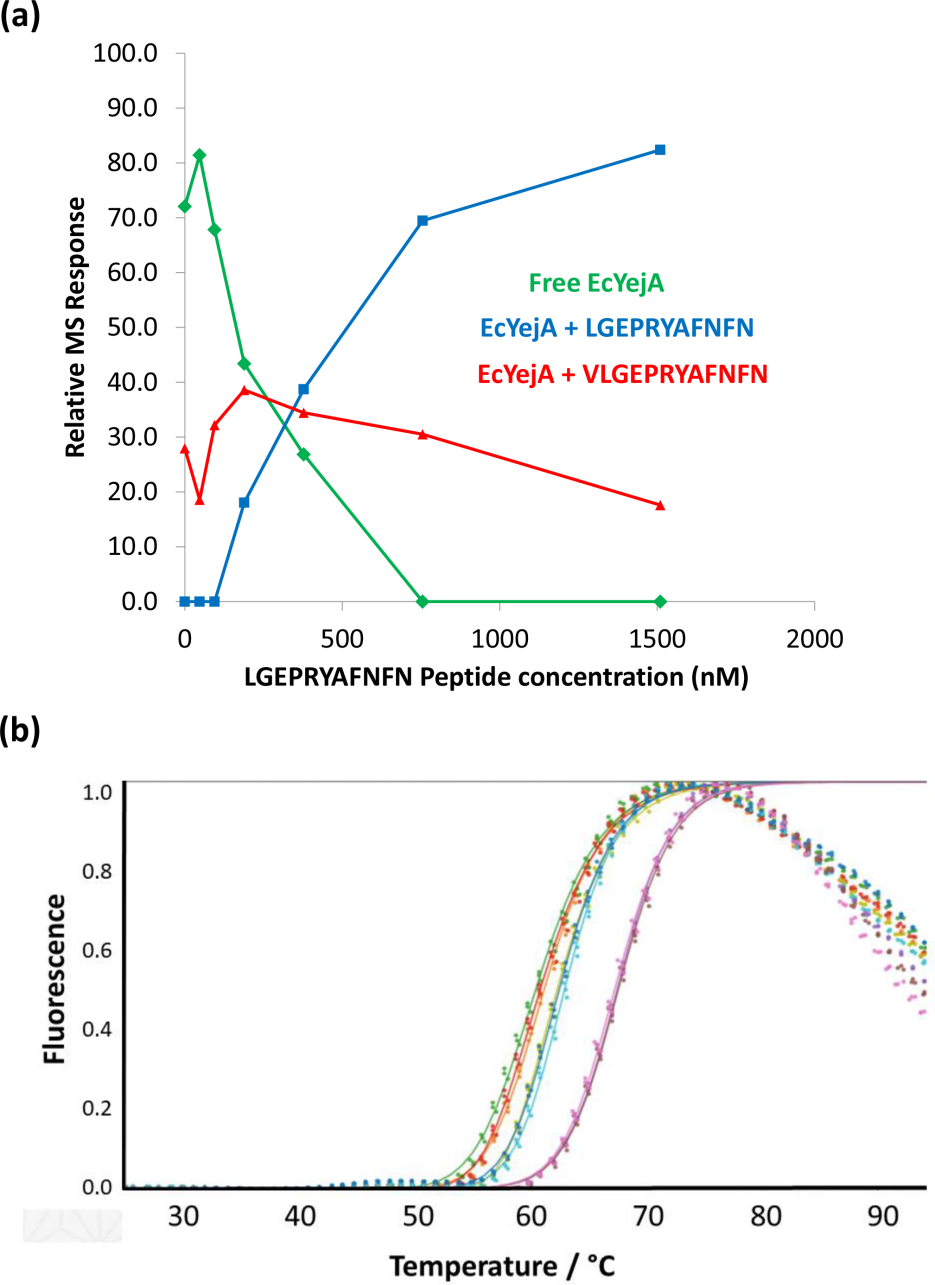
Exogenous peptide binding to YejA. (a) Titration of YejA with LGEPRYAFNFN and analysis by mass spectrometry. The relative intensity of free YejA species decreases as that of the YejA-undecapeptide complex increases during this experiment. This experiment was complicated by the presence in the starting material of YejA-VLGEPRYAFNFN (a+1425 Da species) constituting ~30 % of the YejA species present. (b) Thermal shift assay monitoring the interaction of YejA and the undecapeptide. 0.5 mg ml^−1^ (7.3 µM) EcYejA incubated in the absence of peptide (red, orange, green) has a *T*_m_ of 60.8 °C. In the presence of LGEPRYAFNFN at 0.005 mg ml^−1^ (blue, light blue, lime green) and 0.05 mg ml^−1^ (pink, grey, brown), the *T*_m_ increases to 62.6 and 67.5 °C, respectively.

Next, we titrated newly purified YejA (1.5 µM) with the undecapeptide (0–1.5 µM) monitoring the extent of complex formation using ESI-MS (Fig. 4a). The depletion of the free YejA peak and the appearance of a peak for the YejA:LGEPRYAFNFN complex were plotted as a function of the added undecapeptide concentration. This experiment clearly revealed undecapeptide binding to YejA, however quantitative analysis was complicated by the presence of a peak in the spectrum corresponding to a mass of 69 806 Da in the starting YejA sample implying the persistence of co-purifying ligand with a mass of 1425 Da corresponding to the dodecapeptide VLGEPRYAFNFN described above. Assuming all YejA species respond equally well in the mass spectrometer, the dodecamer-complex accounted for ~30 % of the sample.

The limited solubility of LGEPRYAFNFN hampered attempts to measure the equilibrium association constant for binding of the undecapeptide to YejA by isothermal titration calorimetry. Instead, we monitored binding in a thermal shift assay. In these experiments, YejA was incubated in the presence of the fluorescent dye, SYPRO orange, and fluorescence was recorded as a function of temperature (Fig. 4b). The dye binds to hydrophobic surfaces on the protein, which become exposed as the protein unfolds and its fluorescence is enhanced. Changes in the mid-point unfolding temperature *T*_m_ provide an empirical and semi-quantitative read-out of ligand binding. For free YejA (0.5 mg ml^−1^) the temperature of the mid-point of the fluorescence enhancement (*T*_m_) traces in Fig. 4b, is 61+/-2 °C. The *T*_m_ is shifted to 63, 68 and 74 °C, respectively, in the presence of 0.005, 0.05 and 0.75 mg ml^−1^ (not shown) LGEPRYAFNFN.

### YejA selectively binds self-derived peptides containing a core motif of EPRYAFN

The thermal shift assay described above was next applied to an extended selection of synthetic LGEPRYAFNFN-related peptides including longer and shorter peptides and peptides with amino acid residue substitutions (Table 5). In these experiments, a shift in *T*_m_ was considered significant only if it was greater than 3 °C since the melting temperature of YejA in the absence of added peptide varies by ±2 °C (Fig. 4). The data show a general trend of increasing *T*_m_ as the length of the peptide increases. The longest peptide tested in these thermal shift assays was the 15 residue VLGEPRYAFNFNHFD, which brought about an increase in the YejA melting temperature of 26 °C. The thermal shift data indicate that LGEPR and YAFNFN do not bind YejA. Interestingly, comparison of the data for LGEP**R**YAFNFN and LGEP**A**YAFNFN showed that substitution of the arginine residue in this peptide by alanine led to a of 6 °C drop in *T*_m_ (Table 5). This is consistent with the extensive set of interactions the Arg5 residue makes with the protein in the crystal structure. The peptide LGEPR**W**AFNFN has a 9 °C higher *T*_m_ that LGEPR**Y**AFNFN suggesting that replacement of Tyr6 by tryptophan is well tolerated. A caveat here is that the Trp-containing peptide was present at a higher concentration (Table 5). In the crystal structure, the Tyr6 side chain projects towards the central cavity with one face of its aromatic moiety packing alongside the side chain of the Glu3 side chain of the ligand and the other face packing against YejA residues Tyr51, Phe124, Leu167 and Pro168. Presumably, the Trp6 containing peptide could make more extensive apolar interactions with these residues. Although this study is not exhaustive, taken together with the mass spectrometry data, the thermal shift assay data suggest a core YejA binding motif of EPRYAFN.

**Table 5.** Peptide binding to YejA monitored by thermal shift analysis^*^

Ligand	Ligand concn	Melting temp. change (°C)†
LGEPR	5 mg ml^−1^ (8.76 mM)	0
YAFNFN	2.5 mg ml^−1^ (3.23 mM)	+3
EPRYAFNFN	2.5 mg ml^−1^ (2.16 mM)	+8
GEPRYAFNFN	2.5 mg ml^−1^ (2.06 mM)	+14
LGEPRYAFNFN	0.75 mg ml^−1^ (0.56 mM)	+13
LGEPRWAFNFN	2.5 mg ml^−1^ (1.85 mM)	+22
LGEPAYAFNFN	2.5 mg ml^−1^ (2.01 mM)	+7
LGEPRYAFN	1.25 mg ml^−1^ (1.17 mM)	+17
LGEPRYAFNF	2.5 mg ml^−1^ (2.06 mM)	+20
VLGEPRYAFNF	2.5 mg ml^−1^ (1.9 mM)	+22
VLGEPRYAFNFN	2.5 mg ml^−1^ (1.75 mM)	+21
FAVLGEPRYAFN	5 mg ml^−1^ (3.61 mM)	+19
VLGEPRYAFNFNHFD	2.5 mg ml^−1^ (1.37 mM)	+26

*The EcYejA concentration in all experiments was 0.5 mg ml^−1^ (7.3 µM).

†A shift in melting temperature of greater than or equal to +3 °C was deemed significant, as typical variations in melting temperature of ±1–2 °C were seen in replicate thermal shift runs as shown in Fig. 4b.

In follow-up experiments, with preparations of the ligand depleted refolded protein, we performed thermal shift assays on a series of peptides of different lengths and composition as well as the antimicrobial peptides, polymyxin B, LL-37 and derivatives, melittin, bradykinin and peptides related to microcin C as listed in Table S2. None of these peptides produced a significant positive increase in *T*_m_ in contrast to the undecapeptide control. In these experiments, there is variability in the peptide concentrations tested according to the availability and solubility of the ligands. Although the concentration of ligand would affect the measured Δ*T*_m_ values, all of the ligands here were tested at concentrations well above the *K*_d_ that extracellular solute binding proteins of ABC transporters typically exhibit for their cognate ligands (0.1–10 µM). While the thermal shift experiments provide no evidence for anti-microbial peptide binding to YejA, we cannot conclude that no binding is taking place in the absence of more robust orthogonal assays.

The recombinant YejA protein investigated here was produced in the cytoplasm of *E. coli*. Since the native protein is periplasmic, the purified YejA protein may not have been exposed to its natural ligand(s). We therefore exposed freshly purified recombinant protein to a periplasmic extract of *E. coli* BW25113, a wild-type strain that expresses *lon* and *ompT*. OmpT is an outer-membrane protease that would be expected to influence the peptide composition of the periplasm. Following overnight incubation with the periplasmic extracts, the YejA samples were characterized by ESI- and MALDI-mass spectrometry as before. No peptide ligands other than those derived from the N-terminal segment of YejA itself were identified.

## Concluding remarks

In this study, we have determined the crystal structure of YejA from *E. coli*. Two features of the structure are striking in relation to cluster C solute-binding proteins of known structure. First, YejA has an exceptionally large ligand-binding cavity suggesting it is capable of accommodating large ligands and perhaps even small proteins. This is different to other *E. coli* peptide-binding proteins from the cluster C family, which have small binding pockets, consistent with physiological functions in nutrition. The latter bind peptides that are small enough to diffuse through outer-membrane porins from the external environment. This size limit for diffusion across the *E. coli* outer membrane (five or six residues) implies that for YejA to bind much larger peptides, these must arise in the periplasm either as a result of protein degradation or because they exploit an unknown system that smuggles them across the outer membrane. This is intriguing given the association of YejA with the uptake of antimicrobial peptides and might suggest a novel mechanism of action for this ABC transporter. The enlarged YejA binding pocket contrasts with the findings of a structural study of SapA, the periplasmic component of the Sap (*s*ensitivity to *a*ntimicrobial *p*eptides) ABC transporter [35]. Here a smaller than expected hydrophobic ligand-binding cavity capable of binding only di- or tripeptides was discovered. This would appear to be inconsistent with direct binding of AMPs, yet this system is also implicated in resistance to AMPs through an unknown mechanism.

Second, YejA has a structured N-terminal extension, of 30 or so residues, which adopts elements of secondary structure as part of the core fold. Curiously, this extended segment of the polypeptide gives rise to a set of overlapping peptides that bind with high affinity to the peptide-binding pocket of the protein. This is evidenced in the quality of the undecapeptide electron density in the crystal structure, peaks in the mass spectra of YejA samples consistent with multiple peptide complexes, and the significant upward shifts in the melting temperature of YejA in the presence of these peptides when provided exogenously. While revising this manuscript, we became aware of a recently released coordinate set (7z6f) for a second structure of EcYejA in a different crystal form to that presented here. The two structures are highly similar with an rmsΔof 0.5 Å for 577 matched protein atoms following superposition with Gesamt/SSM routines. Moreover, as observed in the present work, the 7z6f structure also contains a bound peptide, derived from the N-terminal sequence of YejA. This peptide VLGEPRYAFNRN, is one residue longer than the undecapeptide seen the crystal structure reported here, and is among the endogenous YejA ligands discovered by mass spectrometry and studied in the thermal shift assays (Tables 4 and 5). This additional structure provides independent evidence for the natural propensity of YejA to bind a ‘self-derived’ peptide.

It should be noted that in the complexes, the YejA protein is intact – the electron density extends to the N-terminus of the protein, and the mass spectra describe complexes of the full-length protein. Thus, the bound peptides are derived from *trans* cleavage events that take place in at least two places on molecules of YejA that are presumably being turned over in the *E. coli* periplasm. The crystals appeared a month or so after the crystallization experiments were set up. This gives ample time for a proportion of the protein to undergo proteolytic cleavage to form peptides which are subsequently bound by intact YejA molecules, which then form crystals. This peptide binding is selective as the undecapeptide electron density is unambiguous. From the mass-spectrometry analyses performed with fresh YejA preparations, it is clear that cleavage and complex formation takes place during and/or shortly after protein purification. Thus, the presence of the undecapeptide is not an artefact of long-term storage. Clearly these peptides bind to YejA with high affinity and selectivity.

An alignment of 451 YejA protein sequences each representing a single genera across the Proteobacteria is presented in Supplemental File 1 . It is evident that the extended N-terminal element is well maintained and that it is followed by a conserved motif, PXAPKGG (residues 29–35) that in mature YejA appears three residues downstream of the longest peptide detected bound to the protein (the 19-mer ending in HFDY). This sequence may represent a site of auto cleavage though this is a hypothesis that would need to be tested experimentally.

It is tempting to speculate therefore that these ‘*self’* peptides and their transport play a physiological function in *E. coli*. It is well-known that bacteria use exported peptides to send intercellular signals, an example being the deployment of pheromones in plasmid conjugation [36]. In other instances, environmental changes are sensed through extracellular proteolysis of exported peptides, which are reimported to the cytoplasm where they trigger the appropriate response; an example is the Phr peptides, which regulate sporulation in *B. subtilis* [37]. Peptide transporters are key to these systems as the vehicles for peptide import. It is not too far-fetched to conceive of a system in which the signalling peptide is derived from the extracellular component of the peptide transporter itself. Residing on the molecular surface as it does, the N-terminal segment of YejA may become susceptible to proteolytic processing under conditions which pertain when the cell is exposed to anti-microbial peptides (AMPs). In this scenario, the import of the LGEPRYAFNFN-related peptide could conceivably function as a signal to activate downstream AMP resistance pathways. Since the N-terminal extension is not expected to affect the transport of other peptides by this system, it represents an economical way to evolve a new signalling function. Whatever this function is, it must be important as YejA proteins are widely distributed in the Proteobacteria. As their presence confers sensitivity to bacteriocins, this evolutionary retention suggests a key cellular function *in vivo*.

The lack of data demonstrating direct binding of many AMPs (this work) to YejA also suggests something unusual in the function of this ABC transporter. The elegant work of Eswarappa *et al.* [14] in Salmonella, showed that a Δ*yejF* mutant (YejF is the ATPase component) is highly sensitive to AMPs, while the isogenic Δ*yejA* strain has no phenotype. There is no evidence for an additional SBP in *E. coli* or *Salmonella* that might work with the other Yej subunits and account for the lack of phenotype for the Δ*yejA* strain. Together these observations suggest that a simple model in which YejA binds and delivers AMPs to the membrane components of the transporter as part of the resistance mechanism is not correct. However, how the rest of the Yej system functions to confer AMP resistance and what the physiological function of YejA is, remain unresolved but exciting questions with implications for antibiotic resistance in important Gram-negative pathogens.

## Supplementary Data

Supplementary material 1

Supplementary material 2
