# Supplementary material for: Structure and ligand binding in the putative anti-microbial peptide transporter protein, YejA
Source: Microbiology (Reading). 2024 Feb 9;170(2):001430. doi: 10.1099/mic.0.001430 (PMC10924461; doi:10.1099/mic.0.001430)
Supplement: Supplementary material 1 [file mic-170-1430-s001.pdf]

## List of Supplementary Material

**Supplementary Table 1.** Details of Cloning, Expression and Recombinant Protein.

**Supplementary Table 2.** Binding of anti-microbial and related peptides to YejA monitored by TSA.

**Supplementary Figure 1.** Sequence alignment of EcYejA with other *Escherichia coli* K-12 cluster C SBPs.

**Supplementary Figure 2.** Comparison of the YejA structures from *E. coli* and *S. meliloti*.

**Supplementary Figure 3.** Comparison of peptide binding in YejA and other SBPs.

**Supplementary Figure 4.** Mass spectrometry analysis of YejA.

**Supplementary Figure 5.** LGEPRYAFNFN binding to YejA monitored my ESI-MS.

**Supplementary Table 1.** Details of Cloning, Expression and Recombinant Protein.

| | |
|---|---|
| **Source Organism** | Escherichia K12 |
| **DNA Source** | Genomic DNA |
| **EcYejA-F** | 5’ CTTTAAGAAGGAGATATACATATGCAGGCTATCAAGGAAAGCTATG 3’ |
| **EcYejA-R** | 5’ TTGGTCCCTGGAACAGAACCTCGAGCTCTCCCTGTTTGCTGGC 3’ |
| **T7promoter** | 5’ TAATACGACTCACTATAGGG 3’ |
| **T7terminator** | 5’ GCTAGTTATTGCTCAGCGG 3’ |
| **Cloning Vector** | pETFPP_30 |
| **Expression Host** | Escherichia coli BL21(DE3) |
| **Sequence of recombinant protein**[a] | **M**QAIKESYAFAVLGEPRYAFNFNHFDYVNPAAPKGGQITLSALGTFDNFNRYALRGNPGA<br>RTEQLYDTLFTTSDDEPGSYYPLIAESARYADDYSWVEVAINPRARFHDGSPITARDVEF<br>TFQKFMTEGVPQFRLVYKGTTVKAIAPLTVRIELAKPGKEDMLSLFSLPVFPEKYWKDHK<br>LSDPLATPPLASGPYRVTSWKMGQNIVYSRVKDYWAANLPVNRGRWNFDTIRYDYYLDDN<br>VAFEAFKAGAFDLRMENDAKNWATRYTGKNFDKKYIIKDEQKNESAQDTRWLAFNIQRPV<br>FSDRRVREAITLAFDFEWMNKALFYNAWSRTNSYFQNTEYAARNYPDAAELVLLAPMKKD<br>LPSEVFTQIYQPPVSKGDGYDRDNLLKADKLLNEAGWVLKGQQRVNATTGQPLSFELLLP<br>ASSNSQWVLPFQHSLQRLGINMDIRKVDNSQITNRMRSRDYDMMPRVWRAMPWPSSDLQI<br>SWSSEYINSTYNAPGVQSPVIDSLINQIIAAQGNKEKLLPLGRALDRVLTWNYYMLPMWY<br>MAEDRLAWWDKFSQPAVRPIYSLGIDTWWYDVNKAAKLPSASKQGE**LEVLFQPSSGHHHH**<br>**HH** |

[a] Residues in bold were added to the native protein sequence as a result of cloning and tagging

**Supplementary Table 2.** Binding of anti-microbial and related peptides to YejA monitored by TSA

| Ligand | Ligand Concentration | Melting Temperature Change (°C) [a] |
|---|---|---|
| Polymyxin B | 5 mg/ml (3.8 mM) | -1.3 |
| LL-37 | 0.75 mg/ml (0.17 mM) | -0.8 |
| LL 13-37 | 3 mg/ml (0.99 mM) | -1.1 |
| LL 19-29 | 3 mg/ml (2.1 mM) | +0.3 |
| LL 17-32 | 1 mg/ml (0.49 mM) | -3.6 |
| RGDSPASSKP | 0.75 mg/ml (0.75 mM) | +0.2 |
| KKK | 0.75 mg/ml (1.7 mM) | -0.2 |
| RGDS | 0.75 mg/ml (1.7 mM) | +2 |
| GRGDSPK | 0.75 mg/ml (1.0 mM) | -3.17 |
| DWKDDDK | 0.25 mg/ml (0.27 mM) | +0.5 |
| Melittin | 0.75 mg/ml (0.26 mM) | -5.4 |
| KGG | 3 mg/ml (10.1 mM) | -0.7 |
| AK Hydrochloride | 5 mg/ml (19.7 mM) | 0 |
| AQ | 5 mg/ml (21.3 mM) | -0.2 |
| AG | 3 mg/ml (18.3 mM) | -0.1 |
| GPRP amide | 5 mg/ml (11.8 mM) | -0.3 |
| AP Hydrate | 3 mg/ml (16.1 mM) | 0 |
| Bradykinin | 6 mg/ml (5.7 mM) | -1.4 |
| LGEPRYAFNFN | 0.75 mg/ml (0.56 mM) | +13 |
| fMRTGNAD(Dansyl-K)G[b] | 1.5 mg/ml (1.2 mM) | +0.3 |
| fMRTGNAD[b] | 1.5 mg/ml (1.89 mM) | -0.1 |
| MRTGNAD[b] | 1.5 mg/ml (1.96 mM) | -0.3 |

[a] A shift in melting temperature of less than or equal to +3 °C was not deemed significant, as typical variations in melting temperature of ± 1-2 °C were seen in replicate thermal shift runs as shown in Figure 4B.

[b] These peptides are related to microcin C which is a formylated heptapeptide fMRTGNAD linked at its C-terminus to adenosine monophosphate via a phosphoramidate bond.

# Supplementary Figure 1

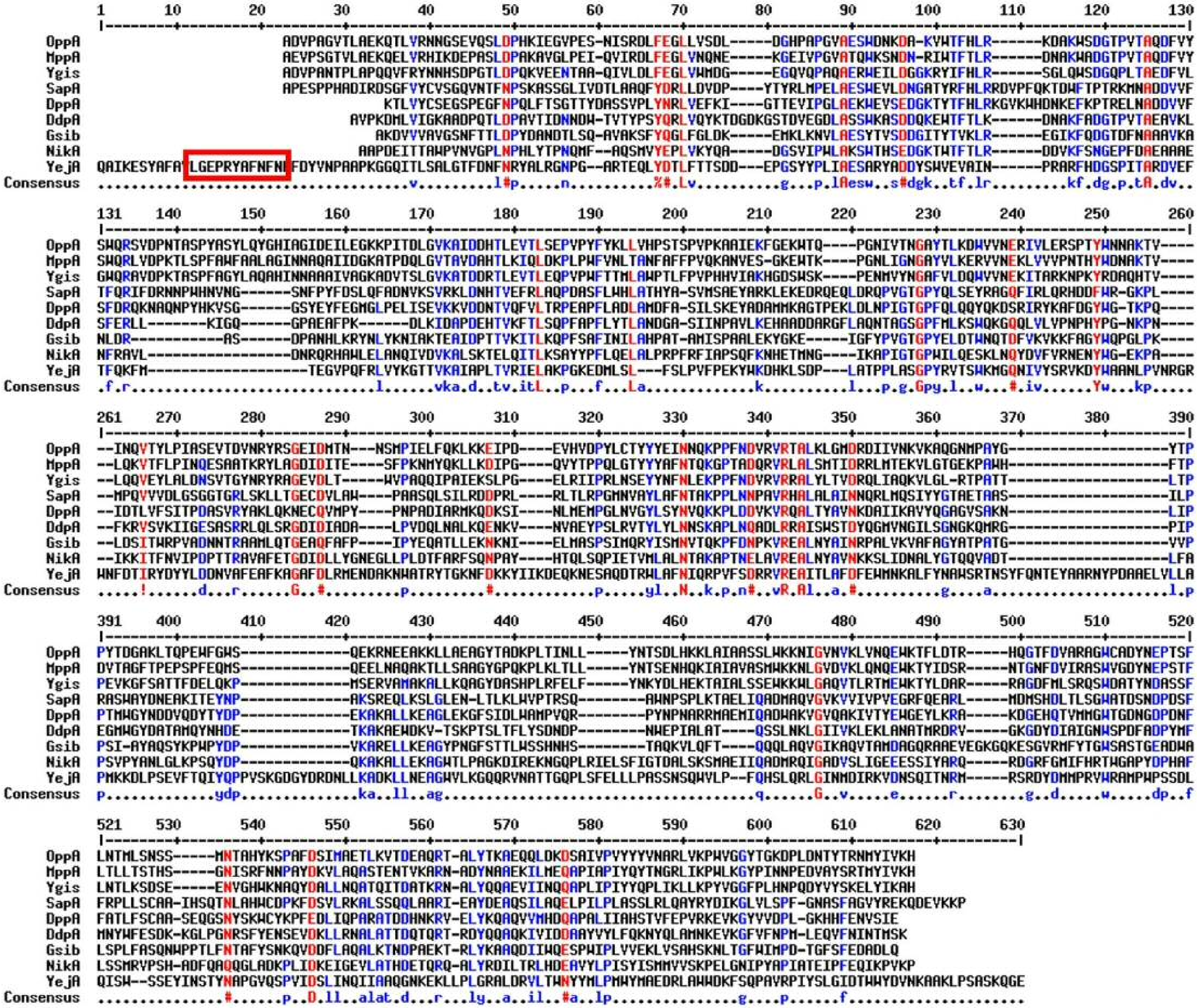

**Supplementary Figure 1**. **Sequence alignment of EcYejA with other *Escherichia coli* K-12 cluster C SBPs**. Native signal peptides of the sequences were removed and sequences were aligned using ClustalW. The unique N-terminal extension of EcYejA is apparent and the residues in the red box correspond to the peptide bound in the crystal structure. Uniprot codes: OppA, P23843; MppA, P77348; YgiS,  Q46863; SapA, Q8X7F4; DppA, P23847; DdpA, P76128; GsiB, P75797; NikA, P33590; YejA, P33913.

# Supplementary Figure 2

**A**

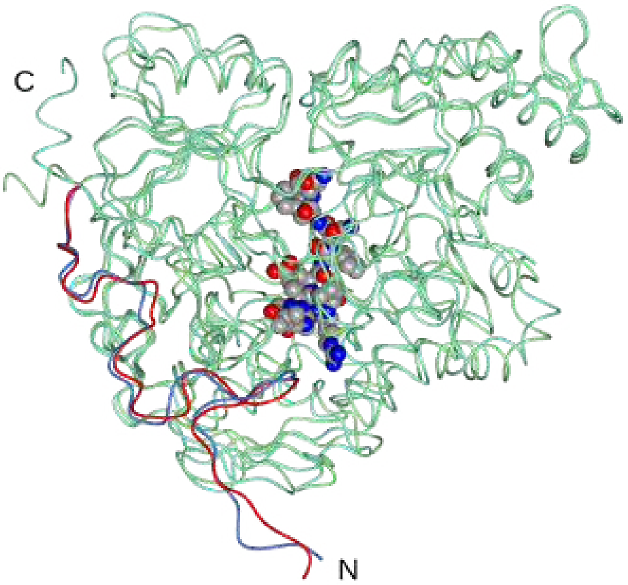


**B**

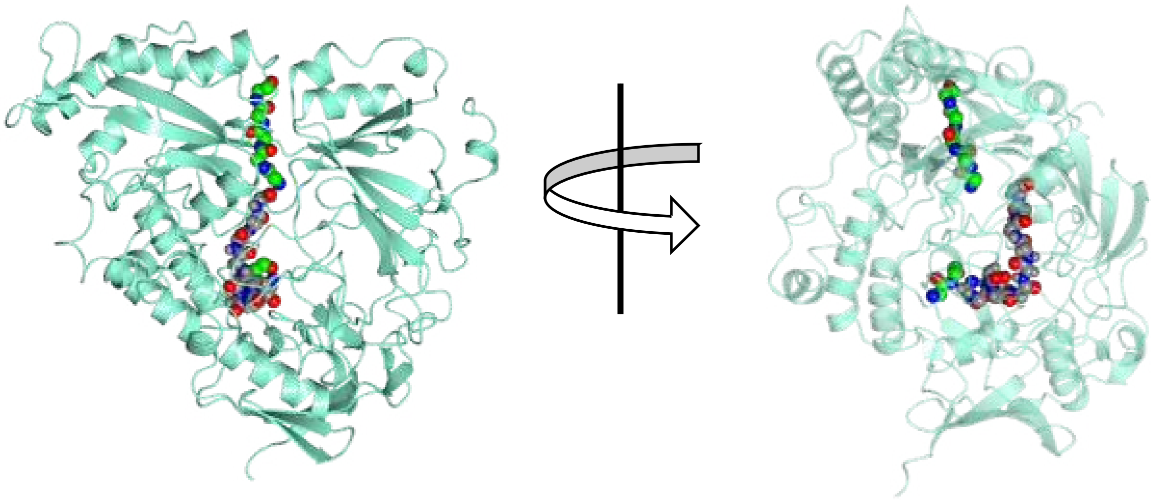

**Supplementary Figure 2.** Comparison of the YejA structures from *E. coli* and *S. meliloti*. **A.** The backbones of the two superposed structures are shown in worm representation with *E. coli* YejA in aquamarine and *S. meliloti* YejA in light green. The N-terminal 35 residues are coloured in red for EcYejA and light blue for SmYejA. The ligand from the *E. coli* YejA structure shown as atomic spheres coloured by element. **B.** The main chain atoms of the bound peptides are shown as sphere in the context of the EcYejA backbone (aquamarine ribbon). The carbon atoms of the undecapeptide ligand in EcYejA are in grey, those of di- and pentapeptide in SmYejA are in green.

# Supplementary Figure 3

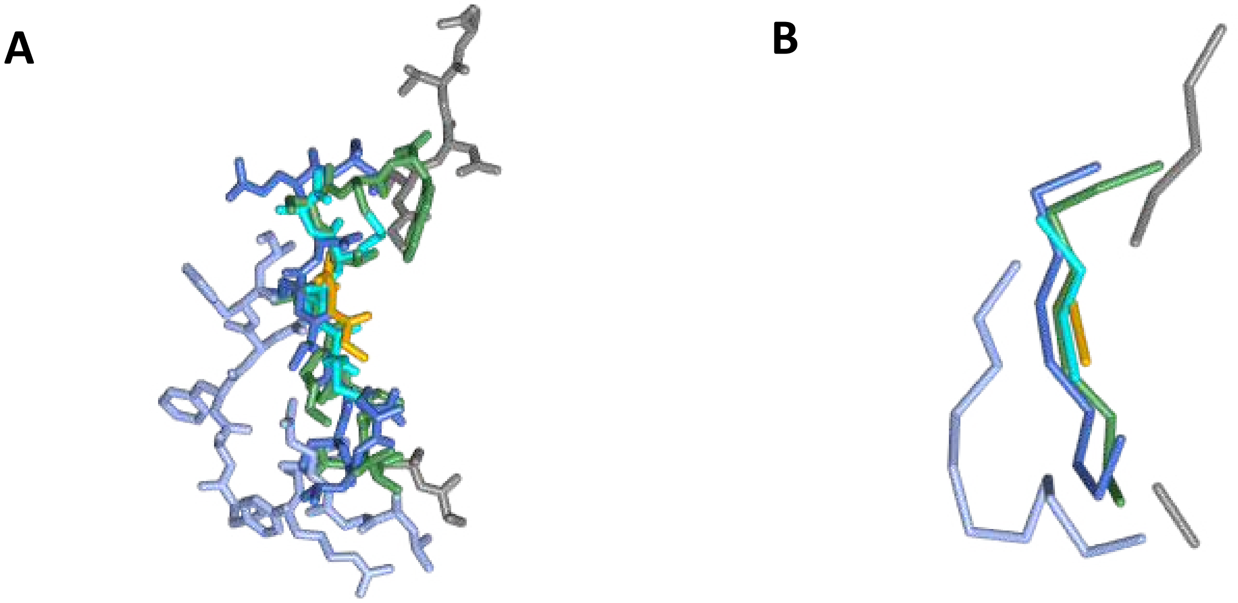


**Supplementary Figure 3. Comparison of peptide binding in YejA and other SBPs. A.** All atom and **B.** Ca atom representations of and Leu-Gly-Glu-Pro-Arg-Tyr-Ala-Phe-Asn-Phe-Asn in EcYejA (ice blue) and the ligand pair Ser-Ser and Gly-Ser-Asp-Val-Ala in SmYejA (1) (grey; 7z8e) together with Gly-Leu in EcDppA (2) (orange; 1dpp), Lys-Lys-Lys-Ala in StOppA (3) (cyan; 1olc), Val-Asp-Ser-Lys-Asn-Thr-Ser-Ser-Trp in BsAppA (4) (lawn green; 1xoc), Ser-Leu-Ser-Gln-Ser-Leu-Ser-Gln-Ser in LlOppA (5) (light blue; 3ryb), The N-termini of the peptides are at the bottom of the image. The displacements of the peptides in the YejA structures relative to one another and the peptides in the other peptide-binding proteins is evident. The structures of the proteins were superposed using the SSM Superpose routine implemented in CCP4mg (6).

# Supplementary Figure 4

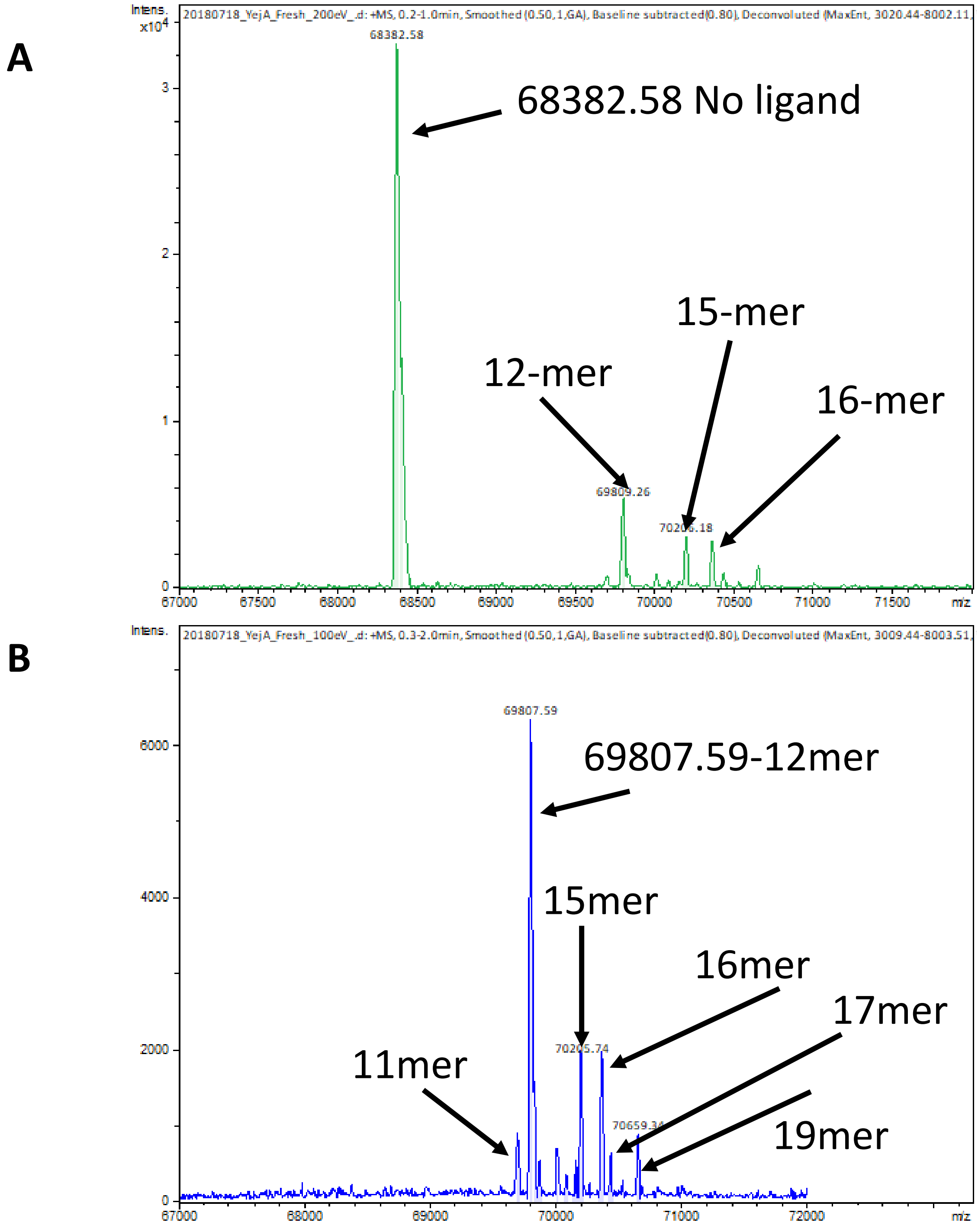


**Supplementary Figure 4. Mass spectrometry analysis of YejA. A.** Native ESI-MS at 200 eV shows uncomplexed YejA (68,382 Da) together with complexes associated with mass increases of 1,426 Da (12-mer, VLGEPRYAFNFN), 1,824 Da (15-mer, VLGEPRYAFNFNHFD) and 1,989 Da (16-mer, VLGEPRYAFNFNHFDY). **B**. Native ESI-MS at 100 eV shows YejA in complex with a wider set of ligands. The principal peak at 69,808 corresponds to the dodecamer complex with VLGEPRYAFNFN. Lower intensity peaks are associated with 15-mer (70,206 Da) and 16-mer above as well as the 17-mer, AVLGEPRYAFNFNHFDY and the 19-mer AFAVLGEPRYAFNFNHFDY (70,661 Da).

# Supplementary Figure 5

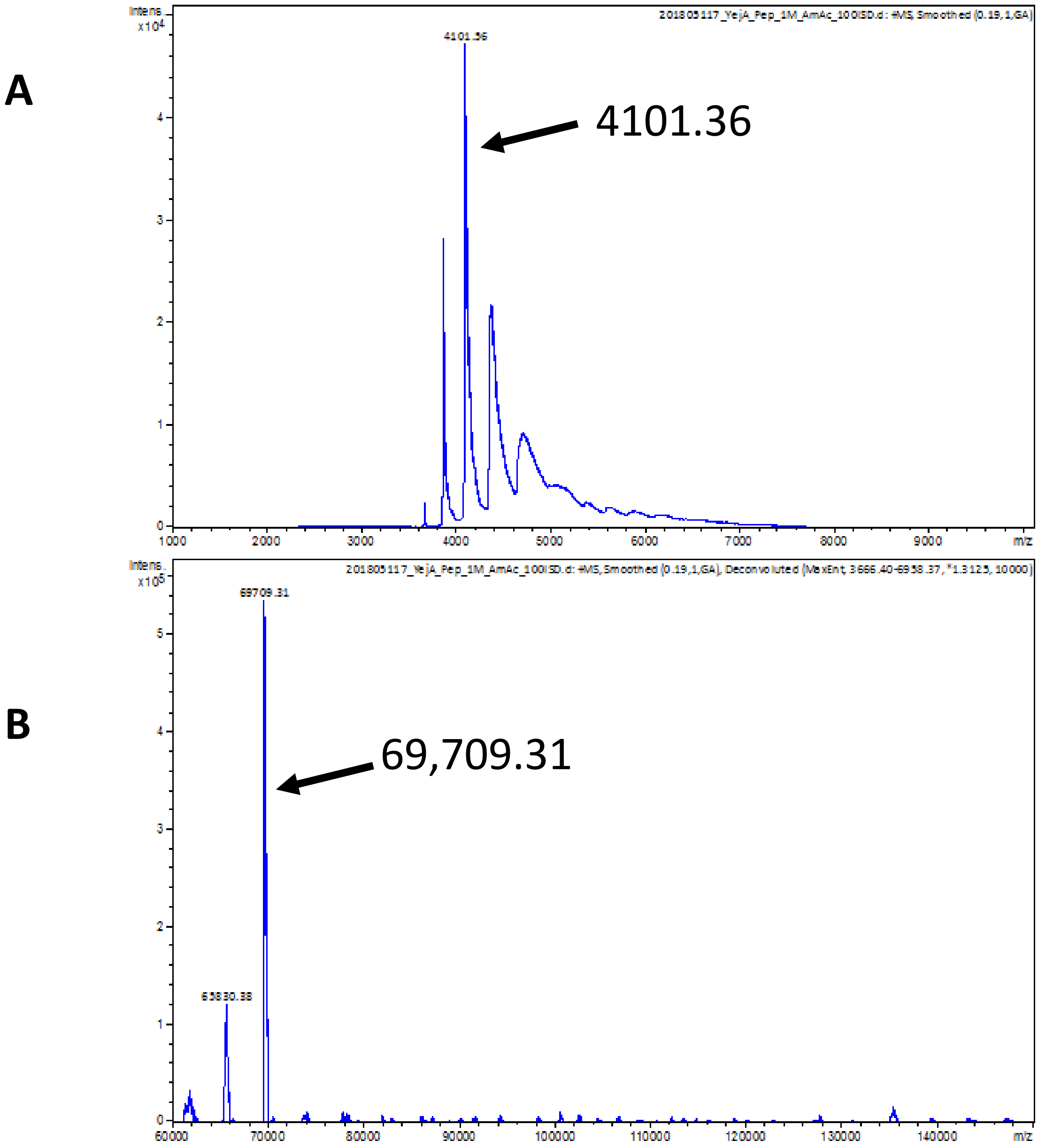


**Supplementary Figure 5. LGEPRYAFNFN binding to YejA monitored my ESI-MS.** The spectra were acquired after mixing EcYejA and LGEPRYAFNFN. **A.** Convoluted (Upper) and deconvoluted (Lower) spectra are shown. The prominent peak at 69,709.31 Da exactly matches the expected mass of the YejA-LGEPRYAFNFN complex.

**Supplementary Literature Cited**


1. Travin, D. Y., Jouan, R., Vigouroux, A., Inaba-Inoue, S., Lachat, J., Haq, F., Timchenko, T., Sutormin, D., Dubiley, S., Beis, K., Morera, S., Severinov, K., and Mergaert, P. (2023) Dual-Uptake Mode of the Antibiotic Phazolicin Prevents Resistance Acquisition by Gram-Negative Bacteria. *mBio* **14**, e0021723
2. Dunten, P., and Mowbray, S. L. (1995) Crystal structure of the dipeptide binding protein from Escherichia coli involved in active transport and chemotaxis. *Protein Sci* **4**, 2327-2334
3. Tame, J. R., Dodson, E. J., Murshudov, G., Higgins, C. F., and Wilkinson, A. J. (1995) The crystal structures of the oligopeptide-binding protein OppA complexed with tripeptide and tetrapeptide ligands. *Structure* **3**, 1395-1406
4. Levdikov, V. M., Blagova, E., Brannigan, J. A., Wright, L., Vagin, A. A., and Wilkinson, A. J. (2005) The structure of the oligopeptide-binding protein, AppA, from Bacillus subtilis in complex with a nonapeptide. *J. Mol. Biol.* **245**, 879-892
5. Berntsson, R. P., Thunnissen, A. M., Poolman, B., and Slotboom, D. J. (2011) Importance of a hydrophobic pocket for peptide binding in lactococcal OppA. *J Bacteriol* **193**, 4254-4256
6. McNicholas, S., Potterton, E., Wilson, K. S., and Noble, M. E. (2011) Presenting your structures: the CCP4mg molecular-graphics software. *Acta Crystallogr D Biol Crystallogr* **67**, 386-394
