# Supplementary material for: Structure and ligand binding in the putative anti-microbial peptide transporter protein, YejA
Source: Microbiology (Reading). 2024 Feb 9;170(2):001430. doi: 10.1099/mic.0.001430 (PMC10924461; doi:10.1099/mic.0.001430)
Supplement: Supplementary material 2 [file mic-170-1430-s002.pdf]

>Escherichia/1-604 coli
-------------MIVRILLLFIALFTFGAQAQAIKESYAFAVLGE--P-RYAFNFNHFDYVNPAAPKGGQI
TLSALG--------TFDNFNRYAL-------RGNPGAR-TEQ-----------LYDTLFTTSDDE-------
--PGSYYPLIAESARYADDY----SWVEVAINPRARFHDGSPITARDVEFTFQKFMTEG-VPQFRL-VYKGT
-TVKAIAPLTVRIELAKP--GKEDMLSLFS-LPVFPEKYWK-------DHKLSDPLAT-PPLASGPYRITSW
KMGQNIVYSRVKDYWAANLPVNRGRWNFDTIRYDYYLDDNVAFEAFK-AGAFDLRMEN--DAKNWATRYS-G
KNFDKKYIIKDEQKN--ESAQDTRWLAFNIQRPVFSDRRVREAITLAFDFEWMNKALFYNAWSRTNSYFQNT
EYAAR----NYPDAAELVLLAPMK----KDLPP------EVFT-QIYQPPVSKGDGYD----RDNLLKADKL
LNEAGWVLKG---QQRVN
ATTG--QPLSFELLLPASS-NS--QWVLPFQHSLQRLGINMDIRKV-DHSQITNRMR---SRDYDMMP----
-RLWRAMPWPS-SDLQISWSSEYIN--STYNAPGVQSPVIDSLINQIIAAQGNKEKLLPLGRALD-RVLTWN
YYMLPMWYMA---------EDRLAWWD-KFSQPAVRPVYS-----LGIDTWWYDVNKAAKLPSARQQGE---
----
>Citrobacter/1-604 farmeri
-------------MIVRILLLFVMLMSAGVQAQAIKESYAFAVLGE--P-KYAFNFDHFDYANPAAPKGGQI
TLSALG--------TFDNFNRYAL-------RGNPAAR-TEQ-----------LYDTLFTTSDDE-------
--PGSYYPLIAESARYAEDY----SWVEITINPRARFHDGSPVMAKDVAFTFNKFMTEG-VPQFRL-VYKGT
-TVKAIAPLTVRMELAKP--GKEDMLSLFS-LPVMPQAYWK-------DHKLSDPLSR-PPLGGGPYRISQW
KMGQYIVYSRVKDYWAANLPVNRGRWNFDVIRYDYYLDDNVAFEAFK-AGAFDLRLES--DAKNWATRYV-G
KNFDNQYIIKDEQKN--ESAQDTRWLAFNIQRPVFSDRRVREAVTLAFDFEWMNKALFYNAWSRANSYFQNT
EYAAR----NYPDAAELILLAPMK----QDLPP------EVFS-QIYQPPVSKGDGYD----RENLLKADKL
LKDAGWELKG---QQRIN
TATG--KPLSFELLISAAS-NS--QWVLPFQHNLQRLGITMTIRQV-DNSQLTNRLR---SRDYDMMP----
-TLWRATPWPS-SDLQISWASEYID--SSYNSPGVKSPVVDKLINQIIAAQGDKNKLLPLGRALD-RVLTWN
YYMLPMWYMA---------EDRLAWWD-KFSRPAIRPIYT-----IGLDNWWYDVNRAAKLPAARRQGD---
----
>Salmonella/1-601 bongori
-------------MIARVMLLLVALVSAGAQAQEIKESDAFAVLGE--P-KYAFNFESFDYVDPAAPKGGQI
TLSAIG--------TFDNFNRYSL-------RGNPGVR-TEA-----------LYDTLFTTSDDE-------
--PGSYYPLIADHARYAADY----SWVEITINPRARFHDGTPITARDVAFTFHKFMTEG-VPQFRL-VYKGT
-TVKAIAPLTVRIELAKP--GKEDMLSLFT-LPIMPEKFWK-------NHKLSDPLST-PPLASGPYRITQW
KMGQYIVYSRVKNYWAANLPVNRGRFNFDTIRYDYYLDDNVAFEAFK-AGAFDLRLEN--DAKNWATRYI-G
KNFDNHYIIKEEQKN--ESAQDTRWLAFNIQRPVFKDRRVREAVTLAFDFEWMNKALFYNAWSRTNSYFQNT
EYAAR----NYPDADELVLLAPMK----KDLPP------EVFT-QIYQPPVSNGDGYD----RENLLKADAL
LTQAGWVING---QQRVN
RVTG--KPLTFELLLPASS-NS--QWVLPFQHNLQRLGITMTIRQV-DNSQLTNRMR---SRDYDMMP----
-RLWRAMPWPS-SDLQISWASEYIN--SSYNAPGVQSPVVDKLIAQIIAAQGDKAKLVPLGRALD-RVLTWN
YYMLPMWYMA---------QDRLAWWD-KFSHPVIRPVYT-----LGLDTWWYDVNKAAKLPAARR------
----
>Lelliottia/1-601 nimipressuralis
-------------MTMRFVLLLMALFSVTCQAQSIKESYAFAVLGE--P-KYEANFTHFDYVNPAAPKGGTL
TLSALG--------TFDNFNRFAL-------RGVAAER-TDA-----------LYDTLFVTSDDE-------
--PGSYYPLIADSARYADDF----SWAEVTLNPRARFHDGSPVKASDVAFTFHKFMTEG-VPQFRL-VFKGA
-TVKAIAPLTVRIELAEP--GKESMLSLFS-LPVMPESFWK-------NHKLSDPIAT-PPLAGGPYRITHW
KMGQYLVYSRVKDYWAANLPVNRGRWNFDTIRYDYYLDDNVAFEAFK-AGAFDLRAEV--SPKNWATRYI-G
KNFSNHFIVKDEQKN--ESAQDTRWLAFNIQRPVFADRRVRQAITLAFDFEWMNKALFYGAYSRTNSYFQNT
EYAAR----QYPDAAELTLLAPLK----AEVPP------EVFT-SIFEPPISKGDGYD----RDNLLKASKL
LDDAGWVLKN---QKRVN
IQTG--KPLSFELLLSSAG-NN--QWVLPFQHNLERLGVTVEIRQV-DNAQITNRMR---NRDYDMMP----
-RVWQAQPWPN-TNLRISWASEYIN--STYNAPGVKSPVIDNLINQIIAAQGDKTKLVPLGRALD-RVLTWN
YYMLPMWFMG---------EDRLAYWD-KFSQPSIRPIYS-----LGLDNWWYDVNKAAKLPAERR------
----
>Leclercia/1-601 pneumoniae
-------------MILRVMMLLLVALSGFCQAQTIKESYAFAVIGE--P-KYAINFDHYDYVNPAAPKGGKA
TLSVIG--------TFDNFNRYAL-------RGVAAER-TES-----------LYDPLFTTSDDE-------
--PGSYYPLVAEFARYADDY----SWVELTINPKARFHDGTPVSARDVEFTFHKFMTEG-VPQFRL-VYKGT
-TVKAIAPLTVRIELAKP--NKEDMLSLFS-LPVMPEKFWK-------NHKLSDPIST-PPLASGPYRITSW
RMGQYVIWSRVKDYWAADLPVNRGRWNFDTLRYDYYLDDNVAFEAFK-AGAFDMRLES--SAKNWATRYT-G

```
RNFERGYIVKDEFKS--DSAQDTRWLAFNIQRPVFTDRRVREAITLAFDFEWMNKALFYGAYSRVNSYFQNT
EYAAT----GYPDAAELTLLAPLK----GEIPA------EVFT-SVYQPPVSKGDGYD----RNNLLKASKL
LDEAGWTLKN---------------------------------------------------------QKRVN
NQTG--KPLTFELLLPSGG-NN--QWVLPFQHNLERLGVTMTIRQV-DNSQITNRMR---SRDYDMIP----
-RLWRAMPWPS-SDLQLSWASAYID--STYNSPGVKSPALDALIARIVDAQGDKEKLLPLGRALD-RVLTWN
YYMLPMWYMA---------QDRVASWD-KFSHPAVRPVYA-----LGFENWWYDVNKAAKLPAERR------
----------------
>Pseudocitrobacter/1-601 corydidari
-------------MFVRMCLVLLALSSFSLGAQTIKESYAFAVIGE--P-KYAINFNHFDYVNPAAPKGGTI
TLSSIG--------TFDNFNRYAL-------RGNPGVR-TES-----------LYDALFTTSDDE-------
--PGSYYPLIAESARYADDY----SWVEVSMNPQARFHDGTPITARDVEFTFHKFMTEG-VPQFRL-VYKGT
-TVKAIAPLTVRFELAKP--GKEDMLSLFS-LPVMPEKFWR-------DHKLSDPLST-PPLASGPYRITDW
RLGQYIVYSREKNYWGANLPVNRGRWNFDTLRYDYYLDDNVAFEAFK-AGAFDLRNES--SAKNWATRYI-G
KNFASGYIVKEAQKN--EAAQDTRWLAFNIQRPVFADRQVREAISMAFDFEWMNKALFYNAYSRPASYFQNT
EYAAR----HYPNAEELTLLAPMK----KDLPP------EVFT-TIYQPPVSNGDGAN----RQNLLKAGDL
LTHAGWVLKD---------------------------------------------------------QQRVN
EKTG--QPLRFELLVASGG-NN--QWVLPFQRNLKRLGVDMSIRQV-DNSQLLSRLR---KRDYDMMP----
-SLWRATPWPS-SDLQISWSSEYIN--STYNAPGVASPVVDSLINQIIQVKGDEKKLLPLGRALD-RVLTWN
FYMLPMWYMA---------EDHVAYWD-KFSHPPIRPLYAS-----GFDNWWYDVNKAAKLPDARR------
----------------
>Phytobacter/1-597 diazotrophicus
--------------FVRLFILSLLLVGTVSHAQTIKENYAFAVIGE--P-KYAINFSHFDYVNPAAPKGGNI
TLSYIG--------TFDSFNRFAL-------RGNAAVR-TES-----------LYDSLFTTSDDE-------
--PGSYYPLIAEVARYADDY----SWVEVSINPRATFHDGSPIRASDVAFTFHKFMTEG-VPQFRL-IYKGT
-TVKAISPLTVRIELGEP--GKENMLSLFS-LPVMPEKFWK-------DHKLSDPLSS-PPLGSGPYRITSW
RMGQYVTYSRVKDYWGATLPVNRGRWNFDSIRYDYYLDDNVAFEAFK-AGAFDMRTEG--SAKNWATRYI-G
SNFDRGYIIKDEQPN--TSAQDTRWLAFNIQRPVFTDRRVREAVSLAFDFEWMNKALFYNAYSRTNSYFQNT
EYAAR----NYPDAAELTLLAPMK----ADLPP------EVFT-SIYTPPVSKGDGYD----RENLLKASEL
LKQAGWEMKD---------------------------------------------------------QKLVN
SQTG--KPFVFEMLLPSGG-NN--QWVLPFQHNLARLGITMEVRQV-DNSQITNRLR---TRDYDMMP----
-RVWRAMPWPS-SDLQISWSSQYID--STYNSPGVKSPVLDKLIAQIIQAQGDKQKLLPLGRALD-RVLTWN
YYMLPMWYMA---------ADQVAYWD-KFSMPGIRPVYS-----LGFDTWWYDVNKAAKLPA---------
----------------
>Pseudescherichia/1-586 vulneris
-------------------------ICFTCQAQAIKENYAFAVIGE--P-KYAIDFTHFDYVNPAAPKGGNV
TLSVIG--------TFDNFNRFAL-------RGNAGVR-TES-----------LYDSLYTTSDDE-------
--PGSYYPLIAEVARYADDY----SWMEIALNPRATFHDGSPITARDVAFTFHKFMTEG-VPQFRL-IYKGT
-TMEAIAPLTVRIKLAKP--GKEDMLSLLT-LPVMPEKFWK-------DHKLSDPLAT-PPLGSGPYRITTW
RMGQYIVYSRVKDYWGANLPVNRGRWNFDSIRYDYYLDDNVAFEAFK-AGAFDMRTEG--DAKNWATRYS-G
KNFSNHQIVKQELKN--ESAQDTRWLAFNNQRPLFSDRRVREALTLAFDFEWMNKALFYNAYSRTNSYFQNT
EYAAR----GYPDAAELTLLAPMK----ADLPP------EVFT-QIYQPPVSKGNGFD----RANLLKASTL
LDDAGWVLKK---------------------------------------------------------QKRVN
AKTG--KPFSFELLLPAGA-NN--LWVMPFQHSLQRLGIVMNVRQV-DNSQLTNRLR---SRDFDMMP----
-RTVRATPWPG-TDLQISWGSQYLD--STYNTPGVSSPVIDALLGQIVANQGNKEKLVPLGRALD-RVLTWN
YYMLPMWFMA---------ADRLAYWD-KFSMPEVRPIYS-----LGFDTWWYDVNKAAKLPA---------
----------------
>Trabulsiella/1-598 guamensis
-------------MILRLSLILIALFSLQGQAQTIKESYAFAVIGE--P-KYAVNFNHFDYVNPAAPKGGAV
TLSEIG--------TFDNFNRYAL-------RGKAGVR-TES-----------LYDSLFTTSDDE-------
--PGSYYPLVAETTRYADNF----SWAEITLNPKARYHDGSPITAKDVAFTFQKFMTEG-VPQFRL-YYKGT
-TVKAIAPLTVRIEMGQP--GKENMLGLFT-LPIMPESFWR-------DHKLSDPLAM-PPLASGPYRITNW
KMGQYIIYSRVKDYWGASLPVNRGRWNFDTIRYDYYLDDNVAFEAFK-AGAFDMRIEG--SAKNWATRYI-G
KNFDNHNIVMDAQKN--TSAQNTRWLAFNVQRPVFSDRKVREAITLAFDFEWMNKALFYSAYSRANSYFQNT
EYAAR----NYPDADELVLLAPMK----PDLPP------EVFT-SIYQPPVSDGGGFD----RDNLLKASKL
LDEAGWPLKG---------------------------------------------------------QQRIN
AKTG--KPLRFELLLGSGG-NN--QWVLPFQHSLSRLGVTLEIRQV-DNAQLNNRLR---SRDYDMMP----
-VLWQAAPWPS-SDLQIAWASQYVD--STYNAPGVKNPVIDKLIAEIIRWQGNKEKLLPLGRALD-RVLTWN
YYQLPMWYMA---------EDRIAYWD-KFSHPVMRPVYS-----LGFDTWWYDVNKAAKLPA---------
```

```
----------------
>Kluyvera/1-606 ascorbata
-------------MVARVFLPLLMLFSLSLQAQTIKESYAFAVIGE--P-KYAVDFNHFDYVNPAAPKGGSV
TLAALG--------TFDNFNRFAL-------RGNAAVR-TDS-----------LYDALFTTSDDE-------
--PGSYYPLVADRARYADDY----SWVELTINPRARFHDGSPITARDVAFTFNKFMTEG-VPQFRL-VYKGT
-TVKAIAPLTVRIELAKP--GKEDMLSLFT-LPILPEKFWK-------DHNLSEPLST-PPLASGPYRISDW
RMGQYIVYQRVKDYWGADLPVNRGRWNFDTIRYDYYLDDNVAFEAFK-AGAYDVRTES--SAKNWATRYI-G
KNFASGYIVKDEQKN--NAAQDTRWLAFNIQRPVFTDRRVREAVSMAFDFEWMNKALFYNAYSRANSYFQNT
EYAAR----GYPDADELTLLAPLK----SELPP------EVFT-SIYQPTTSDGHGFD----RDNLLKAGNL
LTDAGWVLKG---------------------------------------------------------QQRVN
AKTG--QPLRFELLLPSGG-ND--QWVMPFQRNLQRLGVTMDIRQV-DISQITNRMR---KRDYDMMP----
-RLWRAMPWPS-SDLQISWSSEYIN--SSYNSPGVASPAIDSLINQIIRWQGNKQKLLPLGRAID-RVLTWN
YYMLPMWYMS---------GSRTAYWD-KFSYPSVHPLYASGTDAPGFDTWWYNVNKAAKLPASRR------
----------------
>Yokenella/1-598 regensburgei
-------------MIVRLLILMLTLFCMQSHAQAIKESYAFSVIGE--P-KYAVDFHHFDYVNPAAPKGGSV
TLAELG--------TFDNFNRYAL-------RGKAGVR-TES-----------LYDTLFTTSDDE-------
--PGSYYPLVADMARYAADF----SWAEITINPRARYHDGSPITAKDVAFTFNKFMTEG-VPQFRL-IYKGT
-TVKAIAPLTVRIALAKP--DKENMLSLLS-LPIMPENFWR-------NHKLSDPLST-PPPASGPYKITQW
KMGQYIVYSRVKDYWAANLPVNRGRWNFDTIRYDYYLDDNVAFEAFK-AGAFDMRVES--SAKNWATRYI-G
KNFANHNIVKDEQKN--TSAQDTRWLAFNIQRPVFADRRVREAVTLAFDFEWMNKALFYSAYSRANSYFQNT
EYAAR----NYPDADELTLLAPMK----NELPP------EVFT-SIYQPPVSNGDGFD----RENLLKANQL
LDAAGWPLKG---------------------------------------------------------KQRVN
AQTG--QPLRFELLLSAGG-NN--QWVLPFQHSLSRIGVTLDIRQI-DNAQLNNRLR---SRDYDMMP----
-VLWQATPWPS-SSLRIAWASEYID--STYNAPGVKNPVVDKLIGDIIRWQGNKQKLLPLGRALD-RVLTWN
YYQLPMWYMA---------EDRIAYWD-KFSHPAVRPVYS-----LGFDTWWYDVNKAAKLPA---------
----------------
>Enterobacter/1-601 chuandaensis
-------------MLMRFMLLWLAVVSLSSQAQTIKENIAFAVIGE--P-KYAVNFTHFDYVNPAAPKGGKV
TLSATG--------TFDNFNRFAL-------RGVAAAR-TES-----------LYDTLFVTSDDE-------
--PGSYYPLIADNVRYADNF----SWAEITLNPRARFHDGTPVKASDVAFTFHKFMTEG-VPQFRI-VYKGT
-TVRAIAPLTVRIELSEP--NKENMLSLFS-LPVMPESFWK-------NHKLSDPLST-PPLAGGPYRITDW
RMGQYVVYSRVKDYWAANLPVNRGRWNFDTLRYDYYLDDNVAFEAFK-AGAFDLRVEN--SAKNWATRYI-G
KNFAKGYIVKDEHKN--ESAQDTRWLAFNIQRPVFTDRRVREAISLAFDFEWMNKALFYGAYSRANSYFQNT
EYAAR----DYPKADELILLAPLK----AEVPP------EVFT-TVFQPPASKGDGYD----RDNLLKASKL
LDEAGWQLKN---------------------------------------------------------QKRVD
AKTG--KPLSFELLLSSGG-ND--QWVLPFKHNLARLGITMNIRQV-DNAQITNRMR---SRDYDMMQ----
-RLWPAQPWPS-SDLQISWASSYID--SSYNAPGVKSPAIDTLIAKIVAAQGDKEKLLPLGRALD-RVLTWN
YYMLPMWYMG---------EDRLARWD-KFSVPTVRPVYS-----LGFDTWWYDVNKAARLPAERR------
----------------
>Kosakonia/1-600 sacchari
--------------FVRISALLLAFVGLTCQAQTIKESYSFAVIGE--P-KYAINFSHFDYVNPAAPKGGNV
TLSVIG--------TFDNFNRYAL-------RGNPAVR-TEA-----------LYDALFTTSDDE-------
--PGSYYPLVAEMARYADDY----SWMEISINPRAIFHDGSPIRASDVAFTFQKFMTEG-VPQFRL-VYKGT
-TVKAISPLTVRIVLAKP--GKEDMLSLLT-LPVMPEKFWK-------DHKLSDPLAQ-PPLGSGPYRISTW
RMGQYIAYTRVRDYWAANLPVNRGRWNFDNIRYDYYLDDNVAFEAFK-AGAYDLRSEG--DAKNWATRYI-G
SNFSRGYIVKDEQPN--TSAQDTRWLAFNTTRPVFSDRRVREAVSLAFDFEWMNKALFYGAYSRANSYFQNT
EYAAR----NYPDADELTLLAPLK----AQVPP------EVFT-SIYSPAKSNADGYD----RENLLKADKL
LTESGWILKN---------------------------------------------------------QQRVN
AKTG--KPFTFELLLPSGG-NT--QWVLPFQHNLARLGINMDVRQV-DNSQATNRLR---SRDYDMMP----
-SLWRAQPWPS-GDLQISWSSQYID--SSYNAAGVKNPAVDNLIAQINRWQGNKEKLLPLGRALD-RVLTWN
FYMLPMWYMA---------ADRVAYWD-KFSMPGIRPVYS-----LGFDTWWYDVNKAAKLPAARR------
----------------
>Huaxiibacter/1-601 chinensis
-------------MLMRFVLSLLTLFSLTCQAQAIKESYAFAVLGE--P-KYAAHFTHFDYVNPAAPKGGSV
TLSSLG--------TFDNFNRYAL-------RGVPAQR-TDA-----------LYDSLFVTSDDE-------
--PGSYYPLVAEMARYATDF----SWAEVTLNPKARFHDGTPVKASDVAFTFQKFMTEG-VPQFRV-VFKGT
-TIKAIAPLTVRIELAKP--SKEDMLSLFS-LPVMPESFWK-------DHKLSDPLST-PPLAGGPYRITRW
```

RMGQYVIYSRVKDYWAADLPVNRGRWNFDTLRYDYYLDDNVAFEAFK-AGAFDFRQEV--SAKNWATRYI-G
KNFDRHYIVKEAQPN--TSAQDTRWLAFNIQRPFFADRRVRQAITLAFDFDWMNKALFYGAYSRTSSYFQNT
EYAAR----HYPDAAELILLAPLK----AEVPP------EAFT-TIFEPPASRADGYD----RENLLKASKL
LDEAGWVLKN---QQRVN
AQTG--KPLRFELLLASGG-NN--QWVLPFQHNLERLGVKMDIRQI-DTAQITNRLR---KRDYDMMP----
-TLWQAQPWPS-SSLQISWASEYIN--SSYNAPGVSSPVLDTLIAKIIAAQGDKQKLLPLGRVLD-RVLTWN
YYMLPMWFMG---------EDRLAYWD-KFSQPAVRPVYS-----IGFDNWWYDVNKAAKLPAERR------
----
>Silvania/1-601 hatchlandensis
-------------MIVRVVMVLIAMISGLCQAQTIKESFAFAVIGE--P-KYAINFDHYDYVNPAAPKGGSV
TLSANG--------TFDNFNRYAL-------RGVAAER-TDA-----------LYDTLFTTSDDE-------
--PGSYYPLVAEMARYADDF----SWMELTLNPLARFHDGTPVKASDVAFTFHKFMTEG-VPQFRL-VYKGA
-NVTAIAPLTVRITLTHP--NKEDMLSLLT-LPVMPEKFWK-------DHKLSDPLST-PPLAGGPYRITHW
RMGQYLIYSRVKDYWAANLPVNRGRWNFDTLRYDYYLDDNVAFEAFK-AGAFDMRIES--SAKNWATRYS-G
KNFTRGYIVKEAFKN--DSAQDTRWLAFNIQRPVFSDSRVRQAITLAFDFEWMNKALFYGAYSRVNSYFQNT
EYAAH----GYPDAAELTLLAPLK----GAVPP------EVFT-TLYQPPRSTGDGFD----RDNLLQASKL
LDEAGWEIKN---QKRIN
TQTG--KPLSFELLLPSGG-NN--QWVLPFQHNLERLGITLDIRQV-DNSQITNRMR---NRDYDMMP----
-RLWRAMPWPS-TDLQISWASQYID--STYNAPGVKNPAVDALIAQIVAAQGNKEKLLPLGRALD-RVLTWN
HYMLPMWFMG---------QDRVAHWD-KFSLPAQRPVYS-----LGFENWWYDVNKAAKLPAERR------
----
>Superficieibacter/1-601 electus
-------------MLVRLCFLLTALFSLAVQAQTIKESTAFAVIGE--P-KYAVNFTHFDYVNPAAPKGGAV
TIAAIG--------TFDNFNRFAT-------RGNPAER-TGA-----------LYDTLFTTSDDE-------
--PGSYYPLVAESARYADDF----SWVELNITPRARFHDGTPITAQDVVFTFNKFMTEG-VPQFRL-IYKGT
-TFKAIAPLTVRIELAQP--GKENMLSLFS-LPVMPEKFWR-------NHKLNEPLSR-PPLASGPYKISAW
RMGQYVTYSRVKDYWAADLPVNRGRWNFDTLRYDYYLDDNVAFEAFK-AGAFDFRTEG--SAKNWATRYI-G
KNFANHNIVKEEMES--DAAQDARWLAFNIQRPVFSDRKVREAISLAFDFEWMNKSLFYNAYSRPDSYFLNT
KYAAR----HYPDADELTLLAPMK----EDLPP------EVFT-SVYQPAKSNADGYD----RENLLKASDL
LTQAGWVMKG---QQRVN
SQTG--KPLRFELLLPSGG-ND--QWVLAFKHNLARIGVIMDIRQI-DNSQLTSRMR---NRDYDMMP----
-RLVQAMPWPS-SDLQITWASEYIN--SSYNAPGVQSPVIDALIAQIIRAHGNEEKLLPLGRALD-RVLTWN
YYMLPMWYMA---------KDRLAYWD-KFSHPATRPLYS-----VGFDNWWYDVNKAAKLPAARR------
----
>Scandinavium/1-581 manionii
----------------------------------IKESYAFAVIGE--P-KYAFNFSHYDYVNPAAPKGGAV
TLSALG--------TFDNFNRFAL-------RGNAAER-TDA-----------LYDALFTTSDDE-------
--PGSYYPLIADMARYAGDF----SWAEVTINPRARFHDGSKVTAEDVAFTFHKFMTEG-VPQFRL-IYKGT
-TVKAIAPLTVRIELAKP--SKEDMLSLFT-LPVMPEKFWR-------NHKLSDPLST-PPLANGPYKITAW
KMGQYVVYSRVKDYWAADLPVNRGRWNFDTIRYDYYLDDNVAFEAFK-AGAFDFRTET--SPKNWATRYI-G
KNFSNHFIVKEEEKN--NSAQDTRWLTFNTQRPVFADRTVREAIGLAFDFEWMNKALFYGAYSRTNSYFQNT
EYAAR----GHPDADELVLLAPMK----KDLPP------EVFT-SIYSPPKSNGNGFD----RANLLKADKL
LTDAGWVLKG---QQRVN
AKTG--KPLQFELLLPSAG-NN--QWVMPFQHNLKRLGVMMDVRQV-DLSQLTNRMR---TRDYDMMP----
-RPWRAMPWPSGSDLQISWSSEYIN--SSYNGPGVSSPVIDNLIKQMIHWEGNKQKLLPLGRALD-RVLTWN
YYQLPMWFMA---------SDRMAYWD-KFSHPAVRPIYS-----LGFDNWWYDVNKAAKLPEARR------
----
>Atlantibacter/1-582 subterranea
--------------------------------QEIKESYSLAVLGE--P-KYAVDFTHFDYVDPAAPKGGNI
TLSSIG--------TFDNFNRYAL-------RGNAAVR-TEN-----------LYDALFTTSDDE-------
--PASYYPLIAERVRYPDNY----AWAEVEMNPRARFHDGSPITADDVAFTFSKFMTEG-VPQFRI-YYKGA
-TVKAIAPLTVRIELATP--GKENLLGLFS-LPVMPKKYWK-------DHKLSDPLST-PPLASGPYKITAW
KMGQYVTYSRVKDYWGANLPVNRGRWNFDTIRYDYYLDDNVAFEAFK-AGAFDFRSEV--SAKNWATRYI-G
KNFDNKYIVKDEKKS--EAATDTRWLAFNIQRPQFADRRVREAITLAFDFEWMNKALFYNAYSRANSYFQNT
EYAAR----DYPKADELVLLAPMK----ADLPP------EVFT-SIYQPPVSKADGFD----RDNLLKAIDL
LKQAGWELKN---QKLVN
VKTG--QPFSFELLLSSGG-NN--QWVLPFQHNLERLGITLNIRQV-DNSQITNRLR---TRDYDMMP----
-SLWRAMPWPS-SDLQISWASEYID--STYNAPGVKSLVVDKLITQIMAHQGDKEKLLPLGRALD-RVLTWN

FYMLPMWYMS---------SDRYAYWD-KFSHPAVAPVYS-----LGFDTWWYDVNRAEKLPAARR------
----
>Klebsiella/1-598 spallanzanii
-------------MFVRISLILLALFSLQSRAQTIKESDTFAIIGE--P-KYVAGFEHYDYANPDAPKGGAI
TLAAIG--------TFDNFNRYAM-------RGNPGIR-TET-----------LYDTLFTTSDDE-------
--PGSYYPLIAEHTRYADDF----SWMEITLNPRARYHDGSPITASDVAFTFQKFMTEG-VPQFRL-YYKGT
-TVKAIAPLTIRIDLAKP--GKENMLSLMT-LPVMPETFWK-------SHKLSDPLSA-PPLASGPYRVTAW
RMGQYITYSRVKNYWAADLPVNRGRWNFDTLRYDYYLDDNVAFEAFK-AGAVDRREEN--VAKNWATRYV-G
RNFTHGYIIKDEYTN--TSAQNTQWLAFNIQRPVFSDRRVREAITLAFDFEWMNKALFYNSYSRANSFFQNT
EYAAR----NYPDADELALLTPLK----KEIPA------EVFT-QIYQPPVSSGDGFE----RTNLLKADAI
LNQAGWVVKN---QRRVN
AQSG--KPLRFELLLPSGS-SD--RWVLPFQHNLQRLGIVMDIRQV-DNSQYSNRKR---SRDYDMMP----
-NVWRATPWPG-TDLQISWDSEYIN--SSYNASGVQSPAVDQLIAQIIRWQGNKEKLLPLGRALD-RVLTWN
YYMLPMWYMA---------QDRTAYWD-KFSFPQTRAVYS-----SGFENWWYDANKAARLPA---------
----
>Cedecea/1-600 lapagei
-----------------LMLLCLITFSLMPRAAEINESYSFAVLGE--P-KYAVNFTQFDYVNPAAPKGGNI
TLSAIG--------TFDNFNRYAM-------RGNPGVR-TDT-----------LYDTLFTTSDDE-------
--PGSYYPLIAEMARYPSNF----AWAEVSINPRARYQDGSPITAQDVAFTFNKFMTEG-VPQFRL-IYKGA
-TVRAIAPLTVRIELATP--SKDRMLGLFS-LPVFPEKFWK-------DHKLSDPLSS-PPLSSGPYKITDW
KMGQYITYSRVRDYWAANLPVNRGRWNFNTIRYDYYLDDDVAFEAFK-AGAFDFRSEV--SAKNWATRYT-G
KNFSNHYIVKDEQKN--ESAQDARWLAFNIQRPIFSDRRIREAIGLAFDFEWMNKALFYGAYSRANSYFQNT
EYAAR----GYPDADELMLLAPMK----KDLPP------EVFT-SIYNPPKSDGRGFD----RENLLKALSL
LKEAGWELKD---QKLVN
VTTG--KPFVFELLVGTAG-NT--QWILPFQRNLQRLGITMNIRQV-DNSQLSSRLR---SRDYDMMP----
-RVYSAMPYPS-PDLQIIWASEYIN--SSYNAPGVQSPVIDKLIGMIIASQGDKKKLLPLGRALD-RVLTWN
NYMLPMWYMS---------ADRLAWWN-KFSRPAIHPIYN-----LGFDNWWYDVNKAAKLPADRRQGE---
----
>Raoultella/1-598 terrigena
-------------MFARICLILIIFMSLNGQAQAVKESHSFAIIGE--P-KYPDGFSHFAYANPAAPKGGSI
TLASIG--------TFDNFNRYAL-------RGNPGVR-TDA-----------LYDTLFTTSDDE-------
--AGSYYPLIADGARYADDF----SWVEVTLNPQARFHDGTPIRASDVAFTFQKFMTEG-VPQFRL-FYKGT
-RVKAIAPLTVRIELGQA--GKENMLSLFT-LPVMPEKFWR-------DHKLSDPLST-PPLASGPYRITDW
RMGQYIVYSRVKTYWAANLAVNRGRWNFDTLRYDYYLDDNVAFEAFK-AGAVDRREET--MAKNWATRYV-G
RNFTRGYIVKDEYQN--TSAQDTQWLAFNIQRPVFADRQVREAITLAFDFEWMNKALFYNAYSRANSYFQNT
EYAAR----TAPDADELALLTPMK----KDLPP------EVFT-AIYQPPQSDGGGFD----RANLLKADSL
LNRAGWVVKN---QRRVN
GKTG--KPLRFELLLPAGG-ND--RWVLPFQHNLRRLGITMDIRQV-DNSQYSNRRR---SRDYDMMP----
-MVWRATPWPA-TDLQVSWDSAYIH--SSYNAPGVQSPVVDSLIAQIIRWQGNEQKLLPLGRALD-RVLTWN
YYMLPMWYMA---------QDRTARWD-KFSFPPTRPVYS-----SGFDSWWYDVNKAAKLPA---------
----
>Pseudenterobacter/1-600 timonensis
--------------MLRFICLLLAFSALPLQAQNLRQGYAFAVLGE--P-KYPASFSHFDYVNPAAPKGGNI
TLSATG--------TFDNFNRYAL-------RGVAAAR-TES-----------LYDTLYVTSDDE-------
--PGSYYPLLALGARYADDF----SWAEITLNPQARFHDGSPVTARDVAFTFHKFMTEG-VPQFRL-VYKGA
-TVTAVAPLTVRITLAEP--NKENMLSLFT-LPVMPASFWA-------HHKLSDPLAT-PPLAGGPYRITSW
KMGQYVIYSRVKNYWAANLPVNRGRWNFDTLRYDYYLDDNVAFEAFK-AGAFDLRQEG--DAKNWATRYV-G
KNFNRGYILKEEPKN--ESAQDTRWLAFNIQRPVFADRRVRQAITLAFDFEWMNKALFYGAYSRASSYFQNT
DYAAR----GKPDSAELALLEPFR----AELPP------EIFT-TPFRAPASAGDGFD----RANLLAASRL
LDSAGWVIKN---QRRVN
AKTG--KPLSFELLLSSGS-NN--QWVLPFQHNLARLGIRMSIRQV-DRSQLTNRMR---SRDYDMMP----
-RLWQAQPWPS-SDLSISWASEYIN--SSYNAPGVQSPVIDALIAKITAAQGDEAALLPLGRALD-RVLTWN
YYMLPMWYMG---------EDRVARWD-KFSRPAVRPIYS-----LGFDTWWYDARKAARLPAGQR------
----
>Franconibacter/1-601 daqui
-------------MFARALVVLLILYGAVCQAEPIKESYSLAILGE--P-KYAVNFTHFDYVNPAAPKGGNV
TLSAIG--------TFDNFNRYAL-------RGNAAIR-TEA-----------LYDPLFTTSDDE-------
--PGSYYPLVGEMLRYSPDF----AWAEVEINPRARFHDGSSITADDVAFTFHKFMTEG-VPQFRL-YYKGA

```
-TVKAIAPLTVRIELAKP--GKEDMLGLLS-LPVMPHKYWK-------DHKLSDPLSR-PPLASGPYQITDF
RMGQYVVYSRVKDYWAANLPVNQGRWNFDTLRYDYYLDDNVAFEAFK-AGAFDFRLES--SPKNWATRYN-G
KNFDSRYIVKEEQKN--ESAQDTRWLAFNIQRPLFADRRVREAITLAFDFEWMNKALFYGAYSRPVSYFQNT
EYAAR----NYPDAAELTLLAPLK----SDVPP------EVFT-TIYQPPVSHGDGFD----RNHLLKALTL
LKAAGWTLKD---------------------------------------------------------QKLVN
EKTG--QPFSFELLLSAAG-NN--QWVLPFQHNLERLGITMSIRQV-DNSQLTNRLR---KRDYDMIP----
-TLYRAMPWPS-GDLQISWASAYID--STYNSPGVQNPAVDKLIAQILENQGNKEKLLPLGRALD-RVLTWN
YYMLPMWYTS---------ADRFARWD-KFSRPGIHPVYS-----LGFDTWWYDVNKAAKLPAARR------
----------------
>Dryocola/1-580 clanedunensis
-------------------------------AENIKESYSFAVLGE--P-KYAVNFTHFDYVNPAAPKGGNI
TLSAIG--------TFDNFNRYAL-------RGNPGVR-TET-----------LYDPLFTTSDDE-------
--PGSYYPLVGEMARYPDNF----AWAEIEINPKARFHDGSPVTAKDVAFTFNKFMTEG-VPQFRL-IYKGA
-KVKAIAPLTVRIELAQP--SKERMLGLFS-LPVFPEKFWK-------DHKLSDPLSR-PPLASGPYKITAW
RMGQYITYSRVRDYWAANLPVNRGRWNFDSIRYDYYLDDNVAFEAFK-AGAFDFRAES--SAKNWATRYT-G
KNFANHFIVKEEVEN--DAAQDARWLAFNIQRPIFADRKVREAVGLVFDFEWMNKALFYGAYSRANSFFQNT
EYAAR----SYPDADELTLLAPMK----KALPP------EVFT-TVYTPPQSKGDGFD----RDNILKALEL
LKEAGWELKN---------------------------------------------------------QKLIN
VKTG--KPFTFELLIGTTG-NT--QWVLPFQHNLERLGITMEIRQV-DNSQLTNRLR---NRDYDMMP----
-TLYSAMPYPS-ADVQISWASEYVD--STYNAPGVKSPVIDKLIDQIIANQGDKKKLLPLGRALD-RVLTWN
FYMIPMWYMT---------ADRFAWWN-KFSKPAIHPLYT-----SGFDTWWYDVNKAARLPA---------
----------------
>Buttiauxella/1-599 noackiae
------------MFARILTLLSLCLIAAPGLAENIKESYSFAVLGE--P-KYAINFTHLDYVNPAAPKGGDI
TLSAIG--------TFDNFNRYAL-------RGNPGVR-TDT-----------LYDTLFANSDDE-------
--PGSYYPLVAEMARYPDNF----AWAEIEINPQARFHDGTPIKASDVAFTFNKFMTEG-VPQFRL-VYKGA
-KVRAIAPLTVRIELATP--SKERMLGLFS-LPVFPEKFWK-------NHKLSDPLSA-PPLASGPYKITAW
RMGQYITYSRVRDYWAANLPVNRGRWNFDSIRYDYYLDDNVAFEAFK-AGAFDFRNEA--SAKNWATRYT-G
KNFANHFIVKEEQPN--DAAQDARWLAFNIQRPIFEDRKVREAIGLAFDFEWMNKALFYGAYSRANSYFQNT
EYAAR----DYPHADELILLAPLK----KDLPP------EVFT-SVYNPPKSNGNGFD----RENLLKAMTL
LKEAGWELKD---------------------------------------------------------QKLIN
VKTG--KPFTFELLIGASG-NT--QWVLPFQHNLQRLGITMEIRQV-DNSQLTNRMR---KRDYDMIP----
-TLYSAMPYPS-ADVQIAWASEYVD--STYNAPGVKSPVVDELISQIVANQGDKQKLLPLGRALD-RVLTWN
YYMIPMWYMA---------ADRFAWWN-KFSKPAIHPLYA-----TGFDTWWYDVNKAAHLPA---------
----------------
>Siccibacter/1-601 colletis
-------------MIARIAIVLMSLVSLPGLAATIEEAEAFAVIGE--P-KYAVDFTHYDYVNPAAPKGGSI
TLAKIG--------TFDNFNRFAL-------RGNPGER-TGA-----------LYDSLYTTSDDE-------
--PGSYYPLIAESARYSSDY----AWAEIAINPRARFHDGTPVTARDVAFTFQKFMTEG-VPQFRI-FYKGT
-TVKAIAPLTVRIALGEA--GKEKLLSLFT-LPVMPEKYWQ-------SRKLSDPLPT-PPLASGPYKISAW
RMGQYITYSRVKDYWAATLPVNRGRWNFDTVRYDYYLDDNVAFEAFK-AGAFDFRTES--SAKNWATRYI-G
RNFAQGYITKEEQPN--TSAQDTRWLAFNIQRPLFSDRRVREAITLAFDFEWMNKALFYGAYSRANSYFQNT
EYAAR----GYPDAAELTLLAPMK----ADLPP------EVFT-AIYQPPVSKGDGFN----RDNLLKAAAL
LKSAGWEIKN---------------------------------------------------------QKLVN
VKTG--QLFRFELLLSSGG-NN--QWVIPFQHNLERLGITLDLRPV-DNSQITNRLR---SRDYDMIP----
-TLYRAMPFPG-SDLQFSWASQYID--STYNSPGVSNPVIDQLIEKVIARQDDKAALLRLGRALD-RVLTWN
YYMIPMWYMA---------SDRFAYWD-KFSRPAVRPVYD-----LGLDNWWYDINKAAKLPEARR------
----------------
>Cronobacter/1-601 muytjensii
-------------MIARAFVLLFLMFSAVCQAQTIHESYSLAVLGE--P-KYAVNFNHFDYVNPAAPKGGNI
TQAALG--------TFDNFNRYAL-------RGNPGVR-TET-----------LYDTLFTSSDDE-------
--PGSYYPLIAEMIRYPDDF----SWAEVTINPQARHHDGSPITAKDVAFTFHKFMTEG-VPQFRL-YYKGA
-TMKAIAPLTVRIELAQP--GKENMLGLLT-LPVMPEKYWR-------DHKLSDPLST-PPLANGPYRVSQW
RMGQYIVYSRVKDYWAANLPVNRGRWNFDTIRYDYYLDDNVAFEAFK-AGAYDFRPET--SPKNWATRYN-G
SNFERGYIIKDEFEN--TTAQDARWLAFNIQRPFFADRRVREAISLAFDFEWMNKVLFYKAWRRPNSYFLNT
DYAAR----HYPDADELTLLAPLK----KAVPP------EVFT-KIYEPPVSRGDGFD----RDNLIKALAL
LKEAGWELKN---------------------------------------------------------NKLVN
RATG--KPFAFELLLGSAG-NN--QWVLPFQHSLARLGMTMTIRQV-DNSQLTNRLR---KRDFDMMP----
```

-TLYRAAPWPD-TGLQVSWASDYID--SSWNTPGVQNPAVDKLIEQIIENQGNQQKLLPLGRALD-RVLTWN
YYMIPMWYAA---------ADRVAVWN-KFSRPATRPLYT-----LGFDTWWYDVNKAAALPEARR------
----
>Pluralibacter/1-597 gergoviae
---------------VLIALLCLALGSAGVRAQTIKESSAFAAIGE--P-KYAPGFDHFDYVNPDAPKGGRV
TLAAIG--------TFDNFNRYAM-------RGNAAVR-TDQ-----------LYDSLFTTSDDE-------
--PGSYYPLIAEGVRYASDF----SWAEVTINPRARFHDGSPVTAEDVAWSFHKFMTEG-VPQFRL-IYKGT
-TMKAVAPLMVRIDLAKP--GKEDMLSLFS-LPVMPKKFWL-------HRRFDEPLSA-PPLAGGPYKISAW
RMGQYVVYSRVKDYWAANLPVNRGRWNFDTIRYDYYLDDNVAFEAFK-AGAVDFRSET--STKNWATRYL-G
RNFTSGYIVKEEEKN--SSAQDTQWLAFNIQRPVFADRAVREAVTLAFDFEWMNKALFYGAYSRANSYFQNS
EYAAR----GLPDADELALLTPMK----KALPP------EVFT-AAWRPPASDGGGFD----RENLLKADRL
LTAAGWVLKG---QQRVN
ARTG--LPLRFELLLPSSA-NS--QWVIPFQHNLQRIGVRMDIRQV-DNSQATSRLR---SRDYDMMP----
-RPWRAVLWPSGGDLQIAWGSQYIN--SSYNGPGVQSPVIDSLLAQMIRNEGNKPKLLPLARALD-RVLTWN
YYQLPMWYMA---------TDRVAYWD-KFSRPAVRPVYT-----LGFDNWWYDVNKAAKLPA---------
----
>Shimwellia/1-583 pseudoproteus
-------------------------------AAQINEGYSFAVLGE--P-KYATDFSHFDYVNPAAPKGGDI
TLSAIG--------TFDNFNRYAL-------RGNPAVR-TDS-----------LYDSLFTTSDDE-------
--PGSYYPLVAEVARYADNF----AWMEIQINPRARFHDGSPVTAADVAFTFTKFMTEG-VPQFRL-VYKGA
-TVKAISRLTVRIQLATP--ARERILGLLT-LPILPEKFWK-------NHKLSDPLSR-PPLAGGPYRITAW
RMGQYVTYSRVKDYWAANLPVNRGRWNFDTLRYDYYLDDNVAFEAFK-AGAFDFRSES--SAKNWATRYT-G
KNFTSGYIIKEDQQN--SSAQDTNWLAFNIQRPVFADRRVREAIGLAFDFEWMNKALFYGGYKRVNSYFQNT
EYAAR----DYPDAAELVLLAPWK----SRLPP------DVFS-QIWQPPVSKGDGFD----RHNLMKALDL
LKQAGWELKD---QQLVN
SKTG--QPLNFELLLSSAG-NN--QWVLPFQHNLQRLGITMTVRQV-DKSQLINRRR---SRDYDMTP----
-VAYGAMPWPS-SDLQILWASAYVD--STWNAPGVKDPVVDSLVAQIISHQGDKAKLLPLGRALD-RVLTWN
QYMLPMWYVA---------ADHLAYWD-KFSQPATRPIYS-----LGFDNWWYDINKAQHLPAGRR------
----
>Entomohabitans/1-596 teleogrylli
------------------MALLLCAVCLPGRASEVKESYAFAVLGE--P-KYAVNFSHFDYVNPAAPKGGDI
TLSSIG--------TFDNFNRYAL-------RGNPGVR-TDS-----------LYDSLFTTSDDE-------
--PGSYYPLIAELARYPADY----AWAEVEINPRARFHDGTAIVASDVAYTFKKFMTEG-VPQFRL-IYKGA
-TVKAIAPLTVRIELATP--NKDRLLGLLS-LPILPEKFWQ-------NHKLSDPLSQ-PPLSSGPYRISAW
RMGQYITYSRVKDYWAADLPVNRGRWNFDSIRYDYYLDDNVAFEAFK-AGAFDFRSES--SAKNWATRYI-G
KNFTDGYIVKEEQKN--TSAQDTRWLAFNIQRPIFADPRVRQAISLVFDFEWMNKALFYNAYSRVDSYFQNT
EYAAR----GYPDADELILLAPWK----KELPG------EVFS-AVYQPPVSRGDGFD----RDNLLRALEL
MKSAGWELKD---QKLVN
VKSG--EAFRFELLLSSAG-NN--QWVLPFQHNLARLGVTMDIRQV-DNSQLTNRLR---RRDYDMMP----
-TVYRAMPYPS-PSLQISWASEYVD--STWNAPGVKSEIIDSLIDQIIANQGDQRKLLPLGRALD-RVLTWN
YYMLPMWFMS---------ADHFAYWN-KFSRPAITPVYS-----LGFDNWWYDINKAGQLPAQRR------
----
>Jejubacter/1-601 calystegiae
-------------MFVRLFVLLLSLISSSVIAQEIDESYAFSVLGE--P-KYDVDFNHFDYVNPAAPKGGSI
TLSAIG--------TFDNFNRYAL-------RGNAGIR-TGE-----------LYDALFATSDDE-------
--PGSYYPLIAEKARHPEDF----AWTEISINPRARFHDGSPITAKDVEYTFHKFMTEG-VPQFRI-YYKSV
-KVKAISRLTVRIELPKP--SKDTMLSLFT-LPVMPEKFWR-------HHNLGEPLSR-PPLSSGPYRLSAW
RMGQYAIFSRVKDYWAADLPVNRGRWNFDTIRYDYYLDDNVAFEAFK-AGAFDFREEP--SPKNWATRYV-G
KNFTNHHIVREEQDN--NSAQNARWLAFNVKRPIFADRRVREAITLAFDFEWMNKALFYGAYSRTNSYFQNT
EYAAR----GYPQADELTLLAPFK----KSLPP------EVYS-QIYQPPVSRGNGFD----RDNLLKALAL
LKAAGWELKG---GKLVN
AKTG--KPFAFELLLASGA-NN--QWALPFSHSLERLGIQVNVRQV-DNSQLTNRLR---SRDFDMMP----
-TVYRAMTYPS-SDLQIIWDSAFID--STWNTPGVQDPVVDKLISQIIAHQGNEQKLLPLGRALD-RVLTWN
FYMLPMWYMA---------SDRLAYWD-KFSHPQIRPRYS-----LGFDSWWYDINKAARLPAERR------
----
>Mangrovibacter/1-583 yixingensis
-------------------------------APKIEESYSFAVLGE--P-KYAVDFTHFDYVNPAAPKGGSM
TLATIG--------TYDNFNRYAL-------RGNPGIR-TES-----------LYDSLFTTSDDE-------

```
--PGSYYPLIAEKARYPDNF----AWAEISINPEARFQDGSPVRASDVAFTFHKFMTEG-VPQFRL-YYKGV
-KVKAISPLTVRIELASP--SKDMLLGLLT-LPVMPESFWK-------DHKLSDPLST-PPLSGGPYKITAW
KMGQYIVYSRVKNYWAANLPVNRGRFNFDTLRYDYYLDDNVAFEAFK-AGAYDFRSES--SPKNWATRYT-G
TNFDKHYIRKESWPN--QGAVDTQWLAFNIQRPMFTDRRVREAIGLAFDFNWFNKALFYGAYRRPDSYFENT
EYAAR----NYPDANELTLLAPMK----AQLPP------DLFT-HIWHPAQSKGDGFD----RANLLKAMNL
LKEAGWEIRN---------------------------------------------------------QQLVN
TKTG--KPFQFELLLGNGG-ND--QWVLPFQHNLRRLGITMSIRQV-DNSQLTNRLR---KRDFDMLP----
-SRYQAMPWPS-SDLQILWASQYID--STWNRPGVANPVVDKLTADIVAHQGDKAALITLGRALD-RVLTWN
YYMIPMWYMP---------TRQLAWWD-KFSHPEIQPVYS-----VGLDSWWYDVNKAAALPAQRR------
----------------
>Yersinia/1-583 enterocolitica
----------------------------GLQAETIKEGSSFAILGE--P-KYSSDFSHFDYVNPAAPKGGDI
TLSAIG--------TFDNFNRYAL-------RGNPAVR-TER-----------LYDSLFSTSDDE-------
--IGSYYPLIAESARFAPDF----HWIEVDINPRARFHDGKPITAQDVAFTFNKFMTEG-VPQFRI-VYKGV
-QVKAISRLTVRFEFPEP--NKDKMLGLFG-LPVMPEHFWK-------DHKLSDPLSQ-PPVSSGPYRIGKY
KMGQFITYERVKDYWAANLPVNRGQYNFDTIRYDYYLDDKVALEAFK-AGAFDFREET--SPKSWATQYV-G
GNFAKNYIVKQDILD--NSAQNTRWLAFNVQRPIFSDRRVREALTLAFDFDWMNKAFYFDSYQRTNSFFQNT
EYAAK----GYPDSAELAWLAPLK----GKIPP------EVFT-QIYQPPHTDGSGNA----RDNLLKAREL
LSEAGWEIKN---------------------------------------------------------QQLVN
SKTG--KPFVFELLLLSGS---NFQYVQPFKHNLQRLGITMNIREV-DSSQFVNRLR---SRDYDMIP----
-TVYTAFPYPS-PNLQILWSSAYID--SSYNASGIKDPAIDQLIEQIIKHQEQPEVLLSLGRALD-RVLTWN
YLMIPMWYSN---------HARFAYWD-KFSMPADRPTYS-----LGFDSWWFDVNKAARLPT---------
----------------
>Rouxiella/1-594 silvae
--------------------LLLSLIGFCAHAESIEDGYAFSILGS--P-KYLYNFSHFDYVNPAAPKGGSI
TLSAIG--------TFDNFNRFAS-------RGNAAVR-SDT-----------LYDSLFTPSSDE-------
--IGSYYPLIADSARYDSDL----KWVEVEINPRAVFQDGSPITAKDVAFTFHKFMTEG-VPQFRV-VYKGV
-VVKALSPLTVRFEFPQP--NKEQMLGLLG-LPIISQKFWE-------HRKFNEPLST-PPLGSGPYRISAY
KLGQYVTYSRVTHYWAANLPVNRGRYNFDTIRYDYYLDDNVALEAFK-SGAFDYRSEP--SPKNWATQYQ-G
TNFTRGLIIKQDDKN--QAAQDTRWLAFNIHRPIFQDRRVREALTLAFDFDWMNKALFYGAYKRTTSYFQNT
EYAAV----NYPDAAELAWLAPLK----GKIPD------EVFS-QLYQPPHSDGSGSD----RENLLKATAL
LKEAGWTVKN---------------------------------------------------------QQLVN
DQTG--KPFTFELLLPSSG---NSQYVLPFQHNLQRLGINMTLRQV-DNSQFTSRLR---SRDYDMIP----
-TVYRAMTYPG-TDLQIMWNSKYIN--STYNAPGVSDPAIDQLTDAVVAHQGQPDALLSLGHALD-RVLTWH
MYMIPMWYSN---------RDRYAYWD-KFSMPAIRPPYA-----IELDSWWFDVNKAARLQAAKK------
----------------
>Edaphovirga/1-599 cremea
------------MLPRVFTVLLLSMMVFFVRAETINESYAFAILGE--P-KYDASFSHYDYVNPAAPKGGSI
RLAALG--------TYDNFNRWAS-------RGNPAAR-SGQ-----------IYDALFSPSSDE-------
--LGSYYPLIAESARYASDL----SWVEVTINPHARFHDGSPITAADVEFTFNKLMTEG-VPQFRL-IYKGT
-NVKAIARHTVRFEFSKP--DKDQVLGMLG-LPILPMSFWK-------DHRLDQPLMK-PPLGSGPYKISAY
RFGQYVTYSRVKDYWAANLPVNRGLNNFDTIRYDYYLDDKVALQAFA-AGAYDFRMEG--SPKNWATQYQ-G
GNFSKKYIVKEDEEN--KAAQDTRWLAFNVQRPIFQDRRVRQALTLAFDFEWMNKALFYGAYQRTNSYFQNT
DYAAR----GYPDSAELAWLAPLK----GKIPD------EVFS-EIYQPPRSDGSGND----RANLLKATAL
LKEAGWEVQN---------------------------------------------------------QQLVN
SKTG--KPFTFELLLPSGG---NNQFVLPFQHNLQRLGITMEVREV-DNSQFLSRLR---SRDYDMLP----
-KVYQAQSYPG-ANLMIFWGSQYLD--SSYNAPGIQDPAIDALIEQIIAHQGQAEPLLSLGRALD-RVLTWN
QLMIPMWYSN---------HDRYAYWD-KFSMPVNRPAYS-----MELDSWWYDVNKADRLPA---------
----------------
>Sodalis/1-602 ligni
------------MFLRFWIALVFSIVSYGPHAEAINEGYAFALLGE--P-KYSSDFNHFDYVNPAAPKGGAV
TLAAIG--------TFDNFNRYAL-------RGSAAMR-TDQ-----------LYDSLFTTSDDE-------
--MGSYYPLVAESARYPADF----KWIEITINPRARYHNHQSITARDVAFTFNKFMTEG-VPQFRL-YYKGV
-TATAIAPLTVRYEFPQP--DKDKMLGLLD-LPILPESFWQ-------HHKLSDPLSS-PPPASGPYRITAW
RTGQYVTYSRVKDYWGETLPVNRGRNNFDTIRYDYYLDDNVALEAFK-AGAYDLRVET--SPKNWATQYH-G
GNFDRGYIVKQEVPN--NAPVTTPWLAFNIQRPLFADRRVREAIGLAFDFEWMNKALFFNGYERVNSYFINT
DYAAR----GYPDAAELAWLAPLK----GQIPP------EVFT-DIYHPPVSDGSGYD----RKNLLKALTL
LKEAGWELKN---------------------------------------------------------QQLVN
```

```
SKTG--KPFQFEMMLMSG---NNSQYILPFQHNLQRLGITLQIREV-DSAQFTNRLR---KRDFDMMP----
-ATYAPFPYPS-GDLQIMWGSAYVE--STYNRPGVKDPAIDRLINEIVHHQGDEKALLSLGRALD-RVLTWN
QFMIPMWFSH---------QTRLAYWD-KFAMPEIRPTYA-----LGFDTWWYDVNRSAKLPAERR------
----------------
>Chania/1-599 multitudinisentens
------------MSVRIFAALVLSALSFGLQAETLNESYAFATLGE--P-KYSTDFSHFDYVNPAAPKGGNV
KLAAIG--------TYDNFNRFAS-------RGVSGER-TAE-----------LYDTLFTTSDDE-------
--PSSYYPLIAESARFPADM----RWMELDINPLARFHDGSPITAADVAFTFNKFMTEG-VPQYRS-YYKGT
-TVKALSRLTVRIELPKP--DKEKLLGLLS-MRVLPESFWK-------NHKLSEPLNT-PPLGSGPYKVGSY
RLGQYITYERVRDYWAANLPVNRGRYNFDTLRYDYYLDDKVALEAFK-AGAYDFRQES--SPKSWATQYQ-G
GNFARNFIIKQDEVN--QAAQNARWLAFNIQRPIFADRRVREAITLAFDFDWMNKALYYGAYQRTNSYFQNT
IYTAN----GYPDAAELSWLAPLK----GKVPE------EVFT-SIYQPPVSDGSGND----RENLLKATAL
LQQAGWEVKD---------------------------------------------------------QQLIN
SQTG--QPFTFELMLLSGS---NFQYVLPFQHNLKRLGIDMQIREV-DASQFTRRLR---QRDFDMVP----
-TVYPATPFPS-ADLQILWGSQYLD--STWNTPGVSDPAVDALLEKIIKHQGDEEALLSLGRALD-RVLTWN
MYMLPMWYSN---------HDRYAYWD-KLSSPTVRPAYS-----LGFDNWWFDVNKAARLPA---------
----------------
>Erwinia/1-599 typographi
------------MILRWFTFLLFSVTAASAQAENIKVSYAFAQLGE--P-KYAADFTHYDYANPTAPKGGSV
TLSAIG--------TYDNFNRFAM-------RGNPGAG-TDT-----------LYDPLFTTSDDE-------
--TGSYYPLIAESARYPDSS----RWIEVTLNPQARFQDGSAITATDVAFTFHKFMTEG-VPQYRV-YYKGV
-TAKVISELTVRFELPES--DRDKMLSLLS-TPVFPEKFWK-------DHNLGEPLSA-PPLSSGPYRVSSY
KVGQYISYSRVKNYWAANLPVNKGRFNFDTLRYDYYLDDNVAFEAFK-AGAFDFRNES--SAKKWATQYR-G
KNFDNHFITKDEQPN--DVATASSWLAFNVQKPLFSDRRVREAISLAFDFEWMNKALFYHAYHRSNSYFQNT
EYAAR----NYPDAKELEILGPLK----GKIPD------RVFS-QIYQPPHTDGSGYD----RANLLKALDL
LKQAGWTLKN---------------------------------------------------------QKLTN
VKTG--QSFGFELLLRSGG-ND--QWVLPFQHTLSRLGITLTIRQV-DSSQYLARLR---KSDFDMLP----
-SPYPAMPFPD-SNLQIYWASSFVN--SSYNRPHVMSPVLDGLIAQINQHQGDKVALLPLGRALD-RVLTWN
YYMIPMWYNA---------NNRYAYWS-KFSMPTIRPTYS-----LGFDSWWYDANKAAQLPA---------
----------------
>Serratia/1-599 marcescens
------------MSVRIFAALVLSALSFGLRAEALNESYAFALLGE--P-KYATDFSHFDYVNPAAPKGGDV
RLAAIG--------TYDNFNRFAT-------RGVPGER-TME-----------LYDTLFTNSDDE-------
--PGSYYPLIAESARFPADM----RWMELDINARARFQDGSPITAADVAFTFNKFMTEG-VPQFRS-YYKGV
-TVKAISRLTVRIELPKA--NREQILSLLS-LRVLPESFWK-------NHKLNEPLST-PPLASGPYKIGDY
RLGQYITYQRVRDYWAANLPVNRGRYNFDSIRYDYYLDDKVSLEAFK-AGAYDFRIEP--SPKSWATQYQ-G
GNFARNYIIKQDETN--QAAQNTRWLAFNLQKPLFADLRVREAIGLAFDFNWMNKALYYNAYQRADSYFQNT
AYAAR----GYPDAAELALLAPLK----GQIPP------EVFT-SIYQPPSSDGSGND----RQNLLKATQL
LKEAGWVVKN---------------------------------------------------------QKLVN
AKTG--QPFTFELMLLSGS---NFQYVLPFRHNLQRLGIEMQIREI-DASQYTRRMR---ERDFDMMS----
-TVYMAMPFPS-ADLQILWDSQYID--SSYNTPGVKDPAVDSLVRQIAAHQGDENALLPLGRALD-RVLTWN
NYMLPMWYSS---------HDRYAYWD-KFSTPSVRPAYA-----IGFDNWWYDVNKAARLPA---------
----------------
>Obesumbacterium/1-589 proteus
----------------------VAAFSFNLRADVINESYAFAILGE--P-KYNSDFTHFDYVNPAAPKGGDI
TLSALG--------TYDNFNSYAS-------RGHPAVR-ANQ-----------LYDTLFTNSADE-------
--IGSYYPLIGETVRYDDKF----SWAEVNINADARFQDNTPITAQDVQFSFNKFMTEG-VPQYRI-YYQGV
-KVKAISRLTVRFELPKP--DKEMMLSLIGGLKVMPESFWK-------NHKLSEPLKT-PPLGSGPYRISDY
RLGQYVTYSRVTNYWAANLPVNRGQYNFDTIRYDYYLDDKVALEAFK-AGAFDFRMET--SPKNWATQYS-G
GNFAKNYIIKSDETN--QSAQDTRWLAFNTQRPIFADRRVREAISLAFDFEWMNKALYYDAYQRTNSYFQNT
QYAAR----GYPDSAELAWLAPLK----GKIPA------EVFN-QVYQPAATDGSGND----RENLLKATKL
LEEAGWTIKD---------------------------------------------------------QRLVN
KATG--KPFAFELLLPSSS---NFQYVMPFKHNLEKLGITMNLREV-DTSQYSNRVR---SRDFDMFP----
-TVYGGMNYPD-PSLKILWRSDYIS--STWNSAGVQDPAVDSLIDNIISHQGQPNALLSLGRALD-RVLTWN
YYMLPMWYSN---------HDRIAYWD-KFSMPAQRPAYD-----LGFDTWWYDINRAANLP----------
----------------
>Gibbsiella/1-599 quercinecans
------------MLMRVFAALAFAACSFALHAETINESYAFALLGE--P-KYSNDFTHYDYVNPAAPKGGDV
```

RLAAIG--------TYDNFNRFAS-------RGVPGQS-TGD-----------LYDTLFASSDDE-------
--PASYYPLIAESARYPADM----RWMELSINPRARFQDGSPITAADVAFTFNKFMTEG-VPQYRV-YYKGV
-KVKAISRLVVRIELPEA--DREKMLSLLS-TRVLPESFWK-------DHNLSEPLGM-PPLGSGPYKVSSY
RAGQYITYQRVRDYWAANLPVNRGRFNFDTFRYDYYLDDKVALEAFK-AGAYDFRTES--APKSWATQYQ-G
GNFARNYIVKQDETN--QAAQNTRWLAFNIHRPIFADRRVREAITLAFDFDWMNKALYYNAYQRTRSYFQNT
PYAAS----GYPDAAELAVLAPLK----DRVPP------EVFN-QIYQPPSTDGSGYD----RQNLLKATQL
LQEAGWEVKN---QRLIN
SKTG--KPFVFELMLLSGS---NFQYVLPFKHNLQRLGITMEIREI-DVTQYTRRMR---ERDFDMMP----
-TVYPAMPFPN-ANLEMYWGTHYIN--SSYNSPGVSDPAVDALLAQILKSQGDEAALLPLGRALD-RVLTWN
MFMLPMWYSN---------HDRFAYWD-KFSMPSIRPAYA-----VGFENWWYDVNKAARLPA---------
----
>Hafnia/1-590 paralvei
---------------------LVAALSFNVRADVINESYAFSILGE--P-KYSSDFTHFDYVNPAAPKGGDI
TLSALG--------TYDNFNSYSS-------RGHAAVR-ANQ-----------LYDTLFTNSADE-------
--IGSYYPLIGESVRYDDQF----RWAEVNINADARFQDNTPITAQDVAFSFNKFMTEG-VPQYRI-YYKGV
-NVKAISRLTVRFELPTP--DKEMMLSLIGGLKVMPESFWK-------NHKLSEPLST-PPLGSGPYRISDY
RLGQFVTYSRVTNYWAANLPVNRGQYNFDTIRYDYYLDDKVALEAFK-AGAFDFRIET--SPKNWATQYS-G
GNFAKNYIVKSDETN--QSAQDTRWLAFNTKRPIFADRRVREAISLAFDFAWMNKALYYDAYQRTNSYFQNT
QYAAR----SYPDSAELAWLAPLK----GKVPA------EVFT-QIYQPASTDGSGND----RENLLKATTL
LEDAGWTVKD---QKLVN
KTTG--KPFVFELLLPSSS---NFQYVMPFKHNLEKLGITLNIREV-DTSQYTNRIR---SRDFDMFP----
-TVYGGMNYPD-PSLKILWRSDYID--STWNSAGVQDPAVDSLIDNIISHQGQPEALLSLGRALD-RVLTWN
HYMLPMWYSN---------HDRIAYWD-KFSMPALRPAYD-----LGFDTWWYDVNRAAQLP----------
----
>Pantoea/1-595 rwandensis
----------------LVTALLVFIAIAPLHAETINESYAFAKLGE--P-KYAVNFSHYDYVNPAAPKGGKM
TLAVVG--------TYDNFNRYAS-------RGNPGAG-TDT-----------LYDGLFTTSDDE-------
--PGSFYPLIAESARYPNDY----HWMEVSINPHARFQDGSPITAQDVAFTFQKFMTEG-VPQFRV-AFRGA
-QVKALNNLTVRIDMPKP--DKDMILSWLT-LPVLPQQFWH-------DKKFNEPIGY-PPLASGPYRVTSY
KSGQFIQYSRVKNYWAADLPVNRGRFNFDTLRYDYYLDDNVAFEAFK-AGASDLREES--SAKKWATQYL-G
RNFDNHAIVKSTTPN--TVSTDTRWLAFNNEKPLFADRRVREALTLAFDFEWMNKALFYGAYKRTDSYFQNT
EYAAK----GYPKAEELILLAPYK----AELPA------EVFT-SEYAPPHSDGSGFD----RGNLLKAMNL
LKQAGWEIKN---QRLVN
VKTG--QPFRFELLMRSGT-QN--DWALPFQHSLQRLGIQVDLRQI-DTSQYLRRWR---EGDYDMIP----
-TLYQANPWPS-TNLQTSWQSQYID--SSWNTPRVKNPIVDHFVQQIVDHQGDKKALLPLGKALD-RILLWN
AYMIPMWYNA---------EQRLAYWD-KFSHPEIKPAYA-----TGLDNWWYDVNKAAKLPA---------
----
>Mixta/1-601 gaviniae
------------MLCRLLILILVFGSALSAQADIIRESYAFAELGE--P-KYGVGFTHFDYVNPAAPKGGKI
TLPALG--------NYDNFNRYAS-------RGYPGAR-TET-----------LYDPLFTASEDE-------
--IGSYYPLIAEYARYPADF----KWAEITLNPRARFQDGTPITAQDVAFTFEKFMTEG-VPFFRV-IYKGT
-TVKAISRLTVRIELPKP--DRSMMLDLFT-LPVLPEKFWR-------QHKFNEPLGY-PPLSSGPYRITSY
KVGQYIVYSRVKDYWAADLPVNKGRFNFDTIRYDYYLDDNVAFEAFK-AGAYDFRTEI--SAKNWATQYR-G
ESFDRQQIVKEPRPN--QVTTDAAWLAINNEKALFSDRRVRQALTLAFDFEWMNKALFYQSYQRTSSYFQNT
DYAAR----GYPDAAELEILAPFK----GKIPD------EVFS-ERFQPPISDGSGYD----RANLLRALKL
LQEAGWQLKN---RQLVN
-AAG--QPFRFELLVNSGS-S--ISWVLPFQHSLKRLGITMTIRQI-DSSQYLRRLR---QGDYDMTP----
-TNYYAVPWPN-SGLSQRWQSAFIE--SSWNQARVKNPVLDALIDRILAHQGDKNALLPLGRALD-RVLLWN
NYMIPMWYSG---------QDRYAWWN-KFSHPAVIPAYS-----VGLDNWWYDVNKAAKLPAQRR------
----
>Enterobacillus/1-603 tribolii
------------MYARVLIALFFSLLSLNVRADTINESYALSILGE--P-KYTSDFTHFDYVNPAAPKGGDI
TLAAIG--------TYDSFNRYSM-------RGNPAAR-SGE-----------MYDTLFASSADE-------
--IGSYYPLIGESVRYDSQY----RWAEVNLNANARFQDNTPITAKDVEFSFNKFTTEG-VPQFRS-FYRGV
-KVKAISRLTVRFELPEP--DKEMMLSLIGGLKIMPASYWQ-------HHKLSDPLST-PPLGSGPYRVSDY
RLGQYVTYARVRDYWAANLPVNRGRFNFDTIRYDYYQDDKVSLEAFK-AGAYDFRIET--SPKNWATQYK-G
GNFARKYIVTQDDIN--QAAQDTRWLAFNIKRPIFADRRVREAITLAFDFNWMNKALYYGAYQRTDSYFQNT
PYAAR----SYPDAAELGWLAPLK----DKIPA------ETLS-QIYQPPQTDGSGND----RENLLKAARL

LEEAGWEVKD---QRLVN
KQTG--KPFVFELLLLSGS---NFQYVQPFQHNLARLGITMEIRQI-DTSQYLNRMR---KRDYDMMP----
-TVYQGMNYPE-TSLKILWDSHYLD--SSWNAPGVQDPAVDTLVAEIIANQGKPDALRALGRALD-RVLTWN
YYMLPMWYSN---------HDRLAYWD-KFSMPALRPTYS-----LGFDTWWYDVNRASRLPEQRR------
----
>Affinibrenneria/1-602 salicis
------------MFQRIAIAVLLCTLHFGLQAEDRQQGFSFALLGE--P-KYAQGFQHFDYVNPDAPRGGAI
TLSAIG--------TYDNFNRYSM-------RGSPAAR-TER-----------MYDSLFTASEDE-------
--PASYYPLVAESARYPADY----RWIEITLNPDASFHDGTPILASDVAFTFTKFMTEG-VPQFRL-YYKGT
-TVKAIAPRTVRIELPKP--DKDQLLSLFT-LPIMPEKFWR-------QHKLNEPLAF-PPPGSGPYRITAY
RMGQYVTWSRVKDYWGADLPVNRGRFNFDQIRYDYYLDDNVALEAFK-AGAYDFRMES--SPKHWATQYQ-G
GNFARGFIVRQDWAN--QSAQDTRWLAFNIKRPLFADRRVRQALSLLFDFNWMNKALYYQSWQRTDSYFQNT
DYAAR----GEPDAAERAWLTPLA----DQIPP------EVFG-PALRPAVSDGSGYN----RDHLLQALEM
LRQAGWELKN---QQLVN
RQTG--KPFTFELLLLSG---SNSQYVLPFQHNLRRLGINMTLREV-DSSQFINRVR---SRDFDMIP----
-TVYSAFEYPN-PDLQIFWSSQYID--SSYNRSGVQDPAIDRLIELIVQHQGQPEALLSLGRALD-RVLTWN
QFMIPMWYSN---------HDRFAYWD-KFSMPASRPSRS-----LGVDGWWYDAGKAARLPAARR------
----
>Ewingella/1-602 americana
------------MFSRLITAALFSALSLCAQAEVIQDSYAFSVLGE--P-KYFSNFSNFDYVNPAAPKGGTI
RLAAIG--------TYDNFNRYAS-------RGNPAVR-SDT-----------LYDSLFTTSSDE-------
--IGSYYPLVADSARYDSNF----RWVEVDINPRARFHDGSAITANDVAFTFSKFMTEG-VPQFRV-VYKGA
-EVKAISRLTVRIVLPRP--DKDMMLGLLG-LPILPQKFWE-------KHKFNEPLST-PPLSSGPYRISDY
KLGQYVTYSRVPDYWAANLPVNRGRYNFDTIRYDYYLDDSIALEAFK-SGAFDFRMEA--SPKNWATQYK-G
TNFSKEYIVKQDDQN--QAAQDTRWLAFNIQRPLFSDRRVREALTLAFDFEWMNKALYFGSWQRANSYFQNT
EYSAS----NYPNSAELAWLAPLK----GQVPD------EVFS-KLYQPPVSDGSGND----RANLLKATEL
LKQAGWEVRN---QQLVN
AKTG--KPFTFELLLPGGA---NTQYALPFQHNLARLGIKMTLREV-DNSQFVRRFR---QRDFDMVP----
-SVYSAMPYPS-PDLQIIWNSKYID--SSYNRPGVKDPAVDKLTDEIAANQGKPDALLALGHALD-RVLTWN
MYMIPMWYNN---------HQRYAYWD-KFSMPSIQPMNG-----LELDTWWYDINRANRLPAERR------
----
>Acerihabitans/1-603 arboris
------------MFARVWIALVFSVVSLGPRAETINEGYAFALLGE--P-KYPSDFSHFDYVNPAAPKGGGV
TLAAIG--------TFDNFNRFAL-------RGTAAMR-TDQ-----------LYDGLFITSDDE-------
--LGSYYPLVAESARYAADF----SWMEIAINPQAHYHNHQPITAKDVAFTFGKFMTEG-VPQFRI-YYKGV
-TVKAIAPLTVRYEFPKP--DKDMALGLLT-LPVMPESYWQ-------HHKLGDPLGS-PPPASGPYRITSW
RTGQYVTYTRVRDYWAANLPVNRGRNNFDSIRYDYYLDDNVALEAFK-AGAFDLRIES--SPKLWATQYH-G
GNIKRGYIIKQEIPN--DAPVNTPWLAFNIQRPLFADRRVREALTLAFDFEWMNKALFYQGYQRVDSYFINT
DYAAR----DYPGVDELAALAPLK----GLVPP------EVFT-ERYRPPVSDGSGYD----RKNLLKALAL
LKQAGWELKD---QQLVN
GKTG--KPFTFEMMLMSGG--NSSNYILPFQHNLQRLGIRMTIREV-DNAQFTNRLR---KRDFDMMP----
-SSYAAFPYPS-GDLQILWGSAYVE--STYNRPGVKDPAVDRLINEIVRHQGDEKALLPLGRALD-RVLTWN
YFMIPLWFSH---------QTRLAYWD-KFAMPAVKPTYA-----LGFDAWWYDVNRAARLPAERR------
----
>Rahnella/1-601 victoriana
------------MFSRAF-AVVLCLLSFSTQAEVIQDSYAFAILGE--P-KYVSNFTSFDYVNPAAPKGGQI
TLAAIG--------TYDNFNRFAT-------RGNPAAR-SGS-----------LYDSLFTTSSDE-------
--IGSYYPLIADSARYDSQY----RWVEVDINPRARFNDNTPITASDVAFSFKKFMTEG-VPQFRV-VYKGV
-EVKAISRLTVRMVLPKP--DKDMMLGLLG-LPILPQTFWE-------KHKFNEPLST-PPPGSGPYRISDY
KLGQYVTYSRVTDYWAANLPVNRGRYNFDTIRYDYYLDDNVALEAFK-SGAFDFRMES--SPKNWATQYQ-G
GNFSKNFIVKADEEN--QAAQDTRWLAFNIHRPQFADRKVREAITLAFDFDWMNKALYFGAYQRVNSYFQNT
EYAAK----DYPNSAELAWLGPLK----GKVPP------EVFT-TLYQPPASDGSGND----RTNLLKATAL
LKEAGWEIKN---QVLVN
SKTG--KPLTFELLLPSGG---NSQYVLPFQHNLQRLGIKMSIREV-DNSQFVRRMR---QRDYDMLP----
-TVYPATSYPS-SDLRIYWNTQYLD--STYNKSGVSDPAIDKLTDEIAANQGKPEALLSLGHALD-RVLTWN
MLMIPMWYTH---------HERYAYWD-KFSMPAVTPSNG-----MELDTWWYDMNRAARLPAQRR------
----
>Brenneria/1-602 goodwinii

```
------------MLKRVITAVLFSIMPLGLYAETIEHSTAFAILGE--P-KYHADFRHFDYVNPDAPKGGSI
TLSALG--------TFDNFNRFAL-------RGVAAAR-TER-----------LYDSLFVTSDDE-------
--PGSYYPLVAQSTRHDADF----HWIEIDLNPRASFHDGTPITAADVAFTYNMFMTQG-VPQFRI-YFKGV
-TAKTVAPLTVRFDFPTP--DKNRMFSLLT-LPIMPEKFWK-------NHKLSDPLPY-PPPASGPYRITAY
RTGQYVTYSRVKDYWGADLPVNKGQYNFDQIRYDYYLDDSVALEAFK-AGAFDLRVEG--SPKHWAAQYQ-G
GNFDRGYIIKQDQTN--QSAQDTRWLAFNIQRPIFSDRRVRQALTLAFDFNWMNKALYYNAYQRVDSYFQNT
IYAAK----GEPGADELAWLTPLK----DKVPP------EVFG-PSYQPPASDGSGYD----RGNWLKALKL
LEQAGWELKN---------------------------------------------------------QRLVN
RQTG--KPFEFELLLPTA---GNSQYALPFQQNLKKLGITMNIRNI-DSTQFNSRLR---KRDFDMTT----
-TLYRAMLYPN-DDLQIRWSSQYID--STYNTPGVQDPAIDSLIEQIVAHQGQPAPLLALGRALD-RVLTWN
QLMLPMWYSN---------HDRFAYWN-KFAMPAVRPAYS-----LGLDGWWYDVKQAATLPAERR------
----------------
>Winslowiella/1-589 arboricola
---------------------------LSAQAEKINESYAFSKLGE--P-KYATHFTHYDYVNPAAPKGGKM
TLTVVG--------TYDNFNRYAS-------RGYPGIA-TDG-----------LYDSLFTTSDDE-------
--IGSYYPLIAESARYPDDF----SWMEVTINPNARFQDGSPITASDVAFTFTKFMTEG-VPQFRV-AYKGV
-TVKTIAPLTVRIELPKP--DKDQILGLFS-LPVLSQKFWQ-------SRKFNEPLGY-PPLGSSAYRVSGY
KVGQYITYSRVKDYWAANLPVNRGRHNIDTLRYDYYLDDNVAFEAFK-AGAFDFRTEG--SAKKWATQYR-G
KNFENNQIVKDARPN--TVATDTQWLAFNTEKALFSDRKVREALSLAFDFQWMNKALFYNAYKRTDSYFQNT
DYAAS----GYPDARQLEILAPLK----GQIPD------EVFT-SRYQPPVSDGSGYD----RKNLLKALEL
LKQAGWELKN---------------------------------------------------------KQLVN
TTSG--KPFRFELLLNSSS-SSSIQWVLPFQHNLQRLGITLEIRQV-DSSQYLRRMR---EGDYDMIP----
-SQYYAQPSPD-PSLRIIWASQYIE--SSWNRPRVKSPQVDSLIEKILQHQGDKAALLPLGRALD-RLLLWN
YYMIPMWYNA---------DDRYAYWN-KFSMPAIAPTYA-----LGTDTWWYDVNKAAQLPAQRR------
----------------
>Pectobacterium/1-602 carotovorum
------------MLKRVIAAVLLCTAHFGAHAETTENSTSFALLGE--P-KYAENFSHFDYVNPDAPKGGSI
TLSSLG--------TFDNFNRYAL-------RGVAAAR-TER-----------LYDSLFVSSDDE-------
--PGSYYPLVALTTRHSADF----RWIEIEMNPKARFHDGSPITAADVAFTYNMFMTQG-VPQFRI-YFKDV
-TAKAVAPLTVRFDFPVS--DKNRMFSLMT-LPIMPEKFWK-------NHKLSDPLSY-PPPASGPYRITAY
RTGQYVTYSRVKDYWGADLPVNKGQYNFDKIRYDYYLDDSVALEAFK-AGAFDLREEG--SPKHWATQYQ-G
GNFARGYIIKQDQVN--QSAQDTRWLAFNIQRPLFQDRRVRQALALAFDFNWMNKALYYNAYQRTDSYFQNT
EYAAK----GEPSAEELAWLTPLK----DKVPA------EVFG-PSYQPPSSDGSGYD----RQNWLKALKL
LEEAGWELKD---------------------------------------------------------QKLVN
SKTG--QPFTFELLLPSA---GNSQYVLPFQQSLKKLGITMNVRNI-DSTQFNNRIR---KRDFDMTA----
-TVYSAFLYPS-ADLQIRWSSQYID--STYNRPGVSDPAIDSLIEEIVKHQGQKVPLLSLGRALD-RVLTWN
QLMIPMWYSN---------HDRFAYWN-KFAMPAVRPAYS-----LGFDGWWFDTKQAATLPAERR------
----------------
>Samsonia/1-594 erythrinae
-----------------IAALLLCATQFGLHAETIEHSTRFALLGE--P-KYAENFSHFDYVNPDAPKGGSI
TLSALG--------TFDNFNRYAL-------RGLPAAR-TEQ-----------LYDSLFVAAEDE-------
--PGSYYPLIAEKTRYPADF----HWIEIDINPKARFHDGSPITAADVAFTYNMFMTQG-VPQFRI-YFKGI
-TAKVVAPLTVRFDFPVP--DKNRMFSLLT-SPVMPQAFWK-------NHKFNEPLGY-PPPASGPYRITAY
RTGQYVTYSRVKDYWAADLPVNKGQYNFDKIRYDYYLDDSVALEAFK-AGAFDLREER--SPKHWATQYQ-G
GNIARGYIIKQDQVN--QAAQDTRWLAFNLQRPLFQDRRVRQALALAFDFNWMNKALYYNAYQRTDSYFQNT
EYAAK----GEPSAGELSWLTPLK----DKVPA------EVFG-PSYQPPASDGSGYD----RQNWLKALKL
LEDAGWVLKD---------------------------------------------------------QKLVN
RKTG--QPFTFELLLPSA---GNSQYALPFQQSLKKLGITMNVRNI-DSTQFNNRIR---KRDFDMTA----
-TVYRAFLYPS-DDLEIYWSSQYID--STYNRPGVSDPAIDSLIAEIVKHQGQKAPLVALGRALD-RVLIWN
QFMIPMWYSN---------HDRFAYWN-KFAMPAVRPAYS-----LGLDGWWYDVKKAATLPA---------
----------------
>Dickeya/1-603 chrysanthemi
-----------MFTRAILAAMLSASVWLAHAAPVTQQTYAFATLGD--P-KYSQGFTHYDYANPSAPKGGKI
TLSALG--------TFDNFNRFAL-------RGLAAAR-TES-----------LYDSLYVSSEDE-------
--PGSYYPLIALTARYASDY----SWLEVDMNPAARFHDGSPVTAADVAFTFNMFMTQG-VPQFRG-YYKGT
-EAKAITPYTVRFTFPDP--DKEKMLGMLT-LPAMPEKFWR-------DHKLSDPLST-PPLAGGPYRITDY
RMGQYVIYSRVTDYWAADLPVNRGRYNIDQLRYDYYLDDSVALEAFK-AGAFDLRIEG--SPKHWATQYE-G
GNFARGYILKKDETN--QAAQDTRWLVFNTQRPQFSDRRVRQALALAFDFNWMNKALFFNSYQRPYSFFQNT
```

EYAAQ----GTPSAEELAWLTPLK----DKIPA------EVFG-PAYRPEKSDGSGYD----RAGLINALQL
LQQAGWELKN---QVLVN
TQTG--QPFRFELLLPSA---ANAIYVLPFQHSLARLGIRMEVRSV-DSPQFNNRFR---KRDFDMIP----
-KLYPAMPYPS-SDLTVSWSSAYIN--SSYNSPGVQDAAIDQLIDNIIRHQGDKPALLSLGPALD-RVLTWN
QFAIPMWYSN---------HDRFAYWN-KFAMPATRPAYT-----LGIDTWWYDAEKAATLPAARR------
----
>Kalamiella/1-601 piersonii
-------------MLFRLIMLLLVALSTPLRAEVVHESRAFATLGE--P-KYAADFSHFDYADPAAPKGGKL
IQAVVG--------TYDNFNRYAS-------RGNPGAG-TEA-----------LYDRLFVGSDDE-------
--PGSYYPLIAESARYADNF----RWMEIRLNPLAQFHDGTPITAQDVAFTFDKFMREG-VPQFRV-VYRGV
-TIKALDSQRVRIDLPKP--EKDLMLGLLT-LPVLPQKEWQ-------DKKFNEPLSS-PPLSSGPYRISRY
KLGQYIEYSRVKNYWAADLPVNRGRFNFDTLRYDYYLDDNVAFEAFK-AGAFDLREET--SAKKWATQYR-G
RNFDDQQIVKETTPN--RVSTNTTWLAMNNEKALFRDRRVRQAITLAFDFEWMNKALFYGAYQRVSSYFQNT
EYAAQ----GLPDAAQQALLAPFK----EQLPP------EVFS-APYAPPRTDGSGYD----RANLLKALDL
LRQAGWQVKN---QQLVN
DKTG--QPMRIELLLRSGA-SN--DWALPFQHSLSRLGISLTLRVV-DASQYLRRLR---EGDYDMIA----
-RVYLAMPTPS-SDLQTSWQSDFID--SSWNSARLKDPLVDHFVKAIVAHQGDKAALLPLGQALD-RILLWN
AYMIPMWYNA---------EDRMAYWD-KFSHPAVKPAYA-----SGLENWWYDVNKAARLPAERR------
----
>Musicola/1-578 paradisiaca
------------------------------------QAFAFTTLGT--P-KYNSQFTHYDYVNPNAPKGGSI
TLSAPG--------TFDNFNRYAQ-------RGLAAVR-TEN-----------LYDSLYVSSEDE-------
--AGSYYPLIALNASYASDF----SWIEIAINPQARFHDGNPVTAADVVFTFNMFMTQG-VPQFRV-YYKGT
-EAKASGPLTVRFSFAEP--DKEKMLGLLT-LPIMPAQFWK-------DHKLSDPLSA-PPPASGPYRISDY
KTGQYVIYSRVPDYWAANLPVNKGRYNFDHIRYDYYLDDSVAMEAFK-AGAFDFRLEG--SPKHWATQYE-G
GNFTRGYIVKKDLTN--QAAQDTRWLVFNIQRPQFSDRRVRQALSLMFDFNWMNKALFFNSYQRASSFFQNT
EYAAQ----GVPDAGELKWLAPLK----DKLPP------EVFG-PAWQPPQSDGSGYD----RNNLRQALQL
LQQAGWELRG---DKLVN
KQTG--APFRFELMLSTA---SNSLYALPFQHSLQRLGIQMDIRSV-DTPQFTSRIR---KRDYDMTP----
-RLFSATPYPS-SDLAILWSSAYIN--SSYNAPGVQDPAIDRLIDDIVRHQGDKTALLPLGRALD-RVLTWN
QFMIPLWFTN---------HDRFAYWN-KFAMPAQRPAYT-----LGFDSWWYDADKAATLPATRR------
----
>Biostraticola/1-602 tofi
------------MLKRLGVALWLAALSPLTQAATVKESTSFAIIGA--P-RYPPDFRHFDYVFPAAPKGGQV
TMATLG--------TFDNFNRFAL-------RGAAAIR-TEQ-----------LYDSLFTTSDDE-------
--IGSYYPLVGQSVRYAADY----RWAEVTLNPAARFHNHQPVTAEDVAFTFQKFMTEG-VPQFRL-FYKDV
-KVIALSRSVVRYEFKQP--SKDGLLGLFT-LPIMPKSFWS-------QHKLSDPLSF-PPPAGGPYRITAW
KSGQYVTYSRVKDYWAATLPVNVGQFNFDSLRYDYYLDDNVALEAFM-AGAYDVRVES--SPKNWATYYR-G
GNIARGFIKLKTMED--HIPVDTRWLAFNLQRPLFADRRVRQALTLALDFEWINRALFYQGYQRASTYFQNT
DYAAS----GRPDAAELALLTPLK----QHIPA------EAFG-PAWRPPVSDGSGYD----RRNRLQALEL
LRQAGWELNQ---RQLVN
SKTG--QRFSFELLMPSG---GNNQYVLPFQHSLQRLGINLRIREV-DNSQFTNRLR---SRDFDMLP----
-TQYRAVPWPG-TDLPISWGTAYLN--STHNTPGVSNPAVDALLDQIIQHQDDEKALVPLGRALD-RVLTWN
AYMIPLWYSN---------QTRVAWWN-KFAMPATRPAYT-----LGFNGWWLDTTQAATLPAERR------
----
>Lonsdalea/1-595 iberica
----------------VAFTLLFISSPLSGLAETLQQPFAFTMLDA--P-KYDKNFTHFDYANPEAPKGGHI
TLSALG--------SFDNFNRFAS-------RGLPAVR-TEA-----------LYDSLYVNSDDE-------
--SGSYYPLIANGARYDSDY----RWLEVDLNPQARFHDGSPVTASDVAFTFNTFMTQG-VPQFRV-YYKGT
-TARALTPHTVRFDFGTP--DKDKMIGLLT-LPVIPQAFWE-------HHKFNEPLTR-PPLAGGAYRISDY
HIGQYVVYSRVKDYWAADLPVNKGLYNFDSIRYDYYLDENVALEAFK-AGAFDMRIET--SPKQWTTLYQ-G
GNFSRGYIVKKDVLN--TAAQSTRWLVFNTTRPLFQDRRVRQALALAFDFNWMNKALFYNSYQRANSLFQNT
EYAAT----GSPSASELQWLTPLK----DRLPA------EVFG-PAYRPPESDGNGYD----RTHLLQALDL
LKQAGWELKN---NELIN
PQTG--RPFRFELLISNA---GNAQFVLPFQHSLKRLGIDMTVRMV-DAPQFNNRLR---KRDFDMLS----
-RPFATEPYPS-ANLAISWSSKYLD--SSYNTPGVQDPAIDRLIDEIIRHQGDKSALLPLGRALD-RVVTWH
QFMIPMWFSN---------HDRFAYWN-KFGMPAVRPTYA-----LGFDSWWYDTDKAATLPA---------
----

>Symbiopectobacterium/1-598 purcellii
------------MLKRVATLALLGSLCWGAFADTVQQNTSFALLGE--P-KYPEGFTHFGYVNPDAPKGGSI
TLSALG--------TYDNFNRFAL-------RGVAAER-TER-----------LYDTLFTSSDDE-------
--PGSFYPLIALYARHADDF----RWMEVELNPKATFHDGSPILASDVAFTFTMFMTQG-VPQFRV-VYKGV
-TVTAIGPRTVRLEMPEP--DKERLLGLLT-LPVMPESFWK-------NHSLADPLAY-PPPASGPYRITSY
RTGQYVTYKRVQDYWGADLPVNRGLNNFDQIRYDYYLDDSVALEAFK-AGTFDLRVEG--SPKHWATQYQ-G
GNFARGYIIKRDRTN--QAAQDTRWLVFNIERPLFQDRRIRQAIGLNFDFNWMNKAFYYNAYQRVNSLFQNT
EYAAQ----GTPSPEELKWLEPLR----DKIPP------EVFG-PSFQPPESDGSGYD----RAHWQQALQL
LEDAGWELKN---QTLVN
RKTG--EPFSFELLLPSA---ANVQYVLPFQQNLKRLGIDMKLRLI-DSGQFTNRLR---ARDFDMLA----
-RLYPARIYPD-GNLKFGWHTKYLD--STYNTAGVSDPAIDALIEQIDAHQGNKAELLILGHALD-RVVTWN
QFMIPMWYSN---------HDRYAYWD-KFAMPETRPTYT-----LGLDSWWYDAQRAQSLP----------
----
>Limnobaculum/1-604 eriocheiris
------------LVLSIASLFSSSGSYAEDKKEIIKQGTTLSLIGA--P-KYPENFQHFDYTNPQAPKGGQL
TVAAIG--------TYDNFNRYAL-------RGNPLDG-SER-----------LSDTLFASSEDE-------
--INSLYPLIATSVRYVDNY----QWMEVQINPQARFQDGTPITAEDVAFTFEKFMTEG-VPQFRS-IYKGV
-KVTALSPLVVRFDLPKP--DREQMFGLVGGLPVFSKKFWQ-------QHNLGEPLNA-PPLSGGPYTIDSY
KLGQYIVYKRVNDYWAANLPVNKGRYNFDIIRYDYYLDDNVALEAFK-AGAYDFRVES--SPKKWATQYQ-G
KNFDLGYIVKKDWEN--QAAQDTRWISFNIQRPIFSNPKVREAITLAFDFQWMNKALFYSAYQQPRSYFQNT
VYAAT----GLPDKDELAWLMPLK----DKIPA------EVFT-KPYQPPVTDGSGYN----RENLLRATQL
LKEAGWEIKN---SVLVN
SQTG--QPFTFELLLLSGS---DAMYVLPFQQNLAKLGIKMEARYV-DSSQFVSRLR---SRDFDMIP----
-QRYSAMEYPSPVSLMILWNSAYID--STYNRPGISDPAVDELTQLIVTYQGQEKPLLSLGRALD-RVLTWH
HLMIPMWYSN---------HDRFAYWD-KYSMPEIRPKSS-----LGIDTWWYDANKAAHLPEQRR------
----
>Insectihabitans/1-583 xujianqingii
---------------------------------VIKQGTTLSLIGS--P-KYPDNFQHFDYTNPQAPKGGQL
TVAAIG--------TYDNFNRYAL-------RGNPLDG-SER-----------LSDTLFASSEDE-------
--INSLYPLIATSARYVDNY----QWMEVQINPQARFQDGTPITAEDVVYTFEKFMTEG-VPQFRS-VYKGV
-KITALSPLVVRFDLPKP--DREQMFGLVGGLPVFPKKFWQ-------QHNLGEPLNI-PPLSGGPYMIDSH
KLGQYIVYKRVKDYWAADLPVNKGRYNFDTIRYDYYLDDNVALEAFK-AGAYDFRAES--SPKKWATQYQ-G
KNFDLGYIVKKDWEN--QAAQDTRWISFNIQRPIFSNPKVREAITLAFDFQWMNKALFYSAYQQPRSYFQNT
IYAAT----GLPDKDELAWLMPLK----DKIPA------EVFT-EPYQPPVTDGSGYS----RKNLLKATQL
LKEAGWEIKD---NVLVN
SQTG--QPFTFELLLLSGS---DAMYVLPFQQNLAKLGIKMEARYV-DSSQFVSRLR---SRDFDMIP----
-QRYSAMEYPSPVTLMILWNSAYID--STYNRPGISDPAVDELTQLIATYQGQEKPLLSLGRALD-RVLTWH
HLMIPMWYTN---------HDRFAYWD-KYSMPEIRPKSS-----LGIDTWWYDVNKAAHLPEQRR------
----
>Jinshanibacter/1-583 allomyrinae
---------------------------------VIKQGTTLSLIGA--P-KYPDNFQHFDYTNPQAPKGGQL
TVAAIG--------TYDNFNRYAL-------RGNPLDG-SER-----------LSDTLFASSEDE-------
--INSLYPLIATSARYVDNY----QWMEVQINPQARFQDGTPITAEDVAYTFEKFMTEG-VPQFRS-VYKGV
-KVTALSPLVVRFDLPKP--DREQMFGLVGGLPVFSKKFWQ-------QHNLGEPLNA-PPLSGGPYMIDGY
KLGQYIVYKRVNDYWAANLPVNKGRYNFDTIRYDYYLDDNVALEAFK-AGAYDFRVES--SPKKWATQYQ-G
KNFDLGYIVKKDWEN--QAAQDTRWIAFNIQRPIFANPKVREAITLAFDFQWMNKALFYSAYQQPRSYFQNT
IYAAT----GLPDDDELKWLAPLK----DKIPA------EVFT-KSYQPPVTDGSGYN----RENLLKATQL
LKEAGWEIKD---NVLVN
SQTG--QPFTFELLLLSGS---DAMYVLPFQQNLAKLGIKMEARYV-DSSQFVSRLR---SRDFDMIP----
-QRYSAMEYPSPVTLMILWNSAYID--STYNRPGISDPAVDELTQLIATYQGQEKPLLSLGKALD-RVLTWH
HLMIPMWYSN---------HDRFAYWN-KYSMPEIRPKSS-----LGIDTWWYDVNKAAHLPEQRR------
----
>Budvicia/1-583 diplopodorum
----------------------------------IKESYSLSLLGE--P-KYAANFTHFDYANPSAPKGGKI
TIAAIG--------TYDNFNRYAT-------RGNPLSG-NER-----------LIDTLFTSAEDE-------
--ISSLYPLIAESARYTDTY----QWMEIRINPRARFQDGTPITAEDVAFSFEKFMTEG-VPQFRS-FYKGV
-NVRAISTLTVRIELPKP--DRDQMLGIVGGLAIFPKHFWK-------DHNLGEPLNI-PPLGSGPYKIVDY
KLGQYLVYQRVKDYWAAGLPVNRGRYNFDTIRYDYYLDDKVSLEAFK-AGAYDLRIEQ--SPKSWATQYE-G

```
GYFDKGFIVKKDEEN--QAAQQTRWLVFNIQRPIFADPRVRRAIGLAFDFEWMNKALFYGANQQPRSFFQNT
IYEAT----GMPGKDELAWLTPLK----GKIPD------EVFT-QEYQPPKTDGSGNN----RKNLLLATQL
LKDAGWEIKD---------------------------------------------------------KVLVN
TQTG--KPMEFELLLLSGS-SSNSLYVLPFQQSLAKLGIKINVREV-DSSQFISRLR---SRDFDMLP----
-RPYPAIEYPS-SDLMIYWNSAYIN--STYNAPGIKDPAIDELTGKISVYQGQAKPLLSLGKALD-RVLSWH
YLMIPMWYSS---------HDRYAYWN-KFSMPAVRPKGS-----LGFDTWWYDVSKAEQLPEQRR------
----------------
>Pragia/1-605 fontium
------ISFLLFILISALVFSGSSWAEATPDTSKVKESFSLSLLGT--P-KYPKDFKHFDYANPKAPKGGKI
TLPAIG--------TYDNFNVYAL-------RGNPLSG-YER-----------LNDTLFTSSEDE-------
--ISSIYPLIGLSVRYSDSY----QWAEVSINPDARFNDGTAITAEDVAFTFEKFMTEG-VPQFRS-IYKGV
-KVQAVSRLVVRFELPKP--DRDQMLGLIGGLPVLPKHFWQ-------HHSLGEPLST-PPLSSGPYTISDY
KLGQYIVYQRAENYWAANLPVNQGRYNLNTIRYDYYLDDNVALEAFK-AGAYDLRVES--SPKNWTTQYQ-G
KFFDLGYIVKKDLEN--HSAQDTRWLAFNVKRPIFADPKVRQALILAFDFHWMNNALFYSANQQPRSFFQNT
TYEAT----GLPDKDELDWLMPLK----DKVPE------EVFT-QTYQPPITDGSGNN----RRNLLQAVKL
LQQAGWEIKN---------------------------------------------------------NQLVN
SKTG--QPFTFELLLLSGS---DALYVLPFQQSLAKLGIKMNVRTV-DSSQYVSRLR---SRDFDMIP----
-RRYPGMEYPS-TSLLIYWNTAYLD--STYNTPALSDPAVDELTQKISDHQGQEKPLLSLGRALD-RVLTWH
YLMIPMWYTN---------HDRFAYWN-KYAMPAIRPKSS-----LGLDTWWYDADKAAQLP----------
----------------
>Zophobihabitans/1-572 entericus
----------------------------------------FAILGQ--P-KYSDDFTHFDYVNPQAPKGGTI
KFAALG--------NYDNFNRFSR-------RGAPEGR-SGE-----------LFETLFTMSEDE-------
--KGSYYPLLAESVTYSADY----SWAEVSINPAARFHDNSPVTAEDVEFSFDKFMTEG-VPQYRS-YQQGV
-TVETIAPLTVKITLPEP--NRERLIGFLSSLRILPKHFWQ-------DHRLDEPLST-PPFGSGPYYISDY
KLGQYAVYQRDPNYWGKDLPVNVGRYNFDQIRYDYYLDDSVALEAFK-AGAYDFRLEG--QPKNWNTQYT-G
DNFDQGYIIKTELDV--TEAPNARWLAFNINRPIFSDRKVREALTLAFDFNWLNRAFYYDTYKQPQSYFENT
EYAAS----GLPTEEELQWLTPFK----EVIPP------AAFG-EAFQLPPSDGSGFN----RDNLLKADQL
LKEAGWIIEN---------------------------------------------------------QQRVN
AITK--EPFEFELLLYMGS-NL--QYVQPFQKNLQRLGINMKISMV-DYAQINSRMR---VLDYDMLP----
-SVYYAYTYPA-SSLKIMWGSDYLN--SSWNSSGLHNEAIDSLLQSIENNQDNADNLLSLGRALD-RVLTHE
YAMIPMWYPR---------YTFYAYWN-KFSLPEIKPKYA-----MGLDTWWYDKDKVQILPT---------
----------------
>Plesiomonas/1-588 shigelloides
----------------------------FTAHAQINEGYAFAAIGS--P-KYAAEFSYFDYVNPAAPKGGNV
TLHAIG--------TFDNFNAYAQ-------RGVPASD-SST-----------LYDTLFTSSADE-------
--IQSYYPLIAEFARYPDDF----SWMEVRINPNARFQDGKPIRADDVVFSFNKFMQQG-VPQFKT-YYQNV
KTVEALTPLTVRFELKSP--DRALLLSLIGGLQILPKHFWE-------NRNLAEPLMT-PPVGSGPYKITDY
AMGQYVVYQRVADYWAANLPVNRGRYNFDTIRYDYYRDDTVALEAFK-AGEYDFRQEY--VAKYWATLYT-G
PNFTKGYIIRENIPH--SIPQGMQALVFNTQNPLFKERNVREALNLAFDFEWLNKTMFYNQYTRTRSIFQNT
PYEAK----GLPSEQELMVLMPFR----QQVPE------EVFT-DEYQPSKTDGSGNN----RPNLRKAMEL
LEQAGWEIRD---------------------------------------------------------RKLVN
KETG--QPFIFELLTYSSS-NE--RIAAPLKQNLARLGITMNIRVV-DTSQYINRLR---KRDFDMIA----
-SGYAAAPFPS-PDLKLAWHSKFLD--STYNLAGVTDHVIDTLTEEIDRAQSDPEKLLALGKVLD-RVLQWN
MFVIPQWHLS---------NFRVAYWN-KFGKPAVRPAYGLG-----FDTWWVDINKAAKLPKARQ------
----------------
>Photobacterium/1-585 galatheae
-------------------------------AVEVIESASLVGFGT--A-KYAPDFKHFDYVNPNAPKGGSI
TYAEVG--------TYDSFNRYAS-------RGVSVAG-ADE-----------IYDTLFAPANDE-------
--IDSYYPLIAQKARYADDF----SWMEIDVNPKARFHDGQSITAKDVAFTFDKFMKEG-VPQFRV-FYKDV
KSVTAISPLTVRIEMSVP--NREKLFSLVEGMNVLPAHFWK-------DKKLSEPLTE-PPIGSSAYKITSY
QAGQSVTYSRVKDYWAADLPVNVGRHNFDTIRYDYYRDDTVTLEAFK-AGEYDFRQEN--VAKFWATLYE-G
TNFENGYIKKEEIPH--EIPQAMQGFVFNTQRPVFQDVRVREALNYAMDFEWMNKTLFYNQYTRPRSYFQNT
PYEAK----GLPTQAELKYLAPLK----EQIPP------RVFT-ETYQPPVTDGSGRI----RQQARKAFAL
LKEAGWALKD---------------------------------------------------------QVMTN
VKTG--EPLAFELLIYSPT-TE--RIAIPLQKNLKQLGIDMKIRTV-DTTQYIKRLR---DRDFDMVS----
-SGYSANPYPT-QNLLIVWNSKYLD--STYNTAGVQDPAIDSLTEQIAAHQQDPEALAALGPALD-RVLQWN
FFVIPQWYLS---------TFRIATWD-KFDRPAVRPKYALG-----VDTWWVDTAKAAKLPEKRR------
```

>Thaumasiovibrio/1-563 subtropicus
-------------------------------------SHALAMHDV--P-KYDADFQHFAYVNPDAPKGGTL
RLSAIG--------TYDNFNRYAS-------RGLAAER-SGE-----------LYDTLYTSSDDE-------
--LGVYYPLIATQAEYSESW----NWVAVDLNPNARFHDGEPITAEDFAFSFQKFLDEG-VPQFRR-YYADV
S-VYVEHPHRLIITIAQP--DKEKLFSILA-LPVLPEHFWQ-------DKDFSEPLSV-PPVGSSAYYISDY
KTGQQITYSLKEDYWGADIPVNKGRYNFEKIIYDYYRDSNVSLEAFK-AGEYDLRSES--SAKNWATLYT-G
PAFDNNQIIKDVIPH--SIPRPMNALVFNTARPLFSDARVREALAYAFDFEWMNKALFYNAYSRTRSYFQSS
PYEAT----DLPSEEELHYLTPLK----AQLPA------RVFT-ESYQPPVSEGDGLI----RRNLLVAKNL
LADAGWIVRN---QKLVN
AETG--EAFTFELMIYTPD-QE--RTALPFQANLKRLGIDMNIRMV-DTSQFTNRMR---NQDYDMID----
-RGFNSNQYPS-GGLKITWHSEFMD--SSYNAASANEPAIDTLTELIAQSQGDGEALAALGPALD-RVLQWH
FYVIPKWHLS---------AYWVAYWD-KFAKPETRPDYSLG-----LDTWW--------------------
----
>Vibrio/1-603 pacinii
-------------LSRILLGGLIASSANFTVYAQVIETKILVGFGV--A-KYSEDFTHFDYVNPDAPKYGKI
VYGQVG--------TYDNFNRFAS-------RGVPAAA-SGE-----------LYDTLMYSPSDE-------
--VDAYYPLIAEKVRYSDDY----TWLEVDINPKARFHDGKPITAYDVAFTFDKFMAEG-VPQYRV-YYQDI
KSVTAKSDLTVRIDMAKP--NREKMFSFAQGTRVLPKHFWQ-------DKNLSEPLST-PPIGSAAYQVTDY
KPGQSVTYSLADDYWAANLPVNVGRNNFKQKQYDYYRDDTVMLEAFK-AGEFDFRLEN--SAKFWANSYT-G
SNFDKGYIVKQEIPH--QKPESTQAFVFNTKRDIFSNPQVREALTYAMDFEWMNSNMFYGQYSRTRSYFQNT
EYEAK----GTPSADELAILEPFK----AQLPP------RLFS-EEFQPPVTDGSGRI----RSQMRVAFKL
LKQAGWELKN---KQMTN
TKTG--EPLSFELLIYSPT-TE--RIAIPLQKNLARMGIEMKIRTV-DTTQYIKRLR---DRDFDMVS----
-SAYSANAYPS-PNLMIVWNSNYLD--STYNTAGVTDPVVDHLTMAISKHQQDPQALISLGRALD-RVLQWN
FYIIPQWYVG---------QYRVAMWD-KFERPEILPKYDLG-----TDTWWISEQKAQLLPEKRR------
----
>Gilliamella/1-584 bombi
--------------------------------EVIYHKTIFSLSGQ--P-KYHVDFQHVDYVNPNAPKGGVI
KLAEVG--------SFDNLNRYAS-------RGAPERN-SGA-----------LYDTLFTNTADD-------
--ITSFYPLIATSITYSDSY----RWAEVTINSNARFQDEKPITAHDVEFTFHKFMTEG-VPPYRV-YNKGI
-TVKAIDDHHVRVDIPDS--DREKLFSFVGSMRVIPKHFWK-------DHDLSEPLTT-PPVGSGPYYISEY
KLGQYVVYRRNPNYWARNLPINKGLDNFDEKRIDYYMDDNVSLEAFK-AGEYDLRAED--QPKNWFTQYQ-G
RYFDSNHIIKQEKPV--QTATDTRWLAFNLQKDLFKDIKVREAITLAFDFEWLNHAFYYDSYKRPYSFFENT
IYAAI----GTPSEQELKWLNAYK----DIIPK------RAFG-NAYYVPKSDGQGFN----RDNLLKAAEL
LKQAGWEVKD---GKLIN
TKTQ--QPFEFELISYLGS-DI--KYAIPFQQNLARLGITMNITLL-DSAQQLRRVR---ERDYDMVA----
-RDYYAVNYPS-SNLLVLWGSEYLN--SSWNASGLHNKAIDAIIAEITKHVDNQEELVPLGRALD-RILTQE
YPMIPMWYNS---------KTYYAYWN-KFGQPAIQPTYA-----IGVDSWWYDANKAASLPKNRKH-----
----
>Thorsellia/1-570 anophelis
---------------------------------------SLSILGI--P-KYSENTKHPDYVNPNAPKTGQL
VLSSLG--------TFDNFNRFAQ-------RGVAAER-TGE-----------LYDTLFKQSLDD-------
--AASYYPLIAESISIHPNY----QYAEITLNKNARFHDNSPITSEDVLFSFDKFKTEG-VVQFAK-KFEGV
-SLSIIDKNRFSVTLPTP--DKSLLFDFIN-LTILPKHYWH-------DKKLNEPLIT-PPLGSGPYKVKDY
KMGQYVVYERVKDYWAKDLFINTGMYNFDTIRYDYFLDGNVTLEAFK-SGAFDVQYEN--RAKNWATAYH-G
ENFDNGNILKVERKN--NAAANTQWFAFNITSPLFEDRNIREAIALSFDFEWLNKMLFYNSYTRANSFFQNT
KYAAT----GLPSSDELAILLPFK----DILPK------EVFG-PAFKAPVTDSSGFN----RENLIKALDI
LENSGWSLQN---GKLIN
DESN--KQFEFELLTYSGS-PT--SHLLQFQQALEKIGIIMTIRQV-DVSQYVKRMQ---ARDYDMII----
-KSYPAWVFPD-SNLFYFWHSDYID--STYNASGVANPAVDHLIEQIAEAQGDEARLMILSRALD-RILTWQ
YYMFPQWYTA---------NTRFAHWN-KFGIPEIYPDYD-----IGVDTWWYEQSKAESL-----------
----
>Orbus/1-589 hercynius
--------------------------TYTLPDEKIVTTTAFALIGE--P-KYRADFTHFDYAYPNAPKGGEL
KLAEIG--------TYDNFNRYAS-------RGAPERS-ADD-----------MYETLFMQSEDE-------
--LNSFYPLLATSITYSDAG----KWAEVTINPTAYFSDGIPLTAEDVEFSFTKFMTEG-VSQYRV-YNEGV
-TVKALDKYRVRFELPTA--NRERLLTLVGNFTVLPKHFWQ-------NKNFAEPLTT-PPIGSGPYVLSDY

QLGQYAIYRRDPNYWAKNLSVNQGRYNFDTKRIDYYLDDSIALEAFK-AGEYDFRLEG--QPKNWFTQYQ-G
HYFDDGFIVRQEDEV--TTAVNTRWLAFNLERDVFRDTKVRQALTLAFDFAWLNRAFYYNSYQQPTSFFENT
PYAAK----GLPNDLEREWLTPYA----TIIPP------SVFG-SSYQVPPSDGDGFN----RSHLLQAKAL
LEQAGWVMKD---NQLVN
QQTG--KPFEFELLLYMGS-DI--KYAIAYQQNLAKLGIKMVITTV-DYAQINSRLR---ERDYDMMP----
-TSYPAIPYPT-SQLIILWGSEYLN--STWNSSGLHNQAIDDLIMQIPNYSHDEQKLTALARALD-RILTQE
YPMIPMWYPR---------YIYYAYWH-KFDKPAVKPRYS-----IGLDTWWYDANQAAQLPKNNR------
----
>Gilvimarinus/1-600 agarilyticus
----------VLVSLTFSLLASTVFAAESPDAVKVITSHAFAEHGE--P-KYSADFTHFEYVNPDAPKGGEL
VLSTIG--------TYDSFNRYAP-------RGDADVN-SEA-----------FYDTLMVASDDE-------
--LGVVYPLIAESIEYPEDY----TWVSFNVNPDARFQDGSAITAEDVVFSFNKFKEQG-VSQFKS-YYKDV
ESVTAVSPLKVTFTFNQS--NRDYVSSLAS-LTILPQQYWA-------ERDLSEPLAE-PPLGSGAYKVVDY
KMGQFTAYEVIEDYWARNLPSRKGLLNFKRIRYDYYRDDTVALEALK-AGEYDFRREN--IAKQWAESYD-V
PAVKSGDLVKEELQH--EIPQPMQAFVFNTNKELFKDRRVRQALNYLLDFEWLNKNLFYSSYSRNTSYFENT
PYKAQ----GKPEGLELEILSPFK----DQLPA------EVFG-EVWRPNTTNGDGNI----RSEMRQALRL
LKEAGWKLKN---KKLVN
SRSG--EPFTFEMLLYSPT-ME--RVVLPFKRNLERVGIDVTIRMV-DSTQYLNRLR---SRDFDMTP----
-QGYRAVPYPS-ANMKFAWHSDFID--STYNQAGVSNPVIDELLEQIVQAQGDDERLLALGHAFD-RVALWN
FYVIPQWHSK---------SFRIAYWN-KFSRPDVRPKYAVG-----LDSWWYDADKAKSL-----------
----
>Sediminispirochaeta/1-599 bajacaliforniensis
----------------RVILSCIMLLLTLTAWSEVTISHALSIRGT--P-KYGPDFQHFDYVRPDAPQGGTL
RRYNIG--------TYDNFHRYAL-------RGVPDSA-STA-----------LYDTLMTYSADE-------
--VESVYPLIAEKLEYPDDF----TWVIFHIDPRARFQDGEPITAEDVRYSFTTFMEKG-VPQFRS-YFKPV
REVEVLDRLRIKFTLEKG--DREMVLSLAS-LTIIPRQWWK-------DHDFSEPQTE-VPLGSGAYTISDY
KIGQYIVYKRLDDYWARDLPVNRGRDNFDYIRYDYYRDSNVAFEAFK-AGEYDLRFEN--TSKNWATLYT-G
PAFDKGYIKKETIEH--QLPANMQALVFNIQRPIFSDPKVRRAIGYLFDFEWMNKNLFYGQYARTRSFFQHT
EYEAK----GLPSPEEKKILEPIR----DQIPP------ELFT-QAYDPPKTDGSGNI----RGQMRSALAL
FKEAGWEVRN---RKLVN
AATG--KAMEFELLLADAS-LE--RVALAFQKNLERVGITMNIRVV-DVSQFTNRLR---ERDFDMIS----
-SVYRARRYPD-SGLLLPWHSAYLD--STYNTAGVSDPAVDYLIEGIIRNQENGQQLIYWGRALD-RVLSWN
FYLVPEWHNS---------AYWISYWN-KFSRPPLLPKYSTG-----VDCWWYDEQKAAMLPEEHR------
----
>Desulfobacula/1-599 toluolica
--------------KTIIMLIILWMTGICPADEASIISTDFSLRDT--P-KYGQGFTHFDYVNPNAPKGGSV
TLSTTG--------TFDSFNRYAQ-------RGDAAAG-SEQ-----------LYDTLMTPSDDE-------
--IDVLYPLIAEKIEYSKDY----TWIIFTINKNARFQDKKPITSADVVFTFNTFMTQG-VPQFKS-YYKNV
SHVEALDTHRVKFTLKKS--RLELLHSLAG-LAILPKHYWE-------TRDFSAPTTD-VPLGSSGFTIDTY
KMGQYVVIKRLKDYWAKDLPVNKGRHNFDLIRYDYYKDEIVTLEAFK-AGEFDFRMEN--VAKQWATMYK-G
DYFDQHHIIKEEIAH--DIPQAMQALIFNTQKEVFKDVRVREALTYAMDFEWMNQHLFYNQYTRTRSFFQNT
EYQAK----GLPSKDEIACLEPVK----DKIPP------RVYT-SEYQPPVTDGSGNI----RSQLRTALGI
LKKAGWQISN---RKMTH
QKTG--QLMEFELLLYSPT-ME--RIAIPFQDNLKKLGITLNIRRV-DTTQFINRMR---ARDFDMIT----
-GQYAANPFPS-SNLKIVWHSNFFD--STYNTAGVQDPAIDFLTEKIDQNQTDKTALLHYGRALD-RVLQYN
FFVIPEWHLS---------KFRVAYWN-KFSRPAVRPRYAIGF----IDTWWIDKAKQAALPN---------
----
>Marinimicrobium/1-576 agarilyticum
----------------------------------IIKSRSFAVQGT--P-KYQPDFKHFDYVNPNAPKGGEL
RLSSVG--------TFDNFNRYAM-------RGNPVVR-SDE-----------LYDTLMVPSLDE-------
--IDVHYPLIAESLEYPENF----AWVIFHIDPRAQDHSGNAITAEDVAFSFNKFMTQG-VPQFRS-YFKNV
EKAEVLDKQRVKFHLKEP--NRDDIASLIS-LTVFPKHYWE-------ERSLEDPLSE-PPVATGPYKIASY
KMGQSVTYERVKDYWAKDIPSRKGTMNFDTIRYDYYRDTTVSLEAFK-AGEYDFRQEG--VAKHWAEDYD-I
DAVKRGDIVKEELAH--SEPQAMKAFVFNTQHELFKDRRVRQALNYALDFQWLNQNLFYNQYERTVSYFQNT
PYMAK----GKPSEREKDILTPFK----ESIPE------EVFG-EVWRPNVTDGSGNI----RTALRKAMAL
LKEAGWELKD---GKMVH
GETG--KPFSFELIYYSPT-TE--RYALPFKRNLERLGIEVNLRQL-DSSQFLSRMR---DQDYAMID----
-RGFQASPYPS-STLPIVWHSDYMD--SSYNQAAVNDPVVDALMEAIVEHQQDPEALLAHGRAFD-RLALWN

FYVIPHWHSG---------EYRVAYWN-QFSRPEQRPKYDLG-----LETWWYDAEKAKSV-----------
----
>Gayadomonas/1-597 joobiniege
-----------------LFSLIIFLHSVSVLAADVITTSTIALRGE--P-AYQTGFKHWDYVNPNAPKGGLI
QYAQRG--------TFDNFNRYAQ-------RGSAPSMIEDL-----------LYDTLMASNSDE-------
--ISVYYPLIAKQLTYADDY----SWVKFKIDDRAKFHDGSAITAEDVEFSFNLFWEQG-VPQFKK-YFAGV
-TVSANDDNTVTFKLAAP--SRSLMLSLCD-LTVLPKHSYK-------DKAFSEPFAK-PPLGNGPYQVKSY
EMGQYIVYQRVADYWAADLPVRRGLNNFDEIRIDYYLDETVLMEAFK-KGEYDFRLEN--TAKNWATQYT-G
ENFDKGYIVKEEISH--EQPTGNSAFIFNVQKPQFKDRRVRQALGLLFDFEWTNQNLFYGDYQRNYSYFMNT
EFAAR----QMPSEAEKAVLMPFK----QQLPA------EVFN-KVYQPPKTDGSGNI----RQQMRQALAL
FKAAGYQLKN---KQLVD
K-NG--QQFEFELLIFRPS-EE--RFAIPFKKNIERIGAKMNIRLLTDASQYINRVR---EREFEMIS----
-RAFSG--YPS-ETLKIQWHSDYIN--SSYNFVGPNNPVVDHLVDKIAEYQEDLPQLTTYARALD-RVLLWN
YYSIPQWHLS---------KYRVAYWD-KFSRPDTLPKYELG-----ESSWWYDKNKAAQLPAKQRK-----
----
>Frischella/1-583 japonica
----------------------------ATSSEKIYTTTHFALFGQ--P-KYAENFTHFDYVNPNAPKGGII
RQAEIG--------SFDNFNRLAS-------RGVAERH-SHS-----------LYETLFTTSGDE-------
--LDSYYPLLASEITYSDQY----KWAEVVLNPNACFSDGVAVTAYDVEFSFNKMMAEG-VSQYRM-YYQGY
-SVKALDKYRVRFELPTP--NREDLLSFVGDFSVLPQHFWQ-------DKNLAEPLAT-PPIGSAPYVISDF
KLGQYAIYQLNPNYWGNNLSVNQGLNNFEIKRIDYYSDDDVALEAFK-AGEYDFRSES--QPKKWFTQYQ-G
KYFDQEYILKQQDDV--TKAINSRWLAFNLERPLFQDIKVRQALTLAYDFKWLNHVFYYDSFIQPMSFFSYT
PYAAL----GKPSERERQLLMPYA----AILPD------TVFG-EAYNIPSSNGDGFN----RANLLKAKQL
LQQAGWVIKD---HRLVN
IQTG--EPFEFELLTYMGA-DN--KYVIPFQQNLAKLGIKMNVSSV-DYAQITRRLR---KRDFDMIP----
-TNYSKIDYPT-SGLMILWGSEYLN--SSWNTSGLHNAAIDSLIALIPNYIDDEEELLYIGRALD-RVLTHA
YPMIPMWMPR---------QIHYAYWN-KFDKPKIKPVYS-----IGVNTWWYDADKANKLP----------
----
>Nitrincola/1-591 alkalilacustris
-------------------LLAFSLAAEEQSAPQLIKSHAFAMHGE--P-KYGPDFTHFDYVNPDAPKGGHM
VMSAIG--------TYDNFHRYAQ-------RGSAAIN-STE-----------FYDTLMIGSDDE-------
--IDVYYPLIAESLEYPEDY----TFVIFNLNPDARHQDGEPITAADVAFSFNKFMTEG-VPQFRS-YYAGV
TDTEEINPQRVKFSFSGP--NKDYISGLAR-LVILPKHFWE-------ERDFAEPLSV-VPLGSGPYTVSDF
SMGQNVTYKRLDDYWAADLPSRKGTLNFSTVRYDYYRDSTVALEAFK-AGEYDFRQEN--SARQWVQDYT-G
PAFTRGDIKMEELPH--QIPQGMQAFTFNIQRELFSDRRVRQALNFALDFEWMNKNLFYDQYERNTSYFQNT
PYMAS----GKPEGLELEILEAYR----DRLPE------EVFG-EVWTPNETDGSGNL----RRETRQALAL
LKDAGWELRN---QQLTH
VESG--QRFEFELLINSPT-DE--RVALPLQRNMERLGIKMNIRLV-DPTQFINRLR---SRDFDMIA----
-RGYSALAYPS-VSMRILFQSDFID--STWNTAGIEDEVIDSLIEGIIAAQEDEEMLLAYGRAFD-RVARWN
FYVMPQWHSN---------LFRIAYWD-RFSRPETRPVYELG-----LDTWWYDEEKASRI-----------
----
>Marinimicrobium/1-578 koreense
--------------------------------ATPIIKSHAFAARGE--P-KYGPDFEHFDYVNPDAPKGGQI
VLAAIG--------TYDSFNRYAQ-------RGD-AET-SVE-----------LYDPLMAASDDE-------
--IGVYYPLIAESLEYPENY----AWVIFNINPEARDQAGKPITAKDVAFTFDKLMDQG-VPFVSN-HYKNV
IQAEVLDDHRVKFHFETP--SREDIQNLVD-FPVFPAHYWQ-------DRDLSEPLDK-PPVGSGPYRISEY
KMGQSVTYELVDDYWAADLPSRKGLNNFKTMRYDYYRDTNVALEAFK-AGEYDFRQEN--VAKQWAEDYD-V
AAVTRGDIQRDELAH--DIPQPAQGFVFNIQRELFADRRVRQAINYALDFEWMNKNLFYDQYQRTYSFFQNT
PYMAE----GEPSEREMSILEPFR----DQLPE------EVFG-KVWVPNQTDGSGNI----RPALRKAMAL
LKDAGWELKD---GKMVH
GESG--KPFEFELLIYSSS-GE--RIGLPFKRNLERLGITMNLRQV-DSTQFLNRLR---SRDYDMIF----
-QGYRANPYPH-PNMRITWHSDYVD--STYNQAGVQDPVIDALIRGIEENQQNDDRLLAYGHAFD-RVARWN
FYLVPHWHSS---------YFRIAYWN-QFARPEKRPEYAIG-----LSTWWFDDEKAKKL-----------
----
>Saccharospirillum/1-600 mangrovi
------------MRASIAALVALCLSFPVVAEDDIIVSYAIAERGE--P-QYPENFEHWDYVNPDAPRGGYV
TYGVRG--------TFDNFNRFAL-------RGTTPAGIRTY-----------LFDTLMTGNGDE-------
--TGVLYGLIAEEVEYPTSH----DWIIFNLNPAATFQDGSPIEASDVAYTFNILMTDG-VPQFRT-VYEGV

-TVEVLDERRVRFNLPES--NLSNLVGLAS-LPVFPEHDFA-------DRDFAEPFID-VPLGSGAYTISDY
EMGQYVVYERVENYWAADHPAMVGQLNFDQERYDYYLDDTVLLEAFK-KGEYDFRQEN--TAKDWATQYT-G
ENFDAGYIIKEEIPH--NLPQNMQSFAFNIERPQFQDRRVRMAINLLFDFEWTNRNLFYGSYTRTSSYFENT
PYSAH----GLPEGKELEILEEFR----DELPE------ELFT-QEFHNFATDGSGNV----RPQLRQALTL
LRESGYESRD---GQMVN
ASTG--EPLSFELLLYSPA-ME--RIAIPFKENLARAGITLNIRTV-DVTQYTNRMR---ERDYDMIV----
-SQLGGGAYPS-DNLVLEWDSRYLD--STYNAVGTNDPVIDALVRGIADNQQNDELLLAYGRAFD-RVLLWR
YYVIPQWHID---------HYRVAYWN-KFGRPAQIPRYSLG-----SSAWWFDAKAAATLPA---------
----
>Breznakiella/1-585 homolactica
------------------LLFILAVLPVQGQSEKTIVTDHIAMRGE--A-KYETGFTHFEYVNPDAPKGGTV
ILHAIG--------TYDNFHAYAL-------RGDRAAG-WTY-----------YYDTLMVNSYDE-------
--DESMYPLIAEKIEYPEDY----SFIIFYINPKAADQDGVPITAEDVAFSFNIFYEKG-VPQFRV-YYKDI
-TATVIGSHAVRFDLPEPG-NRELMMALCG-LTVLPKRFWE-------SRDFSEPLII-PPVGTGPYRVGDY
KMGQYVTLERVKDYWAADLPSRKGRYNFDRIRYDYYRDDTIALEAFK-SGEYDFRSES--NLMNWVTQYT-G
PAFNAGTIIKEVIPH--TMPRPLIGFNINTQRPVFQDQRVRRAINYAMDFEWINKNLFYSQYTRTRSYFTNT
MYEAT----GLPSREEIEILEPIR----DSIPP------EVFT-EEYNPSRTDGSGFI----RPQMREALAL
LKEAGWELR---NGKLIS
AETG--RQMSFELLIYTSE-SE--RIAIPFQRNLERMGIEMKIRMV-DSSQYVNRLR---SRDYDMLD----
-RGYEAQIYPG-SSLALFWHSDYIE--SSYNMAGVTDPAIDYLIDGIIASQEDEDALLAWGRALD-RVLSWN
YYIVPQWHTS---------EFRVAYNK-KLARPEIPPTYSLG-----FDTWWLE------------------
----
>Treponema/1-565 zuelzerae
---------------------------------------SVAVQGV--P-KYADGFEHFDYANPGAPKGGTL
RRGTTG--------TWDNFNRYAS-------RGVSGAGAGDL-----------FYDTLMAGSLDE-------
--ADVYYPLVAEKVEYPRDF----SWIIFHIDPRVRSRDGVALTARDAAFSFEKFMAEG-VPQFRM-YYKDV
-KAVVLDDRRVRFELPVP--DRSMALSLAG-LKMLPKHWWS-------SRNLADPLTE-VPMGSGPYTVSDY
KIGQYLVYERVKEYWAADLPVNRGLNNFDYIRFEYYRDDTVAFEAFK-AGEYDFYLEN--IAKNWATLYT-G
KQFERGLIKRETIPD--LRPQGMSAFTFNTERAIFSDRRVREAVGCAMDFEWMNKNFFYGQYVRSRSYFTNT
PYEAR----GLPTPAELAILEPIR----ESLDP------RVFT-EEYRPPETAGTGNI----RPQMREALRL
FREAGWEIDA--QGKMRN
QTTG--EQFRFELLVYSPA-ME--RIASPLKRNLERMGILMDIRIV-DTTQYTNRMR---NRDFDLID----
-QGYGALYYPS-TDLELPWKSAYID--STYNLAGVRDPAIDYLVDGIVANQENAEGLLAWGHALD-RALTWN
FYVIPRWHLA---------QYRVAYWD-RFDRPATPPRYDLG-----LDSWWFD------------------
----
>Reinekea/1-594 marinisedimentorum
----------------LLFAGLLLMPLLSAAEVNVIEAQSIALRGE--P-KYADDFTHFDYVNPDAPKGGVL
RNEAIG--------TFDSFNRYGQ-------RGDSVVG-ADA-----------LFDTLMVSSSDE-------
--LDVYYPLIAERIRYADDY----SFLTFILNPAARYSDGKQITASDVLFTFEKFKAEG-VPQFAK-YYEFI
KNVEVKGDLEVTFYMEGA--DREKFLSLID-LPVFPEHFWK-------DHNLSEPLKV-VPVVSGPLLIKDF
KFGQYIVYERNKDYWAKDLPVMKGQVNFDEYRYDYYRDQTVAFEAFK-AGDIDFWDRS--TAKDWATGYN-I
PAIKDGRMIKEEIAH--EIPQNTTGFIFNTKRKIFSDPRVREALAYAMDFEWMNKNLFYDSYIRTNSYFQNT
QYMAR----ELPSEEELAILAPIK----DKVDP------RVFT-EVYNPPVNDGSGNI----RGSLRTALRL
LKEAGWEVKD---KKLTS
VATG--ELFEFELMTYSPV-TE--RIAIPFQENLKKLGIDMSIRQV-DTSQFVNRWH---EHDFDMVS----
-QRYSANPHPS-SSLRIVWHSNFID--SSYNQANVTDQAVDYLVDGIAEHQSDDDALLHWGRALD-RVLLWN
HYLIPQWHYN---------KFRIAYYD-KFSRPDVRPKYSLG-----FDTWWIDPEKEAAL-----------
----
>Catenovulum/1-581 sediminis
---------------------------------TVHKSTSIALRGE--A-KYSQGFKHWDYVNPDAPKGGQI
TMGQRG--------TFDNFNRYAQ-------RGTSPAMIDGL-----------LYDSLMSSNSDE-------
--ISVYYPLIAKQIEYADDF----SWVTFYIDENAKFHDGTAIKASDVAFSYNLFFEQG-VPQFKK-YFEGV
-AVKSVKGNAVTFSLDIP--GKSLMLSLCD-LTVLPEHYYK-------DRKFSEPFEK-PPLGSGPYTIKDY
EMGQYIVYQRKKDYWAAQHPSRVGRFNFDRIKIDYYLDETVLMEAFK-KGEYDFRVES--IAKNWATQYT-G
DNFDKGYIVKDEISH--QVPTGNSAFIFNVQKPQFKDRKVRQALNLLFDFEWTNKNLFYGDYQRNYSYFMNT
EFSVT----DLPNGRELEILSQYK----EQLPS------EIFN-KKFSLNKTDGSGNI----RKEIRQALRL
FKQAGYTLKN---NQLLD
P-TG--KQFEFELLIYRPS-EE--RFAIPFQKNIERIGAKMNIRLVSDASQYINRVR---EREFDMIS----

```
-RSFSG--FPS-ETLKIQWHSDYLN--SSYNFVGPNHPVVDALVEAIAQNQENEAELTAYAKAFD-RVLLWQ
YYSIPQWHLS---------KYRVAYWD-KFSRPDTIPKYELG-----EDTWWYDAQKAKKLPSNKQQ-----
----------------
>Natronospirillum/1-600 operosum
-------------LLVTCLMLSAAALAQATDSPEMIRTHALSILGE--P-KYDADFEHFDYVNPDAPQGGRI
VFAAIG--------TYDNFNRYAQ-------RGLAATG-STL-----------FYDSLMTASSDE-------
--IRTYYPLVAQELEYASDY----SEVTFFLNPDARHQDGEPLTAADIVFTFHKFMDEG-VPFVRS-QYAAV
ADVEALDDYTVRYTFHPDEGSRDLILRIAN-MPILPEHFWA-------ERDFSEPLNE-PPLGSGPYRVSRF
AMGQHVEYERLEDYWARDLPAMRGTMNFDYLRYDYYRDTTVALEAFK-AGEYDFRQEN--VARQWANDYT-G
RAFDRGDIVKEELPH--SVPQPMQAFVFNIERDQFQDHRVRQALNYALDFEWMNRNLFFDQYERTFSYFQNT
PYMAE----GLPSEQELEILEPFR----DQLPE------AVFSEEAWRPNVTDGTGRL----RAETREALGL
LQEAGWELRD---------------------------------------------------------QRLVH
SETG--ERFRFEMLLSSPT-FE--RVALNIQRNVERMGIRMDIRII-DPSQFTNRLR---ERDFDVIA----
-NGYAANPYPS-VSMQTSWHSDYID--STWNTAGVSDPVIDALIEGIIAHQGDEDRLLAYGHAFD-RVARWN
FYVMPNWHSS---------SYMVAYWD-KFDRPETRPDFDLG-----VSTWWYDNDRAARI-----------
----------------
>Hahella/1-593 chejuensis
-----------------VLLLLCAVSLVCYGEPKVITAKGIALRGE--P-KYLDDFTHFDYVNPDAPKGGTI
RLHSIG--------TYDNFNRYAQ-------RGTSGAG-ADE-----------LYDSLLIASQDE-------
--IEVYYPLIAEKIEFASDY----SWIIFHINPKATFQDHKPITAQDVKFSFEKFANEG-VPQFKK-YYSFI
TSIDVLDEHQVKFSMQDA--NREQMFSLFG-LKILPEHYWA-------SRNFGDPIKD-VPLGSSGITISDY
SYGQYVVVKALDDYWGKDLPVNKGRNNITYTRYDYYRDATVAFEAFK-SGEFDFWQEG--EAKNWATAYN-F
PAIRNKLVKKEELAH--SIPQSTTGFVYNTERPIFKDRRVRKALSYLLDFEWMNKALFYGQYVRTASYFTNT
PYTAS----GLPSEAELEILNPLK----DQLPP------EVFE-KPFELPVTKGDGYI----RDNIRIALKL
LKEAGWELRD---------------------------------------------------------GKTVN
AKTG--QQMEFELLSYSPT-TE--RSAAPLRRNLEKIGIIMNLRQV-DPSQYINRVR---THDFDMVS----
-FRYPALSYPS-SDLKILFHSSFVD--STWNSANLRDPAVDAIVEGIEKSQEDPDKLMVYGHALD-RVLLWK
YLMIPQWHLS---------AFRIAYWD-KFSRPETRPKYDLG-----QDTWWFDKDKAAQL-----------
----------------
>Edwardsiella/1-597 tarda
--------MFVSIARPLAALFLLCSPYAAAQADHIDERYALSLFGP--P-RYAAGFNHFDYANPAAPKGGAL
RLAALG--------SYDNFNPYAA-------QGTPAIH-SAR-----------LFDTLFTRSADE-------
--PASAYPLIGSAIRYDRRF----RWAEIRLNPKARFQDHTPIGAADVVFSFQALMQHG-PATFRQ-RFRGV
-TLRPLSPLSVRIDQPGP--DKARIFALLTQLPILPQHAWR-------HQRLDRPRYT-PPIGSGPYRVTCY
QLGHSVTYQRLDDYWASDLPVNRGRDNFTTLTYRYYPNDAAARTAFL-DDALDVRLER--SPAAWAQQYT-G
PAVRQGQIILQVRDS--HAAQATRWLAFNTQLPLFHDRRVRQAISLAFDFPTLNRRWYHAAYRRANSYFQQT
PYAAR----SYPDAAELLWLAPLQ----GQLPP------ALFH-HIYQP-----GGHG----KDALRQAEVL
LAQAGWRRQG---------------------------------------------------------ARLIN
THSG--QRFTFTLLLPYAS-QL--HYVAALGDRLAALGIVMHIRYA-PRAQFATLAR---RHDFGMIP----
-VIYPATPYPS-PALLARWGSRYAD--DGANLANVRNPAVDTLLAAIVARQDDPPSLLALGRALD-RVLTWN
AFMLPLWYTP---------QRRIAYWD-RFAMPSQPVPYD-----LGWDYWWYDVNRAARL-----------
----------------
>Reinekea/1-597 thalattae
-------------MIRKLLICALLVPVLAHAEVTIIEAQSIALRGE--P-KYADDFTHFDYVNPDAPKGGTY
RSEANG--------TWDSFNRYGQ-------RGDPVVL-SGS-----------YYDTLMTSSADE-------
--LDVYYPLIAEKIRYADDY----SFITFFINPAARFSDDKAITAQDVKFTFEKLLSQG-VPFIKT-YYAFV
KEVTIENDHQVTFHLQDA--DRERMIALAG-LAIFPEHFWK-------DHDLSEPLKE-VPVVSGPLLIKDF
KFGQSITYVRNKNYWAKDLPSQKGRTNFDEYHYDYYRDRTIAFEAFK-AGNIDIWIEN--ISKQWATGYD-I
PAVKEGRMIKEEIPH--SVPQGMQAFIFNTQRAPFNDPKVREAMNYALDFEWMNKNLFYGAYKRANSYFQNT
AYMAR----ELPSAEELKILEPIK----NQINP------RVFT-EVYNPPVNDGSGNI----RGSLRQALAL
LKQAGWEIQN---------------------------------------------------------KKLTN
VETG--ELFEFELMTYSPT-TE--RVAIPLQENLAKLGITMNIRQV-DTSQFVNRWH---DHDFDMVA----
-SGFSAMAYPS-SALMNRWRSDLVD--STYNQANVTDDAIDYLVNGILDNLSDDEALLHWGRALD-RVLLWN
HYIIPQWYSS---------SYRVAYVN-KFSRPEIRPTYDLG-----TDTWWIDAEKEAAL-----------
----------------
>Gynuella/1-592 sunshinyii
---------------LIIILLMIFPAFGYPAN--VVVSHAIALRGE--P-KYAQGFEHWRYVNPNAPRGGYV
TYGARG--------SFDNFNRFAR-------RGSS-APLAES-----------MIDSLMTSNEDE-------
```

--DNVLYGLIAQTIEYPDDY----SWVIFHLDPRARASDGVPITADDVVFSFNKFMTQG-IETLAR-LWEGI
-TVTKIDDLTVKFTTPQK--DKSTTMQMAE-LRIFPKHYWK-------DVDLSEPLVE-VPVVSGPYTISDY
SMGQYVVVYKRNKDYWALNHPTQKGLLNFDFVRYDMYKDDTVMLEAFK-KGEYDFRSEP--VAKVWITGYE-G
KNIDQGYIRKEEIPY--SVPKPMIGFIFNVTRPQLSDRRIREAIGYLFDFEWSNTNLFYGLYKRELSYFQNS
DNMAR----GLPQGRELEILNGFK----DQLPP------EVFT-QEFNPPKTDGSGNI----RPQLRQALSL
FKQAGWQLKN---GKLVD
Q-NG--QQLAFELMLWSPT-YE--RVALPFKENLAKAGIDMSIRII-DSAQATNRLR---ERDYDMVV----
-GTLGGGVYPD-NMLQYEFHSKYID--STYNNSGYTSPVVDALVEKIVQNQSNLPELQAYGKALD-RVLLWQ
YLVVPQWYND---------KVRVAYWD-KFSRPEVLPTYGVG-----LESWWYDEEKAKKLP---------
----
>Marispirochaeta/1-655 aestuarii
----------------VLIAAILVFGAAALTAQVTVSHAIALHGE--P-KYGPDFTHFDYVNPDAPKGGTL
RYHSIG--------TFDSFNRYAQ-------RGDTPAG-SET-----------IYDSLMVSSDDE-------
--IDSMYPLIAEKIEYPADY----SYVIFTINPAARFQDGRPIRAKDVVFTVQKFLEEG-VEFIQT-YIPGT
-TGKVIDELTVRFDIPDG--DREKIMYLAS-FPILPPQYWE-------NRDFSEPTTD-VPLGSGAYTISDY
NMGQYVVVYKRIEDYWALDLPVNRGRLNFDFIRYDYYRDENVALEAFK-AGEYDFYLES--IAKNWATLHS-G
PNYDQGYIIKEEVPH--EIPANMQAFVFNTRRPFFSDRRVRQALNLALDFQWMNTNLFYGQYTRTRSYFQNT
EYEAK----GIPSAEELEILEPIR----DKIPE------EVFT-EEYNPPVTDGSGNI----RPQIREALKI
LKEAGWEIREVAAPEKSLEDQEGDSSKEKETEEPGFLGKIWRGIKSFFSAIFSWILGLFGGDSAGGVRRLVN
VETG--EPMEFELINYSPS-TE--RVIIPLQQNLERMGITLNIRTI-DPTQYLNRLR---DRDFDMIS----
-QGFAANAIPG-SGLLLRWHSDYID--STYNQAGVEDDAVDYLLEGIVDNQNDLDALLYWGRALD-RVLTWN
HYVIPQWHLS---------KFRISYWN-KFSRPETRPKYALG-----IDTWWIDTEKAATLPDRFQQ-----
----
>Catenovulum/1-607 maritimum
MSKRTHLLFTCLISLSTFFSFHSSATNSSQNQVKIIKSHAIAMRGE--A-KYPADFKHWDYVNPNAPKGGTI
KTATQG--------TFDNFNRYAQ-------RGSSPSGIDG------------LFDSLMTSNDDE-------
--IQVLYGLIAEYIEYPTDY----GWAIFHINPKAKFQDGKAITAHDVEFSFNLFYTQG-VPQFKK-YFEGV
-TVKALDDSRVKFTLPKK--DKALMVALAD-TTIFPQHYYQ-------DKDFSEPFKS-PPLGSGPYKVFDY
QMGKQITYQIDPNYWAVKHPTNAGRLNFEFEKIDYYLDETVLLEAFK-KGEYDFKQEN--VAKNWVTQYK-G
GNFENGNIVKEEISH--QVPTGNSAFVFNVQKPQFQDRRVRLALSYLFDFEWTNKNFFYGDYQRNYSYFMNT
DYASS----GLPKGQELDILNQYK----DKLPA------ELFS-QAFELSKTDGSGNI----RNQIRSALRL
FKQAGYQLKD---KKLVD
K-NG--KQFEFELLLFRAS-TE--RVAVPFQQNIEKIGGKMNIRLVSDSSQYINRVR---ERDFDMIS----
-RAFAG--YPT-ESLKIQWHTSYLN--STYNWVGANDPMIDNLVDKIAELQQDEVQLTQYARAFD-RILLWN
YYSIPQWHLS---------KYRVAYWN-KFSRPNTVPKYDLG-----ADTWWYDETKASTI-----------
----
>Aestuariirhabdus/1-610 litorea
---------VIIQALLGAVLSINALLLSASELPKVHNSHGIAMHGS---PKYAQGFTHFEYANPEAPKGGLL
RLAVISSG------GFDSLNPFIV-------KGISAAGIGYLG-------SSYLYESLTSRSNDE-------
--AFTEYGRIAERIEWPDDR----SWVTFHLNPKARFHDGEPITAEDVSYTFELLTTQG--HPLYRTYYKNV
KQVSVIDTRTIRFDFEAGDNR-ELVLIVG-QMPILPKHYWQ-------ERDFNSTGLE-PPLGSGPYRIKSV
DAGRSISYERVKDYWGADLPINRGHYNFDEIRFDYYRDDTVAIEALR-AGEYDYREEN--SARNWATLY-EG
KPFEQQLMVKEALPN--SNPTGMQGFVYNTRRELFSDPRVREALAYAFDFEWTNRNLFYSAYTRTESYFSNS
ELAA----RGLPSEEELELLNPYR----DQLP------ERVFSEAYHPPKTAGD-GNI----RRNLGIAMRL
LKEAGWEIRN---KQLTH
SATG--KTFTFEMLLTS---QAFERVVLPFKKNLERLGITMEVRMV-DTQQYIQRVR---SFDFDMIVGG-F
GQ----SSSP-GNEQRDFWHSSQADHQGSRNQIGIQNPVIDGLIDTLIAAGSREE-LITATRALD-RVLLWN
FYVIPNWHIT---------SDRIIYWN-KFGRPQQLPE--YGVS---LDTWWYDASKAARI-----------
----
>Mangrovitalea/1-572 sediminis
--------------------------------KAPQAQYAIAMHGK---PKYGPDFKHFDYVNPKAPKGGTL
KMAAVGN-------NYDSFNPFIL-------KGVAVAGINR------------IYDTLMMSSGDE-------
--PFTEYGLVADSIKVANDR----SWVEFHINPKARFQDGKPITAADVVFSFNILMTQG--QPGYQAYYADV
KSVKAIDSHLVRFDLKKTRNR-ELPLILG-QIPVLPKHYWA-------HHKFDSTKLE-IPVGSGPYRIEKF
DPGSSITYVRDKNYWARDLPVNKGRYNFDKIVYDYFNDDTVALQAFK-AGDLNFRLEN--IAKNWATMY-SG
SKFSSGEIQKEEIPQ--HLPVGMQAFVYNTRRPVFADREVRRALAYAFDFHWTNKALFYGQYQRSDSYFENS
DLAN----QGLPQGKELSLLEPFR----KTLP------PEVFTQHYEPPDTSDK-VTL----RDNLKTAYEI
LKKDGWVIRN---GKMVN

AKTG--QPLSFEILLRS---KSFERVVLPFTQNLARLGIDANIRLV-DSSQYIERMN---HFDFDMAILV-L
PE----SDSP-GNEQRDKWSSHAANTPGSSNYMGVNSPVVDQLVGDIIKADSRED-LVTATRALD-RVLLWG
YYTIPQWFLP---------VSRVAYYR-PIQHPTHYP---KSG-VD-IDTWW--------------------
----
>Marinobacter/1-588 gudaonensis
-----------------MALVAAPLFSAGSLAQEVTPSHGLAMHGE---TKYPEGFSHFDYVNPEAPEGGTL
KLAVVAN-------GFDSFNPFDI-------RGVAAAGISN-----------YLYDTLLASSDDE-------
--PFSAYGLIAESIETPEDR----SYVVFNLREQARFHDGKPITAEDVKFSFETLTTKG--HPFFRNYYADV
SKVTVEGPHRIRFDFGETKNR-ELPLILG-QMPILPAHYWA-------DRDFGDNGLE-RPLGSGPYRIGDF
EAGRSVSYERVKDYWAEDLGVRAGRFNFDRIVYDYYTDDTVALEAFK-AGSFDFRLES--SAKNWATAY-TG
ERFNNGTIIKEAIEH--HRPAGMQGFVFNTRRPVFSDPLVREALAYAFDFEWANKNLFFGQYTRTDSYFENS
ELAS----SGLPQGRELEILEPFR----DQLS------ADVFNEEYQPPTTEGQ-QGL----RQNFKIALDL
LNEAGYEIRN--GKMVN
AESG--KPLAFEILLFQ---KSFERVVLPFKKNLERLGIDVTVRLV-DSNQYIQRLR---EFDYDMITQV-F
GQ----SDSP-GNEQRDYWHSSNVNTKGSRNYAGVSDPVVDELVSKVIQAPNREE-LVHRVRALD-RVLLHG
HYVVPHWHLT---------KDRVAYWN-HLQRPATTP---KNG-ID-LDNWW--------------------
----
>Aestuariirhabdus/1-594 haliotis
------------------------------KATEYHSSHAIAMHGE---PKYPAGFDHFDYANPDAPKGGTL
RLAVISSG------GFDSLNPFIV-------KGISAAGMGYLG-------TSYLYESLTSRADDE-------
--AFTEYGRLAERIEWPEDR----SWVTYHLNPKARFHDGKPITAEDVHFTFNLLTSEG--HPLYRTYYKNV
KKITVLETLSIRFDFEQGDNR-ELALIVG-QMPILPKHFWQ-------DRDFNKTGLE-PPLGSGPYRIQSV
DAGRSITYQRVDDYWGADLAINRGHYNFDHIRFDYYRDDTVAIEALR-AGEYDYRSEN--SARNWATRY-KG
SQFDNNLMIREALPN--KNPTGMQGFVYNTRRELFADPLVREALAYAFDFEWTNRNLFYGTYTRSESYFSNS
DMAS----SGLPSGAELNLLEPFR----NQLP------PRLFTEEYHPPKNSGD-GNV----RRNLGVAMKL
LKEAGWEIRN--KRLTH
TASG--RLFNFEVLLVS---PAFERIVLPFKKNLERLGITMNVRMV-DTQQYIQRIR---SFDFDMIVGG-F
GQ----SSSP-GNEQRDFWHSSQADRQGSRNQIGIKNPVIDDLIEKVISAHSRED-LITATRALD-RVLLWN
FYVIPNWHIT---------NDRLAYWN-KFGRPEIMPK--YGVS---LDTWWIDDAKLARINNAKK------
----
>Desulfovibrio/1-598 inopinatus
--------------LVVVGLLACAGLPASAQTTGSHTSHALALGGT---PKYPADFTHFDYVNPNAPKGGTA
KLSSVG--------TFDTFNPYLP-------KGLPPSGIG------------LIYDSLTVKSDDE-------
--PFTEYGLVAKSIELADDH----SWVAFHIDPAARFSDGEPITADDVVFTFNTLLEKG--GPQYAKYYHDV
KGVTTEGNETVRFTFKNTNNP-ELPLILG-QLPVLPKHYWE-------TRDFTETGFE-LPIGSGPYTIAHF
QPGHTITYKRDPNYWAKDHPVNKGRYNFDEVVIDYYRDETVTLQAFK-AGEYDFRQEY--VSKNWATSY-TG
PAFDKGLIVKKEIPH--NVPQGMQAFFFNTRRDIFKDKKVRQALGLAFDFEWTNANLFYGLYKRATSFFSNS
ELAS----SGLPSPEELAILEPYR----GKIP------DEVFTAVYAPPSTAGK-GGI----RANLRKALTL
LREAGYVVEN--GVLVN
ATTK--KPFTFEFLLSQ---KSMERVVLPFVQNLEKLGIKPTLRVV-DQAQYYNRMR---DYDYDMIVIP-L
PQ----SLSP-GNEQRYYWTSSAADTPGAYNFAGIKDPVIDELVEKIIIAKDRKT-LVTLTHALD-RVLLAG
HYVIPQWYSG---------VYRVAWWD-KFDRPKVHP---LY-DLG-FYTWWIDPAKEAAL-----------
----
>Leucothrix/1-581 arctica
------------------------------EAVTVIEATSIALRGK--P-VYSSDVTHFNYVNPDAPKGGEV
IEWARG--------TFDNFNAYSQ-------RGDAARD-ISD-----------INDSLMESSADD-------
--ISAYYPLIAEKISYNSDY----SAITFHLNKKAVFHDGERIKPSDVKFSFEKLSSEG-LPGLKA-YYSYV
EKIELLDDYRVKFHLSTK--DKGRMIDLVG-FTVFPEHFWK-------DHKLNEPLKI-PPLGSGPYKVGDY
KFGKYVSYDRVKDYWAKDLPIRKGAANIGTIRFDYYLDETVAFEAFK-AGHIDKWSEN--TAKRWATGYD-F
PAIASGQVIKKTTPH--SIPLRASGLIFNLKKPLFADIRVRKAMTQLLDFEWMNKNLFYDQYIRVNSLFMNT
KYVAT----GLPSEAELALLEPLK----DQLPA------SVFT-EPFELPKTNGSGNI----RSNLRQALRL
FKEAGWTVKN--QKLVD
AQ-G--KQFEFEIMLVSAG-YE--KVALAFGKNLKRAGIKLNVRTV-DSSQYMARYS---AHDFDMIA----
-GGYREYSYPS-SGFSQAFESKSVN--SSYNPANLTNDAVDSLLEHMESVQDDEEKLLVVGHALD-RVLMHT
YLMIPTWNLS---------SFRMAHWD-KFGKPETTPLYDEG-----DSFWWVDEKKAATLKS---------
----
>Zooshikella/1-614 ganghwensis
------------INILFLLATLLGFSFGLHAEEKIYKSHAITIHGE---PKYPADFTHFDFVNPEAPKGGTL

HLATISSS------GFDSLNPFIV-------KGIAASGLTPLN-------NNYFYDTLLYHSKDE-------
--PFTMYGLLAEQMEWPENR----AWIIFHLHKNIHFHDGHPLTAEDVVFSFNLLQKEG--HPLYKTYYANV
EHVEALDKWRVKFTFTPGDNR-ELAMIIG-QFAVLPKHYWS-------QHTFNKTSLT-PPIGSGPYTISQV
DAGRSITYQRDPNYWGKDLPVNRGKYNFNTIRYDYYRDPTVAIEALK-AGEYDFRSEN--SAKNWATQY-QG
KLFTNQAMIKKTLPN--LSRAGLQGFIYNIRRPVFADTKVRQALSYAYDFEWANKNLFYNSYQRTDSFFENS
EMAA----SGLPSPAELKILEPFK----KELP------EAMFTQAFELPKTDGS-GNN----RQQLRQALRL
LKEAGYALKE---GKLIN
QQTN--QPFSFEILLAQ---PEMERIVLPFQENLKKLGIEMTIRQV-DAQQYIKRLR---SFDFDMFVGS-F
GQ----SQSP-GNEQRDFWHSSQADITGSRNYIGIKNPVIDQLVDKIITAPSREA-LINYCRALD-RVLLWN
HYVIPQFHIN---------QYRLAYWN-KFGQPEVTPK--YSFT---LDTWWEDPKKAEKIKSFRKKA----
----
>Thioalbus/1-597 denitrificans
---------------------ILALLTTAAPAIGATVSHGLAMHG---DLKYGPGFTHFDYARPEAPKGGDV
RLAAVG--------TYDTLNPFTL-------KGVSASGLGR------------LFDTLTIRSDDE-------
--AFSEYGLLAETIEIPEDR----SWVAYTLRPEARFHDGSPVTPEDVIFSFNTLKEKG--HPFYRAYYGSV
ARAEKVGDRKVKFTFD-AGENRELALIIG-QLPVLSRADWE-------GKDFAATTLK-APLGSGPYEVAAS
DPGRSITYRRVADYWGGDLPVNRGRDNFDTIRVDYYRDATVALEAFK-AGEYDFRLEN--TAKNWATAY-DV
PPVNEGFIKLEEIPN--EQPTGMQAFVYNTRRELFSDPRVREALAYAFDFEWTNKNLFYGAYTRTRSYFSNS
ELAAR---G-LPSPGELKVLEPYR----GKVPE------AVFTTAYEPPASDGS-GNI----RGNLRKATEL
LRQTGWEIR---GRQLVD
SATG--RPFSFEILLVN---PAFERVVLPFVRNLERLGIEARVRTV-DTTQYQNRLN---DFDFDMIVDV-F
GQ----SLSP-GNEQRDFWSCEAARTPGSRNTAGVCDPVVDALVEKVISATDRQE-LIDRTRALD-RVLQWG
FYVIPNWHT---------RVYRVAYWD-KFSRPAVT--PRYDLGFG---FWWVDPDKAAALTAKRGK-----
----
>Halorhodospira/1-578 halophila
--------------------------------------HGLALHG---EPKYPPDFSHFDYVNPKAPKGGTV
IREARG--------SYDSLNGYIL-------RGTKPPGLGM------------VIDTLMVHADDE-------
--PFSVYGLIAERVEVAEDN----AWVEFKLREEARFHDGEPITADDVVFSFEVLREHG--HPRLRSYYRHV
ESAEAEGRHRVRFEFA-HAGNPELPLIMG-ELPVLPQHYWE-------ERDFSRTTMQ-PPLGSGPYRIAEA
RPGRSITYERVEDYWAEDLPVRRGRFNFDRLRYDFYRDATVALEAFR-AGEFDLREEY--TARHWATGY-ET
SAQREGRMVLEEIEH--SRPAGMQGFVFNTRRPVFEDREVRRALSYAFDFEWTNRQLFHSAYTRTASYFENS
ELAA----RGAPGEAEQAILAPFR----EELPE------AVFEP-YRPPVTDGS-GWN----RENLLKALRI
LKEAGWSVGDD---GILRH
RDSG--RPLVFELLLVN---PSFERVALPFVQNLRRIGVLARVRTV-DTTQYQYRLD---HFEFDMAVVV-L
PQ----SPSP-GHEQAMYWSSEAADEPGSRNYAGVRDPVVDELVERLVSAEDRDE-LVHLTRALD-RVLLAG
HYVIPNWHT---------PVHRVAYWD-KFGRPETA--PKYGLGFD---TWWVDPDKEQRLREAN-------
----
>Desulfobotulus/1-603 mexicanus
----------------ICMLLIQGLYFITP--VLAAPSHGIALWG---EPQYGTDFTHFAYARPDAPKGGHL
RMAATG--------SFDSFHPFTI-------RGTAAAGVG------------LLYETLMTASLD--------
-EPSSSYGLIAGSVSVSEDR----TSVTFRLRPEARFHDGHPVLAEDVVFSFQILIREG--SPHYRKYYEDI
TGVHAPDEHTVRFDIR-PGSAMETPYILG-QLPVLPAHVWK-------EREFSRSSLE-TPVGSGPYRIHSF
DAGRRITYIRDKDYWGWHLPVNQGHYNFDFISWEYFRDATVSLEAFK-AGVFDYRLEN--TAKTWATLY-DG
PAFRDGRIRKETIPH--ERPQGMQGFVFNIRKPVFRDRAVRKALSLVFDFEWANTHLFYGQYSRSHSFFQNS
PMAA----EGFPSEEELVLLEPFR----HLLPE------SAFGTAIVPPVTDGS-GNI----RPLLREADRI
LNEAGWILKGGK--RIH
KETG--RPLSFEILLAS---PSFQRIAMPFRQNLARLGVDASVRVV-DRTRYVRQLQ---DFNYDMIVGN-F
RQ----SHSP-GSEQRDFWSSEAASVPGGRNTIGIADPVVDALVEHILNAENREE-LITACRALD-RVLRAS
HYVIPQWFN---------ASYRIAYWD-KLRHPEKSPEHGLGIY-----TWWIDTEAEARLTPGAVMHTE--
----
>Skermanella/1-588 stibiiresistens
-------------MLKRILLIALLTLGQTP-ALAETARHGLAMYG---EPKYPPGFQRFDFVNPDAPKGGSL
VLSAIG--------GFDTLNTFTI-------RGQPAAGVTN------------TFDTLMVESQDE-------
--PFTQYPLVAETVELPPDR----AWVAFNLRPEARFQDGRPITPDDVIFSFDILR-QT--HPFYRAYYANV
AKVERTGDRTVRFTFA-PGDNRELPLILG-QLPVLPKHYWE-------GRTFDRTTLE-PPVGSGPYKIARV
DPGRSVTYERVADYWGKDLPVNVGRFNFGTIRYDYYLDPTVALQAFN-GGLVDFRAEN--SAKNWATAY-DQ
RLVESGRIKKEEIKV--ETPAGMQGFVFNTRRPIFADPKVRYAIAHAFDFEWANAVMFYGAYQRSDSYFENS
DLEAE---G-LPSAAELEILEPLR----GQIPD------EVFTERYEPPSAGDR-RVV----RDNLLKAREL

```
LEEAGWVIR---------------------------------------------------------DGRRVN
ATTG--EPLTFTILLDS---PSFERVALPFVANLRRLGIDARVRTV-DAAQYQNRIS---DFDFDMTTAI-W
GQ----SLSP-GNEQRDYWTSAAADHTGSRNLAGIKSPAVDHLVDLLISANTREE-LRARAAALD-RVLLWG
HYVIPQWYA---------GVSRVAYWT-KLEHPRDL--PPYGIAFD---AWW--------------------
----------------
>Desulfurispirillum/1-596 indicum
----------------LALFFCATLVPLSLQASEPQPVHAIAMHGE---PRYGADFRHFSYVNPDAPKGGRI
SLHSVG--------TFDSLNPFVE-------RGVADSRIT------------LVYDSLLERSADE-------
--PFTEYGLLATKIILPDDR----SWVEFILHPDARFHDGKPVTAHDVVFTFEILRTQG--SPFYQAYYGDI
DSVTAVETRRVRFEFSSAGNM-ELPLIVG-QAAILPKHFWE-------DKDFSRSSTV-IPLGSGPYRVARV
DAGRSITYERVEDYWARNHPIKRGRHNFDAITVDYYRDGNVALEAFK-SGAYDFRLEN--NAKDWAVSY-SI
PAVRQGMIQRENLPH--RNPTGMQGFALNTRRPMFTDPRVRQALAYLFDFEWTNRNLFYNAYARSHSYFSNS
EMAA----VDLPGAAELRLLEPWR----EQLP------DEVFTQVYRAPETDGS-GAI----RDNMRIASSL
LRQAGWEIQG---------------------------------------------------------GRLTH
RSSG--STMDIEFLIFD---TIWERIIQPYRRNLERMGIESSIRVV-DVTQYVNRVR---SFDFDVVVAV-F
PQ----STSP-GNEQRDFWHSAFADQEGSRNLIGIKDPVVDALVENIIAASDREE-LVVSARALD-RVLQWG
HYVVPHWHIT---------SYRVAYWD-LFAKPSENPP--YDLA---FDTWWVDPEKSATI-----------
----------------
>Candidatus/1-567 Competibacter phosphatis
--------------------------------------HGIALHG---QPKYGPNFEHFDYVNPSAPKGGEA
RFAAIG--------SFDTFNPFNI-------KGQPAAGIGQ------------LFETLLIGSADE-------
--PFSEYGLIAESVEIPEDR----SSVTFNLRPQAKFHDGTSITADDVLFSFEILKTKG--TPLYRFYYANV
AKVEKLGERQVKFTFA-PGENRELPLIVG-QMPVLSEKYWR-------DRDFAATTLE-VPVGSGPYRIERF
EPGRFIVYQRDENYWGKDLAVNRGMHNIDRLRYDYYRDVTVALEAFK-AGSYDLRVEN--VAKQWATGY-DF
PALAKGLVKKETFSN--QMPSGMQGFVYNLRRPLFQNPKVREALAYAFDFEWSNRNLFHDQYKRTRSYFDNS
ELAA----RGLPSPEELAVLEPLR----KELPP------EVFSAVYEPPTANDD-AQL----RANFQKALQL
LQEAGWTFR---------------------------------------------------------DRKLVN
AKTG--EPFRFELLIAE---PTWERIALPFARNLERLGIEMSVRSV-DSAQYENRER---SFDFDMIVNV-W
GQ----SLSP-GNEQREFWSSAAAGQPGSRNIAGLKSPAVDALVDQVIAAPDRTS-LVTRVRALD-RALQWN
FLVIPHWHL---------PYARIAFWD-KFGYPSVT--PLQGVQLD---AWWID------------------
----------------
>Candidatus/1-584 Contendobacter odensis
-------------------------------------THGIALHG---QPKYGPEFSHFDYVNPNAPKGGEA
RFATIG--------SFDTFNPFNI-------KGQAAAGIGQ------------LFESLLTGSADE-------
--PFSEYGLIAESVEVAEDR----GSVIFVLRPQAKFHDGSPITVDDVLFSFETLKTKG--SPSFRFYYTNI
AKVEKLGERQVKFSFN-PGENRELPLIIG-QMPVLSKKYWQ-------GREFGATTLE-IPVGSGPYRLERF
EPGRFVVYQRDDQYWGKDLPVNRGRYNIDRLRYDYYRDVTVALEAFK-AGNYDLRMEN--VAKLWATGY-DF
PALSKGLVKKENFPH--QMPSGMQGFAYNLRRPLFQDAKVRQALAYAFDFEWSNRNLFFSQYTRTRSYFDNS
ELAA----RGLPSPEELAVLEPLR----KELPP------EVFSAAYEPPTANDD-AQL----RANFQKALQL
LQEAGWMFR---------------------------------------------------------DRKLVN
AKTG--EPFRFELLIDE---PTWERIALPFARNLERLGIEMTVRSV-DSAQYENRVR---DFDFDMIVNV-W
GQ----SLSP-GNEQREFWSSAAANEPGSRNLAGLKNPVVDRLVDQLIAAPDRVS-LVTRVRALD-RALQWN
YLVIPHWHL---------PYARIAFWD-KFGYPPVT--PMQGVQLE---AWWIDPAKDAALSQSGSGSGK--
----------------
>Arhodomonas/1-570 aquaeolei
--------------------------------------------G---SPKYPSGFEHFDYVNPDAPKGGTL
RLANTVAA------TFDSLNPFIL-------KGNPAADLTR------------TYDTLTVRSLDE-------
--PFTEYGLVAKDIEIAPDR----TWVRYRLRKAARFHDGEPITAEDVAFSFRILKEKG--HPQYRSYYKDV
TGVDVEDAHTVTFHFA-SGDNRELPLILG-QLPILPKHYWA-------ERDFDKTTLD-PPLGSGPYRIAEV
DPGKRIVYERVDDYWAKDLPVNRGRYNFERISYDYYRDATVAVEALK-GGAYDMRIEN--VAKNWATAY-NI
PALEEGRLVKQSIDH--NLPFGVQGWFFNTRRPVFQDPRVREAIGYAFDFQWTNRNLFYGAYKRLDSYFANS
ELAA----RGKPSGAELKLLEPWR----DQLPE------RVFSKAYTPPSTAGE-GGL----RDNLRRAVAL
LNDAGYAIRD---------------------------------------------------------GRMVN
EDTG--KPLSFEILLDS---ASMERVTLPFVKNLERLGIDATVRTV-DPTQYQNRVQ---RFEYDMIAER-I
AQ----SLSP-GNEQRAYWGCEAAKTPGSQNYAGICSPAIDALIDKVIHAPDREA-LVTRTRALD-RALLWG
FYVVPHWYS---------GSFRLVYWD-KFGQPARN--PPYGLALD---SWWVNPEKAAAL-----------
----------------
>Motiliproteus/1-573 coralliicola
```

-----------------------------VQADEPRPQHAIAMHGT---PKYSAGFDHFEYTNPEAPKGGTV
RRWALG--------TFDSFNPFII-------KGTPAAGVG------------QIYDSLMVKSQDE-------
--PFSQYGLLAESVVMPEDR----SWIRFKIRQEAQFSDGKPVTSDDVIYSFKLLREQG--NPFYRAYYADI
ETIEAIDDRTVEFRFKPSQNR-ELPLIVG-EVAILPKHYWQ-------GRDFSSPGLD-IPVGSGPYLIDSY
DPGRSLTYRLRTDYWGQDLPVNRGRHNYGQIRYDYYRDTTVALEAFK-AGEYDFRQET--SSKNWATSY-NG
PMFDNGEILRDEIRH--SNPTGMQAFVLNSRRPLFTDSRVRQALAYAFDFEWTNKNIFYNAYTRTHSYFSNS
EMAA----TELPDAKELKILEPIR----DQLP------AEVFNQVYRAPNTDGS-GKL----RQQLRQGLRL
LKQAGWQIKQ---GKLTN
AQ-G--QVFSFELLLVQ---KEFERVVAPFIRNLNKMGIDVRIRIV-DVSQYINRLR---SFDYDMMVYS-Y
GQ----SNSP-GNEQREFWHSAMADIQGSRNLMGIKNPAIDYLVDQVISAPSREQ-LVLRTRALD-RALQWN
HYLIPQYHIN---------SYRIAYWD-KFGMPDTRPL--YALG---FDTWW--------------------
----
>Hydrocarboniclastica/1-596 marina
-----------IARNLLAVLSAFFILVPPVHAAAPEPRHGIAMHGE---LKHGPDFEHFDYVNPDAPKGGEL
KLAVTGN-------GFDTFNPFVL-------RGVAAAGANA-----------YLYDTLLSQSEDE-------
--AFSAYGLIAEKVQVPEDR----SWVTFHINPQATFHDGEPITADDVIFSFETLTEKG--HPFYQAYYADV
AEVKKLDDLTVRFNFKDTTNR-ELPLILG-QLPVLPKHYWK-------DREFGGASLE-PPVGSGPYEIDSY
EAGRTVTYGRVDDYWAADLPVNRGRYNFDKIRYEYFSDDTVSMEAFK-AGIYDFREET--SAKSWATAY-EG
EKFTEGEFKKEEIHH--EQPAGMQGFIYNTRRSVFQDPLVRQALAYAFDFDWTNRNLFYDQYSRTNSYFENS
ELAS----TGLPSGAELKLLEPHR----KNLL------PAVFEQEYRPPSSDEE-GEM----RKNLARAVQL
LDQAGWGFKD---GKMVN
RKTG--EQLKFEILLAQ---KTFERVVSPFINNLKRLGIDADMRRV-DTTQYVQRIR---NFDFDMFIMS-I
GQ----SNSP-GNEQRSFWSSEAAEQPGSRNYAGVSDPVVDDLVDKVIQAPDREA-LVARTRALD-RVLLWG
HYVIPQWYLP---------YRRVAYTS-ELQRPETLP---ATG-VS-LDTWWYE------------------
----
>Thalassobaculum/1-575 fulvum
-------------------------------------SHGIAMHG---DLKYAPDFTHFEYVEPNAPKGGTL
VSEATG--------SFDSFNPFI-------LRGQPASGVA------------LIYDTLTEQAQDE-------
--PFSEYGALAESIEMPADR----SWVAFNLRPEARWHDGRPVTAGDVVWTFDTLVEKG--APFYRAYYGDV
VEVKATSEHRVLFVFKGPG-NRELPLILG-QLAVLPKHWWQG-------KDFANPPFE-PPLGSGPYRVKGY
EAGRTVTYERVADYWGKDVPAMRGRWNADTIRYEYFRDRDIATEAFK-AGAFDLRAEN--SSKRWATAY-SF
PGLDAGMAIKEEVPH--QNVAGMQGFVFNTRRPVFRDRTVREAIGYAFDFEWTNKALMYGAYTRTDSYFD-N
SELGS---SGLPTGAELALLEPFR----DRLPP------EVFTTEFTVPKTDGS-GNP----RQNLRTALKL
LREAGWAVKN---G-VLT
NAEG--QALSFEILLV--S-PDMERIVLPFAANLEKLGIKASVRTV-DPAQFQNRVR---DFDFDMIVGS-F
GQ----SQSP-GNEQRDYWSSAMADLKGSRNVIGVRDPVVDALVEKVIQAPDREA-LVAATRALD-RVLLWG
FYVVPHFHS---------ETYRLVYWN-KFGKPA--VAPKYGLG--YPDTWWIDPAKAAAV-----------
----
>Spiribacter/1-569 aquaticus
--G---EPRYGPGFEHFDYVNPQAPKGGTL
KLANVAAL------TFDSLHPFIL-------RGTAAEGMGR------------VFDTLTVQSLDE-------
--PFTQYGLIARSIEVADDH----SSVRFNLRDTARFHDGEPITADDVAWTFRRLREDG--HPRYRAYYADV
DRVEVIDPLTVEFHFA-TTDNAELPLILG-QLPVLPAHDWA-------ERDFTATTLT-PPLGSGPYRVASV
EQGRRIVYERVDDYWAADLPVNRGRYNFDRISYDYYRDGTVALQALK-AGEYHLRREN--VARNWATAY-DT
PAVESGLLKRVEIPH--DRPAGMQGFFFNTRRAVFADPAVREALAYAFDFEWTNENLFYGAYDRTRSYFQNS
ELAA----TGLPSEAELELLEPYR----DQLPE------RVFTEAYAPPDTT-D-GSR----RANLRRALAL
LRDAGWAVRD---GTLTH
TESG--QPMAFEILLVS---PSFERVVLPFKRNLERLGAEVSVRTV-DPTQYQNRME---AFDFDMTVHV-A
PQ----SLSP-GNEQRDFWSCEAAEITGSRNVAGVCDPVIDELVTAVIDAPDREA-LVTRTRALD-RVLQWH
FYAIPNWYI---------DRFRLAYWD-RFGRPADN--PPYGLALD---SWWVDPERAARV-----------
----
>Ferruginivarius/1-609 sediminum
-------MRCVV--LAFLIALVTGPALAQAVD-GVEPRHGVAMHG---DVKYGPDFRHFDYVNPDAPKGGTI
RLAAEG--------TYDSFNPYII-------KGVTAEGAGL------------VFETLMASSSDE-------
--AFSEYGLLAESVYVPEDR----SWVAFKLRENARWHDGTPVTVEDVIFSLNVLKEKG--HPFYRFYYKNV
AKAQRVGPRTVRFDFS-GGVNRELPLIVG-QLPVLPKHYWE-------DREFGQSTLQ-PPLGSGPYRVADY
EPGRYVVYERVEDYWGKDIPVNRGRWNYDTIRYDYFRDPTVIREAVK-SGTVDLRLEN--QAKAWATAY-EG
PAVRSGELTLEEIDH--DQPTGMQAFAMNTRRPPFDSPKVREAMAYAFDFTWTNRNLFFGQYSRTESYFSNS

```
ELAS----SGVPTGRELDILERFR----AQLPP------EVFTQEYDPPETNGD-GYP----RENLKAALSL
LNEAGWVVR---------------------------------------------------------DMKLVR
ERTG--RPFSFEILLVN---KAFERVVLPYVANLEKLGMEVEVRVV-DSAQYQNRLD---SFDFDMMIAG-W
GQ----SLSP-GNEQRDFWSSQAAERNGSRNYTGLADPVVDQLIDMIIQAPSREE-LVLRTRALD-RVLLWH
HLVVPNWHI---------GYFRVAYWD-KFGRPELNP-PYGLPYLD---AWWVERDKAAAVKTAQSE-----
----------------
>Agarivorans/1-583 gilvus
---------------------CLLLLVNLAHAEQFVRSHALVMHGA---PALAADFAHLPYVNPNAPKQGSI
THEAQG--------TFDTFNPFIV-------KGNPAAGVG------------QIYDTLLSANQDE-------
--AFALYGLLAETIDLPADR----SWVRFNLRPQARFHDGEPVTADDVVFTFNLLMEQG--APFYRAYYGSV
KSVKAESKLSVRFDFVDGSNR-ELPLILG-QLPILPKHFWQ-------QHDFAKADLT-LPLGSGPYQISEY
DTGRSVSYSLVKDYWGKDLAINRGRYNIATINYEYYRDSSVALEAFK-AGRIDYRLET--SAKDWATAY-TG
PMFDSGKVHTDVLKN--ENPQGMQGFVFNTRREMFKSRDIRKAIGLLFDFEWTNEQLFYGAYKRTESYFAAS
ELAA----TGLPEGAELALLSPFK----EQLP------KELFTTPFSLDKTRGD-GNV----RPQMREALAL
LKGAGWQLDK---------------------------------------------------------GKLVN
AQ-G--QQMHIEFLLYQ---KSFERIVLPYTRNLQKIGISTEIRLV-DVSQYVNRVQ---SFDFDMVVGS-F
GQ----SSSP-GNEQRDFWGSKAADHVGSRNIIGIKDPVIDQLIEAVIAADDRDA-LIAATRALD-RVLLWS
YYVVPQWHLN---------AWRVAYWD-KLKRPARNPK--YGIG---LDSWWVE------------------
----------------
>Desulfurispira/1-594 natronophila
----------------IMLLPFALFFPLILSANDS--LHAIAMHGE---PKYGPDFSHFSYVNPEAPKGGDV
RLHALG--------SFDSLNPFIS-------RGNAASHLE------------LIYDSLLERSLDE-------
--PFTEYGLIADSISIAEDR----SWVEFTIHPDARFHDGTAIGPNDVVFTFDILRSQG--APFYQAYYENI
EKVEALDDKTVRFQFSESGNL-ELPLIVG-QAPILPSHFWE-------NHDFTRSSQL-KPLGSGPYKIVNV
DGNRSITYRRVEDYWARNLPVNRGRYNFDTITIDYYRDASVALESFK-AGSYDFRLEN--NAKDWATSY-NI
PAVANGHIQRQSIPH--RNPTGMQAFAFNTRLDKFSDPKVRLALNYLFDFEWLNRNMFYGAYSRTQSYFSNS
ELAA----RELPSQREREILEPFR----EKLP------PEIFTEVFHNPVTDGS-GNI----RGQIRTAARL
LQEAGWEIKR---------------------------------------------------------GKLTH
NKTG--EVLAFEFLLYD---SMFERVVQPFRRNLERMGIETSIRVV-DITQYINRQR---NFDFNVVVGL-F
GQ----STSP-GNEQRNYWHSDFAQREGSRNIIGISNPVVDELVEKVISAPNREE-LVHRTRALD-RVLLWN
HYVIPHWHIT---------NYRVAYWN-IFGRPETPPE--QGIG---FDTWWVDSDKASQL-----------
----------------
>Azospirillum/1-581 humicireducens
-------------------------------------SHALSLHG---KPKYGPDFQHLDYVNPDAPKGGEV
KLMAFG--------GFDNLNPFIL-------RGQPAAG-AGL-----------VFQTLTTSNGDE-------
--AITEYGLLAESIEVAPDR----TWVAFTLRPEARFHDGKPVTADDVIWSFDTLREKG--HPLYRTYYADV
TKAEKTGERTVRFSFR-NGNNPELPVIMG-QLPVLPKHFFA-------NRDFATTTLE-PLLGSGPYKVADV
QAGRAITYERVQDWWAKDLPINRGRYNFDRIRYDYYRDLDVAFEAFK-AGAYDFRLEN--SSKNWTTGY-NV
PAVQNGQIVKEELKH--EDPQGMQAFVFNIRRPIFEDRRVREALNYLFDYEWTRANLSYGLYQRTKSYFANT
ELAA----TGLPSPAELKLLEPFR----GQIPD------EVFTKPYEPPKTDAS-GNI----RGNLRTALGL
LKEAGWELK---------------------------------------------------------NGKLVN
AQG---QPFRFEILLVQ---AEMERIVQPFVRNLERAGIEAQIRVV-DSAQYQNRTD---NFDYDMIIER-F
PQ----SLSP-GNEQRDYWESSRADQPGSRNSIGIKNPAVDKLVETLIAAPDREA-LITATRALD-RVLLWN
WYVIPHWHD---------TVYRVAYWN-RFSHPAVA--PKYGLGFS--DSWWVDADKNAKLANSARR-----
----------------
>Beggiatoa/1-604 leptomitoformis
--------MRAFFLFTLLLVSNAPTFATEQNNGNVTVGHGMSMYG---DLKYPPNFKHFEYANPDAPKGGTV
RLADIG--------TYDSFNPFII-------KGIPPAGIGG------------LFETLTVNSDDE-------
--AFSVYGLIAETIEVPEDR----TWVAYQLRSIARFHDGTPITPEDVIFSFEILKEKG--NPFYRSYYENV
TKVEKVDEHKVKFTFI-NGTNKELPLIMG-QLPILSKAYWS-------TREFDKTTLE-APLGSGAYKIDTF
EAGRFVVYKRVPDYWGANLPVNLGQENFDTIRYDYYRDSTVALQAFK-AGEYDFRSES--TAKDWATAY-DF
PAVKTGLVIKEEITH--EIPTGMQAFFFNTRRPLFADPKVREAIGYLFDFEWSNKNLFYNAYTRTDSFFSNS
ELAAH---N-LPSAEEQAILEPFK----NKIPE------HIFTTAYQPPSSDGS-GNI----RDNIRKALGL
LTDAGWEFK---------------------------------------------------------QNRLVN
KTTG--KPFDFEILLDS---PAFERISLPFAKNLERVGIKATIRTV-DSSQYKNRLD---DFDFDMIVAV-V
GQ----SLSP-GNEQRDYWGSNKADIKGTRNYAGIKDPVIDALIEQVIAAPDRAS-LIAHTRALD-RVLLSG
YYVIPHWHI---------RTFRVAYWN-KFMHPNIA--PKYGLGFN---TWWIDKEKAQQL-----------
----------------
```

```
>Alkalilimnicola/1-600 ehrlichii
------------MPQRIVRTFVMALGLIGLPAAALAGGHAIALHG---EPKYGPDFEHFDYVNPDAPKGGAV
RLSALG--------TFDSLHPYIL-------RGVPAQGLSQ------------VFDSLTENSADE-------
--PFTEYGLIAETIEVDPEG----YWVRFDLRPEARFHDGEPITVDDVIWTFETLREHG--HPSLRSYYRDV
ERVERTGERQVTFHFA-GNENAELPLIVG-QMPVLPEHWWA-------DREFDRTTLD-KPLGSGPYRVAEV
RQGRHIVYERVEDYWAADLPVNRGRHNFDRIRYDYYRDADVALEAFR-AGEYDFRPEN--IARNWANAY-DF
AAVREGRVQREEIAH--EIPTGMQGFFINTRRDRFSDPRVREALSLAFDFEWTNRNLFHDGYTRTRSYFSNS
ELAS----DGPPSAEELEILEPYR----DQLPE------ALFESAFEPPSTEGD-RGL----RRNLRQAAAL
LREAGWVVED---------------------------------------------------------GRLVH
GETG--ERMRFEVLLDN---ASFERVALPWRRNLERLGMEVSVRTV-DTSQYQSRMD---EFDFDITVQL-I
GQ----SLSP-GNEQRNYWSCAAAETPGSRNYAGICDEVVDALIERIIHAPDRDT-LVAATRALD-RVLLHG
HYVVPHWHL---------PAFRLAYWD-KFDRPETS--PKYALGFD---TWWYDEERAAEL-----------
----------------
>Thiopseudomonas/1-581 alkaliphila
-------------------GILSFALVQS---AVAEVKHALTLYGE--PPKYSANFTHFDFVNPKAPKGGQL
KLASFG--------GFDSLNPYLA-------KGNPADRVS------------LIYDSLMYQSPDE-------
--PFTVYGLIAEGVELDENN----QFVRFILNPKARFQDGTPITSADVAFTFNTLIEKA--HPLYRHYYADV
AEVVIEDPLRIRFDFKHNKNR-ELPLILG-QLEILPKHWWV-------SRDFSKTSLE-PPLGSGPYRISKV
DPGRSIEYQRDENWWAKDLPVSRGLYNFDQIQVDYYRDMSVALEAFK-AGQFDLNQEY--SAKDWATGY-DS
PALRSGKIIKESFAN--HNPVGMQGFTFNLRRPLFQDIRVREAISLLFDFEWTNKQLFYGSYLRTNSFFENS
ELAA----EGLPSAAELEILTPLK----EQLP------ESVFTQVFKNPVSDGS-GII----RDQQRKAFKL
LQDAGYRVEN---------------------------------------------------------DRMVN
AD-G--EPLSFEFLITQ---TNLERVLLPFKRNLEELGIEMIIRRA-DMSQYINRIR---SRDFDMTSSI-W
PQ----SNSP-GNEQREFWHSSSKDNPGSRNYIGLADPAIDQLVEGLINADSREH-LITHTRALD-RALLWG
HYVVPNYYIN---------TWRVAYWD-KFAHPEVTPL--YDFG---LMTWW--------------------
----------------
>Desulfobotulus/1-603 alkaliphilus
----------------ICMLLIQGLYFMTP--ALAAPTHGIALWG---EPQYGPDFTHFAYARPDAPKGGHL
RMAATG--------SFDSFHPFTI-------RGTAAAGVG------------LLYETLMTASLD--------
-EPSSSYGLIAQSIMVSEDR----TSVTFRLRPEARFHDGQPVRAEDVVFSFQILVREG--SPHYRKYYEDI
TGAHALDDHTVRFDIR-PGSAMETPYITG-QLPVLPAHVWK-------EREFSRSSLE-TPVGSGPYRIHSF
DTGRRITYVRDPDYWGWHLPVNQGHFNFDFISWEYFRDNTVSLEAFK-AGVFDYRLEN--TAKTWATLY-DG
PAFRDGRIRKETIPH--ERPQGMQGFVFNIRKPVFRDRAVRKALSLIFDFEWANTHLFYGQYNRSHSFFQNS
PMAA----EGLPSEEELVLLEPFR----HLLPE------SAFGPAIVPPVTDGS-GNI----RPLLREADRI
LNEAGWILKDGK---------------------------------------------------------RIH
KETG--RPLSFEILLAS---PSFQRIAMPFRQNLARLGVDASVRVV-DRTRYVRQLQ---DFNYDMIVGN-F
RQ----SHSP-GSEQRDFWGSAAASVPGGRNTIGIADPVVDALVEHILNAENREE-LITACRALD-RVLRAN
HYVIPQWFN---------ASYRIAYWD-KLRHPEKSPEHGLGIY-----TWWIDTKAEARLTPGAVMHTE--
----------------
>Pseudomonas/1-561 cavernae
-------------------------------------------------PKYPANYQHFDYVNPDAPKGGTL
RLSGNG--------GFDSLNPFIP-------KGNAESRIG------------MVYESLTYHSPEE-------
--PFTEYGLLAEKIEKAADN----SYVRFYLHPKARFHDGAPVTAEDVQFTFETLMKNG--DPMYRSYYADV
DKVVVESPLKVRFDFKHMGNR-ELPLILG-QLQILPKHWWA-------TRDFAKTSLE-PPLGSGAYRVASL
ESGRSIRYERVKDWWGEDLPVNRGQYNFDAIVVDYYRDMAVALEAFK-GGQFDLNLEY--SAKDWATGY-QS
AALSDGRMLKEAITN--HNPVGMQAFAYNIRRPVFQDRRVREAIGLLFDFEWSNKQLFFGSYKRTTSYFENS
EMAA----SVLPDAAELKILEPLR----DKLP------PEVFTQVYLPPVSKGD-GII----REQKRRAYQL
LQEAGYHIDN---------------------------------------------------------DRMVG
PD-G--KPLSFEFMLFQ---TNMERILLPYKRNLSELGIDMQIRRV-DASQFINRLR---SRDFDMTSAT-W
GQ----SNSP-GNEQREYWHSSSADNPGSRNFIGLRDPAIDQLVDGLIRADSRQS-LIEHARALD-RALQWG
YYVVPNYYVD---------TWRIAYWN-KFGRPEKTPL--YDYG---LMTWWQKSDK---------------
----------------
>Limibacillus/1-579 halophilus
---------------------------------AVHKSNAIAMHG---EPKYGPDFKHFDYVNPEAPKGGSL
RLADVG--------AFDSFNNFII-------KGEAAAGIGG------------IYDSLMESSADE-------
--AFTEYCLLCESVEWPDDR----SWVAFTLRDDAYWHDGKPITVEDVIFSLDLLRTQG--HPFYRFYYGSV
ASTEITGPRTVRFTFS-ETGNRELPLIIG-QLSILPKHYWE-------ARDFSTTTLE-PPLGSGPYKVESF
EPGRFVTYKRVENYWARDLPVQKGRNNWDEIRYDYYRDDTIVREALK-GGDIDLRVEN--QAKAWAVDY-DV
```

QAVRDGKVILAEIPN--ERPTGMQSFAMNTRRAPFDNRLVRRALAFAFDFEWTNRNLFFSQYTRTESYFSNS
ELAS----RGLPTGAELEILESYR----DSLPP------EVFTETYSVPSTDGS-GWP----RDNLRQAFAL
LEEAGWIVR---DMKMVN
AETG--EQMGFEILLSS---PAFERIVLPYIRNLRRLGIDARVRLV-DSSQYVNRIR---SFEFDMIVGS-W
GQ----SDSP-GNEQRNFWGCQAAESPGSRNLTGVCDPVVDSLIEQLIAAPDRES-LVATTRALD-RVLLWG
HYVVPNWHI---------RIDRIAYWD-IFGQPEIV--PKNGAQID---SWWIDRAKADKL-----------
----
>Oceanibaculum/1-586 indicum
--------------------------TVPATAQEVTTSHALILHG---TPKYPADFKHFEYVNPDAPKGGEV
IQAAIG--------TFDSLNPFIL-------RGVSAAA-LGL-----------TFDTLLQSSSDE-------
--AFTEYGLLAESIELPADR----KWVAFTLRPEARFHDGKPVTAEDVVWSFETLKAKG--APFYRAYYADV
LKAEALGERKVRFSFG-DTVNMELPLIIG-QMPVLPKHYFA-------DKEFDRTSLE-PITGSGPYKVASF
EPGRTITYERVADYWAKDLPIMRGRYNYDRIRYDYYRDATVAIEAFK-AGAYDFRLEN--SAKEWATAY-DF
PAQRQGLAVQREIPH--ELPTGMQGFAMNTRRPIFSDPRVRLAMNYAFDFEWSNKALFYGAYTRTESYFSNS
ELAS----RGLPEGEELKILEQFR----GKIPD------EVFTTEYKAPTTDGS-GNN----RANLRKALEL
LQSAGWQVK---GGKLVN
AAG---EPFTFEILLSS---PAFERISLPYAKNLERLGIEASVRTV-DTAQYQNRID---SFDFDMTVDV-W
GQ----SLSP-GNEQRDFWGSSRRDEQGSRNTAGIADPVIDQLIDMVIQAPDRDS-LIARTRALD-RVLLWG
HYVVPHWHI---------RAFRTVYWD-KFAQPEIT--PKYALGFS--DTWWIDPAKAEKV-----------
----
>Pelagibius/1-590 marinus
----------------------------GAQDQIIHKSHAIAMHG---EPKYPADFQHFDYVNPDAPKGGTL
RFGASG--------TFDSFNPYII-------KGNTAAGLGF------------LYETLLTSSADE-------
--AFSEYGLLADSVEWPDDR----SWVIFHLRPEARWHDGEPITAEDVIFSLNVLREKG--HPFYRFYYQNI
EKATKLGERSVRFDFS-EGENRELPLIAG-QIPVLPKHYWE-------DREFDKTTLE-PPLGSGPYKIAEF
EPGRYIDWQRVDDYWGKDLPVNRGLYNFDRLHYQYYLDDTVIRQALK-AGDLDYRVEN--QAKAWAVDY-DV
PAVERGLLIKRGFEN--ERTSGMQAFAMNIRRDQFKDPRVREALSYAFDFEWTNKNLFFSQYTRTESYFANS
ELAS----SGLPEGEELEILERFR----GRVPE------EVFTTKYHAPKTDGS-GWP----RDNLAKAFEL
LAEAGWVVR---DLKLVN
AETG--VQLRFEILIVS---PAFERIVLPYTRNLERLGIDARVRLV-DQSQYINRLR---AFDFDVIVSS-W
GQ----SESP-GNEQRDFWGSESANSPSGRNYVGIADPVIDELIELLIAAPSRES-LVARTRALD-RVLLWH
HFVVPNWHS---------RVDRILYWD-KFSQPEII--PKSGTSWA---YWWWNEEKAKLLEQRRAQ-----
----
>Amphritea/1-593 opalescens
-----------------CLLSATLIGSLAAPLQAATPQHAISMYGD---LKYPADFKHFDYVNPDAPKGGSV
SQDAIG--------TFDSFNPFII-------KGAAADGIG------------LIYDSLLTRSQDE-------
--AFSLYGLLAESLEVADDR----SWIIFNLNPKARFSDGQPVTAEDVIYSFKLLREQG--SPFYQSYYREI
SSIEALSERRVKFSFSESENR-ELPLIVG-EVSILPKHYWQ-------QRDFSKPSLD-VPIGSGPYVVDSF
DAGRTINYKRNPDYWGADLPVNRGRFNFANIRYDYYKDGNVALEAFK-GGEYDFRQET--SSKEWATGY-KG
SVFDDGKIITRNITH--ENPTGMQAFILNTRKPYFADSRVRQALAYAFDFEWTNKNIFYNAYTRTHSFFSNS
EMAA----TELPTAEELKILEPVR----DQVP------PEVFTQVYKAPTTKGD-GKT----RGQLRSALRL
LKSAGWALKD---GLLLG
PD-G--KQMTFEILLVQ---PAFERIVAPFSRNLERMGIKPTVRVI-DASQYINRVR---NFDFDVIVSG-F
GQ----SNSP-GNEQREYWHSSTADQPGSRNMIGIKNPAVDYLVEQLIQAPNREQ-LVLRTRALD-RVLQWN
HYVIPQYHIN---------SYRVAYWD-KFAFPAIHPK--YSLG---FDTWWVKPNQAEQ------------
----
>Roseospira/1-579 marina
-------------------------------------SHAIAMHG---APKYGPEFTHFDYVNPEAPKGGTL
KLAWVG--------SYDSLNPFII-------KGRPAIGLGL------------VYDTLMVASSDE-------
--PFSHYGLVAESVETPNDR----SWAIFRLNPKARFHDGHPITAEDVLFSFDILRTEG--APLYRFYYADV
SEAVALDDHTVKFTFS-TNRNRELPLVLG-QLPVLPKHAWE-------DRTFSDTTLE-PPVGSGPYRVAAF
EPGRYIRYERVKDYWGADLPVMVGMYNFDAIQYDYYRDFTVAVEALK-AGEYDFRSEN--IAKNWATAY-EG
ENKNKGLLIQHAFKH--SRVAPTQGYVMNMRRPPFNDPAVREALAYAFDFAWANQNLFYDAYARTRSNFDNS
ELAA----TGVPEGDELALLEPYR----DQLPP------RLFTEEYNPPSVPAP-GEI----RQNLRTALTI
LNKAGWEMK---DGVMTQ
TERG--DTLRFQILLDN---PSLERVTLPYVQNLKRLGADVDVRVV-DTAQYTNRIN---SFDFDMIAEI-W
AQ----SDSP-GNEQREFWSRDTANVEGSRNLTGVSSPVVDALIDDIVRADSRED-LVTAVHALD-RVLQWT
FYLVPMYHS---------EADRFVYWN-RFGMPEIT--PDSGASVM---TWWIDPEKDAALRAAG-------

----
>Neptunomonas/1-584 phycophila
-------------------LIVVAGLVCYSSFTLAAPSHGIAMHGD---LKYSSDFTHFDYTNPDAPKGGSV
KQWALG--------TFDSFNSFII-------KGTPADGLG------------LIYDTLTSQSTDE-------
--PFSEYGLIAKTIDVADDN----SSVTFVLNKNAKFSDGEPVTANDVAFTFKTLTTEG--SPVYRIYYGDV
KDVVIVDEHTIRFEFKSTKNA-ELPLIIG-QLPVLPEHYWK-------DKDFNAPTLD-KPVGSGPYMLDSY
EAGRSITYKRNPNYWGADIPVNKGRYNFDFITYDYYRDTTVSLEAFK-AGEYDFRQET--SSKNWATSY-VG
PMFDNGQIIKAEIPH--SRPTGMQAFVFNTRRDIFSNPDVRKALSYAFDFEWTNKNLFYSAYARTNSYFSNS
EMAA----EQPITPQETALLEPFK----DQLP------SEVFTDVFKAPVYDGS-GNN----RRELRTALRL
LKSAGWELKN---GKLLN
KETG--QAFTFEILLVQ---KEFERIVAPMLKNLKQMGIEANIRIV-DVSQYINRLR---DFDFDMVVSS-F
GQ----SSSP-GNEQREFWGSYNADMKGSRNIIGIKNPVIDALIEKIIEAPSRQE-LVYRTRALD-RVLQWN
YYVIPQFHST---------SYRIAYKP-FFGMPKTRPL--YDIG---FDTWW--------------------
----
>Pseudomaricurvus/1-591 alkylphenolicus
--------------LLLAGLWTVTS-QLALAESTIIKTHAIAMHGD---PKYGSDFKHFEYVEPSAPKGGTL
RLHSIG--------TYDSLNPYIA-------KGTPATGLG------------LIYDTLMTSSADE-------
--PFTEYGLLATKVEYPEDR----SWVIFHLNPEARFHDGMPVTAEDAAFTFDLLIKEG--RPLYAFYYADI
TSAEALDKHRVKFNFKSN-ASRETLLITG-QLPVLPKHYWK-------DRKFSESSLE-IPVGSGPYKIARI
DAGRSIVYERVKDYWGGDHPVQKGHYNFDKMVYDYYRDDVVALEAFK-ADEYDFRRER--ISKLWASAY-DG
DALQSGRIQKEQIPH--QNPTGMQCYLFNLRKPIFQDETLRRAIVLAFDFEWANKNLFHNAYTRTESYFSNS
ELAS----SGLPSAAELKLLEPHR----DKLP------PELFTKAFKAPRSTGD-DRN----RKNLRAAKKL
LEQAGYKLGG---GQLKS
PQG---QDIKFDILMTD---ASSERILNPFIQSLKKLGIMVKLRRV-DPSQFIERRR---NFDYDMISHV-Y
SQ----SLSP-GNEQRDFWHSASVDSIGSRNLVGISDPVVDDLVAKVIEAHSREE-LVVATRALD-RVLLNR
HFVIPAWHIS---------SHRIAYWN-RYERPAISPIYDLVYQTG-LMTWW--------------------
----
>Motilimonas/1-583 cestriensis
----------------LGLAASFSSFATTSDLHP---QHGIAMHGD---LKYSAGFSHFDYVNPEAPKGGAM
RQAARG--------TFDSFNPFIV-------KGTPANGLA------------LIYDTLMVRSDDE-------
--PFSKYGLIAAGVKVAEDF----SSTTFYLNPKATFHDGHPITSEDVIFSFNTLIEQG--NPQYKSYYAGV
DNVVEIDPHTIRFDFKPGDNR-ELPLILG-ELQILPKHYWQ-------QHNFSRSSLE-VPLGSGPYKIASF
DAGRKVVYQRVKNYWAADLNVNKGRHNFDRLVFDYFRDTTIAFQAFK-AGVFDYHQEY--SAKNWATAY-TG
EVFDSKKIIRRLLDD--ANPQGMQGFFFNLRRDKFKSREVRQAIGLMFDFEWLNRQFFYGAYTRSDSFFAHS
ELAA----TGLPSPDELAILEPYR----EQLP------KDVFTQVYQPPATQGD-GNI----RPQMRQAVAL
MEQAGYQLKN---GKMLD
PQ-G--NQLFFEFLVYD---KSFERVIQPYRRNLSRIGIATEIRLV-DISQYINRLN---SFDFDITTMR-Q
GQ----SISP-GNEQPIYWSCDAASTPGSRNYAGLCNPAVDELVQQLIRSNTREE-LVLHTRVLD-RVLQHL
HFVVPQWYSP---------SHRIAYWD-KFSQPKIKPA--YAIG---LDTWW--------------------
----
>Tamilnaduibacter/1-589 salinus
------------------LVLSVLSTLAVHAAEPVEPVHALAMHGE---PKYGPGFEHFGYVNPEAPKGGTL
RRSVVAN-------GFDSFHPYAL-------KGVAAAGIQQ-----------YAYDSLLVSSDDE-------
--PFTMYGLIAESVETPEDR----SYVIFNLRPEARFSDGEPITAEDVVFSYQTLVTDG--HPFYRNYYANV
GQATALGKHRVRFDFGEDTNR-ELPLILG-QMPIFPKHFWQ-------EHDFTGPGLT-VPVTSGPYTIDEF
EAGRSVVFQRQEDYWAEDLPVNVGRHNFDHIRFDYYTDSTVALEAFK-SGNFDLRIEN--SAKNWATGY-QS
DALENGRIIKETLEH--QRPAGMQAFVMNTRRAPFDNKHVRKALAYGFDFAWTNKNLFYGQYKRTDSYFENS
ELAS----SGLPSDAELKLLEPHR----DALP------DAVFNKAYQPPSTQGD-NTL----RDNLRQALKL
LNDAGYAIED---GTMTH
QQTG--EPLAFQILLRQ---KTFERVVLPFARNLEKLGIQVDVRLV-DTNQYVQRVR---QFNYDMIVQT-L
PQ----SRSP-GNEQRDYWHSVNADVSGSKNYMGIQDPVVDDLVSKIIQAPDREA-LVTRTRALD-RVLLWG
HYVIPQWFLD---------VDRVAYWQ-KLSRPDTIP---ESG-TD-LSTWWSD-------------------
----
>Novispirillum/1-574 itersonii
-----------------------------VPGSAVTVSHGIAMHG---ALKYPAGFHHFDYVNPAAPKGGTL
KMYSLG--------GFDSFNAFVV-------KGTPAPGVGM------------LFDTLLVSAADE-------
--AFSEYGLLAETIEAPENR----AWVAFTLRKQARFHDGTPVTAEDVVFSFNTLREKG--DPFYRLYYANV
TKVEAEGPLKVRFTFD-GADNRELPLILG-QMPVLSKAYWK-------TRDFSASTLD-APVGSGPYRVKAF

EAGRYVVYERVKDWWAKDLAVVKGQYNFDEIRYDVFRDETVALEAFK-AGAYDLRTEG--VAKQWATAY-EF
PARAEGKVRLKLFEN--HMPSGMQGFVFNQRRPVFEDPRVREALGYAFDFEWANQNLFYGQYTRTRSYFDNS
ELAA----TGLPSPEELKLLAPWR----GKIPE------EVFTKIYMPPTTEGT-GSQ----RANLRYALDL
LQQAGWDVK---NGVLTN
AKTG--KAMSFEILLGS---PTFERVVLPYVQNLKRLGITATVRTV-DAAQYQNRVK---SYDFDMVVMV-W
GQ----SMSP-GNEQREFWGSAAADQPGSRNIAGLKNPAVDALIDAVVKAQTREE-LITATRALD-RVLQWS
YLVVPHWHF---------PATRLAWWD-RFGMPDVV--PDKGVDLM---TWW--------------------
----
>Ketobacter/1-591 alkanivorans
-------------FIKLFLTACVAITTVTALASEVITSHGIAMHGD---MKYPSDYTHFEYTNPDAPKGGSV
VLAAYG--------TFDSFNPDVI-------KGDPAAYME------------LTYDTLMVQAMDE-------
--PFTVYGHVAEKVEYPEDR----SWIIFHINPKAKFHDGQPITAEDVVFTFKRLMEDG--QPFYRAYYADV
VNVEALDSQRVKFTARDDKNR-ELPLVLG-QLKVLPKHYWE-------PRDFSQASLD-IPLGSGPYRIKSF
DTGKSIELERVKDYWAADLPSHRGMYNFDNIKVEYYRDQTVMLEAFK-GGRYDYREEK--QSKRWATEY-EG
PKFASGDIIKLEVTH--QNPTGMQAFVMNTRRDLFKDARVREALDLAFDFEWTNKQLFYGAYNRTESYFSNS
DLAS----TGLPSEGELKLLEPFR----DDLP------AEVFTKPYVSPKTNGS-GNN----RKNIRAAINL
LKEAGWRFEG---TDLVN
AETG--KPFQFEILLYS---KDFERIVLPYVKNLKKLGIEANARIV-DSTNYIRIVR---DFEYDMIIGG-F
GQ----SNSP-GNEQRDYWFSKFADHKGSRNYIGVQDPVVDVLIDAIIRSQTRAD-LIAACRALD-RVLLWN
HYVVPQWHIN---------KHRIAYWD-KFEHPEITP---IYGDVG-FFSWW--------------------
----
>Elioraea/1-581 rosea
-------------------------------------THALSLLG---EPRYPPDFTHFSYVNPNAPKGGEV
TLAAIG--------SYDSFNPFIV-------RGVAAAG-IGG-----------VWMTLLKSSQDE-------
--ASTEYANLAEWVEIARDG----TWVAFEINPKAVWHDGRPITADDVVWTFDTLRQNG--RPFYRAYWGDV
TEAVAEGQRRAVFRFR-DGANRELPLILG-QLMILPKHWWE-------GRDFTRPLLE-PPLGSGAYRVDRF
EAGRTVTYRRVADWWAKDLPSERGTDNFDVMRYEYFRDATVAFEAFK-AGQIDFRAEN--VAKEWATGY-DF
PARRRGLVKLEEIRH--ELPTGMQAFAMNIRRPLFKDRRVREALMLAFDFEWSNRNLFFDAYARTSSYFSNS
ELAS----SGLPQGEELALLERFR----GKVPE------EVFTTEFKLPVTDGS-GNN----RDNLRRALTL
LQQAGWTVR---NQRLVN
ARG---EPFSFEMLLSS---PSFERVALPYAQQLQRLGIEMRTRTV-DTAQYQARTD---SFDFDMTVDV-F
GQ----SLSP-GNEQRDYWTCEKAKLEGSRNTIGVCDPVIDELVELVINAPDREQ-LITRTRALD-RVLLWG
HYVVPHWHS---------RVFRVAFWD-KFDRPATP--PRYGLGF---DTWWVDTAREARLAEARRSG----
----
>Halopseudomonas/1-593 aestusnigri
-------------------LALLT--CWAG-ALNAAPQHALTLYDE--APRYPADFSHFEYVNPDAPKGGTL
RLSGFG--------GFDSLNPYIS-------RGTSADQLG------------LIYDTLTFHALDE-------
--PFTEYGLVAESMEKADDG----SWVRFTLRPEARFHDGVAITADDVVFTFNTLIEHG--APFYRAYYGDV
DRVVADNPQSVTFHFKHTGNR-ELPLVLG-QLPVLPKHYWQ-------GRDFSKGSLE-PPLGSGPYRIGQV
RPGRSISFERVEDYWAKDLPVMRGFYNFDTVVVDYYRDGNVTLEAFK-AGQFDFNQEM--AAKNWATGY-AS
PALDAGQIVKEEIPN--NNTQGMQGFVFNLRKPYFQDARVRQAITLLFDFEWSNATLFHNAYTRTSSYFDNS
ELAA----RGEPDASELALLEPLR----DQLP------AAVFGPAWVPPKTDGS-GAI----REQRRTAYAL
LKDAGWQIVD---DQLVN
AD-G--EPLQFEFLLVQ---PEFERVLLPFKRNLASLGITMELRRV-DVSQYINRLR---SREFDMVVTG-F
GQ----SNSP-GNEQREYWHSSSADNPGSRNLMGLKDPAVDALVEGLVQADSRES-LITHTHALD-RALRSL
HLLVPNWYTS---------VYRVAYWN-KFGHPATPPK--YDLG---LFTWWVDPDKEARLRGA--------
----
>Fodinicurvata/1-612 sediminis
--------ILKAAGLCFLLWSFPVFAQEDQESEVPQPRHGIAMHG---EPKYPADFTHFDYVNPAAPKGGSV
RLAARG--------TYDSFNPFIV-------RGVAASGASY------------LNETLMVSSADE-------
--PFSMYGLVAESIRVPEDR----SWAEFTLREEANWHDGEPITVEDVIFSLNILREEG--HPIYRQYYADV
TDAEKTGPRKVRFTFS-EGENLELPLIIG-QLPILPKHYWE-------ERDFTRSDLE-APLGSGPYRVADF
EPGRHVAYERVEDYWGKDLPVNKGRYNFDRIRYDYFRDETVIRQAVK-AGEVDFRQEN--QAKAWALDY-DT
PAVEQGRLVMEEIPH--ERPTGMQGFVMNTREPVFSDPRVRRAMAYAFDFEWSNRNLFFGLYDRTESYFSNS
ELAS----SGLPEGRELELLEPYR----GQVPE------EVFEEEYFAPSTDGS-GWP----RQNLKMAFEL
LEEAGWVVR---DMELVH
KETG--EQMTFEIMIYD---TAFERVVLPYVRNLRRLGIDPTVRLV-DTSQYVNRLR---DFNFDMTITS-W
GQ----SDSP-GNEQRNYWSSAAANMTGSRNLAGIQDPVVDALIDEVIRAGSRED-LVAATRALD-RVLLWG

HYVVPHWHS---------RVDRLVYWN-KFGHPENP--PLHGFEFD---NWWIDEDRARRLREGDEMVE---
----
>Endozoicomonas/1-586 gorgoniicola
--------------------LSTPLFTPLRADDSVTRSHALSVYDQ--F-KHPEHFTHFDYANPQAPKGGTL
RKAATG--------SFDTFNTFAP-------KGNWAAE-SYI-----------LYDTLMVRAGDE-------
--PYTVYGLIAQSIEYPKDL----TWVAYNIHPEARFHDNAPITAEDVVYTFKALKEKG-PPHYRH-LYADV
TSVKATSRLRVEFRFSRP--QGKHLLLRLSQLRVLPKHFWTRP-----KHDIGNADLT-VPLASGPFKIKDF
EAGRHVTYERVKHYWAKDLPVNKGRHNFDEIRVDYYRDGQIALEAFK-QGAYDLRVDG--NPKNWAEGYK-G
NRLKNGDIIQEAVPN--KTLG-MRAFVFNLRKARFADRRLRQAISLALDFQWINKHLFFDIYKQAYSLFSNS
ELAAD----TLPSAKEIELLSPWR----KDLPE------EVFT-RVYQPPTTDGSGNW----RNNQRQALKL
LKEAGWQLRN---GKLVH
QKTG--EPMEFEILLSQPE-FE--RFVLPFANNLKALGIHARVSTI-DTSQYINRLR---EFDFDMVI----
-HGFYPGISPS-TELKSFWGSESARARSGKNISGISLPVLDALIEKSVKTQSRSE-LVDIARALD-RVVLWH
HAVIPQWYLP---------YWPFIYKK-GLKHPKTAPDYA-----SGLDTWWWE------------------
----
>Alkalimarinus/1-602 coralli
-------LVCCTVLLTLSVATYASEKSSVSNSQKTTPRHAVAMHGE---VKYPADFTHFEYANPKAPKGGKA
RLGVVSN-------SYDSFHPFVL-------KGVPAAGVSH-----------YLYDTLTVQSEDE-------
--AFSQYGLIAEKIEMPEDR----SWVVFHINPKARFQDGKPVTPEDVIFTFNSLIEHG--APFYKAYYGDV
SNVSKVAPNQVKFDFKNNENR-ELPLILG-QLPVLPKHYWD-------TRDFSKSTLD-APIGSGAYKIKSF
NTGRSITYERVKDYWAKDLPVRKGHYNFDEVIYEYYGDQTIALEAFK-AGEYDFRVEN--TAKNWATAY-TG
EKFDKGIIVKEEVKH--QQPVGMQAFIYNTRRKIFSDPKVREALGYAFDFEWTNKQLFFGQYKRTNSYFENS
ELAS----EGLPSEAELKILNAFK----DQLP------ASIFTTANEVPKTDGS-GNI----RKNLRKALKL
LKEAGWSIKN---RKLVN
NATS--EPFTFEILLYQ---KSFERIIHPFTKNLEKLGIDAKIRLV-DTNQYIGRIR---KYDYDMFIMT-L
AQ----SNSP-GNEQRDYWYSSNKDIPGTRNYIGVSDPVVDQLVDMIIAAPDRDS-LVTRTRVLD-RVLLSK
HYVIPQWHLP---------KQRIAYWK-TLNHPKVFP---KSG-VN-FDTWWTRIN----------------
----
>Desulfovermiculus/1-568 halophilus
----------------------------------VRTAHAVAMSGD---PKYPAGFSHFAYVNPQAPKGGLF
RQATIG--------AFDTFNPYVP-------KGYPAEDIG------------LIYDTLTVRSEDE-------
--PFTQYGLVADRITLPQDR----SWVRFHIDPRARFHDGHPITAQDVAFTFRLLMKHG--APTYKQYYADV
KDVQVLDHARVEFTFKAGENK-ELPLIIG-QLPVLPEHFWE-------EKSFPDAGLT-VPLGSGPYRIADF
KAGQFVRYERVEDYWARNHPANVGRYNFDAVRYDCYRDETVALEAFK-AGEFDFRLEN--TSKSWAKGY-DC
PALKQGRITKEEISH--SLPQGMQAFAMNLRRSVFQDRKTRLALAYAFDFQWSNEHLFFGQYTRTASYFSNS
ELAS----SGPPSEAELNILEPYR----KHLP------PEVFTQAYSPPSTAGD-TTI----RENLRTAHEL
LAQAGWRMEN---GRLTD
AQ-G--RPLSFEILLQS---PAFQRVCIPYTKNLQRLGIDASIRLV-DTSQYITRMR---EFDFDMTVAV-L
PQ----SLSP-GNEQRSYFHSSAADMPGSNNYMGIANPAVDALVDKVISAPDRES-LIVRTRALD-RALLWG
HYLVPHWHTD---------VFRVAYWD-KLKHPHNTP---PY-GLG-LYTWW--------------------
----
>Caenispirillum/1-589 bisanense
---------------------------------AVEPVVGVAMHG---EPRYTHGFPHFDYVNPNAPKGGEM
RLFTTG--------TFDTFNPYIT-------KGTPAAGVSM------------IYDTLLAPAEDE-------
--PFSEYGQIAETIQMPEDR----SWVAFNLNPKARFHDGEPITPEDVVWTFRFLTEKG--DPLYRFYYATV
KDVVKTGEHQVTFLFD-GTANRELPLILG-QLPVMPKHYWEKE-----GNDPTATTLT-PPLGSGPYRIGAF
EPGRYITFERVDDYWGKDLPVNVGRYNVDRIRYDYYRDRTVALEGFK-AGAYDLRVEN--SAKNWSVGY-DF
PAFHQGLVKKETFDN--DLPSGMQGYVMNMRRPVFQNPLVREALAYAFNFEWTNATLFYGLYARTNSYFDNS
ELAA----EGLPSPEELEILEPLR----AQLPE------RVFTEVYTPPKGDES-GNI----RDNLRTAFGL
LQKAGYEVDPK---TRKMVN
KETG--KPLAFEILLVD---PTFERVSLPFVENLKTLGIDATVRTV-DTAQYVNRMR---DFDFDMATSI-W
GQ----SLSP-GNEQREFWGSQAASQPGSRNYAGVQDPAVDRLIEQVVQAPDRES-LVTRVRALD-RVLQWK
HLVIPHWHS---------PVQRIAFWD-KFGYPEAR--PMRGIDLM---SWWVVPEKDGRVAAEQKK-----
----
>Methylonatrum/1-565 kenyense
--------------------------------------HGLSLHG---DLKYGPDFTHFDYVNPDAPKGGSV
RLSALG--------TFDNLNPYIL-------RGTSPSGMSD------------VFDTLTVQSADE-------
--PFSSYGLIAETIEVADDS----SWVRFTLREEARFHDGEPITVEDVIWTFDTLQSDG--HPQFRMYYSEV

ERAEKVGERAVKFHFT-STENAELPLIVG-QMPVLPKHYWE-------DRDFTRTTTE-PPLGSGPYRVSRV
DTGRAIVYERVEDYWAADLPVNRGRFNFDRLRFDYYRDATVAVEAFK-AGEYDLRQEN--IARNWATQY-DI
PAVRDGRIIMEEIDH--EIPTGMQAFFFNTRKDKFADPRVRKALGYAFDFEWTNRTLFNDSYARTKSFFSNS
ELAS----EGLPEGRELEILENYR----EQLSE------AVFDTVYEPPTTEGR-GGM----RANLRRALEL
LGEAGWEVGD---DGLTH
TETG--EVMRLTVLLDN---PSFERVTLPFTNNLERLGIDANVRTV-DSSQYQNRMD---EFDFDMTVQQ-I
GQ----SMSP-GNEQRSYWSCAAAETGGSRNYAGICDEAVDSLIDRVIYAADRDE-LVAATRALD-RVLLHG
HYVIPHWYL---------RSFRLVYWD-KFERPEIS--PDFSLGFD---TWW--------------------
----
>Neptuniibacter/1-589 pectenicola
----------------------LAVLSASVPADSVQVAHGISMHGD---LKYPPGFKHFDYVNPKAPKGGQV
IQSAIG--------TFDSFNQFIV-------KGNAADGLG------------LIYDTLLSRALDE-------
--PFSLYGLLAESIEVPNNR----SWIIFNLRPTATFSDNHPLTAEDVVFSFNLLRTEG--APFYKSYYADI
DKIEALNPHRVKFTFKNTLNR-ELALIVG-EVPILAKHYWQ-------DKAFQKPSLD-IPVGSGPYTIDSF
DAGRSITYKRNPNYWAKELPVKVGYNNFDTLIFDYYRDGTVALEAFK-AGEYDFRQEN--SSKRWATSY-SG
NAFDDGRIKTTELEH--QNPTGMQAFVINNRREQFADPRVRRALALAFDFEWTNKNIFFNAYTRTHSFFSNS
EMAA----TELPTPEELAILEPVR----DQVP------PEVFTQVYKAPETKGD-GKI----YTQLREARRL
LQDAGWIFKA---GKLVN
AETG--KQLSIEMLLYS---PAFERVVSPFIRNLERLGIKANIRLI-DVSQYIARLR---SYDFDIVVSG-F
GQ----SSSP-GNEQRDYWHSSSANKEGSRNLIGIQNPAIDYLVDQLIQSPNREQ-LVYRTRALD-RVLQWN
HYVIPQYHIN---------KYRVAYWD-KFEMPNIRPK--YDLG---FNTWWIKPEKDVK------------
----
>Magnetospirillum/1-591 molischianum
-----------VVVLCLALSLGLFMSATLCAAETPAPVHGLAMRG---APKYPAGFSHFDYVNPNAPKGGEI
RLSETG--------GWDSFNPFIV-------KGEAAGGSEL------------PFETLMIESADE-------
--PFSEYGLLAESIEMPADR----SWVAFTLRSKARWHDGKPITADDVVFSFDILKSKG--HPRFRFYYAAV
DKVEKVGERKVRFSFK-PGDNRELPLILG-QLPVLPKHYWK-------GRDFAATTLQ-PPLGSGPYKAGPF
ETGRSVTWVRVKDYWGTDLPVRRGQYNFDRIRYDSYRDTTVALEAFK-AGEYDWRMEM--EAKKWATGYQDW
SGLKDGRGFKEVFPN--QRPAGMQGYVYNLRRPLFQDPRVREALALAFDFEWTNKTLFYGQYKRTASFFANS
EMAS----TGLPGPKELEVLEPLR----DKVPP------QVFTTTYKPPETEGD-GNI----RANLRAAMRL
LEEAGWTVE---NGKLVK
DG----RPFVFEILLNQ---PIWERIALPFARNLERLGIEANVRVV-DTAQYKNRVD---QYDYDMVVQV-W
GQ----SQSP-GNEQVSFWGSTAAAEVGGQNLAGIQNPAVDALVAAIIAAPDRET-LVAYCRALD-RVLLWN
HYVIPQWHM---------ASDRVAWWD-KFGRPAII--PAAGVRPL---AWW--------------------
----
>Isoalcanivorax/1-587 indicus
-------------RVLLPALLAGLFSHG---LSAAPGEHAIAMHGE---PKYPADFAHFDYVNPDAPKGGRL
RRHTIG--------TYDSFNPFIS-------RGSPAAGL------------GMLYDTLTTSSSDE-------
--PFTQYGLLAERIEMPEDR----SWVIYHLREEARFADGKPVTAEDVVYTFTLLTEKG--QPFYSFYYGDV
EKVEALDKHRVRFSFSGG-DNRELALIVG-QMQVLPKHYWE-------ERNFERGTLE-PPLGSGPYRIVSH
DPGKRVVYERRTDYWGKDLPVNRGRHNFDRISYEYFLDDTVALEAFK-GGRYDLRLEN--VAANWANGY-QG
PALSRGDIIMDTFDH--DLPSGMQAFTYNLRRPLFQDRTLRKALAYAFDFEWTNQNLFHNQYRRTRSYFENS
ELAA----EGLPGKDELAILEPLR----DKLP------EEVFTSVYNPPKSDGS-GRP----RDNLLRAQTL
LREAGYTLRN---GQLHT
PDG---QPVRFEILIDS---ATWERVSLPFANNLRGLGVRAEVRRV-DHTQYIERLR---RFDFDMVVNV-F
PQ----SNSP-GNEQRDFWHSSAADRPDSRNLLGLQDPAIDALVDLVISAADRDA-LVTRVRALD-RALQWG
HYVIPNWYTD---------HFRIAYWK-HLAHPGKP----APHGLP-LDTWWH-------------------
----
>Oceanicoccus/1-612 sagamiensis
---------MIRNLLVAFSIYTLATSALAAPDNHSSLIHGIAMHGE---VKYPADFQRFDYTSAQAVKGGTL
RRGLQG--------TFDSLNPFIA-------KGSSGDKLS------------LLYDTLTVQSGDE-------
--AFSQYGLLAEHIEIPEDR----SWVIFHLHKTAKFHDGEAVKASDVVFTFNLLMEQG--APFYRSYYGGV
STVEALSPHQVKFTFKDG-VNRELALIIG-QLPVLPEHYWS-------SRDFSASSLE-FPLGSGPYQIQSA
DAGRSIVYQRVKDYWAEDLPVNRGINNFDTIQIDYYKDGVVVLEALK-AGRFDFRWEN--ISKQWATSY-TG
PAFDQGLLQKSMIEH--DNPSGMQCFLINLRKDKFKDSRVRQALNYAFDYEWSNKNLFYGLYARTNSFFANS
ELAS----SGIPEGRELEILEEFR----GSVP------DSVFTQTYTNPISDGS-GNN----RGNLRKAAKL
LKEAGWVVKD---NQLVN
AKTG--QPFTIEMIIYA---PSSERIVNPYAKALKRLGIVMTVKNV-EISQYINRMR---SFDYDMVTGG-M

GQ----SLSP-GNEQMEYWHSSSADKQGSRNYAGIQNKAIDKLVELVIAAPSREE-LVYRTRALD-RVLLHN
HYVVPQFHSG---------AHRIAYWD-KFGQPDIAPKYDSGYSMG-LMTWWVDPDKEKQLNNAKQ------
----
>Aestuariirhabdus/1-595 litorea
---------------------IACLFPAIGSAEQSIQSHGLSLYG---EPKYPVDFHHLDYVNPDAPKGGSV
RFMATG--------TFDTLNPYTL-------KGLSPVNTPGFF----LYGVSESNETLMAGTDSMTRS---G
DEPLTAYGLIAERIEYPADF----SWISFHLRPEARFHDGSLILATDVGFSFNTLIA--EGHPRYRALYRNV
TSAEVIAERSIRFNLQ--GNNRRRLAISLGELPVFSERYWRER-------TFAATTLE-PPLGSGPYRISAV
DPGRSITFERVEDYWGSDLAINRGRNNFDRIRFDFYRDLTVAFEAFK-AGQYDIHLEY--ISKNWATGY-DF
IDKHPGEVIKREIPD--ANPANAQGFVFNTRLPLFSDRRVREALTLMFDFEWVNQSLFYGAYRRSNSYFANS
ILASR----ELPPPEERALLEGLD----SLDPR-------LFNQPFQLPVTRGD--GN---QRVQMRQALAL
LKEAGWSLRQ---GQLVH
QRTG--HPFRFEFLIRQ---QSLGRVLVPYQQSLKKIGIDMEIRLI-DSAQYKNRMD---SFDFEMTTIV-L
GQ----SLTP-GNELRQYFHSANANIQGSQNYAGIDNPAVDSLVERILSAASLEE-LGVATRALD-RVLLWQ
YYMVPNWYIA---------SHRVAYWN-RFGFPQSTPPYD-----LSLSTWW--------------------
----
>Moritella/1-565 dasanensis
--------------------------------------YAIAMHGQ---PVYDKNFTHFDYVNLNAPRSGSL
RRAAMG--------SFDNFNAYIV-------KGVTADGTG------------YLFDTLMQQSSDE-------
--AFSLYGLVAEFIEVPDDR----SWVRFHLNPKARFSDGSALTASDVEFTFNVLMEKG--VPQLRAQYKEV
TKVEVESKSVIKFSFKDNKNK-ELALILA-QLPVFSEKDWQ-------GKDFAKATLN-IPLGSGPYTIKKF
DAGRSIDYQRNDDYWAKDLPVNRGRYNFKNVIFDYYKDGSIAFEAFK-AGDVDFRAEN--ISKQWATGY-QG
KQFTSGNIIKEEIQH--QNPQGMQAFWFNLRKDKFKDPNVRKALGLLFDFEWTNKTLFYGAYKRSDSFFSNS
ELAA----SGIPQGDELALLNPFK----AQLP------AELFTQVYTLDKTKGD-GRV----RKQQRQAITL
LKQAGWTLKS---GKMLD
TN-G--KQLSLEFLVYD---PSFERIIQPFRKNLQRIGIASEIRIV-DVSQYINRLN---NFDFDIYTLT-Q
AQ----SLSP-GNEQLSMWGSEFANVPGTLNRIGLQDPVVDALVMQVIKAEDRDS-LITATKALD-RVLLWK
NLMIPQWHIS---------SYRVAYWQ-QIQRPEKLPK--YGLA---IDSWWH-------------------
----
>Psychromonas/1-600 arctica
---------------LIRICLFYMLFSVSIGAQSASWNNAISMYGD---LKYDADFEHFDYANPNAPKGGVF
KQASIG--------SFDSLNPFIV-------KGNAASGIT------------RIYDTLLQQSTDE-------
--PFSLYALIASQVKVADDF----SSVSFLINPKATFQDGEAITASDVKFSFDLLVNEG--APHFSSYYAGV
EKVTVDALLQVTFHFKEVGNR-ELPLIIG-QIPILPEHFWK-------DIDFSKSGLI-VPVGSGPYQIESF
DAGKQVVFQRVKNYWGKDLAVNKGRHNFDHIIVDYYRDDSVAFEAFK-SGAFDYRLET--SSKRWSTGY-VG
EQFNKQHILLETIAD--KTPQGMQGFWFNLRRDKFKDPKVREAISLLFDFEWANKTLFYDAYTRIDSFYSGS
ELAT----QSEISPAEQAILLPYK----EQLP------ASVFEPIKAN-KTSGN-GNV----RQQMREAVAL
LAEAGYQLKD---SKMQN
QQ-G--EQLSFEFLLYS---KDFERIIHPFRRNLQRIGIKADIRLV-DVSQFINRLN---SFEFDMLSLR-K
GQ----SISP-GNEQASFWGCDTANDAGTSNWAGICSPVIDALTQQLIMATTREQ-LVNTTKALD-RVLLLQ
HMVIPQWYLP---------AYRIAYWD-KFSRPKVSPY--YDLG---LDTWWLKASSGVTTSEGSQ------
----
>Marinospirillum/1-567 minutulum
-------------------------------------SHALTLHGE---AKYPADFAHLDYVNPKAPKGGSL
SQAAQG--------TFDSFNPFIV-------QGTAAAGLGN------------IYDSLTYHTADE-------
--PFTEYGLLASCMQLDPAG----RWIEFELRPEARFHDGKPVTAEDVAFTFKLLREEG--RPFYRAYYADI
TAIKVLNSQRIRFELASHENR-ELPLIIG-QIPILPKHYWQ-------DRDFKQPSLD-KPLGSGPYKIAEV
DSGRRLVYQKVANYWGENLPVNLGRNNIEQLVYDYYRDATVALEAFK-AGRLDFRLEN--IARNWATGY-SG
SALSSGDLVLESIPH--KNSAGMQGFFFNTRRDVFKDPKVRLALTQMFDFEWSNQQLFHGAYTRTQSYFSNS
ELAS----SGLPEGNELALLTPFA----KQLP------DEVFNQEYSLPVTDGS-GNI----RNQTRKALGL
LREAGWTMDG---RRLVN
AA-G--EQLRFEILLHD---TSFERVVLPWRQNLERIGIRMDVRVV-DVTQYLNRLR---SFDYDMVVSS-I
GQ----SLSP-GNEQREFFHSEFAHSANGRNTSGVEDPVVDALIEQLINAQDRDS-LITATRALD-RVLLWG
HYVIPHWHLN---------EYRIARHK-QIKIPEVRPD--YGLP---IDTWWVE------------------
----
>Desulfonatronum/1-589 thiodismutans
----------------LFMFFSLLLSAPSPSSAEQPWRHGLALHY---DLKYPPDFTHFDYVNPDAPKGGEL
RLAGIG--------TFDSLNPFIL-------RGTPPLGMGM------------IFETLTERSQDE-------

--PFSEYGLIAESMLIPEDH----SWVAFTLREEARFHDGSPITVQDVIFTLELLQTKG--HPFYRAYYANV
VSAEEIGPRQVRFSFG-ESVNRELPLIIG-QMPVLSKAYWQ-------DRDFDRTTLD-IPLGSGPYRIARV
EPGRSITYQRVEDYWAADLPVNRGRYNFDVMRYDYYRDVNVALEAFK-AGQYDFRQEN--VARNWATGY-DG
PALRQGRIVMAEIPH--ELPTGMQGFVFNTRRPMFRDPLVRQALAEVFDFEWSNANLFHGAYTRTASYFSNS
ELAS----AGLPPPEELTLLEPHR----DILPE------SVFSTEFQPSVTDGS-GNI----RENLRRALDL
LEQAGWTISGR---DRKLRH
ASGG--EALEFEILLND---SIFERVCLPYARNLERLGITARVRTV-DATQYQNRMN---DFDFDMTVGL-F
PQ----SLSP-GNEQRDFWTSEAAATPGSRNIAGVRDPVVDELVDLVIAAPDRDS-LVTRARALD-RVLLHG
HYVIPHWHS---------REFRVAYWD-KFARPEIN--PRYGLALD---AWW--------------------
----
>Terasakiella/1-606 pusilla
----------------VVLAGLLGLSLTFSASAAEKRYAVSMHG---DIKYGPDFTHFDYVNPDAPKGGSI
RLSQIGS-------AFDTLNGYTL-------KGIPAIGSSL------------PIETLTVQSMDE-------
--PFTSYGLLAETIEMPEDR----SWVAYHLRKEARWHDGKPVTADDVVFSFNTLMKEG--HPQYRFYYGDV
AGVEKTGDLSVKFTFK-EGENRELAMILG-QLPILPKHYWE-------DKDFTKTTLE-APLGSGPYKVKSF
EPGRNIVFERVTDYWGKDLAVNAGQNNFDEIRFDYYRDATVALEAFK-GGAFDYRYEN--IAKNWATAY-DI
PEVEKGLIQKLRIEH--EVGTGMQGFAFNIRRDKFKDPLVRQALAYGFDFDWANKNLFHNEYSRTTSYFSNS
ELASS---G-VPTGRELEILEKYR----GQIPD------EVFTTEYQPPKTDGS-GNI----RGNLKQAVTL
LKKAGYGVK---DSKMVN
LKTG--EPLAFEILLQA--SPQWERIVLPFKKNLERIGIDVTIRMV-DTAQYKERTD---TYDYDLTVEV-F
GQ----SQSP-GNEQYDFWTSKSADRQGSRNAIGIKSPVIDELVQMVVNAPDREE-LIMRTRALD-RVLLWG
HYVIPNWHL---------KVARIVAWD-KFGRPDTW--AKYNPGYTAWQFWWYDQEKAKKLESQRKK-----
----
>Micavibrio/1-597 aeruginosavorus
----------MLKILSGVVVLALLSFPALAQDAAVKPVHALAMHG---APKYAADFKHLDYVNPNAPKGGTF
RQHVIG--------TFDSLNPHII-------KGAPGAGSS------------LVNETLLEQSADE-------
--PFSEYGLIAETIEVPEDR----SWVIFNLRPQAKWHDGQQITADDVVWSFNTLTTEG--APFYKAYYHNV
KTVEALSPTRVKFTFDMAG-NRELPLIVG-QLPILPKHYWTAEG-----RKFGESSLT-PPLGSGPYKYGTV
KPGELIELVRVQDWWAKDLPINVGRYNFDKLTYEYYRDANVALEAFL-GGQYDFRQEN--TAKTWATAY-DA
PVVKSGQVKKEEVHH--TLPQGAQGFIFNLRRPIFQDISVRRAISYAFDFEWSNKQFAHDAYTRTRSYFSNS
DMEA----KGQPSAAELAILDPFR----DQLLP------GVLAEEFNPRKTDGS-GNN----RDMLAGANEL
LNKSGYVMGK--DGVRVH
SITG--QRLEFEFIDN--N-PAFERWIAPFIQNLKKLGIKATYRTV-DDAQYQNRMN---NFDFDMTVMV-Y
PQ----SSSP-GNEQREFWGSDRAEMPGSRNYIGIKDPVVDALIEKIVAAQTREE-LIVLCSSLD-RVLQWG
YYMVPNWYL---------SAWRIAYWD-KFEKPA--IAPTYGMG--VTDTWW--------------------
----
>Denitrificimonas/1-591 caeni
------------MLTALRRGCLSLCVLVCSTAALAEPQHAMTLYDE--APKYPSDFTHFDYTNPDAPKGGTL
RLAGLN--------GFDSLNPFIP-------KGNAGDQIG------------LLYDTLTYHSADE-------
--PFTEYGLLAERIEKADDH----SFVRFYLNPKARFNDGQPVTAEDVLFTFNTLIEKG--HPLYRHYYADV
AEVVVENPLQVRFDFKHSNNR-ELPLILG-QLQILPKHWWA-------ERDFTKTSLT-APLGSGPYRLVQV
DAGRSLRYERVKDWWGKDLPVSRGLYNFNTIYIDYYRDMSVALEAFK-AGQFDFNLEY--SAKDWATGY-NS
PALRSGAIIKKSIKN--HNPVGMQGFTFNLRKKMFQDPRVRQALSLLFDFEWTNKQLFYSSYIRTQSYFENS
ELAA----RGLPSEEELAILEPLR----GSIP------EQVFTQAYQTPISDGS-GII----RERRLEAYRL
LQEAGYRIEN--DQMVN
AE-G--EPLAFEFIIAQ---ANLERVLLPFKRNLAELGIEMTIRRA-DVSQYINRMR---SRDFEMTSSI-W
SQ----SNSP-GNEQRDFWHSSSVDNPGSRNFIGLQDPAIDSLVEGLINAPSRAS-LITHARALD-RVLLWG
HYVIPNYYID---------TWRTAYWQ-HLAQPEVAPL--YDYG---LMTWW--------------------
----
>Alloalcanivorax/1-593 venustensis
------------MRCLVCCLFFVAALFGGPALAETQRSHAITMHGE---PKYPAGFKHFDYVRPDAPKGGSL
SLHVVG--------GFDNFQPWLP-------KGQAAAGS-----------QGLVFDTLTVRSKDE-------
--PFTEYGLLAESMEWPEDR----SWVTFTLREQARFADGHPVRAEDVVWSFKQLRDKG--APFYAYYYGDV
EKVEALSERKVKFSFKAG-DNRELVMIVG-QLPVMPKHYWD-------DKPFDDANLV-PPPGSGPYKVDSF
KAGKRVVYQRRDDYWAKDLPVNRGHHNFGRIIYEYYLDQTVALEAFK-RGDYDWRSEN--NSKYWATAY-TG
EAFRDGEIITEEVTH--QNPAGMQGFIFNTRRPLFQDPVLREAMTYAFDFEWSNKNLFYGQYKRTRSYFQNS
ELAA----TGLPDEKELALLKPLR----EDLP------PRVFTEAYQPPVSDGS-GRP----RDNLRQAQAL
LKDAGYQVKD--GKLHT

PDG---EPVSFEFLLYQ---PAFERIVLPYARNLKTLGIDADVVRV-DQSQYVQRVR---NFNFDMMVGG-W
GQ----SPSP-GNEQRGYWGSAAAERDSSQNYAGVSNPAIDELVSKVIAANTREQ-LVTRTRALD-RALQWG
FYVVPNWYLD---------YHRFAYQS-RLAHPPLPPY--VGFDGA-TELWW--------------------
----
>Desulfonatronum/1-589 lacustre
----------------LLLVFTLFLAAPASSGAEPPWRHGLALHY---DLKYPPDFSHFEYVDPEAPKGGEL
RLAGIG--------TFDSLNPFIL-------RGTPPLGIGM------------IFETLTDRSQDE-------
--PFSEYGLIAESMRIPEDH----SWVAFTLREEARFHDGSPITVEDVIFTLHLLQTKG--HPFYRAYYANV
ASAEEIGPRQVRFSFG-EAVNRELPLIIG-QMPVLSKAYWE-------NRDFERTTLD-IPLGSGPYRIVRV
EPGRSITYERVQDHWAADLPVNRGRYNFDVMRYDYYRDVNVALEAFK-AGQYDFRQEN--VARNWATGY-DG
PALRQGRIVMEEIAH--ELPTGMQGFVFNTRRPMFRDPLVREALAEVFDFEWSNANLFHGAYTRTRSYFSNS
ELAS----SGLPSPEELALLEPRR----DILPE------EIFTAKFQPSATDGS-GNI----RENLRRALGL
LEQAGWTISGR---DRKLRH
ASGG--EAMEFEILLND---SVFERVCLPYARNLERLGITARVRTV-DATQYQNRMN---DFDFDMTVGL-F
PQ----SLSP-GNEQRDFWTSEAAATPGSRNIAGIRDPVVDELVDLVIGAPDRDS-LITRTRALD-RVLLRG
HYVVPHWHS---------REFRVAYWD-KLARPEIN--PRYGLALD---SWW--------------------
----
>Aliagarivorans/1-590 marinus
--------------MLRLFVWCFSVLLGVAHAQDFTRSHALIMHGE---PALAADFAHFDYVNPEAPKQGMV
TFDATG--------TYDTFNPFIV-------KGNSVAGIG------------RIYDTLLTSNPDE-------
--AFTQYGLLAETIDLAEDY----TWVRFNLRPEARFHDGHPLRAEDVVFTFNTLIEEG--APFYRAYYGNV
VKVEAESELAVRFTFKDGSNR-ELPLILG-QLPVLPKHYWA-------DKEFNKADLT-RPLGSGPYQIDSF
DAGRSIRYALVKDYWGKDLAVNVGRNNFAQINYEYYRDSSVALEAFK-AGRIDYRLET--SAKDWATAY-TG
PQFDSGEALTVSLEN--HNPQGMQSFAFNTRRDKFSDIRVRQALGLMFDFEWTNQQLFYGAYSRSESFFAAS
ELAA----TGLPSEGELALLKPFK----AQLP------EALFTQPFRLDKTGGD-GNL----RPQMRQALAL
LKEAGWSLQQ---GKLVD
DK-G--EQFKIEFLLFQ---KNFERIVLPYTRNLQRIGIASEVRLV-DVSQYINRVQ---SFDFDMMVAS-F
PQ----SSSP-GNEQREFWGSKAADQVGSRNVIGIKSEVIDSLVETLIHAPDRQA-LIDASRALD-RVLLWS
YYVVPQWHLS---------AWRVAHWN-KLQRPERNPQ--YDIG---LDSWWVE------------------
----
>Hypericibacter/1-592 adhaerens
----------------------TFGPKARADGGTNGKAHALAMHG---EPKYGPDFKHFDYVNPDAPKGGQL
RMSVPNST------TFESFNPFIV-------RGAPAAGSDG------------IYESLTVASNDE-------
--AFTQYGLLAQYIETPDDR----SWVEFTLNPAARWWDGTAITPADVIFTLETLRAKG--KPFYRFYYRSV
AKVEQTGDLGVRFTFV-PGDNRELPLIIG-QMSVLCKAYWE-------TRDFEKPSLD-LPMGSGAYRITAF
EPGRFVVTTRVQDYWGRDLPVNVGTGNWDSVRYIYYKDETVALEGLK-AGDFDIRQER--SAKIWATGY-DA
RPVRDGRIVREQIPN--EIPTGMQCFPFNIRRPIFQDRRVRQALGYAFDFEWSNKALFFGQYTRNASYFSNT
ELASS---G-LPQGEELAILEKYR----GKVPD------EVFTSVYEPPKTKGD-GDI----RDNLLKARAL
LQASGWVIR---NERLVK
QETG--EPFQFEFLLFD---PMFERIVLPFLRNLRRLGIVTTLRTV-GVAEYQNRTD---NFDFDMISGG-F
GQ----SLSP-GNEQADYWGSASADQPGGQNIIGIKDPVIDELVDLLIASPDRES-LIQRTRALD-RVLLWG
FYVIPNWHL---------RSWRVAYWN-KFSRPAIA--PKYDLGLD---TWWIDPAKAAAL-----------
----
>Rhodospira/1-579 trueperi
--------------------------------------THAIAMHG---DPKYPPDFTHFDYVNPDAPKGGTL
RLAWRG--------GFDSLNPYII-------KGRPPIGIGL------------TYDTLMTASADE-------
--PFSHYGLVAESIETPDDR----SWVIFRLNPDARFHDGHPITAEDVLFSFDVLREHG--APLYRFYYKDV
DGAEALDDHTIKFTFS-TDMNRELPLVLG-QVPLLPKHYWE-------GRDFAETTLE-PPVTSGPYRIADF
EPGRFIRYERVKDYWGNDLPVTAGTYNFDAIVYDYYRDETVMVEALK-AGDYDFRSEY--VAKTWATAF-DG
EDLEAGRLVKRVFET--SQVAPTQGYVMNMRRPPFDDPAVREAMAYAFDFAWANQNLFYGSYTRTRSNFDNS
ELAA----TGVPEGDELALLEPFR----DQLPP------RLFTEEYNPPSVEEA-GNI----RSNLRRGLEI
LREAGWTVQ---DGVMTN
AETG--QTLAFEILLRQ---PSMERLTLPYVQNLKRMGVEATVRIV-DTAQFVNRVN---TFDFDMISAI-W
AQ----SDSP-GNEQRDFWSSETANVEGSRNLPGVTSPVVDALVEEVIRADSRED-LVTAVHALD-RVLQWT
FYLVPHYHE---------ENDRVVFWN-RFGIPEVT--PDSGAAVM---TWWIDPEKDAALRAAG-------
----
>Defluviicoccus/1-598 vanus
------IRRCLVVAVALIAFHLSPADHALAAEAGIHTGHAIAMFG---EPKYPADFKHFDYVNPAAPKGGEM

RQGVAG--------TFDSFNPFIT-------KGNPAADV--------------ANESLTVASQDE-------
--PFTQYGLLAESISWPEDR----AWVAFTLRPQAHWHDGAPVTVEDVIFSLQLLKTKG--QPFYRFYYAAI
DHAEKIDARTVKFVFK-QAGNRELPLIAG-QMPILPKHYWE-------GRDFERSTLE-PPLGXGPYRVGTF
EPGRFVVLERVADYWGRDLPVNVGQNNFDRLRFDYFRDETVLRQALK-AGVLDFRVEN--QAKAWALDY-DI
PAVREGWLQKVLFPH--RRPAGLQAFVFNTRRPVFADPRVREALGYAFDFEWTNRVLFFDQYTRARSYFNNS
DLAA----IGTPSPAEAAILAPYR----GRVPD------AVFGPAYEPPATDGT-GWP----RANLERAFAL
LREAGWVVR---DLKLVN
AASG--EQLAFEILIDT---PTFERIMLPFVRNLTRLGIDVSLRLV-DDSQYVNRLR---AFDFDMAMLV-W
GQ----SXSP-GNEQRDFWSSTAAQAEGSRNYAGIHDPVVDELIEQLIVASDRDS-LIAHTRALDXRVLSWN
FYVIPGWYS---------QADRVLYWD-KFAYPAVS--PDQGVSRD---TWWYD------------------
----
>Aestuariicella/1-593 albida
-------------LLISTALLMIVN-GPAAVSAEIIKSHAIAMHGQ---PKYGPDFTRFDYTSADAKKGGEI
KLHSIG--------TYDSLNPFVA-------KGNPADRLG------------LVYDTLTTSSADE-------
--PFTQYGLLAKTLEYPEDR----SWVIYHLDPDARFNDGSQVTAEDVDFSFKLLLEKG--NPFYGYYYADV
IKSEVLDRSRIKFTFREG-SSPETLLITG-QLPVFPKHFWT-------DKDFEHSALL-RPLGSGPYQITQV
DPGRSITYERVSDYWGKNKPAQKGLFNFDKIVVDYYRDDTVALEAFK-AGEYDFRYER--VAKLWATAY-DS
QAINDGRIIKKEVAH--NNPAGMQAFVFNLRNPLFQDIALRQAIGMAFDFEWTNKNLFYGAYSRTNSYFANS
DLAS----SGLPSKAELALLEPYR----DQLP------STVFTEEYKSPESKGD-QHN----RKNLRAAKKL
LDSAGYKVVN---GKLMT
PDGK--QPVNFEILLYD---SAFERIANPFVQGLKKLGIDATVSKV-DTSQYINRRR---DFDFDMITHV-F
GQ----SLSP-GNEQRDFWSSHAAETPGSRNIIGISNPVIDALVDKVIAAQTREQ-LVTATHALD-RVLLNM
HYVIPQWYTS---------SHRLAYWD-KFNQPSTSPEYDVGYGTG-LMTWW--------------------
----
>Ventosimonas/1-593 gracilis
------------------MRLLPLLFACLAPLAFAAPKHAITLYDE--APKYPPDFTHFDFVNPDAPKGGRL
KLASYG--------GFDSLNPFIP-------KGNVESRIG------------LIYDSLTFQSPDE-------
--PFTEYGLLAETIDKAADN----SSVTFNLNPKARWHDGEPVTAEDVQFTFEQLLKHG--DPMYRHYYADV
EKVEILSAKTVRFVFKRNDNR-ELPMILG-QLQILPKHWWA-------SRDFSKTSLE-IPLGSGAYRVEKL
EAGRSIQYSRVKNWWAKDLPVTRGFNNFDRIDIDYYRDMSVALEAFK-GGQFDFNLEY--SAKDWATGY-AS
PALNDGRMIKMEIPN--HNPVGMQAYIFNLNRPLFKDKRVREAIGLLFDFEWTNKQLFFGAYKRTSSFFENS
EMAA----HQLPSAAELAILEPLR----GQIP------DEAFSQVFTLPQTQGD-GII----REQKRRAFSL
LKEAGYSIQN---NQMTG
PD-G--KPLSFEFMLHQ---TNLERVILPFKRNLSELGIDMQIRRV-DVSQFINRMR---SRDFDMTSAI-W
SQ----SNSP-GNEQREFWHSASADNPGSRNLIGLRDPAIDQLVEGLIRADSRQE-LINQARALD-RVLQWG
YYVIPNYHTD---------QWRVAWWN-KFGRPKITPL--YDIG---LMTWWQKSEKAQR------------
----
>Denitrobaculum/1-582 tricleocarpae
-------------------------------SQVVYKSHAIAMHG---EPKYPADFKHFDYVNPDAPKGGEI
RTAARG--------TFDSFNRFIA-------KGTPAGTG--------------SIESLTVSSTDE-------
--PFTQYGLLAESIEWPEDR----SWVAFTLREEARWHDGMPVTVEDVIFTLETLKTKG--HPFYRFYYGSV
ERAERVGERKVKFVFS-EKGNRELPLIVG-QLEILPKHYWE-------DREFDETTLE-PPLGSGPYKVSRF
EAGRYIIRERVEDYWGKDLPVNVGQNNFDRLRTDFFLDTTVIRQALK-SGTIDYRNEN--QAKAWASAY-EV
PAVRRGWLQKEQIKH--ERPTGMQAFVFNMRRQLFQDPLVRQAISYAFDFEWSNPTLFFGQYNRTESYFSNS
ELAS----SSLPQDEELEILERYR----GRVPD------EVFTKTFAAPQTDGQ-GWP----RDNLDKAFRL
LSQAGWVVR---DLKLVN
AETN--EQFGFEILLVS---PEFERIVLPFKRNLERLGMNIRVRIV-DTSQYVDRLR---NRDYDMISTG-W
GQ----SESP-GNEQRDFWGSVAADQAGSRNYTGVKDPVVDELIELVITAPTRES-LIQRTRALD-RVLLWS
HLVVPAWHL---------QYDRILYWD-KFSRPDVT--PKRGTSTN---YWWYDEAKAAALREA--------
----
>Azotobacter/1-561 vinelandii
---PKYPADYRHFEYVNPDAPKGGTL
RLADYG--------GFDSLNPFIP-------KGNVERRIG------------MVYDSLTYHAQDE-------
--PFTEYGLIAEKIEKAPDN----GFVRFYINPKARFHDGRPITAEDVKFTFETLIEHG--DPMYRHYYADV
AQVVVEEPLKVRFDFKHRDNR-ELPLILG-QLQILPKHWWE-------SRDFAKTSLE-APLGSGPYRVAKL
ESGRSIRYERVADWWAKDLPVSRGQYNFDAIVVDYYRDMSVALEAFK-GGQFDLNLEY--SAKDWATGY-ES
AALNDGRMIKKAVPN--HNPVGMQAFAFNIRRPLFQDRRVREALGLLFDFEWSNKQLFFSSYKRTSSYFENS
EMAA----HQLPDKEELKILEPLR----EQLP------PEVFSEVYRPPVTNGD-GII----RDQKRRAYQL

LQEAGYRIEN---DRMVG
PD-G--KPLAFEFMLHQ---TNLERILLPYKRNLGELGIDMQIRRV-DVPQYINRMR---NRDFDMTSAT-W
PQ----SNSP-GNEQREFWHSSSADNPGSRNFIGLRDPAVDRLVDGLIRADSRKG-LVAHARALD-RALQWG
FYVVPNYHVN---------TWRIAYWN-RFGQPQKTPL--YDYG---LMTWWQDSDK---------------
----
>Fodinicurvata/1-589 fenggangensis
-----------------------ALAEEGPEDEAPRPRHGIAMHG---EPKYPADFTHFDYVNPDAPKRGAV
RLAARG--------TYDSFNPFVV-------RGVAASGASY------------LNETLMVSSADE-------
--PFSMYGLVAESISVPDDR----SWVEFTLREEARWHDGEPISVEDVIFSLNLLREEG--HPIYRQYYADV
TAAEKTGPRKVRFTFS-EGENLELPLIIG-QLPILPKHYWE-------ERDFTRSDLE-PPLGSGPYRVADF
EPGRHVTYERVEDYWGKDLPVNTGRYNFDRIRYDYYRDETVIRQAVK-AGEVDFRQEN--QAKAWALDY-DT
PAVEQGRLVMEEIPH--ERPTGMQGFVMNTRRPVFSDPKVRRAMAYAFDFEWSNRNLFFGLYDRTESYFSNS
ELAS----SGLPEGRELELLEPYR----EELPE------EVFEEAYFAPSTDGS-GWP----RQNLKKAFEL
LEEAGWVVR---DMKLVH
EDTG--EQMTFQIMIYD---TAFERVVLPYVRNLRRLGIEPTVRLV-DTSQFVNRLR---DFNFDMTISS-W
GQ----SDSP-GNEQRNYWSSSAAEMPGSRNLAGIQDPVVDELINEVIRAGSREE-LVAATRALD-RVLLWG
HYVVPHWHS---------RVDRLVYWN-KFGHPEDP--PLHGFEFD---NWWIDEDRARRL-----------
----
>Varunaivibrio/1-583 sulfuroxidans
----------------------------------VAPRHALAMFG---APKYPAGFAHFDYADPKAVKGGSV
RLAAIG--------TFDNLNPFIL-------NGVAAAGAGL------------PFETLTVSSEDE-------
--PFSVYGLLAESMQVPKDR----SWVAFTLRKEARWQDGAPVTTDDVIFSLNILRTKG--HPFYRSYFARI
DKAVKIGPRTVKFIFK-PGSNRELPLIAG-QLPILPKHYWQ-------GRDFSKTTLE-APLGSGPYRVASV
DPGRSVTYARVKNYWGAKLGVNVGQNNFGAMRYDYYRDATVALEAFK-AGDYDFRQEN--ISKNWATAY-TG
PAVAKGLIRRASIAN--EQPTGMQGYVFNIRRPLFKDRRVREALTYAFDFEWTNKNLFYGQYTRTESYFSNS
ELASS---G-LPKGRELAILERYR----GKIPD------AVFTRPFHAPRSDGT-GFN----RKNLRIAAKL
LQQAGWSVK---NGVLTN
EKTN--QPFRFEILISQ---PAWERITLPFIKNLKVLGIDARLRVV-DSAQYIKRSE---SFDYDMIVDV-F
GE----SLSP-GNEQRDFWGSQSADRRGGRNTIGIKNPVIDDLVNLVVSAKTRRQ-LLDRTHALD-RVLLWE
YYVIPHWHS---------QAFHVAYWN-KFSRPKVS--PKYGLCFS---CWWVDPQKAAHLKALKK------
----
>Natronocella/1-590 acetinitrilica
------------MRHRIARWMVSGL-LCGTMGVALADGHGLSLYD---SPKYPADFEHFDYVNPDAPKGGQI
RLAGFG--------TFDSLNQFIL-------RGNPASGLGR------------VYDTLTVNSDDE-------
--PFSIYGLVAERIELADDR----SWVRFHLREEARFHDGEPLTADDVVFSFDLLRTEG--HPNFRLYYADV
ESVEREGDHQVRFNLG-SPDNRELALIVG-QLPILPKHYWE-------GREFNRTTLE-PPLGSGPYRVTRV
DQGRQITYERVDDYWARDLPVNRGRFNFDRIRIDYYRDMDVAVEAVK-AGAYDLRYEN--IARNWATAY-DV
PAVRDGRMRMVEIPH--ERPTGMQAFIINNRQERFSDPRVRKALGYAFDFESTNEAIFYGAYTRTESYFSNS
DLAS----TGVPEGQELEILEQFR----DELPE------SVFDQAFQAPRTDGG-GSI----RDNLRRALAL
FREAGWEVRD---GRLVN
AESG--ERMSFTFLMRD---QSFNRVIERYRPNLERLGIEARVRIV-DDSQYQNRMN---EFDFDVTVLV-L
PQ----SRSP-GNEQRSYWSCEAAETPGSRNYAGICDPVIDELVDQLIRARDWDT-LQATARALD-RVLLHG
HHVIPHWHT---------RSDRVVYWD-RYAYPDTP--SGDGFDVD---LWW--------------------
----
>Paradesulfovibrio/1-604 bizertensis
--------------VVCFVFLGLGLLGFYPQSSEAKVLSALTLGK---APQLSSDFQHFPYANPDAPKGGQL
RLASIG--------TFDSFHPYVA-------RGIPAAGIG------------LVNATLTTSSRD--------
-EPFTQYPYVASSFELVDGG----ATLLCHLNPRARFSDGHPVSAEDVVFSFDALMHKG--SPMYRKYYAGI
VRVTARDAHTVRFELA-DAKNPELPVVAA-QLPVLPAHWWK-------GRDFSKPTLE-QAPGCGPYVVKRS
STGYSVEYERVKDWWGADLPVNAGRFNFDRIRFDYYRDRTVAGEAFR-SGEFDFQMVS--SAKSWAEEY-VG
PAVDAGLLKREAIRH--TRPSGMQGFVMNTRRPLFADRKVRQALGLAFDFEWTNRALFYGQYARCLSFFSNS
EFAS----SGVPQGKELSLLEPYR----SALPQ------ELFVSPFTLSKSDGS-GRI----RPQLRKALTL
LREAGWTLRDG--VLR
NAQG--KSFEFEMLLRS---PSMERVVLPYQKNLKRLGITMHVSLA-DSSRYIRRIR---SYDYDMVVGV-M
RQ----SDSP-GSEQRLFWTSSAAKTPGTRNLAGIQNPVVDGLVDKVIGAQNRDE-LLTAVHALD-RVLLWE
AYVVPGWYS---------PIDRIAYWD-RFGMPKLRPSRGTDLF-----SWWFSPEGDARIQAAGFSG----
----
>Helicobacter/1-571 canadensis

```
---------------------------------------AFAINGE---VKY-KNFKHFDYVNPDAPKGGHI
KEYAIG--------TFDSFYDFLL-------KGTSAQGLH------------LIYDTLMVRSLDE-------
--PSSQYGLIAKQIQRAKDN----TFVIFHLDPNARFNDGKEITAFDVEFSFNLIAHGE--NPSMNRYYADI
KEAIVVDKHTIRFNFKDSKNR-ELALILG-DLPILPKHYYE-------TTTPKANPLR-IPLGSGPYKIESF
EAGRSITYKRVENYWAKDHPTRIGYFNFDRITFDYYKDDSVALEAFK-AGRYDYRQEM--SAKNWALGY-NG
KALKNGEIIKQEILH--SLSSGMQGFVFNLRKNIFQDIKVREALGLAFDFEWSNKNLFFNQYARTTSFFDNS
EFAS----VGIPLGEELKLLESFK----DNLP------QELFTQSFKLPTTQGN-GNN----RDNLKKAQKL
LKEAGFVTKN---------------------------------------------------------GKLYD
KNN---QPFVFELLLVS---PAMERVAIPFAKNLQILGIEMKIRLV-DISQYINQLR---TFDYDMIVGV-F
PQ----SLSP-GNEQSFFWGSSAADAEGSYNYAGIKNPVIDSLIQKVINAEDYQD-LLTATRALD-RVLLWN
YYVIPHYHTK---------TFRIAFWN-FLEHPKITP---IY-DVG-FETWWVN---ENKLLKI--------
----------------
>Balneatrix/1-590 alpica
---------------LLARLLSFSLALTPLAQADDHVTHALAMHGQ---PKYGADFSHFDYVNPDAPQGGTL
RQASIG--------TFDSLNPFIG-------KGVSAAGSG------------LLYDTLAVHAYDE-------
--PFTMYGLVAKDIILANDR----SWIEFRIHPDARFQDGHPISAEDVAYTFKLLTEQG--NPFYQAYYHDV
GQVEVLDSQRIRFQLTNPENL-ELPLILT-QLPVLPKHYWQ-------DRDFQTTSLE-PPLGSGPYKVEKV
DAGRSISYVKVADYWAKNHPAVKGHYNFERMQYDYYRDEDVAVEAFK-AGEFDLRIES--SAKRWATGY-QS
PALEQGKIKLLTLED--HSPAGMQGFVYNIRRSQLSDPRVRQALAYAFDFEWSNTNLFYSSYQRTNSYFANS
ELAA----TDLPSQAELALLEPLR----EQVP------AEVFSQVYQAPTSDGS-GNI----RNNLRQALSL
LKEAGWTMEQ---------------------------------------------------------GKLVN
QASK--APFQFEILLYS---PAMERVATPFVRNLERLGIQVSLRMV-DVTQYVNRLR---SFDFDMVVAT-F
PQ----SNSP-GNEQREFWHSSKADQADSRNLIGIKNPAVDTLIEHIINAKDREA-LVAACRALD-RVLLWN
HYVIPQFNAG---------IFRIATWD-KFGHPDPLPE--YAPSPL-LETWW--------------------
----------------
>Oceanospirillum/1-571 beijerinckii
-------------------------------SHAASPQHGIAMHGD---LKYPADFAHFDYVNPNAPKGGVL
KQSSLG--------SFDSLNPFII-------KGVPAAGIG------------LIYDTLLEASSDE-------
--ALSKYGLLAKSVTRADDN----SWVEFELNPQAKFHDGHPVTAEDVIFSFTTLMRDG--RPLYRAYYADI
AEINQDGDYKVRFNFKTTENG-ELPLIIG-QIPILPKHYWQ-------DKDFTKTTLD-APLGSGPYTVGKI
DNGRSITYQRNENYWAKDLPLNIGRYNFDQIVYEYYRDGTVALEAFK-AGNLNFRREN--SSKFWATAY-DT
PAVQDGKIIKEEIKH--QNPTGMQAFAFNLRRDEFKNKQVREALNLAFDFEWTNKNLFYNAYTRTSSYFSNS
ELAS----SGLPSEAELALLNPFK----DQLD------PSIFTTPFKLTETKGD-GRN----RIELRQASRL
LTKAGWPIVD---------------------------------------------------------GKRVD
EN-G--KPVEIEMLLYD---TAFERIVHPFKKNLERLGIDLNIRIV-DTTQYINRVR---SFDFDMIVHS-I
GQ----SNSP-GNEQREFWHSEYANKTDSSNIMGVSNPVVDALVEKIITAKDRQT-LITATRALD-RVLLSG
HYVIPHWGIT---------AFRVAYWN-NLKHPEKMPP--YDLA---MDAWW--------------------
----------------
>Inmirania/1-599 thermothiophila
------------------MLWAAMLWASSPLAAAEP-VHALAMWG---QPKYGAGFTHFDYVDPNAPKGGDV
RLAEIG--------TFDSLNPFIL-------KGIAPAGIGM------------TFDTLTVASADE-------
--PFTRYGLVAESMELAPDR----SWIVFNLRPEARFHDGSPITAEDVVFTFETLRTRG--HPFYRAYYASV
DRAEVLGPRRVRFAFK-PGANRELPLIVA-ELPVLSKRDWQ-------GRDFTKTTLQ-PILGSGPYRVKTV
DPGRSVTYERVRDYWAKDLPVNRGRFNFDRIRIDYYRDTTVALEAFK-AGEYDFREEN--VAKNWATAY-DF
PALRQGLVKKVEIPH--RRPAGMQGFVFNTRRWMFRDRRVREALGYAFDFAWTNRNLFHGAYTRTTSYFANS
ELAA----TGTPGPEELAILARYR----GRVPE------EVFTRPYAPPPGDPQ-GDI----RGQLRTAMGL
LRAAGFDV--R-------------------------------------------------------DGRMVE
RASG--RPFRFEILLVN---PSFERIALPFARNLRRLGIEAGVRTV-DPTQYQNRLQ---QFDFDMTVAV-F
PQ----SLSP-GNEQRDFWTSAAADTPGSRNLAGIRDPVVDELVELLIAAPDRAS-LVARARALD-RVLLWG
HYVIPHWHL---------NYFRVAYWD-LFGRPPVN--PPYALAFD---TWWVDRAKAERIAAARTR-----
----------------
>Fluviicoccus/1-598 keumensis
-----------------LLFATLMSLTVTMGHAAIISSNAIALHG---KPTQPDNFKAFPYARPDAPKGGEL
RLRA-LG-------TFDSTNPFIN-------KGNPVEGTD------------YLYDSLTVAALDE-------
--PFARYGLLADRIDRDPEDP---SWITYHLNPAARFSDGSPVTADDVVFTFDLIRKEG--APNLRNYYNEI
SKVEALDKQTVKFSFKNKN-NRELSLIVG-EMSILPKQFWS-------KRTFNSTNLD-IPIGSGPYLLTKL
DPGKSATYTRNPKYWAANLPVNRGKFNFDTIKFVYYQDMTVAFEGFK-AGQYDFLTEF--KAKTWATEY-TF
PAVKQGLVKKVEQPN--FNPAGMQGFVYNLRRPLFQDRRVRQALSYAFDFEWSNRNLFFSAYSRSQSYFDNS
```

ELAA----TGLPSPAELALLNPLK----AQVPP---EVFT---TPFQAPKTDGS-GNV----RANLMAAQQL
LQSAGWTIKN---GKLVN
AKNE---PFKFEILLVQ---PEFVRIVEPFTRNLARLGIEVSIRPV-DSSQYINRER---NFDFDMVVEG-F
AQ----SLSP-GNEQRGFWGSQAADTPGSHNLAGLKSPAVDVLVDKIVGARSRED-LVTATRALD-RVLLWN
HLVIPQYHNR---------SYRLAYWD-FLEQPAIK--PKYSPG---IDFWWANAAKQARIRAA--------
----
>Spartinivicinus/1-607 ruber
-----------IIKFIWLLCIQQATLAAEAKNPAVHKAHAIAMHGQ---PKYPTNFTHLDYANPNASKGGAF
KQAVVSSS------GFDSLNPFIV-------KGVAAAGMAYLG-------GGYIYDTLLAQSYDE-------
--AFTMYGLLAESMEWPEDR----SWITFNLRKNAKFHDGHPITAEDVVFTFNLLIEKG--HPLYKTYYHNV
KTVTALNNYSIKFEFDTKTNR-ELILIVG-QFPILPKHYWE-------THDFTKTTLE-FPLGSGPYKVSKV
DPGRSIHYERAKDYWGQNVAVNKGHNNFDTLMFDYYREPSVAIEAIK-AGKYDLRIEN--SAKSWATEY-NI
SAVKEGRLIKASITD--YSPAAMQSFIFNIRHGKFKDPKVREAIGYAYDFEWQNKTIFYDSYTRIDSYFAGS
ELAA----TGLPSKAELALLEPFK----NQLP------NELFTKPFTLPKTDGS-GNV----RNNLRQALRL
LKQAGWQIRN---KQLIN
AKG---EPLTFEILLTQ---ANIERIVLPFQKNLKRMGIETTIRKI-DTQQYIERRN---NFDFDMIIGG-F
PQ----SNSP-GNEQRDYWHSSQANIKGSRNYIGIENPVVDQLISKIIQAPDRKT-LIASTQALD-RVLLWN
HYVIPQYYLN---------QSRLVYWN-KFGMPKQPPK--YTVG---LNTWWYDPTKAKKL-----------
----
>Desulforegula/1-602 conservatrix
-----------------IILCISLLWAASAYSAETHTTHALSLGNP---PKYPSDFKHFDYVNPDAPKAGHY
RAEANG--------TFDSFNPFII-------KGAAAAGVD------------LLFDTLTESADDE-------
--VFARYGLIAEKMEMPSDK----SWIIFHINPKARFSDGTQITAEDVEFTFKLLVSKG--APMYKQYYRDV
VKAETLDEKRIKFSFKDGKNP-ELPLILG-QLTVLPKHFWE-------SRDFSKGGLE-IPVGSGPYLIDKF
IPGKSVTYKRNPEYWAKDHPACKGRYNFEKISYEYFRDDTVSLEAFK-AGKYDFITES--SAKNWATQY-TG
KFFDNGVIKKELIPH--EMPQGMQGFVFNTRRDFFSDRKVREALTQALDFEWSNKALFYGQYKRSSSYFSNS
ELAS----SGLPSEEELKLLSPFR----DELP------DDLFSKPFKLPVSGDR-DKL----RENLKKADKL
LNEAGFIVKD---MKRIN
KETD--QPFRFEILIHQ---KNFERVCLPFKRNLERLGIKVSIRLV-DTAQYINRVN---SFDFDMIVGG-I
GQ----SLSP-GNEQLEFWHSSAADSPGSRNMAGIKNPAVDSIVEKLITAKDRKT-LITTCQALD-RVLLWN
YYMIPQWHLP---------AIRVAYVD-KFERKEPLP---KYNSVS-IMNWWINEEKENRINSLLKR-----
----
>Alginatibacterium/1-590 sediminis
--------------MLRIITLIWIVFSVNAQAQDYVRSHAIAMHGA---PQYSADFEHFAYVNPNAPKQGDV
TFASIG--------TFDTFNGFVV-------KGTPAAGVG------------LVYDSLMTSNSDE-------
--PFTYYGLLAQTIDLANDN----TWVRFNLHPDAKFQDGMPVTAQDVEFSFNTLLENG--TPFYRSYYGGI
SSIEVESDRSIRFNFENGDNR-ELPLILG-QLLILPKHFWQ-------ERDFTKADLT-IPMGSGAYTLKSF
DAGRSVTYELDPNYWGAKLPVNLGQNNIKTLNYEYYLDSSVALEAFK-AGRIDFRLEN--SSKNWATAY-TG
PQFEDGRVIREEIAN--LNPQGMQGYVFNTRKDKFSDIRVREAISLLFDFEWTNDQLFYGAYKRTNSFFAAS
ELAS----SGLPEGRELKLLEAYQ----DQLP------EQLFTQPFEVNRTSGD-GTI----RKEMREALRL
FKEAGWALDK---GKLLN
AE-G--EQFEIEFLIRD---KSSERIVLPFTRNLQKVGIKTEVRLV-DISQFINRIQ---SYDFDMMTYV-F
AQ----SNSP-GNEQREFWGSLVADKPGSRNLIGIKSPVVDALIEELIHSDDREQ-LVQATRALD-RVLLWG
HYVVPQFHLN---------TWRVAYWG-KLKRPENSAK--YNIE---LNSWWVE------------------
----
>Stutzerimonas/1-571 nosocomialis
---------------------------------AQAAPQHALTLYGE--KPKYPANFQHFDYAHPDAPKGGTL
RRSGSG--------GFDSLNPFIN-------KGVPADEIG------------LIYDTLTTNSLDE-------
--PFTVYGLLAEKIERAPDN----SWVRFHLRPEARFHDGEPVTAEDVVFTFETLISQG--APWYRAYYADV
AKVTAESPRRVRFDFKHAGNR-ELPLILG-QLLVLPKHWWA-------ERDFNSSGLE-PPLGSGPYRIERV
QAGRSVRYQRVEDYWGKDLPVNRGFYNFDRITFDYYRDNDVALQAFK-AGQFDFWLET--SAKNWATAY-DI
PAVRDGRIVKEEIEN--HNPTGMQGFILNTRRPLLHDRRVREALGLLFDFEWTNRQLFNGAYTRTTSYFDNS
EMAA----TGLPDADELKILEPLR----GQIP------DEVFERPFELPRTEAN-GII----RDQQRQAYKL
LMDAGWKIEN---DRMVD
TE-G--KPVKLEFLLVQ---AEFERVLLPYKRNLADLGIELEIRRV-DVSQYINRLR---SRDYDMIVSS-F
GQ----SNSP-GNEQREYWHSSSADNPGSRNLIGLKDPAIDVLVDKLIAADSRKE-LVTRTRALD-RVLLWG
HYVIPNWHIK---------TWRVAYWN-QFGHPEVTPD--QDIG---LMTWW--------------------
----

```
>Entomomonas/1-584 asaccharolytica
-------------------LLIILIVSFICPLTQAKPQHAMTMYGE--PSKYPADFKHFAYVNPDAPKGGTL
RLASYG--------GFDSFNGFIP-------KGQVADYVG------------LIYDTLTIQSYDE-------
--PNTIYGLVAEKIDKAPDS----SWVRFYLRKEAKFHDGHPITAEDVAFTFDILKSDG--RPFFRQYYADV
SKVIVENKHQILFRFKVKNNR-ELPMIIG-QLPILPKHWWQ-------DKDFTVTNLI-PPLGSGPYKIGKV
KANASIEYERVEDWWAKDLAVVKGFYNFDKIIIDYYRDMSVALEAFK-AGQFDFNLEY--SAKSWAIGY-EG
TALKEGKLIKKELTN--HNTANIQALAFNLRRPIFQDKKVRQAISLLFDFEWSNRQLFYNSYKRISSYFENS
EMAA----HELPTEKELTILEPFR----GQVP------DEVFTTVFTAPQSDGS-GII----REQQRKAYQL
LQEAGYEIKN---------------------------------------------------------GKMVD
KE-G--QQLKFEFLMHQ---ANLERVLLPFKNNLAEIGIDMEIRRV-DTSQFINRIR---SHDFDMVSYI-W
GQ----STSP-GNEQRNYWSSSSADVVGSQNILGLKDPVVDKLVEQLIQTDSRES-LILHARALD-RVLQWG
YYVVWNYYTD---------KWRIAYWN-KFGQPEIAPD--YDYG---LFTWW--------------------
----------------
>Plastoroseomonas/1-584 arctica
---------------------------------GITRTHALSLLG---EPALPRDFRHWPWVNPNAPKGGEI
VLTALG--------SYDSLNSFIL-------RGTAAVG-LGN-----------LYDTLLKESSDE-------
--GSSEYAHLAAEIELPADR----LWVAFTLHEQARWHDGRPITAEDVVWTFNTLRTAG--RPTYRGYWADV
RECVAEGPRRVVFRFT-SAENRELPLILG-QLPVLPKHFWE-------GRDFSRPLLE-APLGSGPYRVERF
ETGRGIVYQRVADYWGRDLPTMRGTNNWDRIRYEYYRDTTVAFEAFK-AGQIDFRQEN--SARDWATGY-DF
PAIRRGLVKREEIRH--EIPTGMQAFAMNHRRPLFQDARVRRAMIEVFDFEWMNTNLFFGSYTRTASYFSNS
DFAS----SGIPSGAELAVLEPFR----AQLPP------ALFTEEYRLPVTDGS-GNN----REGLRRALAL
LREAGWTVR---------------------------------------------------------DRRLIN
AQG---QPFEFEILIQG---ATFERIALPYVQALERLGVTARVRTV-DPAQYQVRID---AFDYDMTVDS-I
GQ----SLSP-GNEQRDFFTCEKARENGSQNVTGICTPVVDALVDQVVNAPDRDA-LVARTRALD-RVLLWG
NHMIPQWHM---------RSFRIAWWD-KFGKPARN--PKYGIGL---DSWWVEAQRVGTVEEGRRQ-----
----------------
>Arenibaculum/1-591 pallidiluteum
----------------------------GHAAGAVENAAGLALHG---EPKYKPGFAHFDYVNPQAPKGGEI
KLAAMG--------GFDNLHPFIL-------RGIAPAG-IGL-----------VFETLMTQNADE-------
--PFSMYGSVAESISVPADR----SSTTFALRPEARWHDGRPITAEDVIWTFETLMREG--SPIYRTYYGDV
VRAEKVGDRQVRFTFK-SGGNAELPLILG-QLPVLPKHWWE-------GRDFGATTLE-PTLGSGPYRVAQV
EPGRAITYERVADWWGKDLPINRGRWNFDRIRYDYYRDRGVLLEAFK-AGAYDFRLES--TAKDWATGY-DI
PAVRDGRIVREEIRT--EDPRGMQGFVLNTRRPLFQDRRVRAALGELFDFEWLNKNIFYDQYKRTRSYFSNS
ELAS----SGLPSPAELKILEPYR----GKVPD------EVFTTEYKPSETDGS-GNI----RANLRTALDL
FKEAGWTLR---------------------------------------------------------NGALLN
AAG---QQMQFEILLDD---PALERIVQPWMRNLERAGIKPVLRIV-DTAQYKNRTD---AYDFDVTTEV-I
GQ----SLSP-GNEQRDLWSSAAADVPGGQNLAGIRDPAIDDLIERLIRAESRED-LVNHTRALD-RLLLWG
HYVIPQYHS---------DKNNVAYWN-RFGHPAQP--PRYGIGFP--DTWWIDPAKDARLASLDRGG----
----------------
>Insolitispirillum/1-566 peregrinum
--------------------------------------HALTLHG---SPKYGPDFKHFAYVNPDAPKGGEL
KLAAMG--------SYDTFNSFTI-------KGTPPAGIGM------------VYETLLTPAADE-------
--AFTEYGLLAESVTLAPDN----TAVTFTLRPQAKFSDGKPVTPEDVIWSFNTLKEKG--RPFYAFYYGDV
KEVSKSGPRQVTFTFK-DGSNRELPLTLG-EMPILPKHFWD-------GKDFSASTLE-PPVGSGPYRLKSF
EAGRYVVYERVADWWAKDLPVTRGLYNFDTIRYDYYRDTTVALEAFK-AGAYDVRPEN--VAKLWATGY-DF
PAVKDGKVLKLDVPH--KMPSGMQGFAYNLRRPLFQDDRVRQALALAFDFEWSNQNLFYGQYLRTVSYFDNS
ELAA----TGLPGKDELALLEPLR----KDLPP------DVFTTVYQPPSTAGD-NGL----RVNLRQAMEL
LKAAGWSVK---------------------------------------------------------DGVLTN
AQ-G--QPFRFEILLSD---PTWERIALPFAQNLKRLGIEASVRTV-DSAQYQSRMD---TFDYDMTVEI-W
GQ----SLSP-GNEQRDFWGCAAAKQDGSRNTTGICSPAIDALIEKVVQAKDRNE-LITATRALD-RVLLWS
HQVIPHWHI---------SSSRVALWN-RFGMPAVS--PMQGMQLM---AWWAD------------------
----------------
>Limimonas/1-613 halophila
------MLRCLVTTLILLVMTVAGPALAADTYNGVPLRHGVAMHG---EVKYPADFEHFAYANPDAPQGGKI
KLAAQG--------TYDSFNPYIV-------QGVSVQGAQL------------MYDTLMVSTDDE-------
--PFSEYGLVAEGIYMPEDR----SWVAFKLREGARFHDGEPITPEDVLFSLNVLREKG--HPFYRFYYKSV
AKARKVGPRTVRFDFA-GGTNRELPLILG-QLPVLPKHYWA-------ERNFKESTLK-PPVGSGPYRVESF
EPGRFVVYEKVDDYWAADLPVTRGRWNFAKVRYDYFRDPTVIRTAVK-GGSIDFHLEN--QAKAWATAY-DT
```

```
PAVQSGQLERVEIEH--DTPTGMQGFAMNTRRSPFDDPKVRRAMSYAFDFPWTNQNLFFGQYERTDSYFSNS
ELAAG---DGPPDGRVRDILKRFE----DELPD------SVFTQRYDPPTTDGD-GYP----RENLKKALKL
LNEAGWVVR---------------------------------------------------------DMQLVN
AETG--RPMRFEILLVN---TSFERIVLPYVANLEKLGIEVEVRVV-DSAQYQNRLD---NYDFDMIVAN-W
GQ----SLSP-GNEQREFWGSTAAERPGSRNYTGVADPVVDKLIGMVIGAPSREA-LVQRVRALD-RVLLSK
HLVVPHWHI---------GHYRVVYWD-KFGRPESNP-PYGLPYLS---TWWVQPDKARDVASAQQ------
----------------
>Stella/1-578 humosa
-----------------------------------KPRHGMSMYG---ELKYPPDFKHFEYANPAAPKGGTV
RLAAIG--------TYDTLNPFTL-------KGVPAAGAGL------------LFQTLTANSGDE-------
--AFSEYGIVAESFIVPEDR----SWVAFNLRPEAKFNDGTPITPDDLIFTLETLKTKG--APFYRTYYADV
AKGEVTGPRQVRFTFR-AADNPELPLIVG-QMPILPRAYWA-------NREFDRTTLE-APLGSGPYVVESV
DAGRSITYKRIENHWSDQVPALKGQYNFARIRYDYYRDSTVALEAFK-GGEYDFRQEN--VAKDWATGY-DA
PAVTEGLIKREEIAN--EQPTGMQGFVFNIRRPIFADRRVRDAIAHAFDFEWTNKTLFYGAYARTRSYFSNS
ELASS---G-LPGPAELAILEPFR----GQVPD------EVFTKEYVPPSTDGS-GNL----RDNLRVAFDQ
LKAAGWVIK---------------------------------------------------------DRRLVH
EKTG--EAMSFEILLSQ---PTWERIALPFAKNLERLGIQARVRTV-DSAQYQKRME---TFDYDVTVEV-F
GQ----SLSP-GNEQRDMWSSAAAKVEGSRNTIGVADPVVDKLIELVINAPDRQA-LVDRTRALD-RVLLWG
HYAIPNWHS---------RTWRIAYWD-RFARPPVT--AKYSLGFLA--TWWIDPAKESAL-----------
----------------
>Pararhodospirillum/1-573 oryzae
--------------------------------------HGLAMHG---AVKYPADFTHFDYVNPDAPKGGAI
RLAAMG--------GFDTLNVFTI-------RGEAAAGLGL------------MVDTLTARAADE-------
--PFSHYGLIAQSMEMPEDR----SWIIFNLRPEAVWSDGEPITADDVAFSLETLRTQG--IPMYNMYYGDV
ERVEVLGPKRVRFVFK-GTENRELPLIVG-QMPILPKHYWE-------GRDFSAVTLD-PPVISGPYRIASF
EPGRSITYARRPDYWAKDLPVSRGFYNFDEIRHDYYRDATVMLEAFK-AGAYDFRAEN--VARQWATGY-DG
PAVRDGRIIMERLPH--HRVQGMQGFIFNLRRPLFADPRVREALGWAFDFEWTNQNLFYGEYTRTRSYFDNS
ELAA----TGLPDAAELAVLEPWR----DQLPP------RVFTEVYAPPVVDEAKGGL----RANLAHALDL
LRQAGWTVK---------------------------------------------------------DGALRN
AD-G--QPFEVEVLLAQ---PEFERLVLPFARTLERLGIRLSLRTV-DTAQYINRVT---HFDFDIMVGS-W
GQ----SSSP-GNEQRDFWSQAAAHTPGSSNYSGIASPAIDGLIESIIAAPSRAD-LVTRVHALD-RVLQWS
FLVIPQWHQ---------SSDRVAYWN-LFGHPP-T--PDDGIQFN---TWWVDPAKAAAL-----------
----------------
>Perlucidibaca/1-604 piscinae
-------------LAVVVLLLAQPVQAAG-EQPRHISHHALSLHG---SPALPAGFRHFPYANPQAPKGGLL
RLDA-LG-------TFDSLNPFIN-------RGVAADGLS------------RIYDTLTVSSEDE-------
--PYSRYGLLAERIEEDPQDG---SWIIYHLRPQARFHDGRAVTSRDVVFSFETIQRVG--SPAYKAYFAGV
ARVEALTPHSVKFHFRQRD-NRELALIVG-ELAILPAHYWR-------TRNFDSTSLE-PPLGSGPYRISKV
DPGRGIHYERVPDYWGRDLPVNRGLHNFDRIQYRYYRDGSIAFEGFK-AGQYDIRHEN--KAKTWATEY-NF
PAVQKGQVLRIEQRH--QRPAPAQGFFFNTRRKPLDDRRVREALAQGFDFEWANPRLFYNAYARTRSYYDNS
ELAA----QGLPGQAELRILSPWR----TQLPA---TVFG---PAVLPPVSQGD-GYN----RRNLLKAQTL
LQQAGWRYRD---------------------------------------------------------GALRN
AAGQ---PLVLEMLLVQ---PEFERIVQPLRRQLARLGVTLNIRIL-DAAQYVERLR---QFDFDLTVSG-V
PA----SISP-GNELWNYWSSDAARTPGSQNLTGLQSPVVDALIAGITQARSRAD-LVDQVRSLD-RVLRAE
WLMIPNYHTP---------FFRIAAWD-RFQRPALT--PRYGDG---LDSWWFDAAKARRLDAATGK-----
----------------
>Tistlia/1-571 consotensis
---------------------------------VVIRAHAIAMHG---EPKYGPGFKHFDYVNPDAPKGGEI
RLAAEG--------TFDSFNPYND-------KGNPAGV---------------AYETLLTPSADE-------
--AFTEYGLIAESLEWPLDR----SWVIFHLRPEAHWHDGEPITVEDVIYSLETLKAKG--QPFYRYYYRAV
QKAEKVGPRSVKFTFN-EKGNRELPLIVG-QMPIIPKHYWE-------SRDFGATTLE-PPLTSGPYKITKF
EPGRYYVQERVKDYWGKDLPVRVGTENFDRIRTDFFLDATVIREALK-SGSIDYREEN--QAKAWALDY-DV
PAVRNGWLIKAEVPN--QRPTGMQAFIFNTRRPLFQDPRVREALGYAFDFEWTNKVLFFGQYTRTRSYFSNS
ELAS----RGLPEGQELEVLDRFR----DQLPA------EVFDKPFTVPETDGT-GWP----RANLLKALAL
LKQAGWVVDPK-------------------------------------------------------SLKLVN
EKTR--QPFRFEILLTQ---QAFERIVLPFTRNLKRLGIDANVRLV-DQSQYINRLR---SFDYDMISLG-W
GE----SDSP-GNEQRNYWTSESAKRQGSQNYAGIESPVVDKLVDLVINAPDRES-LVARTRALD-RVLLWG
QYVIPAWHL---------AMDRILYWD-KYSRPEVT--PKDGTAID---YWWYD------------------
```

>Pokkaliibacter/1-578 plantistimulans
--------------------------------------HAWAMYGA---PKYPADFRHFAYANPDAPKGGTL
RRSAMG--------TFDSLNSFIS-------KGTPAAAST------------LLYDTLMVHSQDE-------
--TSSIYGLIAQTITVPPER----TWVEFGINPKARFQDGQPITAADVKFSFETLINKG--SPLYAVYYHDV
TAVDIKDPLTIRFTFRDGNNR-ELPLILG-QLPIFPAHYWQ-------DKDFAAANLT-VPVGSGAYTVEKI
IPGRTIVYKRLDNYWAKDLPSRKGIDNFDHLSYDYYRDGDVALEAFK-AGEFDLRQEN--SASRWVTRY-DI
PAVHDGQILKLSIPD--HNPSGMQGYIYNTRRELFQNIKVREALAYAYDFEWANKNLSYGGLTRTRSYFMNS
EMES----TGTPSADELSLLEPLK----DEIP------ARVFSEQYNPPQTDGS-GNI----RPQLRQALRI
LREGGWEIHG---NRLLS
DKTG--EQMRFELLIYSD--PGMERTVLPYIRNLRRLGIDVEIRRV-DVTQFINRLR---SFDFDMTVAV-I
PM----SNSP-GNELRDYFNSANANVPDSSNLAGISNPAVDTLVEKIINAPSKEM-FVAACQALD-RVLLWN
FYLVPQFTIP---------DTHIAVWD-KFGRPSTLPL--YSPG---IDTWWAKSAEHAASSQG--------
----
>Azomonas/1-588 agilis
---------------------MLLISLGSMQKANAAPQHALTLYGE--PPKYPENFSHFEYVNPDAPKGGVM
RQAGIG--------SFNTLNPFIN-------KGVAAENIE------------LLYDTLTKNSQDE-------
--AFTVYGLLAERIEKAPNN----RWVRFYLRPEAKFHDGQPVTAEDVAFSFEILTTQG--APQYRAYYADV
ERVVVESKSRVRFEFKHGGNR-ELPLILG-QLPVLPKHWWE-------NREFNSGSME-PPLGSGPYRVERV
DPGRSIHYARVPDYWGKDLAVNRGLDNFDRLIFDYYLDTDIALQAFK-AGKFDYWLEI--MAKNWATAY-DI
DAVRSGKIKREELPN--HNPSGMQGFIFNTRRPQLQDRRVREALSLLFDFEWANQRLFNNAYTRTISYFDNS
ELAS----SGLPSPEELKLLEPLR----AQIP------PEVFEQPFVLPVTQGN-GII----RDQQRRAYQL
LTQAGYRVEN---DQMVN
AQ-H--QPLVLEFLLTQ---AGFERILLPYKRNLADLGIELNIRRV-DISQYINRLR---SRDYDMIVSG-F
SQ----SNSP-GNEQREYWHSSTADNPGSRNFIGLKSPAIDQLVDQLIQSDSRAE-LVTRTRALD-RVLLWG
YYVIPNWHIK---------TWRVAYWN-HLDRPAVTPL--YDIG---LRGWWYKASPA--------------
----
>Roseospirillum/1-581 parvum
----------------------------------IPAVHALAMHG---APRYGPDFTHFDYVVPTAPKGGEA
RLSAIG--------TFDTLNPFTL-------KGVPAAG-LGG-----------TFDTLLERSDDE-------
--PFTMYGLLAESIRLPEDR----AWVEFTLRPEARWHDGQPVTAEDVAWTFKTLTSEG--SPFYAAYYADV
TEVSAPAPNTVRFTFTPGQINPELPLIIG-QLPVLPRHAWE-------GRDFAQTTLE-PLLGSGPYKVAEV
DPGRAITYQRVADYWAAGLPLKRGRHNFDRLRYDYYRDAAVALEAFK-AGAYDFRQEN--TAKVWATGY-DF
PARDQGLVKLEAIEH--SLPTGMQGFFYNTRRAVFADPRVRRALAYAFDFEWTNANLFHGQYTRTHSYFSNS
ELAA----QGAPQGRELEILESFR----GRVPD------SVFGPALKAPDTDGP-GGL----RDNLKQAVDL
LKEAGWAIG---KDRKLR
AEGGQGAPLSFEILLYN---PAFERISEPFARNLERLGIEARLRVV-DTAQYQARVN---DYDFDMIVYS-A
GQ----SNSP-GNEQRDYWSSAAAGKPGSRNVAGLADPAVDGLIDLVITAPDRAE-LVARTRALD-RVLRAH
YLCIPHWHS---------TQFRVAYWD-KFGHPDNP--PPYALPI---DTWWIDPDKAARL-----------
----
>Geminicoccus/1-594 harenae
--------------------------FRFARAADAVPAHGLSMYG---ELHYPPGFTHFDYVNPDAPKGGNL
VLSAVGT-------TFDTLNPFV-------LRGVPASGVG------------LVYQTLAENSQDE-------
--PFAEYGLIAETIATPEDR----SFVEYRLRENARWHDGRPVTVDDVVFSFDILRSKG--APLYRVYYADV
ERTEKIDERHVRFLFKGSF-NRELPLIMG-QLPVLPAHWWQE-------RDFEAPSLE-PPLGSGPYRVDRV
ESGRSVSYALVEDWWAKDVPVMKGRFNYGRIRYDYYRDANIAFEAFK-AGAFDWQIES--SAQRWVTGY-DI
PAVRQGLLIKEEIKR--ESGGVIQGFWLNQRRDQFKDRRVREALGYAFDFEWSNRTLFYDQYTRCNSYFSGS
SVFAA---TGLPEGAELALLEPYR----DQLPP------ELFSQEFTLPVTDGS-GNN----RPQLRRALEL
LKEAGWEVQN---RRMVE
VATG--RPMQFEILLD--S-PLFERIAGPFVKNLERLGIAATVRTV-DSAQYQNRVT---AFDYDVIAQI-I
GQ----SLSP-GNEQREYWGSQAATQEGSRNYAGISDPVVDAMIEKVIYANSREE-LETACRAMD-RVLLWG
YHLIPHWYA---------MYDRLARWD-KFGRPE--QMPRYSID--LFS-WWIENQRATKVEQAKSE-----
----
>Rhodomicrobium/1-596 lacus
-------------------LLTTAALLFAEGAFAAERTHGLSSFG---TLKYPADFKHFDYVNPDAPKGGRL
ATMP-----TSSINTFNHFNAYIL-------RGDAAEGVSLL------------FDSLMARA---------M
DEPDAVYGLVAHAAEVADDR----LSATFYLRPEARFSDGSALTAEDVVFTLDKLKKDG-HP-RYQMLLRDV
VSATAVDPQTVRYTFKGENLRDLPLTVA--QLPIFSKAFYTAN-------DFLKESLD-PPLGSGPYQVGEF

RQGTFVSYNRRKDYWAADLPVNRGRYNFDTIRFEYYRDRAVGLEAFK-ARAYDLREEF--TSKSWATEYD-V
PAVREGKIVKLTLPD--GRPSGAQGFFLNMRREKFADSRVRKALDYAFDFEWTNKALFFGLYTRTASYFENS
-DLKAE---GKPTDAELALLEPF----RDKLPPE------VFGEPYTPPVTDGS---GR--FRPSIREASRL
LDEAGWK-L---DGGV
RKNAKGEILEFEFLIDD---PVTERIVGPYAGRLSDLGVKATLRRV-DPTQEQERIRR---YDFDISTQ---
--RYSLSQTPGPE-VRAFWNSEAGKEDGSYNLSGIADPVVDVLIDKVLAAKSRDE-LRTACRALD-RVLRAG
HFWVPQWYKA---------AHNIAMWD-KFARP--AVKPAYDP--AILDTWWYDAEKAAKL-----------
----
>Oleiphilus/1-578 messinensis
-----------------------------VAAKEVAAVHGLAMHGT---PKYGADFTHFEFVNPNAPKGGTL
TRETIGD-------NFDTFNPFLV-------KGVPAAGVG------------LMFDTLTKHSGDE-------
--AFTEYGLIAEKIQMPEDR----SWVIFHINPKAKFSDGQPITAEDVKFTFEMLTTSDKVQPFYKTYYGDV
TEVQILDPLRIKFQFKNDQNK-ELPLILG-QLNILPKHFWA-------TRNFEKADLT-VPVGSGPYLLESY
EAGRRVVWKRNPDYWAKNLPVNKGFYNFDEIIYEYYKDRTVALEAFK-AGEFDFTVEN--TARNWANAY-VG
PKFDSGELVKEEIHH--KRPAGMQAFVFNTRRTKFDNPQVRRALAYAFDFEWTNQNLFYSQYKRTNSYFENS
ELAS----RGLPSAAELEILQPYK----SQLP------EEVFTKAYQAPTTAAP-SNL----RKNLREGLKI
LKAAGWNIVN---GTLVN
GKTG--QPLKFEIMLYS---KDFERIVQPFIQNLKKMGVQASIRLV-DTTQYINRIR---EFDFDMLVYT-I
PQ----SNSP-GNEQRDFWYSGNADVQGSRNIPGVKDPVVDALIEKVISATDRED-LIAHTRALD-RVLLWN
HYVIPNWHNP---------SARVAYKA-NLMRPAITP---DSG-AD-LMTWWF-------------------
----
>Terasakiispira/1-571 papahanaumokuakeensis
-------------------------------SEQVQCQGAMALHGE---PALPEDFKHLPYVNPDAPKGGRW
RQAAIG--------TFDSLNPFII-------KGTPAAGTSTL-----------LYDSLTVQSLDE-------
--PFTEYGLVAKCMRLDPEN----HWIEFKLRPEARFNDGQPITAEDVIFSFNILKEQG--RPFFRAYYHDV
AQVSAPDDHTVRFEFANTTNN-ELPMILG-QLPVLPQHYWQ-------DKDFTAPGLD-VPIGSGPYRISHV
DPGRKITYERVKDYWAKDLPINVGRFNVDTQSFDYYRDQGVALEAFK-AGRLDFRLEN--SASQWAEAY-NG
TALEQGDIVKEKIRH--HNPAQMQGFVYNTRRAPFDDPKVRAALLNAFDFEWTNKQLFHGAYQRLHSYFDNS
ELAS----QGIPKGAELALLTPWK----DQLP------EALFKEPYQLPVTQGD-GNI----RRQLRDALRQ
LKSAGWTMQG---GKLVK
N--G--QPFEFEMLLVS---SQFERIVLPMKRNLQRMGIDMKVRLV-DTTQYLNRLR---DFDFDMTVGS-F
PQ----SLSP-GNEQRDFWGSESAERPDSRNIAGVSSPVVDDLINKVIHADSREQ-LVTRVHALD-RVLLWG
NYMIPQFYLD---------SYRIARSQ-KLQHPEQIPR--YGID---LDSWW--------------------
----
>Wolinella/1-597 succinogenes
-----------------VLWLFLCAGGLEFAFASTFSDKGFSLSGE---VKY-KELKYFDYVNPEAPKGGAI
KRYEIG--------GFDTLNAFAL-------KGTPADGLE------------LLYDTLTVHSEDE-------
--PFSEYGLVAERIQRAKDN----SFVIFHLNKNARFHDGNPITAFDVEFSFNTLIGTG--NPAIKRYYEDV
KEVVVVDKYTVKFNFSNKENR-ELPLILG-QLRILPKHFYE-------NRPFGENPLE-IPLGSGPYRILSF
ETGKEIVYERVKDYWAQKHPTRLGYFNFDRVAYEYYKDETVALEAFK-AGAYDFRQES--AAKTWALGY-EG
EPLKKKQIIKEEIAH--SLPSGMQGYFFNTRRDLFKDIRVREALSYAFDFEWSNKNLFFGQYTRTKSFFDNS
ELAS----FGTPSPQEREWLTPFK----EQLP------EGIFDQPFTLPTTKGD-GNI----RPQLKRAQQL
LKEAGYEIKN---KKLIH
TTTG--QPFVFELLLLS---PAMERVALPFKRNLATLGIEMKIRTI-DLTQYINRLR---EFDYDMIVGV-I
GQ----SLSP-GNEQRYYWHSSSKDERGSKNYAGIDHPAVDRLVEMVVNAKDRRE-LVDYTRALD-RVLLWN
HYVIPHFHNR---------TFRVAHWN-RFSRPNISP---LY-GLG-FWTWWVDPQKEEELLKA--------
----
>Roseomonas/1-585 harenae
---------------------------------APRRVHALSLLG---EPALPADFTHFPWVNPNAPKGGEI
TLYALG--------SFDSFNAYIL-------RGTPAVG-SGL-----------LYDTLLKESADE-------
--ASAEYGHLAGMVELPADG----RGVTFELREGARWHDGRPITADDVVWTFNTLRQHG--RPFYRAYWADV
SEVVAESPRRVTFRFS-TDQNRELSLILG-QLAVLPKHWWE-------GRDFARPLLD-PPLGSGPYRIERF
EAGRSILYRRVEDYWGRDLPTMKGTNNFDVMRYEYFRDATVSLEAFK-AGQIEFRTEN--VARDWATAY-DF
PAVRRGWVKRDEIRH--ELPTGLQGFIMNERRPLFQDRRVREALGLVFDFEWMNANLFYNSYARTSSYFSNS
DFAA----RGLPEGREKEILERFK----DRLPP------EVLTREFKLPVNDGS-GNN----REQARRALEL
LREAGWTVR---DRRLTN
AQG---QRFEFEILLNG---PTFERVALPYIQSLRRLGIEARVRTV-DPPQYQVRMD---GFDYDMTVDV-F
GQ----SSSP-GNEQRDYWTSAKADENGSRNTIGIKDPVVDEIVEMIVNAQDIQE-LTASCRALD-RVLLWG

FHTIPQWHS---------RTFRLAWWD-KFGRPERN--PKRALGL---DSWWVDPAKERALAAARSAG----
----
>Caenispirillum/1-575 salinarum
---------------------------------AVEPVVGIAMHG---EPRYTEGFAHFDYVNPNAPKGGEM
KLFTTG--------TWDSFNAYIA-------KGTSVAGIGM------------IYDTLLTPSDDE-------
--PFSEYARVAEAVRMPEDR----SWVAFDIDPDARFHDGEPIKPEDVIWTFNFLVEDG--DPLYRFYYGNV
KDVVKTGDRQVTFLFQEGSENRELPLIVG-QLPVLPKHYWTAE-----GRDPTATTLE-PPLGSGPYRVAGF
EAGRYVVYERVTDYWGKDLPVNVGRNNAGRIRYDYYRDTTVALEAFK-AGAYDIRMEN--SAKNWSVGY-QF
PAFEQGLVKKATFDH--DLPAGMQGWVYNMRRDIFADPLVREALAYAFNFEWANQTIFYDQYARTRSYFDNS
ELAA----TGLPSQEELEILEPLR----GEIPE------RVFTESYTPPMGDAS-GNI----RENLRAAFLL
LQEAGWQVDPK---TRMLVD
PETD--EPFSFEILLVS---PEAERYTLPFVQNLKTLGIDARVRTV-DTAQYINRLR---SFDFDMTVVV-W
GQ----SLSP-GNEQREYWSSAAAEHTGSRNYAGLQSDAVDKLVELVIQAPDRKS-LITRVHALD-RVLQWN
FLVIPHFHA---------PYQRVAYWD-KFGFTDAR--PMRGIDLM---AWW--------------------
----
>Oleidesulfovibrio/1-599 alaskensis
----------------CMMAVCALWLMQAGAAAAQPLTVLTMDG---EPRFAWNGRHFPHADPDAPKGGTL
RLSARG--------NFDSMHAFIA-------RGLPAAGIG------------LTVETLGTGAPDN-------
-TLFEYYGLIAQSFEVAPDF----SHVTFNINPAARFHDGRPVTAHDVAVTFRLLMEHG--APRYKQYYAAV
DRAEELSPLSVRFYLK-EKNNKELPVILA-QLPVLPAHYWQ-------NHDFSQPSLV-PAVGSGPYRVKDF
AMGSYVEYELVDDYWARDLPVNKGRYNFGTIRYEYYRDETVAREAFK-AGEFDLYPEG--TAKAWVSAY-TG
PAVAAGHIRREELTT--NRPMGMYGFFFNTRKDLFQDRRVRQALALLFDFEWTNKAIFHGSYTRSTSFFANS
ELAS----SGKPSQAELDVLRPFA----GQLPP------EVLTSAWDVPRTAGD-GNI----RPQMRQALAL
LQQAGWTLQDG---RLR
DAAG--RPFEFTLLLQS---AGSTRVVLPYRRNLERLGIIMNVAMS-DPTQYVNRVR---SFDYDMIMGR-V
PQ----SASP-GNEQRSYWTSASAGTPGSRNYAGVRSAVVDALVERLIASPDRDS-LVVNCRALD-RVLLWG
AYVIPGWYS---------SSVRIAYWD-KFGRSETPPATGFDVH-----SWWVDKEAEKALRNAG-------
----
>Thioflexithrix/1-582 psekupsensis
--------------------LALPSYAEEN--VSIHRSHGLAMYE---DLKYPADFKHFDYVNPNAPKGGLL
RLSATG--------TFDSLNPFII-------KGLSASGLSGW-----------FFETLTSSSKDE-------
--PFSEYGLIAETIETPEDR----SWVAYILRDKARFHDGSPITVEDVIFSFEILKTKG--HPFYRSYYNHV
TEVIKVNDNTVKFVFN-TNKNRELPLIMG-QLPILSKAYWE-------QRDFDKTTLD-IPISSGPYKIAAI
DPGRSITYQRNPEYWGKDLPVNLGQYNFDQIRIDYYRDETVELQAFK-AGEYDFRAEN--SAKNWATAY-DF
PALTQGRVIKEDIEH--QIPTGMQGFVFNTRRDVFKDPKVREAISYLFDFEWANKNLFNGAYTRTHSYFSNS
DLASP---SELPHEAELAILEPLR----AHLPE------RVFTEIYQPPQTDGT-GNI----REQLRLAIRL
LKEAGWELK---NGKLLN
EK-G--QLLQFEILLVS---PLFERIVLPFKRNLARVGIEVSVRTV-DTTQYQNRVD---NFDFDMIVHV-F
SQ----SLSP-GNEQHSYWHSENANVVGSRNLAGIQNPAIDQLVDLVIGAPNRDE-LMVRTHALD-RVLLWG
HYVLPHWHV---------RTFRVAYWK-PLTRPEMI--PPYDLAFD---AWW--------------------
----
>Thalassospira/1-575 xiamenensis
-------------------------------------SHGISLYG---DLKYGPDFRHFDYVNPDAPKGGTL
VQSSIV--------SFDTLNPFTL-------KGTAASGLG------------LMYDSLMVSSADE-------
--PSSLYGLIARSVEYPVDR----SFVIFHLDPRARFQDGTDITAEDVVFSFNILIEKG--SPVYRQYYAQV
DKVEALDDLTVRFDFK-PGNNRELPMIVA-ELSIMPKHYWE-------GRDFSKTTFD-PPVGSGAYKIKSF
EAGRQITYERVKDYWAADLPVNRGSNNYDTLRFDTYLDPDVSRQAFF-AGEYMIRSEH--SSREWSTAY-NT
PAIQNGQIRKEFLPD--NLPNGMQAYVFNTRKPLFSDIRVREALQYAFDFEWLNRAMFYGAYKRNVSYFANS
ELAA----TGVPEGEELDILKGFR----SQLPP------ELFTQPPQLPNFDAP-NGR----REALRHSMKL
LQEAGWQLRN---MQLVN
SKTG--EPFRFELIIRQ---PGLEKIALVLKARLRQLGVTMDIRMI-DTGQWVNRIQ---AYDFDMTTFW-W
TQ----SLSP-GNEQRFFWSSQAADQPGSRNFAGIKNPVIDELIDLVIEADGRES-LVQRVRALD-RVLQWG
FYVIPQYYLG---------GDRMAYWD-VFGRPEEVPLKGTSVM-----TWWIDPEKSRQL-----------
----
>Atopomonas/1-594 hussainii
----------------MRHIALLLGFCFSSSHLLAAGQHAITLYDE--APKYPADFQHFDYVNPDAPQGGTL
RLGGFG--------SFDSLHPYIS-------KGVAGDQLH------------LLYETLTFHSLDE-------
--PFTEYGLLAETIEKDPAN----QWVRFTLRPQARFHDGQPVTANDVAFTFNALMEHG--APGYKAYYADV

DKVVVESERQVRFDFKHSHNR-ELPMILG-QLPVLPAHWWA-------ERDFSKTTLE-PPLGSGPYKITQV
EAGRQLSFSKVADWWGKDLAVNRGFYNFAKVQIDYYRDNTVALEAFK-AGQFDFFLEM--SSKNWATAY-DT
PAIKDGRLIKEELAN--RNPAGMQGFVFNLRRPLFADRRVREALSLLFDFEWTNRQLFYGAYTRTRSYFDNS
ELAA----TGLPDTHQQYLLEPWR----KHLP------AEVFDKEYQPSKTDGS-GVI----REQRRAAFKL
LQEAGWRIEG---DQLVN
AQ-G--QPLRFEFLLAQ---TDFERVLLPYKRNLKELGIEMEIRRV-DVSQYINRLR---SRDFDMIVSG-F
GQ----SSSP-GNEQLEYWHSSSADNPGSRNLMGLKDPAVDALVESLIKSTTREN-LINHVRALD-RVLQWG
HYVVPNWYVD---------TWRVAYWQ-PLQRPANPAP--LDYA---LFTWWKAPTEPS-------------
----
>Pacificispira/1-593 spongiicola
-----------------------------IAQNTVVRSHGWAIHG---ELAYPPDFKHFDYVNPDAPKGGEL
VQGAEG--------TFDSLNPYIT-------KGNPAIG-TND-----------IYDTLMADAWDE-------
--PNSEYGLLAEWIEVEKDDDGIFTAVRFGLRPEARFHDGHPITAEDIVWSFNILREQG--APQYRFYYASV
TEVLALSDRTVEFRLA-PGDNREMPVILG-QVPALPKHYWQ-------DKDFTRTTLT-PPLGSGPYKIGAV
RAGQSITLIRVDDYWGKDLPVNVGHHNFASMRYEYFRDPTVMLQAFK-AGNLDLREEN--TSKFWATAY-DV
PAVDDGRILRKEFPH--HRPAVIQGYVMNLRRPIFQDITVREAMSYLWDFEWVDRVVTYNAYQRTDSYFENS
EMEA----QGLPGPKELEVLDPLR----GQVPD------RVFTQAYHAPVSDGS-GIN----RDNQIKAMEL
MKRAGWVVR---DGKLVN
AETG--QPFAVEILLSS---EALTPHTQTLLRGIERLGGQVTLRIV-DAAQYRARLD---SYDFDIVVGV-W
GQ----SFSP-GNEQREYWGSASAARPGGRNLFGISDPAIDRLIEELIASPDRET-LDARCRALD-RVLQWN
FFMIPMFHS---------KADRYAFWN-RFGYPETP--PMHG-TET--DFWWVDAEKDRALKRRGRK-----
----
>Tepidicaulis/1-573 marinus
-------------------------------------AHGLSLFG---SVKYEKGFERFDYVNPEAPKGGKL
RYGAIG--------SFDTLNPYIV-------KGRPAAGIQ------------LIYDTLMTLSLDE-------
--PSSEYGLIAESVTYPEDF----SSVTFTLNARATFQDGTRIKPEDVVWTFETLKAN---HPFYNAYYADV
VKAEKLSPLEVKFTFAVSG-NRELPQIVS-QLPVLPKHYWAEREFN-------ATTLE-PPLGSGPYRVKEV
KAGQSITYERVEDYWAQDLNVNIGAHNFGEMEFIYFGDPTIALEAFK-AGNVDVRLEN--SAKNWATGY-DV
PAVKDGRIVRDELET--GQGEGMQAFVFNLRREKFQDPRVRKALNYAFDFEWMNKNIFYGQYTRTDSYFENS
ELAA----TGLPEGREKELLEEHR----ENLPE------ALFTQPYENPATDGS-GNN----RDNLRKAVML
LQEAGWNVKDNK---LVN
EETG--KPLEIEFLLV--S-PTFERVVQPFARSLERLGIESSIRLV-DTAQYQNRTD---NYNFDVVVGS-F
PQ----SLSP-GNEQRDFWGCEAGKAPGGRNLMGICNPVVEALIDKVIFAKDRAE-LVAATKALD-RVLLWN
HYVIPQWHS---------PVTRVARWA-YIKHPD--PTPEYAIG--FPNIWWDARAASE-------------
----
>Dongia/1-587 mobilis
--------------------ILASLASPGARAQSSEITHALSLGD---AVKYPPDFRHLDYVNPDAPKGGTV
RFAAIG--------SFDSLNPFIV-------RG-EAASMP------------GFYELLTGSADDD-------
--IMSEYGLIAETMEVATDN----SWIVFNLRAEARWHDGKPITAEDVVFSFNILKEKG--RPQFRYYYGNV
EKAEVLGPLKVRFTFNTSD-NRELPVIMG-QLPVLPRHYWEG-------RNFDEPSLE-IPVGSGPYKVKSF
EAGRSITLERDPGYWGKDLPINIGNDNYDIVRIDYYRDPNVSREAFK-AGAYDFRVES--SAKEWATGY-ES
PALRDGRFKMELIET--DNPQGMQGFSMNLRRPLFQDARVREAMILAFDFEWSNKTLFFDQYTRTRSFFQNS
PMEA----KGLPSPAELALLEPWR----GQIPE------EVFTTEYQPPVSDGS-GTN----RDNLSRAAQL
LEAAGWKLEG--ARRI
K-DG--QAFEFEFLIDAGN-SQFERIVQPYLRNLDRLGIKGSLRAV-DSSQYEKRVS--EDFDFDMISSL-Y
PQ----SLSP-GNEQRYYWSSEAADAPGSQNYIGIRNPAIDALIETLIMAPTREE-LITASRALD-RVLQWS
HFVVPNWHM---------RGVRIAYWD-KFGRPEKLPQPTYGIG---NSAWWID------------------
----
>Zavarzinia/1-596 marina
---------------LVGAFPFFARAQTPPDIAAPAATHGIAIYG---DLKYPAGFPHFDFVRPDAPKGGDL
RLATIG--------SFDSLNPFI-------YLGDPAVAAA------------AIYDTLTVQSPDE-------
--PFSEYGLLAEAITVGPDR----AYVEFALRPEARFHDGTPVTAADAVFSLNVLRDKG--APFYGAYYANV
AKAETSGDHLVRFTFGTGG-NRELPLILG-QLPVLPAHWWEG-------RDFGRPSLD-VPPGSGAYRIGAV
EPGRGITIERLPDYWGRDLNVNRGRDNFDRIRYDYYRDETALFEAFK-AGAVDVRQER--VARLWATAY-DF
PAVRDGRVKKLELPD--NTPAGMQAYVINTRRPKFQDRRVRQAIGLCFDFEWSNKALFFDAYTRNDSYFA-N
SEMAA---TGLPEGDELALLEPFR----DRLPG------EVFTEVFELPVSDGS-GRD----RRLLLKARDL
LREAGYRVVD---KKLVD
-ETG--EQLSIEFLLF--D-VTFDRITDPFVQNLQFLGIAAGVRSV-DQSAYINRLN---DFDFDMVVGS-F

PQ----SLSP-GNEQRDFWGSAAADAKGSRNLIGVKDPVVDALIDEVIFAESRQA-LVTACRALD-RVLSWG
FYVVPHWYV---------AVTRIAAWD-RFGRPD--TAPEYGTG--FPDTWWFDPEKAAK------------
----
>Agitococcus/1-596 lubricus
-------------------LGLLIAILSNYSVAAVEITNAIALHG---KPLLSQNFSAFPYANPQAPKGGDL
RLSS-LG-------GFDSTNPFID-------KGNAIDGTD------------YLYDSLTVASLDE-------
--PFSRYGLLADKIERDTDDA---SWIIYHINPAAKFSDGVAVTAEDVAFTFNLLKKEG--SAGLKNYYRDI
DKVEALDKQRVKFSFKVKN-NRELGLIVG-EQSILPKHFWA-------KRNFNSTNLD-IPLGSGPYILSKI
DAGRSITYQRNPNYWGANLAVNKGRYNFNTITYVSYRDTTVALEGFK-AGQYDFRREN--SAKNWATAY-DF
PALKQGLVQKYTETD--NTPQGMQGFLFNIRRPVFQDIRVRQALAYAFDFEWSNRTLFYNAYTRTNSYFANS
ELAS----TGLPSAAELALLEPYR----QQLPA---SVFA---NAYQAPKTDGS-GNL----RASLMTAQQL
LASAGWTIKN---GKLTN
NKNE---RFSFEMLLVQ---PEFERIVQPFKQNLARLGIDMSIRVI-DTPQYINRMR---DFDFDMTVGG-F
PQ----SLSP-GNEQRGFFGSKAADTDSSHNLIGVKNPVVDALIEKIIAASTREQ-LVTASRALD-RVLLAH
HYVIPHYHIN---------KYRIAYWN-YLEQPKIK--AKYSLG---LDFWWANSQKLTKVRAA--------
----
>Cobetia/1-566 marina
--------------------------------------HGLAIYGE---PAHPAGFTHFPYVNPDAPIGGRM
TRAAIG--------SFDSTNPFIL-------KGNSVAGLNYFG-------G-LIYDTLLTQSADE-------
--PFTEYGLLASGIRLAEDR----SAIEFDIDPRARFHDGEPVTAEDVVFTYRLLMKDG--LPFFKSYYHDV
TRVSLIDTDTVRFDMAPDAAR-ELPLIIG-QLPVLPEHYWK-------TRDFTRATLE-LPLGSGPYRMASL
DAGRQVRYERVADYWGKDLPVNKGRHNIQTLVYDYYRDQSVSLEAFK-AGSIDLHVES--SAKNWATAY-DT
PALRDGRLKKLEVPD--SNPAGMQGFVLNERRARFQDPRVREALALVFDFDWVNRNLFYGAYQQTHSYFENS
DMAA----NGLPEGRELALLEPFK----DQLP------AAVFREP--LPIDEPT--EL----RPRLRKALNL
LRDAGYRVAD---GQLVN
HETG--KPLTLEVLNYD---TQFERVIQPWIRNLARIGIQARIRTV-DVNQYINRRR---SFDFDVVVGS-Y
PQ----SNSP-GSEQREYWTSDYADVAGSRNLIGLKSPVIDALTDALIQAQTRED-LLSATRALD-RVLRWG
FHVVPQWHLA---------ATRIALWD-KFGYPQPFPE--YLLS---LDSWW--------------------
----
>Alcanivorax/1-595 profundi
---------------MLRGLFLGLCLCSGLASAEVHKGHAIAMHGE---PQYPASFSHFDYTNPNAPKGGSL
RLHVVG--------SFDSLNPFVP-------KGRPAAGMGATD-------NSHLYDSLTVRGEDE-------
--PFTQYGLLAKTIEWPDDR----SWVRYHLHEEARFSDGHPVRAEDVAWTFNTLMEKG--RPFYSYYYAEV
DNVEINDPLTVTFHFKPGSINKELPLIIG-QLPVLPKHLWE-------KQDFEKSGMS-IPVGSGPYRIVKA
DPGKQITYALRDDYWAKDLPVMRGRNNFGTITYDYYLEENVALEAFK-SGNYDWRHEN--NSKLWATAY-KG
PAFSKGQIKTETVAH--HNPWGAQGFLFNIRKPLFQDMTLRKAIGYAFDFEWSNSNLFYGQYKRNRSYFENS
DMAA----TGLPSKAELALLEPFR----DQLP------DEVFTTAYQPPQSDGS-GRP----RENLRQAQQM
LKQAGYQFRD---GTLFS
PKG---TPVKFEIILSS---PAFERVVLPFSRNLKALGIDASVITI-DSAQYVERIR---NFNFDMIVGR-I
GQ----SSSP-GNEQKEYWSSAAADQPNSRNLIGIQNPVVDALVDQIIAAPNREA-LVTSCRALD-RVLQWN
YYSVLNWYTN---------EHRIAYQS-RLRHPDFARY--VPLDTS-LDTWW--------------------
----
>Thiopseudomonas/1-567 denitrificans
--------------------------------------HALTLYGE--APKYPADFRHFDYVNPDAPKGGTL
RLSGLN--------GFDSLNPFIP-------KGNAADHIG------------LIYDTLTYHSPDE-------
--PFTEYGLLAERIERDPDN----LFVRFQLRPEARFHDGQPVTAEDVAHTFELLTTQG--HPLYRQYYADV
AEVVVESQHSVRFDFKHGHNR-ELPLILG-QLQVLPKHWWQ-------GNDFSKTSLQ-PPLGSGPYRIGRV
SAGRSIIYERVADWWGKDLPVSRGQYNFDRVHIDYYQDMSVALEAFK-AGQFDFNLEY--SAKDWATGY-NS
PALKRGDILREEIHN--HNPVGMQAYVFNLRRPMFQDRRVREAIALLFDYEWTNKQLFYGAYLRTNSFFANS
ELAS----SGLPDEAELELLEPLR----GQIP------DEVFTTPFKLPVSDAS-GII----RERRLQAWQL
LQDAGYRIEK---DRMVN
AD-G--QPLAFEFIIAQ---TNLERVLLPFKRNLEELGIEMTIRRA-DVSQYINRMR---SRDFDMTSAI-W
SQ----SSSP-GNEQRDFWHSSSFDNPGSRNFIGLQDPAIDSLVEGLINADSRDS-LITHTRALD-RVLLWG
HYVIPNYYTD---------SWRIAHWK-HLQRPAKPPL--YDYG---LFTWWID------------------
----
>Pistricoccus/1-602 aurantiacus
-------LASLLLAYLVLCGSSRDAFAEEESGGTVSTVQGLALYGE---PALSEDFTHFPYVNPQAPKGGSL
ARAAIG--------SFDSTNPFII-------RGTPATGLT------------QIYDTLMETNPDE-------

--PFSQYGLLAKGVRLDPKR----RWMEFDLNSEARFHDGKPVTAQDVVFSFEILRDKG--QPFYAAYYADV
TAVKALNKKTVRFEFAESNSR-ELPLILG-QLPILPRHYWQ-------ERDFSATTLD-KLLGSGPYRIAEV
QPGRRIVYQRVEDYWGKDLPVKQGRHNIDRLIFDYYRDQTVALEAFK-AGNLDFRLET--SARQWATAY-DF
PAAKEGFVKKLEVPD--GQPAGMQAYVINLRRDKFQDVRVREALNLAFDFPWLNKKLFYDAYERTHSFFENS
EMAA----QGLPSEAELELLEPYR----DQLP------ERVFTEP--LPIEHPE--EL----RPRLAEALNL
LQEAGYVVRN---GKMVN
TETG--EPLSLEVLLYD---TQFERVVQPLLRNLSRLGIDGKIRVV-DVNQYLNRLR---KFDFDMIVGG-F
PQ----SNNP-GNEQREFWTSAYAHQPQSRNLIGLENPVVDELVEKLIRADSREE-LDTAAHALD-RVLRHG
FYVIPQWHLA---------ATRIALWD-LYGYPQPHPE--YNLD---LEAWWVDPERAREI-----------
----
>Dasania/1-574 marina
-----------QNIIKS---------------------HAIAMHGS---AKYPASFSHFDYTSPHSAKGGQL
RLGLQG--------SFDSLNPFIA-------KGNSAAHLG------------LIYDSLTVSSMDE-------
--PFTRYGLLAESMEYPADR----SWIIFNLQPKAKFHDGVAITADDVVFTFNTLMEKG--NPFYQSLYADV
ESVEALNNKRVRFSFKNT-VNHELALIVG-ELAILPKHYWQ-------DKDFASSSLE-MPLGSGPYSLDNI
NPGRSLSYKRVKDYWAKDIAVNIGLYNFDRIVIDYYKDANVLLEGLK-AKQYDFRLEN--SSKQWATGY-AS
DALANGELIQEEIAH--QLPRGMQGFLMNLRRPLFQDMRVRQALNLAFNFEWSNSNLFYNAYTRSESYFANS
ELAA----TGLPSPQELAILEPYR----SQLP------NSVFTHPYQAPVSSGN-KYN----RANLIKATQL
LKQAGWRIVD---NQLVD
SNN---QPFEFEITLVQ---PSFERIVNPYIQSLKKLGIKAHIQHS-EVSQYINRLR---DADYDMIVSG-F
GQ----SLSP-GNEQKEYWHSSTADTPASRNLMGVKNPVIDHLVELVISAPDRES-LVTRTRALD-RVLLHH
HYLIPQFYIA---------SHRIAYWD-KFNKPATSAKYDYDYQYS-LITWW--------------------
----
>Acidisoma/1-571 cellulosilyticum
-------------------------------------GYGVSVTG---LPALPAGFKNFPYVNPNAPKGGSV
TFSQIG--------GFDSFNPFII-------RGNPAAG-ANY-----------LWDTLLRQSSDE-------
--AASAYGLLAETVAVAADH----SSVTFTLRPEAKFADGTPVTASDVVWSFETVCAKG--QPFYQQYYEAV
SSAQAPDARTVVFNLH-PGAAKELPLILG-GLTVFPQAWWK-------GRDFTAPLTD-APLGSGPYKVESL
ALNRSVTYRRRADYWAANLPVCLGFYNFDRITYDYFGDPSVAMEAFK-AGDIDFRNEN--IAKNWATGY-DF
PAVQKGLVKKQVFPD--RLPAGMQGFGMNTRRAVFSDPRVRQAIASAYDFEWANKVLFYDGYQRTLSYFSNS
DLAS----SGLPSGAELALLEPFR----AQLPS------ELFTKPFALPVNDGS-GNN----RPALIAALHL
LEQAGWTVQ---DRMLKN
KAG---ETMAFEILLSD---PSFERVTLPYVQDLKRLGIDVSVRVI-DPSQYQSRMN---NFDYDMTVVL-F
PE----SDVP-GSEQRDYWGSAAAKLTGSNNQMGVSSPVVDALVDKVIAATDRDT-LYAAVHALD-RVLLWG
WYVVPQWHL---------GAYRLAFWD-VFGFPNQP--LRAGFDI---DSWWIDQAKA--------------
----
>Rhodovarius/1-587 lipocyclicus
------------------------------QGAAPRRVHAISLLG---EAALPADFPHWAWANPDAPKGGDV
TFAAIG--------SFDSFNPYIL-------RGAPAAG-SSI-----------LYDTLTSSAQDE-------
--ASTEYAHLAAFIELPADG----LGVAFELRPEARWHDGRPITAADVVWTFETLVRQG--RPFYRAYWGDV
ESVRAEGERRVVFRFK-TNENRELAQVLG-QMPVLPKHWWE-------GRDFSRPLQE-APLGSGPYKLERF
ESGRSVTYRRVEDYWARNLGTARGLNNFGSIRYEYFRDSTVAFEAFK-AGQVDARMEN--VARDWATGY-DF
PAIRRGLVKREAIRH--EIPTGMQSFSFNLRRPVFQDARVREALVQVFDFEWLNANIFFGSYARTSSYFSNS
ELAS----SGLPEGRELEILSQFR----AQLPE------RLFTEPFRLPATDGS-GNN----REGLRRALDL
LRQAGWTVR---DRKLVN
AQG---QQFRFEILLDS---ATFERVALPYVQWLQRLGIEASVRTV-DPAQYRVRMD---AFDFDMTVDV-M
GQ----SLSP-GNEQRDYFSCEKANEHGSQNVPGICSPVIDALVDQVINAPSREE-LIARTHALD-RVLLWQ
HFVIPNWHS---------REFRLAWWD-RFGRPDRN--PRYGSGFP--TSWWVDAAKDRALTEARR------
----
>Tepidamorphus/1-611 gemmatus
-----------IIRRVTAALAIGLLTATAAVAQDDGWKHGLSLFG---EVKYPPDFTHFDYVNPDAPKGGVF
RQATVG--------TFDTLNPFNL-------KGTPAAGTG------------LLYDTLLSNALDE-------
--PSSEYGLIAEAVKYPDDY----SSVTYRLNPKARWHDGQPITVDDVIWSLDTLKRS---HPFFNAYYRNI
VSAEATGEREVTFRFDQTG-NRELPQITG-QLYILPKHYWEGTDAQGRQRDFTASTLE-PPLGSGPYRIKEV
QPGRTITYERVPDYWAADLPVNAGQHNFDEMRFEYFRDTTVALEAFK-ADQFDFRVEN--SARRWATGY-DF
PAARRGDVILQTFRT--ANAEPMQAFAFNLRRPKFQDPRVRQAFNYAFDFEWLNANIFYGQYERTNSFFENS
ELAAT---G-LPEGRELALLEEIR----DMVPP------EVFTTEFRNPVGGDQ-RKV----RENLRIATQL
LREAGWEIRD---GRLVN

TQTG--EPFEVEFLND--Q-PDSERILAPYVQQLARLGIRATIRTV-DTSQYRNRVD---NFDFDIITAV-F
PQ----SLSP-GNEQRDFWGSDAADAVGSRNVIGIKNPAVDFLIDKIVFAEDRES-LVAASRALD-RVLLWN
HYVVPQFYS---------PDIRTARWN-RFGLPE--ISPDY--G-FTTSTWWWDAEKAASIRRGS-------
----
>Algihabitans/1-580 albus
-------------------------------GQRVYRSHAIAMHG---EPKYPANWTHFDYVNPDAPKGGQM
TVGSQG--------TFDSFNAENG-------RGNPVGAG---------------YESLLESSDDE-------
--AFTKYGLIAEWVEWPEDR----SWVTFKLRDEARWHDGQPITVEDIIWSYNTLVNDG--QPFYRFYFGAV
EVVEEVGPRQVRFIFN-ERDNRELPLIIG-QMPVMPKHYWE-------ERDFTTTTLD-PPLTSGPYRISAF
EPGRYVVRERVEDYWGADLPINVGRHNFNRMRSEFFRDDTVIRTALK-AGTLDFRREN--QAKAWALDY-DI
PAVQQGWLRQEEVRH--NLPTGMQAFVMNTRRPLFQDARVRRALAFAFDFEWTNSALFFDLYARTSSYFSNS
ELAA----SGLPEGEELEILEGHR----GQIPD------EVFTTPFWVPGTDGS-GWP----RDNIIQGLEL
FEEAGWEVR---DLQLVN
VETG--QPMRFEILLVS---PAFERIVLPFVRNLTRMGIDARVRLV-DQSQYINRLR---SFDFDMIVSV-M
PQ----SDSP-GNEQRDFWSSEAADQPRSRNLAGIKDPVIDALISQVITAPDRES-LVARTRALD-RVLQWG
HYTIPQWHS---------RSLNLLYWD-KFARPETDGLSNNGTAID---RWWYVQAKADEL-----------
----
>Cobetia/1-575 amphilecti
--------------------------------------HGLAIYGE---PAHKPGFTHFPYVNPEAPVGGRM
TRAAIG--------SFDSTNPFIL-------KGNSVAGLNYFG-------G-LIYDTLMIPSADE-------
--PFTEYGLLASGVRLAEDR----SAIEFDIDPRARFHDGVAVTAEDVVFTYRLLKDEG--LPFFKSYYHDV
TGVSVIDADTVRFEMAPGAAR-ELPLIIG-QLPVLPEHYWK-------SRDFTRATLE-LPLGSGPYRMASL
DAGRQVRYERVDDYWGKDLPVNKGRHNIQTLVYDYYRDQTVSLEAFK-AGSIDLHVES--SAKNWATAY-DT
PALHEGRLKKLEVPD--SNPAGMQGFVLNERRERFQDPRVREALALVFDFDWINRNLFYGAYQQTHSYFENS
DMAA----DGMPTGKELALLEPFK----DELP------SAVFDKP--LPIDEPQ--EL----RPRLRKALGL
LREAGYEVVK---GLLVS
RNSG--KPLTLEVLNYD---TQFERIIQPWIRNLARIGIKARIRTV-DVNQYINRRR---SFDFDVVVGS-Y
PQ----SNSP-GSEQREYWTSDYADVAGSRNLIGLKSPVVDALTDALIQAQTRED-LLSATRALD-RVLRWG
FHVVPQWHLA---------ATRIALWN-KFGYPQPFPE--YQLS---LDSWWIEPDKDASL-----------
----
>Parvibaculum/1-587 sedimenti
-------------------------------APATTERHGASLFG---DLKYGPDFKHFDYVNADAPKGGEL
RYAAIG--------TFDSLNPYIV-------KGEVGAGIN------------LIYDTLLEPSMDE-------
--PGAEYGLIAETISYPDDF----SSVTFKLRKEARFQDGTPITPEDVIWTFETLKKL---HPFYAAYYRNV
EKAEKVGADSVRFVFSVKG-NRELPQITG-QLPVLPKHYWMAKDAKGRPRDISQTTLE-APLGSGPYRIAEV
IPGRTIVYERVKDYWAKDLPVKRGTNNFDRLRFDYYGDPVVAFQAFK-ADQVDLRIET--SAKNWATGY-DF
PAAKDGRVKREEIRM--RNPAGMQSFAFNLRRDMFQDERVREAFNWAFDFEWQNKNIFSGQYARTDSYFANS
ELAS----RGVPQGLELEMLKPFE----KELPP------ELFTTPYSNPKTDGS-GNN----RANLRHAAEL
LDAAGWKIVNG---TR
MKDG--KPLAVEFLLA--D-PQFERVVAPYKQSLDRLGIKVTLRTV-DTAQYQNRLD---NRDFDIVVES-F
GQ----SLSP-GNEQREFWGCEAARRVGSRNVIGICDPVIEKLIDRVIFAKSREE-LVAATHALD-RVLLWR
HYVVPQWYS---------PFARVAYWS-RLAHPK--KMPDYALG--FPDIWWYDASDQKASP----------
----
>Chromohalobacter/1-608 salexigens
----LFVRALLVIPGLWALSLSALAVPAAD----VATVGGISLYDS---PALPDDFTHLPYTNPDAPKGGEL
RQAAQG--------SFDSTNGFII-------QGNPADGLS------------HVYDTLMEASADE-------
--PFTMYGLLAGGIRLDPDR----HWMEIDLRRSARFHDGHPVTAEDVVFSFRLLRDQG--QPFYRAYYAGV
DQVEALDDDTVRFEFSDNESR-ELPLILG-QLPVLPKHYWQ-------SRDFTSPTLD-KPLGSGPYEVASI
LPGRRIMYRRVDDYWGRNLPINRGRHNIDRLVYDYYRDQTVALEAFK-AGNLDLRRES--SAKNWATAY-DT
PALEAGFIKRMTVPD--AQPAGMQAFVMNLRRAPFQDRRVREALTLATDFDWLNTHLFYGAYQETDSYFESS
EMEA----QGLPSDDELALLAPYR----DILP------DDVFEEP--LPMSRPD--TL----RARLKKALSL
LREAGYEVRD---GVLVD
TDTG--RPMRLQFLLYD---TQFERVTLPLIQNLERLGIQASVRVV-DVNQYLTRRR---NFDFDLMIGS-F
PQ----SANP-GNEQREYWTSEYADAPRSRNLIGLRNPAVDALVDRLIGANSRQA-LDTTARALD-RVLRWG
FYVIPQWHLD---------GTRIAMWD-KFGYPQPFPE--YTFD---LSSWWVDPQRAARVEERQRGE----
----
>Acidibrevibacterium/1-580 fodinaquatile
--------------------------------------YVVTLLE---KPGLPRDFSHFPYVDPAAPKGGVV

VLSAIG--------SFDNFNGFIV-------RGSAPDD-IGR-----------IYDTLLRANINE-------
--PSTAYGHLAETIELARDH----SRVVFTLRDGAYFHDGAPLTANDVVWTFDTLRRDG--RPFYRLYYGDV
ERAEALDARRVVFHLK-PTPDRELPLILG-EMPVLPRHFWK-------DRDFSRPLTD-MPLGSGPYRVEKA
EFGRSITYRRVHDYWALDLPTVRGQFNFGRIQVEYFRDTTVAFEAFK-AGQVEFREEN--VAKQWATAY-DF
PAIQRGLAKKENLPQ--HLPTGMQGFAMNTRRPLFQDRRVRQAMDLVFDFEWCNANLFYNAYTRTHSYFSNS
DMAS----SGVPEGAELALLEPWR----AHLPP------ALFTEPFSLPVTDGS-GNN----RANLKKALGL
LEQAGWRVK---DRMLAN
GEG---QPFTFEILLDE---PAFERVALPYVQWLAKLGITATVRTV-DPAQYQQLLN---TFSFDMTVAV-F
PQ----SDYP-GNEQVGYWSSGAAKQEGSDNLMGVADPVVDALVQNVVTAPDYDH-LLPAAHALD-RVLLWN
WYMVPHWYL---------QSVRVAYWR-RFGRPDKP--VRSGLAF---DSWWVDEKLAEETDNARRAG----
----
>Kiloniella/1-606 litopenaei
-----------MRALLLVNFVLCVLLPFSATAQTT-KSHGIAIHG---EPGYPADFTHFNYVNPDAPKGGEI
RLASEG--------TFDSFNPYII-------KGVPGPG--F------------TPESLLVASADE-------
--AASAYGLIAESLEWPEDR----SWVIFTLRPEARWHDGVPITVEDVIFSWETIRNDA--GPYVKNYYAIL
KNVEKVGDNQAKFTSG-EPGNKEFPIRAG-SLPILAKHYWE-------DRDFSKTTLE-PPLGSGPYKITDF
EAGRYVVQERVKDYWGKDLPVNKGQDNFDKIRTTYYHDDDVIKLAIK-SGEIDYRNER--SSSAWAQDY-DV
PAITKGWLKKESIPH--KRPQGIQGFFYNTRRDIFKDPKVREAIGYVYDFDWSNKNLSFGLLTRTTNYFGNS
ELSS----TGIPKGRELEILEKYR----GKVPD------NLFSEVFHVPTSDGN-GWP----RHNYRKAFEL
LAEAGWVVK---DLKLVN
EKTG--EQMSFEMLVSS---KGMERTILPFVTSLSKLGIDARIRLV-DRSQYINRIR---DFDYDIVIIK-L
SA----SITP-GTEQRGFWTSETADKKGSGNFAGIKDPVVDELVEAIANADDREE-LVATVRALD-RVLLWG
YYVIPQFHT---------EDDRVLYWD-KFSRPKMT--PWRGTSTS---YWWFDNDKEAALMASMAKNE---
----
>Dichotomicrobium/1-581 thermohalophilum
--------------------------------------HGLSAFG---ELKYPPDFKHFDYVNPDAPVGGSL
SMIG-----TAGVITFNSFNPFII-------KGDAAQGMALL------------FDSLMTRA---------Y
DEPDAVYGLVAHSAKLEPDR----SGVTFYMRPEAEFADGSPVTAEDVVFTYETLKRNG-RP-IYNAMLRDV
EKAEALDDHTVRFTFNAPGKRRLPMMIA--ELPILSKAYYQEQ-------PFNQSTLE-PPLGSGPYEVVDF
KQGRFVTLRRREGYWGWHLPVNQGRYNFGELRYEYFRDRTAELEALK-AGEFDLREEF--TSKSWATEYD-I
QQVRSGQMKLLTLED--GRPSGAQGFFINTRREKFADRRVRLALDYAFDFQWTNKHLFYGLYERTASYFENS
-ALKAQ---GKPSEAELALLEPY----RDQLPES------VFEDAYVPPKSDGS---GR--DRRLLRQASRL
LNEAGWVIR---DGRR
VNAETGEPLEIEFLIFS---PTFERVIGPYVKNLEMIGIDARIRRV-DPSQFQERMKS---FDFDITTQ---
--RFVLNQTPGPE-LRNYFGSDAAGAKGSFNLAGVSSPVVDALIDEVLAAETREE-MQTAARALD-RVLRAG
HYWVPQWYKT---------SHHLAFWN-KFSWP--ETKPPYDR--GVIETWWYDEDKAARLEDA--------
----
>Thiopseudomonas/1-602 denitrificans
---------------MLRFLVIATLLHAGLVQAAVTLSHGFARFG---QPQYPASFSHFDWVNPAAPKGGHL
RLMALG--------TFDSLNPYIL-------KGTSPIGTGDFY----QYGISELNAPLMVGHGSFDPS---G
DETASAYGLVAESIQYADDL----RWVVFNLRKQARFHDDRPITASDVEFSWRTLKK--HGHPQYRNYLKDV
ARVEILAPLKVRFVFK--QPATRQQILDLGDLPVLPKHYWSKR-------DFTQTSFE-PPLGSGPYRITEV
KPGRSLRFERVKNWWGKDLPVNVGKYNIDRIDVDFYRDRHVAFEAFK-AGSFDLYIEH--QAKNWASNY-RF
PALLRGDVLRAEIPH--QIPTPTQALFINTRRAPFDDVRVREALSLLFDFEWANRTLFNSVYQRTTSFFPNS
PFAAR----DIPLGAEWLLLKPWQ----EQLPDG------LLEQPFQVPVTDGR--GI---PREQLARALAL
FQQAGWKAGR---NGLKN
SQG---KLLQLEILLVN---PGLERILQSYVQTLRRIGIEARLRTV-DRAQFKHRLD---AFDYDMTLLV-L
PQ----SLNP-GPEQWLYFHSSQAGIRGGKNYAGIQNPVVDAMLEAVMQARTRNE-LESAMQALD-RVLLWQ
HYVVPNWYIS---------QHRIAYSK-RLQHP-QIPPYT-----LGLRAWWLD------------------
----
>Methyloceanibacter/1-588 superfactus
--------------------------------------HAVSLVG---EPKYQAGFTHFDYVNPDAPKGGRA
RIASIG--------GFDSLNPVLY-------RGEQAAGLG------------LVTESLMSDSIDE-------
--SSTSYGLIAECASYPADY----SSVTFKLREGARWHDGTPITTDDVIYSLEVNKTA---NPRMGLYYKNV
TRAEATGDNEVTFYFDSKG-NRELPMIMG-QLTILPKHYWTGKDAKGEQRDPMKTTLE-PPLGSGPYRIKEA
TPGRTVVYERVDDYWGKDLPVNKGQWNYDELRFDYYRDTTVAFESFK-AGNLDYWQET--SAKNWATAY-DV
PAVRDGYIKRQEVKI--KRTQPMQAFVLNLRRPQFQDRRVRQALNLAFNFEWANKNLFYGQYERVGSYFENS
ELAAP---PEPPQGRELEILNEVK----DQVPT------EVFTEIHKNPVNEEQ-ADL----RGNLRKAVML

LKEAGWQVKD---GVLTN
AKTG--QKMTIEFLLV--S-PLFERIVQPYLRNLERLGIKGSIRLV-DSAQYTRRLN---VFDYDIIVST-F
AQ----SESP-GNEQRDFWGSEAADREGSMNLIGIKDPAIDKLVDRIIYAKDREE-LVAATRALD-RVLLWH
DFVVPQWFS---------PYVRIAYWD-RYGQPE--QLPGLTPG-FLQ-VWWYDEKLGERLPGPSKR-----
----
>Futiania/1-575 mangrovii
-------------------------------------AHGLSLTG---ETKYQEGFAHFDYVNPQAPKGGAT
TLFAIG--------GFDSLNPFIA-------KGNAASGLG------------LIYDTLMANALDE-------
--PNAEYGLVAKTVTHPADF----SWVEFELRPEARFHDGKPVTAEDVVFSFETLRDEG--QPFYRLYYANV
TTAEALGPHRVRFTFDTAG-NRELPVIMG-QLPVLPKHWWETRAFG-------ETTLE-PPLGSGPYRVASV
DTNRSITYERVPDYWAKDLNVNVGANNFGRIRYEYFRDMDVAFEAFK-GGSYDFRSEN--SARRWATGY-EF
DAVRKGEVTVETVPD--RNPQGMQGFVMNLRRAPFDDPRVRQAFNLAFDFDANETLFYGQYARTRSYFEGS
ELAA----TGLPEGRELALLEPFR----GEVPE------EVFTTEYNPPETGGR-RGL----RENLRKATEL
LNAAGWAFRDG--ALR
NGEG--KLLNVEVLLV--S-PDFERIVAPYVQNLERLGIEATIRIV-DTAQYIQRLN---VFDYDMIVMS-W
GQ----SLSP-GNEQRNYWGCAARDRDGSRNYAGICNPAIDALIDKVIFAETRQD-LVAATRALD-RVLQWG
RYVVPHWHFRTVIDGVSQPAFRLAFWN-RLAHPE--ALPPYTPG--FPTVWW--------------------
----
>Perlucidibaca/1-586 aquatica
------------------LVLSCTLSAAGFSQARIEISHALALHG---QPALPLGFTHFPFANPSAPIGGHL
RLDS-TG-------TFDSLNPFIS-------KGISAEGIN------------RIYDSLTVASPDE-------
--PFTRYGLLAERIERDPKDA---SWVIYQLRPEARFSDGKPVRAADVVETFRLIQSHG--APAYKAYFADV
KHVQALTPLRIRFEFRSKT-NRELPLIVG-EMSILPKHYWQ-------NRAFEKSSLD-IPIGSGPYTVANV
DPGRRISYARNPNYWGWKLPINRGLHNFTRISYRYYRDGSVAFEGFK-ADQYDIRLEN--KAKTWATEY-NF
PAVQNGQVIKLEQRH--ENPSGMQGFLFNTRRSPLNDIRVREAMSLAFDFEWSNRALFYQAYKRTSSYFDNS
ALAA----TGLPSQAERRLLTPFR----RQLPP---QVFG---PAVMPPRSQGD-GYN----RDNLIAAQQL
LKQAGWRVQQ---GQLIN
AKGE---PLRLEMLLVQ---PEFERITQPFKRNLARLGITLDIRIL-DAAQYVERLR---QFDFDLTVGG-F
PA----SSSP-GNELWSFWSSTAAATPGSRNLTGLKSAAVDALVGQVTQAQDRGE-LVTAVRALD-RVLRAQ
HLVVPHYHTP---------VIRIAAWN-RFGRPERP--PRFGDG---LDTWWF-------------------
----
>Ferrovibrio/1-590 terrae
-----------------LLGAIGLLSLCLIAPALAQPRHGLSAFG---DLKHPPGFRHFDYVNPDAPKGGEL
RSWQLES--------YDNLNPLIL-------KGVMARNLSLT------------FQGLMARG---------M
DEPDAVYADLAESAELADDR----SWVAFNINPKAKFSNGTPVTAEDVVFTVEAAKKDG-HP-VYQLVLRDV
DSVRAESRLRVVFRFAETESRRDLPLTVA-QLPILPKAWFAGR-------DFASPTME-PIPGSGPYRISRV
DAGRSLTYERVKDWWARDLPVNRGRHNFDWYRDDYYRDREIAFEAFF-AGEYDFREEV--TARHWATSYTPK
PAFQDGRVKREVFKD--ETPSGVQAWFLNSRKSHLADPRVRQAINLAYDYEWANKTLFYGLYKRTRSMFENS
-DLAAT---GLPSPAELELLEPYR----DRLPPE------LFTREFQPPQTDGS---GN--NRANLKKAQAL
LLEAGWKIS--NGK
LVDAKGKPFELEFMLYE---PSFQRIINPFARNLERIGIDISIRVV-DISTFENRMRS---FDYDVMSR---
--RFVQPLTPGIE-QRNYWASRSAGTVGSFNFSGVNDPAADDLIEHIVKARNRDE-LRAATRALD-RLLMWG
WYVVPHWYSG---------TFKLGYWD-RFHRP--ARKPIYDV--GLVDTWWID------------------
----
>Hyphomicrobium/1-569 sulfonivorans
--------------------------------------HGISTFG---ELKYGPDFKHFEYANPDAPKGGRF
SSTG-----TGGTKTFDSFNAFIL-------KGDSAQGLEYL------------FDSLMTRA---------M
DEPDAAYGLVAESADVAEDG----RSVVFKLRPEAKFSDGTPVTADDVVFSLEVLKEKG-HP-NYSLSLRDV
AKAEALDPHTVRYEFKGNLIRDLPLTVA--GLPILSKAFYTKH-------PFDQSSLE-KPVASGPYEIGNF
KPGTFVSYKRRPDYWAKDLAVNRGRFNFDEIRYEYFRDRTLELEGLK-SGTFDYREEF--TSIDWATAYD-I
PAVKEGRLQRLIMPD--GRPSGAQGFFINTRKDQFKDKRVREALDLAFDFEWSNKNLFFDLYSRTQSFFENS
-ELKAS---GLPSAAELALLEPF----KDKLPPE------VFGEPYVPPATDGS---GR--NRDNLRKAHQL
LAEAGYG-Q---GG--
-KPLNVEILSFEEGFD--------RIIIPYIENLKRIGVNASLRRV-DPAQYERRIKS---FDFDLAIQ---
--RYALRLTPGIE-VKTYWGSDAAKIDGSFNLPGIADPVVDALIDKMVEAKSREE-LTVAARALD-RVLRAG
NYWVPQWYKA---------SHNVAFWD-MYGRP--AVKPKYDD--GVIDTWWFDADKAAKL-----------
----
>Microvirga/1-584 solisilvae

------------------------------------ETHGISSFG---EMKLGPDFKHFDYVNPAAPKGGTL
VLQIRQAGGNQNFDTFNTLNIYVL-------RGDGAAGMGST------------FDTLLVGS---------G
DEPNTMYGLVAKSVRISDDK----LTYRFILRPEARFHDGSKLTARDAAFSLMILKEKG-HP-VYRSILAQM
TSAEAEGDNVLHVQLSPQRSRDLHLVVAS--MPIFSERYWQGK-------DFEASTLE-APLGSGPYKVSRF
EQGRYIEFERVADYWAKDLPVNVGQNNFDRVRYEYYRDRTVAFEAFK-SGSINYHEEF--TSRIWATNYD-F
PALKDGRVKREELPN--DSPAPIQGWYFNTRREAFKDPRIREAIGLAFDFEWTNANIMFGAYRRTTSYFENS
-DMKAM---GLPSPEEIALLEPF----RAQLPAA------VFAEPYTPPVSDGS---GQ--DRQLLRRADEL
LRQAGCK-R---EGGT
LKLPNGQPLTIEFIDFQ---NSLQPHTQPFQANLKRLGIEARSRIV-DAAQYQQRMND---YDFDMTTR---
--ALVGTTTPGDD-LRAVYASEAAKTPGTRNIAGIAHPAVDALVEKIGNAKTREE-LNIASRALD-RVLRAG
HYWVPMWFKA---------SDWIAYWD-EFSRP--QTKPRFSS--GAPGTWWYDAEKAKRI-----------
----
>Siccirubricoccus/1-582 soli
-------------------------------------THALSLLG---EPALPADFPHFPWVNPEAPKGGEI
ALTALG--------SFDSFNPFIL-------RGTPAVGILAN-----------VYETLLRDSADE-------
--ASTAYCHLAAAIELAADG----LGVTFMLREEARWHDGKPITAEDVVWTFNTLREQG--RPNYRSYYGDV
ASVAAEGPKRVTFRFK-TNENRELALILG-QLRVLPKHWWE-------GRDFARPLLD-PPLGSGPYQLERF
EPGRSLVYRRVEDYWARDLPTMKGTANFDTMRYEYFRDSTVALEAFK-AGQIDFRTEN--VAKDWATAY-DF
PAVRRGLVKLDEIRH--ELPTGMQAFAMNLRRPLFQDARVRRALIEVFDFEWMNANLFYGSYTRTTSYFSNS
ELAS----SGVPEGREKEILEKYR----GQLPE------KLFSEPYRLPVTDGS-GNN----REGLRRALDL
LRQAGWTVR---DRRLVN
ARG---EGFAFEILLNG---PTYERVALPFVQSLQRLGMEPRVRTI-DPAQYQVRMD---SFDYDMTMDV-L
PQ----SLSP-GNEQRDFFTCAEAQKQGSQNIAGICNPVVEELVELVISAPDRAE-LVARTRALD-RVLLWQ
DYMIPNWHS---------RTFRVAYWD-KFGRPPRN--PRYALAL---DSWWVEGGREGAVEQGKREA----
----
>Methyloligella/1-583 halotolerans
-----------------------TFGAEASEP-----RHGLSAFG---DLKYPAEFEHFDYVNPDAPKGGRF
SLIG-----WGGVTTFNSLNPYIL-------KGDAAQGMEML------------FDSLMTRA---------Q
DEPDAVYGLVAKSGEVADDG----MSVTFKLRPEARFQDGSPLTAEDVAFSFETLKEKG-HP-LYRQMLRDV
EKAEALDDETVRYRFKGNQTRDLPLTVA--TLPIFSKAYYADK-------DFAETTLE-APLGSGPYTVEGL
HQGRNITYKRDPDYWGKDLPVNRGRWNFDEIRFEYFRDRTAAMEAFK-SGAYDFREEF--TSKVWATEYD-F
PAITSGRVKKATLPD--DTPSGTQGWFLNTRRDALKDPKVREALGLAFDFEWTNRNLFYGLYDRTQSYFENS
-AMRAE---GEPTDKELALLDTL----SAPVPEA------AKGEAYAPPVTDGS---GH--DRALLKKADAL
LREAGWTVK---NG-V
RVNEDGEPLKLEFLNFE---PAFERVTAPYIRNLQRLGIQARMRMV-DPSQYQQRLKG---FDFDVVTQ---
--RYGMELTPGPE-LRSYFGSEAADMTGSLNLAGIANPAADELISDIIEAKSRGE-LVTAARALD-RVLRAG
HYWVPHWYKG---------THTVAYWD-KFSQP--EQKPRFDR--GILDTWWYDEAK---------------
----
>Salinicola/1-582 lusitanus
----------------------------------VPTVHAIALYDD---PALPAGFQHLPYANPEAPRGGTL
RRAANG--------SFDSTNPFII-------QGTPAAGLN------------QIYDTLMESSADE-------
--PFTMYGLLAGGIRLDPDR----HWMEIDLRPEAHFHDGHPVTAEDVVFSFQLLRDKG--SPFYRAYYASV
ESVSAVSERTVRFEFSNNQSR-ELPLILG-QLPVLPEHYWQ-------DRDFTRPTLD-KLLGSGPYEIATV
DPGRRIVYRRVDDYWGRDLPLNRGRYNIDRLVYDYFRDQSVALEAFM-AGALDMRIES--SAKNWATAY-DT
PAVEDGAIQRVIVPD--GQPAGMQGYVINTRRAQLSDPRVRRALGLAFDFDWVNQHLFYGAYQRTESYFQNS
EMAA----RGLPGDEELALLAPYR----DQLP------AAVFDQP--LPIAQPE--AL----RPRLREALGL
LRQAGYDVRD---GTLVN
LETG--RPFTLEFLLYD---SQWERITQPFVRNLERLGIRSKIRVV-DVNQYLDRLR---RFQFDMIVGS-F
PQ----SANP-GNEQRDFWSSEYADVPQSRNLAGVRDPVVDALVDDLIRADSREK-LDTASRALD-RVLRWG
FYVVPQWNFD---------GTRVAIWN-KFAYPRPFPR--YTPD---FSVWWVDPERAGAIAARQRGQ----
----
>Crenalkalicoccus/1-580 roseus
-------------------------------------THALSLLG---EPALPPDFPHWPWVNPDAPKGGEI
VLSALG--------SFDSFNQFIL-------RGTPAVG-LGN-----------LYDMLLRQSYDE-------
--ASTEYCHLAGAIELPADR----RGVSFELREEARWHDGRPVTAEDVVWTFQTLRSHG--RPFYRSYWADV
TEVVAEGPRQVTFRFR-TDENRELALILG-QMQVLPRHWWE-------GRDFARPLLE-PPLGSGPYRIERF
EPGRGITYARVPDYWGRDLPTMRGTNNFDVMRYEYFRDATVALEAFK-AGQVDFRTEN--IAKEWATAY-DF
PAVRRGLVRRDEIRH--ELPTGMQCFVMNLRRPLFQDARARRALIEVFDFEWMNANLFYDSYARTTSYFSNS

ELAS----SGLPEGREREILEGFR----GQVPE------QVFTEEYTLPVTDGS-GNN----REALRRALAL
LREAGWTVR---DRRLVN
GQG---QPFEFEILLNG---PSFERVALPYVQWLQRLGMAPRVRTV-DPAQYQVRLD---AFDYDMTVEV-F
GQ----SLSP-GNEQRDYWTCEKARENGSRNTAGICNPVVDALVEMIIAAPDREE-LVARTRALD-RVLLWN
NYVVPHWHS---------RTFRIAYWD-KFGRPPRN--PRYALAL---DSWWVERGREGAVEQGKRE-----
----
>Reyranella/1-587 soli
-------------------------------SQKINVSHGFAMHG---APKYAADAGPPDYLNPNAPKGGSV
RLGARG--------TFDSLHPFII-------KSVPAAG-VSA-----------IWDTLCWNSRDE-------
--ASTEYGLIAESIEWPEDR----SWAAFTLRPQARFHDGSPITVEDVIWTFDTLKAKG--LPNYAFYYGDV
LKAEKVGDRKVMFTFR-DNTNKELPLIIG-QLPVLPSKWWA-------TRDFEKVSLE-IPLGSSAYKVDSF
DVGRSITYRRVEDWWAKDLWMNRGRNNFEVMRYEYYRDVTVQFEAFK-AGEIDLRQEN--IARNWATAY-DI
PAVRDGRIQRAEIPH--ELPTGMQCFAFNTRRDFFKDKRVREAISTMFDFAWTNKNLFYGMYKRNISFFGNS
ELAS----SGLPTAAELKYLEPLR----GKIPD------EVFTKEFKLPESDGT-GNV----RDLARRALSL
LKEAGWEVK---DGKMTE
TKTG--KKLAFEMLLND---ASFERVVLPYKQNLERIGIDMNVRTI-DTAQFKRRED---EFDYDMMVEG-F
GQ----SLSP-GNEQRDFWGSKAADTKGSRNSIGIRDAAIDNLIETLIGAPDRES-LINATRALD-RVLLWS
HFVVPNWHS---------NTAYVAYWN-RFARPAKA--AKYSPVAF--DTWWIDEAKDKALQRDKK------
----
>Paraperlucidibaca/1-586 baekdonensis
-----------------CCFLAMGLFGMS-AQAAVQHSHAITLRG---APALAEGFRHFNSVNPDAPKGGRL
RLDA-LG-------TFDSLNGFIN-------KGVAAEGLN------------RLYDSLTAGSEDE-------
--FGTRYGLLAKRIIRDPNDA---SWVIYELRPEARFSDGHPLRAEDVVFTFETLLSEG--APAYKAYFADI
ASVTALSPLRVKFRFKHKT-NRELPMTVG-EIGILPKHYWA-------TREFNRTSLE-VPIGSGPYVISDI
DPGRRISYARNPDYWARDLNVNRGRYNFDQISYQYYRDGSVAFEGFK-ANQYDVREEN--KAKTWATEY-NF
PAVTDGRVILQRQRH--ENPAPMQGFAFNTRRTPLNDLAVREALSLAFDFEWANRALFFGAYTRTTSFYANS
DLAA----QGVPSKAELALLKPWR----KQLPA---AAFG---PAVTPAKSAGD-GYD----RANLLRAQAL
LKQAGWHYRD---GALRD
VAGT---PMRLEMLLVQ---PEFERIVQPLRRNLARLGIDLSIRLL-DVAQYIERLR---QFDFDLTVTG-V
PA----SLSP-GNELWSYWGSQSADTPGSGNLVGLKSPAVDALIARITQAKSRDT-LVTSVHALD-RVLRAQ
WLLIPQFHIP---------FFRIARWD-FFGLPNDP--PRYGLG---LDSWWW-------------------
----
>Kushneria/1-592 marisflavi
----------------LAVSGVQAAPVSD-----VPTVHGISLYDT---PELPSDFDHFPWVNTQAPRGGDM
VRSAMG--------SFDSLNPFIV-------RGDPASGLTAFG-------G-LPFDTLMVESPDE-------
--PFTLYGLLASGIRLDPDR----RWMEIDLDSRARFHDGSPVTAEDVVTTFETLEAQG--SPMYRAYYADV
TDVEVVSPFTVRFEFSEHNSR-ELPLILG-QLPVLSAKDIE-------RRDMTRPTLE-PLMGSGPYRISDV
QPGQRIVYERDDDYWGRDLPVNRGRYNIDRLIFDYYRDPSVALTAFR-AGRVDLRIET--IARNWVTGY-DF
PAARQGLIKRVEIED--HKPAPMQGFVMNLRRDVFKDRRVRRALGLAFDFNWLNRNLFYGLYARTHGFFDHS
SMEA----KGLPDDAERELLTPWR----DQLP------EEVFKTP--LPIKAPQ--AL----RPRLKEALEL
LREAGYEVRN---NRMVN
RETG--RPLRFELLLGS---SSIERMALPMVRNLARLGIEVTLRTV-DQSQYLVRLR---QFDFDMTTTV-I
AQ----STNP-GNEQREYWGSEYADQPQSRNLAGISSPAVDDLIDRIIRADSRKA-LDTAASALD-RVLRWG
FYVIPQYHSP---------VTRLAYWK-KMAMPDQSPE--YGLD---PDSWWIDPQGARQI-----------
----
>Camelimonas/1-583 lactis
--------------------------------------HALSLMG---QPKYGADFTHFDYVNPAAPKAGVV
RFGSQG--------GFDSFNLAVAGV-----KGEVEDRLT------------LIYDQLMEPSLDE-------
--VNTEYGLIAEAARHAGDY----SWASYRLRPEARWHDGRPVTVEDVIFSFESLKKL---SPQYAAYWRDV
TAVEKTGPREATFRFATTG-NRELPQIVG-QLPVLPKHWWEGTDASGRKRDIAATTLE-PPLGSGPYRIKAF
DAGRWCEYERVKDYWGEKLPIRVGMHNFDVVRVDYYRDITVLFEAFK-ADQFDFRTEN--VAKNWATGY-DF
PAVREKRVILEEFPQ--RSSGVMQAFVFNMRRPQFQDERVRRALNYAFDFEEVNRTLFFGQYKRIDSYFSGT
-ELAS---SGLPHGLEKEILESVR----DKVPA------SVFTEVYKNPVAGSQ-EAF----RANLREAHRL
LGEAGWVLKG---RNLVN
SKTG--APFVIEFLS--FD-PSFERYVLPYQNALKRLGMNVNLRVV-DATQYQNRLR---AFDFDAVTFL-W
PE----SLSP-GNEQWNFWGSEAAKRPGSMNVAGISDPGVDALIGKVVYATTREE-LVAATRALD-RVLLHH
NYVVPQWTY---------NFQRTARWN-RFGRPQ--TMPEYGAA-AFPTIWWWDAALAAK------------
----

>Minwuia/1-594 thermotolerans
--------------------LFAVPATAGAQDD-IVRSHALSLIG---EPQYERGFDHLDFVNPDAPKGGTL
RMSAFG--------GFDSLNPYIP-------KGNPAGAAS------------LAVETLMDQHLGE-------
--PSTEYGLIAESVEHPKDH----SWVIFNLRPEARFHDGSPVTAEDVAFSFEVLRDKG--QPFYRYYYRNV
TEAKVLDTHRIRFDFSETG-NRELPQIMG-QLPVMSKAYWAD-------REFGATTLE-APMGSGPYRVSAV
EPNRRVVLERVEDYWGRDLGINRGRYNYDRVQYEYFADRTAVLEAFK-GYVYDYRTEN--SSKDWATAY-EF
PAVERGDVKVELVPH--SRPTGMQGFIYNLRRPLFQDPALREALSYAFDFEWANKNLFYGQYERTESFFSNT
ELAS----QGLPSEAELELLEPLR----GMIPE------EVFTEPYSPPSTAEN--SL----RENLGKAFQI
LREAGYKFEN--KTLL
TPEG--EPVSFEILLAD---PQFERIVLPFIENLRRLGVDASLRTV-DAAQYQNRV---RDFDYDMVVGS-Y
PQ----SESP-GNEQRDFFGSEAAERPGSRNLAGIRSEAVDRLIDNIIFAENREA-LVTATRALD-RVLLHS
HIVIPQWHI---------SALRIAYWD-KFGRPP--VDPDYGVD---VFAWWILPEKAAELAETYK------
----
>Rubritepida/1-597 flocculans
-------------------VGLVAFSGRAMARQGTIRTHALSLLG---EPALPPGFAHWPWVNPHAPKGGEV
ALTAIG--------SFDSFNGFIL-------RGTPAVG-LVN-----------LYDSLLQESADE-------
--ASTEYAHVAEVVELPADR----MGVSFELREGARWHDGRPITAEDVAFTFNTLRSQG--RPFYRAYWSDV
SEVLAESPRRVTFRFR-TNENRELALILG-QMQILPRHFWE-------GREFARPSLD-IPLGSGPYRLERF
EPGRTVIYRRVEDYWARDLNTRRGQHNFDAMRYEYFRDVTVAFEAFK-AGQIDFRTEN--IARNWATGY-DF
PAARRGLVRRDEIRH--ELPTGMQAFVVNLRRPLFQDARVRQALIEVFDFEWLNANIFNGLYNRTTSFFSNS
ELAS----SGLPTGRELEILQRFR----GRIPE------SVFTTEHRLPVTDGS-GNN----REGARRALAL
LREAGWTVR---DRRLVN
AQG---QRFSFEILLNG---ASFERIALPYVQWLERLGIEARVRTV-DPAQYQVRID---AFDYDMTVDV-M
GQ----SLSP-GNEQRDYWTCEKARQNGSQNVAGICDPVIDELVELLITAPDREE-LIHRTRALD-RVLLHH
HFVIPQWHN---------RRFWIAFWD-RFGRPERN--PRYGLGF---DSWWIDPTRDRALAEARR------
----
>Azorhizobium/1-573 oxalatiphilum
--------------------------------------FATSLMG---EPKYKDGFKHFDYVNPEAPRGGTV
RLSADG--------TFDSFNFIIP-------RGTSGAGLS------------QIYDTLTTQSRDE-------
--VATAYGLISDGLRFPEDY----SSVTYRLNPNAKWHDGQPITPDDVVWSFEVLTKN---NPGQAFYYRHV
KGVAKTGDHEVTFTFDQGG-NRELPQIVG-ELLILPKHWWTGTDANGKPRDITQGTLE-PPLGSGAYKIKSF
VPGRTIVYERVKDYWAKDLPVNVGRNNFDEIRYEYYRDDTVELEAFK-ADQYDFRVET--SAKNWATAY-DF
PAKQQGKVKLELFEN--RSVGQMQAFIPNLRREKFQDARVRRALNYALDFEGMNHTLFFDQYKRTMSYFSGT
ELAS----SGLPTGAELDILNGVK----DKVPP------GVFTTPYTNPENPDE-QSR----RANLRQAADL
LKQAGWQVK---GRQLVN
AK-G--EPFTLEILLA--A-PAFERVALFYKPALERLGIQVSVRLV-DSSQYTNRVR---ARDFDMIITG-W
GQ----SLSP-GNEQRDYWGSESADREGSRNYAGIKNPAVDALIEKVIYATDRTQ-LVAATHALD-RVLLWN
DYVIPSWTF---------GYSRTARWD-RFAHPP--VLPQYSYE--FPDIWWWD------------------
----
>Rhodoligotrophos/1-580 defluvii
--------------------------------------HGSSLFG---DLKYGPDFQHFDYVNPQAPKGGRV
RFQALG--------AFDSLNPFTY-------KGRPATAIV------------YAFDALTMPSMDE-------
--PSTSYGLIAEAIKFPADF----SQVTYRLRKEARFHDGQPVTPADVIFSMEAIKKG---LPQYAAYYRNV
VRGEQTDDNEVTFFFDQKG-NRELPNITG-QLIVLSKHWWEGKDASGNQRDITTTSLE-PPLGNGAYRFERM
NVGRSIVMKRVEDYWAKDLPVNRGTNNFDEIVQEYFLDPAVAFEAFK-ADQYDWRTEN--SARNWATGY-NF
PAAQRGDVVREEFKF--DNPAPMQAFAFNTRRPKFADARVRLAFNYAFDFEWANRTLFYGQYKRPDSYFAGS
ELASS---G-LPQGKELALLEPLR----EQVPP------QVFTEPYSNPVNATP-AQQ----RENLRKAMML
LNEAGWTFDK---GALKN
-AAG--EVLSVEFLLD--D-PALERVVLAYVQSLKRLGINATIRTV-DTAQYQARVN---TFDFDVITHI-W
AQ----SLSP-GNEQRTYWGSAAADEPGSDNVVGIKNPAIDTLIDKVIYAESRED-LVAACRALD-RVLLWN
HYVVPQWYS---------PLTRTARWD-RFGHPE--IIPKYQTG-FPT-VWWYDAEKAAKI-----------
----
>Sediminicoccus/1-598 rosea
------------------LVSFSAALPGAAQTTAPLRTHALSLLG---EPALPADFPHFPWVNPDAPKGGDI
ALTALG--------SYDSFNQFIL-------RGTAAVG-LNN-----------LYDSLLMESADE-------
--ASTEYAHLAEIVEIPADR----MGVSFELRDTARWHDGRAITAEDVAWTFNTLRSQG--RPFYRAYWGDV
SEVVVESPRRVTFRFR-TNENRELALILG-QMNILPKHFWD-------GRDFARPTLD-VPLGSGPYRIDRF
ESGRSVAYRRVADYWAVNLNTRRGTQNFDVMRYEYFRDVTVAFEAFK-AGQIDFRTEN--IARNWATGY-DF

```
PAVRRGLVKRDEIRH--QLPTGMQAFVMNLRRPLFQDARVREALLQVFDFEWLHTNIFNGAYARTTSFFSNS
ELAS----SGLPAGRELAILEPFR----AQLPE------SVFTTEHRLPTTDGS-GNN----REGLRRALDL
MRQAGWTIR---------------------------------------------------------DRRLVN
AQG---QRFEFEILLNG---ASFERIALPYVQWLQRLGVEARVRTV-DPAQYQVRTD---AFDYDMTVDV-M
GQ----SLSP-GNEQRDYFTCAKAQENGSQNIAGICHPVIDALVEQVIMAPDRQE-LIARTRALD-RVLLHS
NFVIPQWHN---------RAFWIAFWD-RFGRPERN--PKYALGL---DSWWIDPTRDRALQEAKR------
----------------
>Mesorhizobium/1-615 onobrychidis
------------------MLVVAAFAGGLQAAFANEWRTTSSLIG---ESKYGNNFQHYDYVNPDAPKGGTL
NSVVLG--------TFDSFNPYIV-------QGSFAAGF-------VPFGGGLLYDTLMEQATDE-------
--GSVSHPLIADAYKHPDDY----SSATYRLDPRAKWHDGKPITTDDVIWSFQVLKTN---SPMYSRYFENV
TDAVAVSDREVEFHFDQKG-NRELPKILG-DVVVLPKHWWEGTDATGKKRDITRPTQE-PPLGSAAYKIASF
KPGSEIVWQRVPDYWGAKLPVKIGRENFDRQRFSYFLDDNASWQAFTKGGFEDFRVES--RAQRWAVEY-TF
PAIKAGDVIKAEYPT--TSPEPMQAYVMNTRRPLFQDVRVRQALTYAYDFESMNRTIFFGAYTRTDSYFEGG
DLAS----SGLPQGKELEILEPYR----DKLPP------ELFTQEFKLPVYSTP-QSG----RENLRKAYDL
FKQAGWVNRG---------------------------------------------------------GRLVN
EKTG--EPFRIEFLGN--D-PVDERVAGPLIDNLRRLGIDATLRIV-DDSQYTNRTR---AFDFDMLAVAGF
QQ----SNSP-GNEQRDFFSSTAADTPGARNLAGIKNPVVDALIDRVIFATDRDD-LVAATHALD-RVLLWN
YYMIPQWHL---------GKIRIAYWN-KFGIPE--KQPTYSGVD--QNSWWIDADKEKALAAKYKSG----
----------------
>Tistrella/1-573 bauzanensis
-------------------------------------AHGLSMYG---DLKYPSDFTHFAYAYPDAPKGGTL
KTAARG--------TFDSLNPFT-------LRG-ISAGVG------------STFDTLTVQSLDE-------
--PFSEYGLIAETIDIAPDN----SSVTFTLRRQARFHDGSPVTAADVAFTFDTLKTKG--HPMYRIYYAEV
EAAEVIDDHTVRFSFSNTS-NRELPLILG-SLPVLSKAFFET-------RDFTATTLE-PLLGSGPYKVASA
DPGRSLVLERVKDYWARDLPVNVGRHNFDRQVVDYYRDDEVSVEAFK-AGQYDLRLEN--SARRWATAY-NM
PEVESGKLQLAVIPH--RISVGMQGLVMNTRRPKFQDPALRRAMQYVFDFEWSNKTLFYGAYTRTDSYFE-N
SSMAS---SGVPEGDELALLEPFR----DQLPE------ALFTEPYTVPRTDGS-GSN----REGLKAAYEI
LQDAGYEVRN---------------------------------------------------------GKLYA
PDAK--EPLRIEFLID--D-AATERIVMPITRSLGRLGIDANVRMV-DAAQYENRLQ---TFDYDITTEV-W
AQ----SQSP-GNEQREYWGSDAADRPGSRNYAGIKNPVVDALIQKIIDAKDRSS-LVTAVKALD-RVLLWG
DYVIPNFHL---------PAFRLVYWN-KFGRPQ--VLPDYGID--L-DTWWVDQALARK------------
----------------
>Halotalea/1-599 alkalilenta
-------------LTLLAVAPVTWAASPEQ----VPTVHGLSLYGE---PGLPAGFTSFPYVNPNAPKGGSL
TRAAPV--------SFNSTNPFII-------TGSAASGIDYLG-------QSYLYDSLMVSDPAE-------
--IFSAYGLLASGIRLDPQR----RWMEFDLDPRARFHDGEPVTAEDVVFTFNLLVEKG--APFFRGYYADV
TSVRAEGPLTVHFDFAEHNSP-ELPLILG-QLPVLPAHYWK-------DRDFTRPTLE-IPVGSGPYRIERL
DPGRQIVYRRDPDYWARDLPVNRGRFNIDRLIFDTYLDDSVALEAFR-AGNVDLRVEM--TASNWATGY-QG
PAMERGLIGQLVVAN--HNPAPLQAFVPNLRRAQFQDPRVRRAIGLAYDFEWQNRNLFYSQYRRTRGMFDGS
EMEA----TGLPEGEELALLEPLR----DQLP------PEVFEEP--LPIGEPS--DL----RERLRQALAL
LEEAGYRVEN---------------------------------------------------------DRMVD
-AQG--HSLSFEILLWD---GRMQRMVLPYVRNLRRIGIDARVRVV-DPSQYQVRIS---QHDYDMIIGS-F
AQ----SSSP-GNEQREFWTSDYADRPQSRNTAGIRNPAIDQLVESLIRADSRES-LDAHARALD-RALRWN
FYLVPQYHNP---------GTRIAYWR-KLAFPLPFPD--YGLD---LDAWWVDSQRASRVEAA--------
----------------
>Thalassospira/1-581 mesophila
-------------------------------ASLARPEYAISLYG---DLKYGPEFTHFDYVNPDAPKGGTL
LDSSTG--------SFDTLNPFIL-------KGDAAIGLG------------LMYDTLLVQSMDE-------
--PFSEYGLVAQKVEVPQDR----SYATFFINPAARFSDDTRITADDVIFSFNTLIEKG--NPTYRQYYASV
DHLEKLDDLTVKFVFK-PGDNLELPLILG-DIPVLPAHYWK-------DKDFSKTTFD-IPVGSGPYMIKDF
EPGRHITYVRNKNYWAKDVPSVKGMYNFDTIRYDYYRDLDVERQAFF-AGEYYYRGEF--SSREWATGY-DK
PPVRNGNIKKDLIPN--HQPRGMQGFIMNARRPLFADHRVRRALQYAMDFQWLNRAMFYGAYQRTDSYFVNS
ELAA----TGLPHGEELDILNKFR----DKLPP------DLFTKPYSLPDYDQE-NGR----RKALREAMTL
LNDAGWELRD---------------------------------------------------------QKLVN
KKTG--EPFRFSLMLRQ---PGFEKIALMMQTRLRQLGIEMDFRLI-DSGQWINRLQ---SYDFDMTSFW-W
QQ----SLSP-GNEQRFYWSSAAADQPGSRNFAGIKDPVVDQIIDLVISASNRDS-LVQRTRALD-RVLLWG
DYTIPQWYQP---------ADRLAYWN-IFGRPSVVPTKGNSMM-----TWWIDPDKSKKL-----------
```

>Stappia/1-614 stellulata
------VKILPLFLGLLAFVLVSAGGDTARAQEDPAWRHAGALTG---DPAYPEDFRHFDYVNPDAPKGGTV
RLSDPS--------GFDTLNPVL--S-----RGNPAPGLG------------LMYDSLLEPALDEI------
-DINAEYGLIAEAFRYPEDY----AWVEYRLNPEARWHDGTPISVDDLLWTFEKLIEV---NPSQAFYYRHV
AKAEAVGERTVRFTFDAPG-NRELPKIVG-QMIILPKHWWTGTGANGQPRDIASSTLE-LPLGSGPYRLKAM
EPGRFVVYERVKDYWAKDHPARVGTNNFDKVRYDSYRDQTVLLQAFK-ADQYDFRAEN--SAKNWATGY-DF
PAVKQGRVVLETFPD--KARGMMQAFVMNLRREPFDDLRVRQALNLVFDFETANKTVFFDQYKRIPSFFAGT
-ELAS---SGLPEGKELEILESVR----DLVPE------RVFTEPYENPVNGDP-ARL----RANMRKAVAL
LKEAGYELRG---RQMVN
VETG--APLEIEFVD--ND-PNSERYVLPYARNLERIGIKMTLRVV-DTPQYVNRIR---ERDFDMTTLG-W
AQ----SLSP-GNEQRDFWGSEAADRAQSRNYAGIQDPGVDALIEKVIFASDREE-LVAATRALD-RVLMAH
SYVVPQWYI---------DVDRTARWN-RFSHPE--NLPEYSH--GFPSIWWWDAEKAAK------------
----
>Ketobacter/1-603 alkanivorans
---------------LLILAISLNGFARNTAAADVLVATAIALYD---QPKYPQGFQHFDFVNPEAPKGGKI
TLPALG--------SFDTLNPYVL-------KGISPSEFS--A----MYGITQLNEPLMMGTHYYLES---G
DEPQTAYCLICEWIEYPADY----RWVIFQINPKARFHNGDPITAADVAFSYKLLMTD-QAHPMFPNNLAAV
ESVEVLDTHRVRFTFK--GQPERSNLLRVGEIPVMSKRFWESH-------SFGASSGT-PQPLSGPYRVSDF
VLGSYVTLQRVDDFWAKDHPVYQGMFNFDEVRVEFYRDRTVAFEALK-SGGVDFWVEN--VSKSWATGY-DF
PAVRDGRIIKEELQH--SIPSGTQAFFLNMRRDVFKDVRVRKAISLMFDFQWTNRNIFSDAYARNNTHFPNS
EMGAT----GLPSAAEKALLEPFR----KQLPHA------LFTEEFKYPRYDSA--KD---LRQAMREAIGL
LKQAGWVYNN---KRLVN
AETG--EPLRFEMLINS---PSFQRVLHPYVKNLSKIGIEANIRIV-DRAQYKVRLD---DFDFDMTVYV-L
PQ----SASP-GQEQRLYFHSAQANVRGAKNLSGIQDPVVDALIEQITAAKSRND-LIAAVRAMD-RVLLWN
YYTIPHWHLG---------YHRLAYKN-TFARPETSSAMT-----LGFQTWWLN------------------
----
>Aidingimonas/1-595 halophila
------RYALLFIVIPWLLPASLLADPAED----VETVHGLSLYDE---PALSEDFDHFPHVNPDAPKGGTL
RRAATG--------SFDSTNPFIT-------SGTSASGLT------------QTYDTLLVANPDE-------
--PFSMYGLVAEGIRLDPDR----RWMEFDLRSEAHFHDGEPLTADDVVFSLETLVEKG--QPFYASYYADV
TDAKAMDDHTVRFEFAEENSR-ELPLILG-QMPILPEHFWK-------DREFNRSTLD-PLLGSGPYRIDEI
DPGRRITYRRVEDYWGRDLAVNQGRYNIDRLIYDYYRDQTVALEAFK-AGNLDMRQET--SARLWATAY-DF
PAAEDGYIERLIIPD--GQPAGMQAYVMNLRRETFQDRRVREALNLAFNFPWLNDNLFYGAYEQTHSFFENS
EMAA----EGMPSEEELALLEPHR----DELP------DEVFDEP--LPIDHPE--DR----RERLKKAYEL
LLDAGYEVRD---GVLVD
TENG--RPLRLEVLLYD---SQFERVTQPLLHNLERLGIQGNIRLV-DVNQYLNRLS---NFDFDMIVGS-F
PQ----SANP-GNEQREFWTTPYADRPRSRNLIGLRDPVIDTLVEQLIRADSREA-LDTAARALD-RVLRWG
FYVIPQWHMS---------GTRIAMWD-KFEWPEPFPE--YQRD---MDAWWIDPER---------------
----
>Neoroseomonas/1-589 soli
----------------------------GDRPGEVVRTHALSLLA---PPSLPADFPHWPWANPNAPKGGEV
VLARLG--------AFDSFNPYIL-------RGTPDIG-VGL-----------IYDSLMAGNPDE-------
--ATAEYGHVAETVELPADR----KGVTFELRAAARWHDGRPMTAEDVVFSFNALRTHG--RPFFRAYWADV
TEVVAEGPRRVTFRFR-SADNRELAQILG-DLPILPKHWWE-------GRDFARPSLD-VPLGSGPYRLERF
EPSRSVVYRRVDDYWARDLGVRRGLNNFDTIRYEYFRDTTVAFEAFK-AAQTDFRQEN--VARDWATGY-DF
PAARRNLVKREEIPH--EIPTGMQCFAVNLRRPLFQDARVRRALIEVFDFEWMNTNLFYGAYTRTSSYFSNS
DFAA----RGLPEGREKAILEGFR----GRIPG------TVFTEEYKLPVTDGS-GNN----RDGARRALAL
LREAGWTVR---ERRLVN
AQG---RPFEFEILLQG---ATFERVALPYVQWLERLGISARVRTV-DPAQYQVRID---AFDYDMTVES-M
GQ----GFSP-GNEQRDYWTSAKARENGSQNVAGIADPAVDELVELVVGAPDHEE-LVARTRALD-RVLQHN
NFVIPHWHS---------RTFRIAFWD-KFGRPERN--PRYGLGFP--GAWWIDPARERALAEARR------
----
>Rhodovastum/1-610 atsumiense
--------MRIIALITALLACAPVAGAPPAHAEAPTRSHGVSLLG---APALPPDFPYFPWVNPDAPKGGEV
ALGAVG--------TFDSLNPFIV-------RGRAAGA-AGR-----------IYDTLMVRNFDE-------
--AQTEYGNLAGVIEIPADH----MGVAFELRPEARFHDGTPVTADDVVWSFNTLRDKG--RPNYRQYYGDV
TEAVVEGPRRVVFRFR-THANRELPMILG-DLAVLPRRFWE-------GREFDRPLTD-VPLGSGPYRIGKV

DFGRSITLERVPDWWGRDLPVAKGLYNFDRIRTEYFRDGTVAMEAFK-AGQIDYRSEN--IAKNWATAY-DF
PAVQKGLVRKESIRH--RLPTGMQGWAMNTRRPLFQDRRVREALALAFDFEWANANLFYGEYTRTESYFSNS
DLAS----SGTPQGAELALLDKWR----GKLPP------ELFTQPFRLPVTDGS-GNN----REGLRRGLAL
LKEAGWTVK---DRKLVD
AQG---RPFTFEILLSE---PTFERVALPYKQWLSRLGIEASVRTV-DAAQYQRRLD---TYDYDMTMMV-I
PQ----SESP-GNEQVGFWTCGAVNLEGGDNLMGVCDPVVDALVPQVVTAADRTQ-LLTTVHALD-RVLLWG
WYMVPNWHL---------QSVWIAYWD-RFGRPSAP--VRPGVVF---DSWWVSPEKDTALQAARRAG----
----
>Sulfurivermis/1-560 fontis
---PAYAPGFAHFNYVNPEAPKGGSV
NLAAVG--------GFDSLNPFTL-------KGQAAAGLTE-----------LMFETLMESS---------L
DEPFSQYGLLAEDVELAPDR----RSVTYRLNPRARFHDGTPVTAADVKFSFETLRD--KGHPQYRFYWSDI
ERAVVLDERIVRFEFA--RVNPELHMIAG-QIPIFSRAWVGDS-------DFDKMALV-EPLSSGPYRIEKY
ELGKYITFVRNPDYWARDLNVRRGMFNFDRVTFKFYRDFTVALEAFK-AGEFDFNVEY--NSKTWARDY-VG
SKFRDGRIVKTELEH--HNNAGMQGFVFNLRRPQFRDIRVRRALSLAFDFEWSNRQLFYNQYQRCDSYFSNS
ELASR----GVPTGAELALLEPFRAQ----LPAE------LFQ-RPWLPPSTDP-PHS---LRENLRDAKAL
LHAAGWEYR--DGALR
NGQG--EPFQFDIMLVQ---KGFERIVAPYARNLGKLGITVSYRTV-DPALYQRRID---TYDFDMVVAG-F
GQ----SQSP-GNELMGMFHSSAATQEGTRNLIGIQNPVVDALVEQVIYARDRAA-LVTAVHALD-RVLLWG
EYLVPHWYIG---------THRVAYWD-RFGRPERLPLYY-NAANWMLTTWW---------------------
----
>Elstera/1-584 cyanobacteriorum
---------------------------------AAEPVHGIALYG---EPKYKPGFSHFDYVNPNAPKGGEV
RQAVLG--------GFDSLNPFIL-------KGNAAAG-LAL-----------LFDTLTIASDDE-------
--PFTRYGLLAETMEIAADR----ASITFTLRADAKFHDGSPVTVQDVAFSYKMLKEKG--SPHYRSYFGDV
TAVETPDARTITFRFK-DGTNRELPLIIG-EFPVFSAGYYA-------NRPFDQTSLD-APLGSGPYKVKAV
DANRSITYQRDPNYWAKDLGPQKGHHNFDTLRYDYYRDDGVALEALM-AGDLTLRQET--SSSRWATAY-DK
PPVRQGLIQRTEIGD--SSPRGMQGWVYNTRRPIFADRRVRAALAYAFDFEWTNKTLSDGKLIRTASYFDNS
ELRA----RGLPQGDELALLEPFR----GKVPD------EVFTQEYLPPQTDGS-GNI----RDNLRTALRM
LREAGWTVK---DNKLVN
-AEG--KPLTFEILIDN---QAFERTTLPFIKNLEQLGVTATLRLL-DVAQYQKRIE---AYDFDMMIGI-F
PQ----SISP-GNEQRDFFGAHAADIPGGRNLAGIKDPAIDALIEQVIYAPSRAA-LVTRTRALD-RLLRWG
HYVIPQFHG---------RTTRYAHWD-FYGRTG-V--ETLQRESF--DRWWWDAEKAAATQAKKGK-----
----
>Chelatococcus/1-584 composti
------------------------------------EVHGLSAFG---DLAYPADFKHFSYVNPDAPKGGTL
SLQIKQAGGNQNFDTFNTLNIFVL-------RGDGAAGMDLT------------YDTLMAGS---------G
DEPDALYGLVARAVRISPDE----LTYRFLLRPEARFHDGSRITAEDVAFSLDILKTKG-HP-NYRAILTQV
VGAEVEREDIVAIRLSPQRSRDLHLVVAS--LPVFSKAYWSGR-------DFEATTLE-PPLGSGPYRVGRF
EVGRFIEFERVEDYWGRDLPVNVGVNNFDRIRYEYFRDRQVAFEAFK-SGVFNYHEEY--TARIWATGYD-F
PAVREGRVRREEIPT--RSAVPTQGWYFNTRRPQFRDPRVREAIGLAFDFEWTNKNIMYSAYKRLTSFFEAS
-EMKAT---GLPSPEELALLEPF----RAELPAE------VFGEPYVPPVSDGS---GS--DRALLRRADEL
LREAGCK-R---DGRR
LLLPDGQPFTIEFLDFQ---PTLQPHTQPFQENLQRLGIEARSRIV-DAAQYRRRIDD---FDFDMTSM---
--ALSGSLTPGDS-LRVVYGSEAARTPGSRNIAGIAHPAIDALIERIGRARTRQE-LTIACRALD-RILRAG
RYWVPMWYKD---------KTLVAYWD-VFSRP--DKTPKYGS--GAPGTWWYDAEKAKRI-----------
----
>Futiania/1-587 mangrovii
--------------------------GASAEP-----RHGLSAFD---ALKYPADFPHFSYVDPAAPKGGEI
STIG-----TAAVTTFDSLNYWIL-------KGDPAQGLPLL------------FDTLMVRA---------E
DEPDAVYGLLAESAEVSADG----RAVTFRLRPEARFHDGTPVRADDVVFSFEALKEKG-HP-DFRLSLRDV
AAAETVDALTVTYRFEGDNLRDLPITVA--TLPVLSRAYYETQ-------PFEETTLE-PPVGSGPYRVGDF
KAGSFIVYERDPNYWGRNLPVNLGRHNFDRIRYLYFRDRVAGMEAFK-AGAYTLREEF--TSKTWATEYD-F
PAAEAGLVKREVLPD--ARPSGTQGWFLNTRRPHLADWRVRKALDYAFDFEWTNATLFYGAYSRTDSFFENS
-DMQAE---GPASAEERALLEPF----LAHLPPG------ILDAPYVPPLSDGS---GR--DRGNLREADRL
LREAGYTVQ---DG-R
RTMPDGRPFRLEFLMFE---PSFERVNAPFLQNLERLGIEAAMRIV-DPAQYQRRLKS---FDFDIVTS---
--RFVLRPTPGAE-MRNFWTSAAAATEGSRNYAGIASPVVDALTERMIAAGSRAE-LATAARALD-RVLRAG

HYWIAHWYSA---------SHRIAYWD-MFGRP--EAKPAYGR--GILDLWWVDPEKKAALDAA--------
----
>Xanthobacter/1-581 agilis
--------------------------------------HGLSFFG---DLKYPADFTHFDYVNPEAPKGGPF
SQMGRATFYNQSLSTFDTLNPYNQ-------RGNGAQGIELI------------YDTLMTAA---------G
DEKSAMYPRLATAVEIADEG----LTYRFPLNPAARFHDGTALTAADVVASFAALKADG-YE-TIRMALRDL
IAVEPDGDQAVVMRFKPGRARDVPLTAAG--MPIFSKAHLAHH-------PFNQSSLE-LPLGSGPYKVGRF
EQGRFIEYDRVKDYWGARLPALTGRFNFDTVRYEYFRDRIAGFEGFK-AKAYLFREEF--TAREWATQYD-F
PAVRDGRVKLETLPD--ESFSGVQGWFFNTRRPQFFDRRVREAIGLLFDFDWTRRNLMYDAYHRTVSFFQNS
-DMMAQ---GLPSPAELKLLDPF----RAQLPEE------VFGEPYAPPASDGS---GE--DRALRLKAVAL
LKDAGYL-I---RQGK
LVNAKGEPFTMEFLDDD---GAMERHHARFIGHLKKVGIDATFRIV-DPAQFQARLQA---FDFDVVVR---
--RYSFAPYPGDE-LRAYFSTEAARTPGSYNVAGIADPVVDALIAQALAAGNADA-LVTCCRALD-RVLRAG
HYWIPQWYSG---------ENRIAYWD-VFGRP--DVKPRYEL--GVLDTWWSTAPGAAK------------
----
>Micavibrio/1-568 aeruginosavorus
-----------------------------------EEYYGLAMHG---TPKYGPDATHLDYANPDAPKGGTL
TQAAIG--------SFDSLNPYAIKG-------TAAQGLA--------FVH----DRLMARVWDE-------
--AFTLYPLIAEKVDVPEDR----SSITIYLNKAAKFQDGTPITADDVIFTFETLRDHG-RPN-MRRVYKLV
KTVDRIDAHTIKFAFGEGYDR-ETMMIIA-MMPVLSKAWWS-------GRSFDSATLD-IPVASGPYKITSV
EPGRKIVYERDPNYWGANLPVNVGHYNFDRIVFDYYRDDSVAFESFK-SGGMDLRREW--DAGKWAKG-YDI
PAVTRGEIVKEALPH--GRPDRTNALIFNTRKAPFDDIRVREALSLSLDAPWINQNMFHGLYKRIDSYYPNA
ELAAP---NTAPDAAELALLEPHR----AQLRA------SVFEADTPDPTG-----------RDAMLRADTL
LKEAGWIVKDG---VRVN
EKTG--APFTFEIMLSS---PDDEKLALSFTRGLKRLGIDVRVRVL-DSAAFLGRLN---EYDYDMVLYFWL
S-----TLSP-GSEQILYWGCEAAKQPARWNYAGICDPVVDALSMQIAAAKDRDG-LVTAARALD-RVLMSG
RYMIPLFHPG---------ADFVARRA-SLKHPETM-PLYGMV----LETWWDESGK--------------
----
>Afifella/1-601 pfennigii
---------------------------AQEVQEGTQWRHAASLIE---APKYGPDFARYDYVNPDAPKGGTL
NQVALG--------TFDSFNPFLI-------RGTPAAGLGQI---VNARGG-IGYDSLMEPSTEE-------
--PSTSYGLIAEAMTYPDDY----SSATFRLNPKARWHDGEPITPQDVVWSFETLKEI---NPQYNQYYHNV
VTAEVTGEREVTFTFDEKG-NRELPHIMG-DLVVVPKHWWTGTDASGKARDISQPTLE-PPLGSGPYRIGSW
DAGRNVVWERVPDYWAAEHPLRVGRYNFDAIRYEYFRDLNAAWEAFTKGGIYDFRAEN--RAQRWAQGY-DF
PAAQRGDVKKDTFAT--TSGEPMQAFVLNTRRDKFADRRVRQALTYAFDFEALNKNLFFDLYTRTDSYFEGT
ELAA----SGLPEGKELEYLEKVR----GEVPE------EVFTEEFKLPVNDSR-EAM----RANLREAVRL
LGEAGFEQRG--GQLVD
GETG--EPFTVQFLGN--D-PSDERIFGPYAASLRRLGIDTSIRIV-DAAQYQALSD---EFDYDII-SGVF
GQ----SQSP-GNEQREFWGSVAADQPGSRNAAGIKSEAIDKLIDDVIYAPDRDS-LVAATRALD-RVLLWG
FYMVPQWHN---------PEIWLAYWN-KFGIPD--EQPSYSGVD--IFSWWVDPEKERQI-----------
----
>Camelimonas/1-578 fluminis
---MG---QPKYGADFKHFDYVNPAAPKAGVV
RFGSQG--------GFDSFNVAVAGV-----KGEVESRLT------------LIYDQLMEASLDE-------
--VNTEYGVIAAAARYPEDY----SWASYRLRPEARWHDGRPVTVEDVIFSFESLKKL---SPQYAAYWRDV
SAVEKTGPREATFRFAAKG-NRELPQIVG-QLPVLPKHWWEGTDASGRKRDITATTLE-PPMGSGPYRIKAF
DAGRWCEYERVKDYWGEKLPVRVGMHNFDVVRVDYYRDITVLFEAFK-ADQFDFRTEN--VAKNWATGY-DF
PAVREKRVILEEFPQ--RSSGVMQAFVFNMRRPQFQDERVRRALNYAFDFEEVNRTLFFGQYKRIDSYFSGT
-ELAS---SGLPEGLEKQILESVR----DKVPA------SVFTEVYKNPVAGSQ-EAF----RANLREAHRL
LGEAGWELKG--RNLVN
SRTG--TAFVIEFLS--FD-PSFERYVLPYQNALKRLGMTVNLRVV-DATQYQNRLR---AFDYDAVTFL-W
PQ----SLSP-GNEQWNFWGAEAAKRPGSMNIAGISDPGVDALIGKVVYATTREE-LVAAARALD-RTLLHH
NYVVPQWTY---------NFQRTARWN-RFGHPQ--TMPEYGAA-AFPTIWWWDATLAAK------------
----
>Rhizobium/1-596 halophilum
------------IIT----LATALLTLPGLADEQPQWRHGIAVIG---EPELPADFKHLPYVDTDAPKGGEL
RLASEG--------TFDNLNLVID-------RGAPAAGLG------------NIYDTLLKRSEDE-------
--VFGSYGLLAEAVTYPDDM----SSATFRLRAEAKWADGAPVTVEDVIFSMEQLKQH---SAFYSGYYRHV

SGVEKTGDREVTFRFDEKN-NKELPSIVG-DFPIFPKHWWEAKDEDGNERDISRTTLK-PPLGSGPYKVASV
QPGGKITYERRDDYWGKDLPINLGQYNFGTISYTYFADSDVAFEAFR-AGTIDYRREN--SSSSWATRY-DF
DAVKEGRVIREALTNPFKATGVMQAFVPNIRREIFKDARVREALNYAYDFESLNKNLAHGGLKRVDSFFWGT
DLAS----SGLPEGRELEILKELE----DQVPP------EVFTKPYTNPVGGEP-DKI----RENLRTAVGL
LKEAGWELR---GNRMVN
VQTG--EPLSFEILLS--S-ASQERTVLPYVASLKRIGIDARLRSV-DTSQYINRVR---SFDYDMIYGV-W
AQ----TMNP-GNEQMEYWGTESVNREGSRNYAGISDPAVDTLIRKIVFAATREE-QVAAVKALD-RVLLAN
HFVVPMFYS---------GEEKIAYWN-RIAHPE--PLPEYGTG--FPDAWF--------------------
----
>Rhodopila/1-581 globiformis
--------------------------------------TYALSQLG---QPALPPDFKYFPYVNPDAPKGGEV
VLGAIG--------TFDSFNPFIV-------RGTPASD-ILR-----------VWDTLMKPNADE-------
--AESEYGLLAQTVEIPADR----MGVAFELRPEAKFNDGTPVTAEDVAWTFETLRDKG--RPFYRQYYADV
ASVSVEGPRRVVFHFK-SNTNRELPLILG-QMVVLPKHWWQ-------GRDFDKPLTD-PPLGSGPYRVGHY
EFGRTLTLERVPNVWSKDLPVMRGQDNFAKRRTEYFRDSTVALEAFK-AGQIDFREEN--IAKEWANSY-DF
PAVQRGAVIKREFRH--HLPTGMQGFGMNTRRPLFQDVRVRHALALAFDFEWANANLFYGAYTRTTSYFSNS
DLAS----SGIPQGDELALLNKYR----EALPP------ELFTQPFTLPVTDGS-GNN----RKELRAALKL
LEQAGWKVR---DRKLVN
ANG---QPFSFEILLAQ---PAFERVALPYVQWLSKIGIDAHVRTV-DPAQYERMLD---TYDYDMIVVA-F
GE----SESP-GNEQSGYWTCPAVKPEGGDNLMGVCSPVIDDLVHQVLTAPDRAH-LLTATHALD-RVLLFN
WYVVPHWYL---------QSVRAAYWN-RFGFVDKP--VRTGIAF---DSWWLDPVKAAANDAARRSG----
----
>Blastochloris/1-581 sulfoviridis
--MSLLG---EPKEKPDFAHFGYVNPAAPKGGMV
RLAAMG--------TFDNLNPVVANV-----RGNIAMGLD------------LIFDTLMTPSSDE-------
--VGTAYGLLAEAVSFPPDF----SSVSYRLRPQARWHDGKPVTVEDVIWSFEVWRKN---SPTHQRYYRHV
KTVEKTGPHEVTFTFDEPG-NRELPQIVG-EFQVLPRHWWEGTDPSGKPRDVTATTLE-VPLGGGAYRIKSA
EPGRTIVYERVADYWGDKLPVNVGINNFDGIRFEYFRDTTVALEAFK-ADQLDWRTES--LAKDWATAY-DF
PAVTEKRVVLEEFPI--RNIGIMQAFAFNLRRDKFSDWRVRRAFDLAFDFEDLNQALFYGQYRRIGSYFDGT
-ELAA---SGVPAGQELEMLESVR----DKVPP------ALFTEPYPTPTGGSP-EAT----RANLREALKL
LNEAGWEVKD--RRLVN
VKTG--EPFMVEFLI--AQ-PTFERVVLRYKPALERLGIVVSVRTV-DETQYQNRLR---QFDFDFVVNS-W
GQ----SLSP-GNEQRYFWGSEAADVPGTGNVGGIRNPAVDALIERVIFAKTREE-LVAATRALD-RVLLWN
HYVVPQWTY---------GKQRTARWD-RFGRPE--RMPAYGAA-AFPTIWWWDAERADR------------
----
>Aureimonas/1-584 psammosilenae
--GLSLIG---EPKYAAGFERFDYVNAQAPKGGSI
RLSETG--------SFDTLNPILS-------KGDPGAGIG------------LPFDTLMKASEDE-------
--VSTEYGLLADGVSYPEDY----SSVTYRLHPQARWHDGKPVTPEDVVWSFTQLKEL---NPSQGFYYRHV
ASAAQTGEREVTFTFDEKN-NRELPNIVG-QLIVLPKHWWTGTDANGRQRSISETTLE-PPLGSGPYKVASV
NAGRGIVFERVEDYWGADLPVNVGQNNVDRIEYVYFRDDSVAFEAFK-ANQFDYWFEL--RASRWATAY-DF
PAVRDGFVKKERYENPYRTAGLMIGFVPNLRRPLFQDLRVRQAMNYALDFETMQRTLFYGEYKRPNSFFFNS
DLAS----SGVPEGQELAFLEPLK----GKIPD------SVFTAPYENPVGGDN-TKI----RDNLRRAVQL
LKEAGWEQK---GGRLVN
AQTD--APFAFEIILN--G-DTLGGMVGQYQQSLKRIGIEMTIRPV-DPSQYINRIR---AREYDMIYTG-L
AQ----SLSP-GNEQYDYWSSGAADQAASQNYAGIKNTAVDQLITNVVQAKDRDT-LVAATKALD-RVLLAN
QYMIPSYVG---------VDARIALWD-RFSHPE--KLPEYSIG--WPALWWYDEAKAAKIPA---------
----
>Pannonibacter/1-586 phragmitetus
---------------------------------------HATALTG---EPRYGADATHFNYVNPDAPKGGTV
KLSAIG--------GFDNFNVLA--P-----RGNLAPGLG------------LIYESLMESSLDEV------
-DISSMYGVIAEAMRYPDDY----SWVEYRLNPKARWQDGTPITAQDVIWSFEKAIEL---SPQQKFYYKDV
SKVEELPGGVIRFSFASAG-NRELPHTVG-QILVLPQHWWEGTDAKGNKRDISRTTLE-PPLASGPYRMKSF
VANREVVYERVQDYWGEDLPIRVGTNNFDEVRYISFLDRTVAFEGFK-GGQYDFHLEN--SASAWLKNY-DF
PAVRDGRVVKEEFVD--HGSGRMQAYVPNLRLKKFQDPRVRQALNYAYDFETSNTIISGGLNKRINSYFAGT
DELEA---KGLPEGKELEILETVR----GEVPP------EVFTTPYVNPVGGSP-EKA----RANLREAVKL
LREAGYSLKD--GKMVD
AQ-G--TPLSFEMLY--FD-QGAERTLLPYQQNLRDIGITMTLRVV-DMPQYINRVN---SRDFEMTTLV-W

AQ----SLSP-GNEQRNMWGSESADRENSQNYAGIRNPAVDKLIDRVIFAKDREE-LVAATRALD-RVLLWN
QYVIPQFYA---------DVDRTARWN-RFGHPD--NMPEFTY--GFPTIWWYDASLAAKTGGA--------
----
>Laribacter/1-569 hongkongensis
---------------------------------------AVALGY---APRYAAGFRQFDYVNPEAPKGGEL
RLPALG--------GFDTLNPFTL-------KGDKEAGVTE-----------LLFDTLAEKS---------L
DEPFSMYGLLADDIRLAPDG----LSVTFHLNPKARFSNGDPVLARDVVSSFVTLTQDKAAHPRFRFYWDGI
RQAVAVDERTVRFDFK--EKNAELHMIIA-ELPVFSHKWLAGA-------ALGSKVLE-PPVGSGPYRLAGF
DLGKQSQYQRRDDYWAKDLPVRRGQYNFDRIRFRYLRDDAVRLEAFK-AGEFDVVAEN--VAKLWARGY-KG
EKFDSGRIVKAEFGH--ENPAGMQGFAMNLRRAPFQDRRVREAFVLAFDFPWANHKQFYDQYRRSPSYFSNS
ELAAS----GSPGADELALLAPWR----RALPEA------VFG-PVVEPPANAS-ADE---LRRNLKRAAAL
LDAAGYPMRG--SVRV
GPDG--QPLSVEFLTYA---KTYERIVAPYARNLARLGITLKTRTV-DPAVYQQRMD---GFDYDMTVAV-Y
PM----SLSP-GNEMLEYFGSRAAGQSGSNNVVGLANPAVDALLPNFLKFQSREQ-LVTASRALD-RILRAE
YLLVPNWHLA---------MHRVAYWN-RFGHPAHLPRYY-QPQDWVIKTWW--------------------
----
>Acuticoccus/1-587 sediminis
--------------------------------------HAGALTG---DPRYPADFKHFDYVNPEAPKGGRV
RLADPG--------GYDTFNPIL--Q-----RGNPAPGIS------------LIYDPLMTPSMDEQ------
-NISASYGLIADRMRYPDDF----AWVEYHIDTNAKWHDGTPITAEDVAWTLTAEKEA---DPNRAFYYKDV
VKAEVVDDGVVRFTFARPG-NRELPYIVG-QLQPLPKAWWTGTAADGSQRSITQGTLE-PPLGSGPYKIARF
EPGRFIEYDRVDDYWAKDLPSQVGTNNFDTIRYDVYRDQTILVEAFK-GGRFDWRTEN--SAKNWATAY-ES
DALKRGDIIKEEFPI--RSQGVMQAFVMNLRLPKFQDERIRRALNLMYDFESQKRTIFYDQYERISSYFMGT
-ELAS---TGLPEGKELEILEEVK----DLVPA------SVFTEPYTNPVGGTP-EKV----RANALEAVKL
FREAGYEIRD---GRMVN
VETG--QPFVIDFVD--NS-PASERYVLPYANSLKRIGINLNFRVV-DSTQYVEKVR---TRDFEMSTLA-W
GQ----SSSP-GNEQAYFWGSEAADQPQSQNYAGIKDPGIDALIEKVILAKDREE-LVAATHALD-RVLLAH
NYVVPLFYS---------PNQRTARWN-IFGRPD--DIPPYGS--GFPTIWWYDSEKAATVEGRS-------
----
>Kiloniella/1-597 laminariae
---------------ILVSFVLSAFLPLSALAETTQKSHGIAIHG---EPGYPADFTHFNYVNPAAPKGGEL
RLASEG--------TFDSFNPYII-------KGVAGPG--F------------QPEALLAASADE-------
--AASEYGLIAESLEWPENR----AWVTFTLRPEARWHDGQPITVEDVIFSLETISTKA--DPYVQNAFAII
KSVEKVGDRQVRFYSA-EIGNREFPITAG-SLPILAKHYWE-------SRDFAKSTLE-PPLQSGPYRIKDF
EAGRYVIQERVKDYWGKDLPVNRGQDNFDSIRTTYYQDDDVIKLAIK-SGEIDYRNER--SSSAWAQDY-DV
PAIAKGWLKKESIPH--KRPQGIQGFFLNTRREIFKDPKVREALGYVYDFEWSNKALSFGLLTRSTNFFGNS
VLAS----TGLPEGRELEILEQYK----DQLPE------RLFTDPFTIPTTDGM-GWP----RDNFRKAFIL
LEEAGWIVR---DLKLVN
AQTG--EQMTFEMLLAS---KGMERTVLPFVNNLRQLGIDVRTRLV-DQSQYYNRIS---DFDFDIVIIK-L
SA----SISP-GTEQRGFWTSETADRKGSGNFSGIHDPAIDALVERVVSAKDRED-LVASVRALD-RALLWG
FYVIPQFHT---------EDDRVLYWD-KFSRPEIT--PWRGTSTA---YWWFDQEKAEALAT---------
----
>Bradyrhizobium/1-592 lablabi
--------------------------------------HALSLFG---DIKYPADFKRFDYVNPDAPKGGTV
RNAAFG--------TFDNFNVVVSGV-----KGALAGGVQ------------LIYETLTTQSLDE-------
--ASTEYGELAEAVSHPDDF----SWVIYRLRPEARWHDGKPVSADDVIFSLEAFKKH---HPQYSAYYRHV
VKTEKFSDREVKFTFDGPG-NRELPLIVG-QLTVLPKHWWEGTDGEGRKRDISATTLE-VPLGSGPYRLKEF
VAGRSITFERVKDYWGRNLAVNVGRANFDELRYDYFRDAIVALEAFK-GDQVDFRVEN--SAKNWATAY-DF
PAVTDKRVLKEEFPN--SRSGVMQAFVPNTRRSKFADPRVRLALNFAFDFEEMNKQIFFGQYTRVSSYFHGI
DELMA---TGLPTGRELEILESVR----AEVPK------EVFTTAYSNPVGGSP-EAV----RSNLQQALRL
MKEAGFEVRD---RKLVN
ATSG--EPFSIELFSRADD-PAFERVALFYKPSLERLGVSVSVRSV-DAIQYQNRLR---NWDYDMLTIQSW
GQ----SLSP-GNEQREHWSSKSADQPGSDNYIGIKNPAIDKLIERVIFATDRQD-LIAATKALD-RVLLWN
HYVVPQWTY---------SKQRTARWD-RFSRPA--EMPKYGLS-SFPDIWWYDAEKAARIGKRS-------
----
>Methyloferula/1-574 stellata
--------------------------------------HGVSVFG---DLALPADFPYFPYVNPEAPKGGTV
SRELFG--------TFDSLNDFIL-------RGDPAAGMDMT------------FDSLMKSS---------L

DEHDALYGLVARAVRISPDK----LTYRFLLRKEARFHDGSPLTAKDVVFSLETLKAKG-HP-RIRQYLRDL
VSAVAEADDTVVVTLAKGRSRDLPLLVAG--YPIFSAAYYAKH-------PFEETTLD-PPLGSGPYKVGRL
EQGHFISFERVADYWAKDLPVNRGQSNFDLIRFDYYSDRSVAFEAFK-AGAFTVHEEF--TSANWAKGYD-F
PAMRDGRVIRETIPD--ENISGIQGWFFNIRRPAFKDPRIREAIGTAFDFKWTNANLMYGSYERTTSYFENS
-DMEAK---GLPDAEELALLEPF----RDKVPAE------VFGQPYVPPESTEP---GQ--PRDLLRKANEL
LLSAGCK-R---QDTL
LLLPDGKPFKFEFLYDE---RGLEPHTQSFIRNLRQLGIDANIRVV-DAAQYKQRMDD---FDFDVTTE---
--RLVMSFSPGEE-LRSRFGSELASVHGSSNVVGIQDPVVDALIAKALVANSRDE-LVHICRALD-RVLRAG
RYWVPHWYKP---------THWIAHWD-VFSRP--DKTPRYDP--GIVSTWWYDESKAKRI-----------
----
>Propylenella/1-585 binzhouense
---------------------------------------AVSLIG---TPKYGDDFRHYDYVNPDAPKGGTL
NQVSVG--------TFDSFNPFIV-------RGTVAAGL-------TPTGG-LLYDTLMDQATDQ-------
--AGTSYGLIAEAVRYPADF----SSATIRLNPDARWHDGQPITADDVVWTFGVLKGT---SPFWNQYYNHI
EKAEKTAADEVTFTFDQAR-NRELPNIIG-DMPVLPRHWWEGTGANGRKRDITQPTLE-PPLGSGPYKIGAF
EGGRSITWQRVADYWGADEPTRVGRYNFDQVKYVYFRNVDAVWEAFKKGGLYDFRMEN--RAQRWAQGY-DF
PAFQRGDVKKHTYLQ--EAGEPMQAFVFNTRRAKFQDPRVRRALTLAFDFESMNRTLFYGLYKRTDSYFEGT
ELAS----SGLPQGKELEILETVR----DEVPP------ELFTEPFTLPVYEKP-GDE----RTYLRQASQL
LREAGWTPQG---GRLVN
EATG--EAMSIEFLGN--D-PTDERVMLPYVNALRRLGIDARIRLV-DTAQYQALTD---NFDFDVV-VDIF
AQ----SQSP-GNEQREFWGSQAADQPGSRNAAGIKNPAVDKLIDRVIYARDREE-LVAATHALD-RVLLWN
YYMVPQWHN---------PEVWIAYWD-KFGIPE--KQPAYIGVD--TFSWWVDQNRESRV-----------
----
>Xanthobacter/1-581 aminoxidans
---MG---EPAYPAGFPHFKYVNPAAPKGGLL
RLAEDG--------TFDTFNFVVP-------RGTPAAGIN------------LIYDTLMVAASDE-------
--VASEYGLIAEGVSYPADF----SSVTYRIRADARWHDGKPITPEDVVFSFEALTKN---NPNQRFYYSHV
TKAEKTGERDVTFTFDQAG-NRELPQIVG-QLTILPRHWWTGTDANGKPRDIAQGTLE-VPLGSGAYRIKSF
IAGRSITYERVKDYWAKDLNVNVGKDNFDEMRYEYFRDDTVMLEAFK-ADQYDFRVES--SAKNWATAY-DF
PARADGRVVLEMFEN--RSSGVMQAFIPNLRRDKFKDPRVRRALNYALDFEGMNHTLFFGQYKRTSSFFSGT
ELAS----SGLPQGRELEILNSLK----DKVPP------QVFTTAYTNPESGSE-EAR----RANLREAARL
FKEAGYEVK---NRKLVN
AK-G--EPFTIEFLLS--S-PAMERVAVFYRPALERLGIEVSIRMV-DTSQYINRVR---SRDYDIIVSG-W
GQ----SLSP-GNEQREFWGSEAADREGSRNLAGIKDPAVDALIEKVIYATDRAD-LVAATHALD-RVLLWN
EYVIPAWGL---------NYTRTARWD-RFAHPA--QLPTYSFA--FPDIWWFDPAKASKTGAGAGK-----
----
>Neorhizobium/1-599 alkalisoli
------------VISGTFLIAIAVMTAPAAAQE-PQWRHAIAVLD---EPKLPADFKRLPYVNPDAPKAGEL
RLSEEG--------TFDNFNPLID-------RGTPAVGLG------------NLFDTLLKHSEDE-------
--VFGSYGLLAEALSYPEDM----SSVTFRLRPEAKWADGQPVTPEDVIFSFEKAKEH---SALYSNYYRHV
TVAEKTGEREVTFRFDEKN-NRELPSIVG-DFPVLPKHWWEGKTAQGQQRDISRTTLE-PPMGSGPYKMASF
QPGSTIRYELRDDYWGKDLPINVGQYNFKVISYTYFADRDVEFEAFR-AGNIDYQQEN--SSSRWATRY-DF
DAVKDGRVIREALTNPFRATGIMQAYVPNIRREIFKDARVREALNYAYDFEDLNKNLAYGGLKRVDSFFWGT
ELAS----SGLPQGRELEILKDLK----DKVPP------QVFTTPYTNPVGGDP-QKV----RDNLRKAVAL
FKEAGWELK---GNRMVN
IKTG--QPMGFEILLN--S-PSQERTVLPYVASLKKIGVDARIRTV-DASQYVNRVR---SFDYDMMYGV-W
AQ----TMNP-GNEQSDYWGSESVSRIGSRNYAGIADPAVDQLIRMITAAPNREE-QVAAVKALD-RVLLAN
HFVIPMFYS---------GEAKIAYWN-RIARPA--ELPAYGIG--FPGIWW--------------------
----
>Halomonas/1-582 salipaludis
------------------------ATEADE----VPTVHGLALYDE---PALPADFSHFPHVNPEAPKGGSM
TRAAVGS-------SFDSTNPFII-------RGTAAIGIS------------QIYDTLLEANPNE-------
--PFSLYGLLAEGIRLDPER----HWIEFDIRREARFHDGEPVTAHDVAFSFELLIEQG--NPFYSGYYAEV
QSVEALDDHTVRFEFASNDSR-ELPLIIG-QLPVLPKHYWE-------SRDFSAPTLE-RHPGSGPYRIGEV
QPGRSITYVRDDDYWGRDLPVNRGRYNIDRLVYDYYRDRDIAWEAFK-AGVMDYRTDA--RAATWAIGY-DF
PAYRDGLVKRLAIPD--VNPSVMQAFVFNLRRDKFSDPRVREALSLTFDFPWLNANIFYDTYKRTESFFQNS
EMAA----EGLPSDAELELLEPFR----DQLP------ERLFEEP--VPIEHPD--DL----RERLRVALDL
LREAGYEVED---GVLRH

```
TETG--RPLSIEVLLYD---SGLERVVQPMLRNMARLGVQSSIRIV-DINQYLNRLR---SFDFDMTTAH-F
PQ----SNNP-GNEQRDFWTSAVAEVPQSRNLMGLQHPVVDALVEQLIRADSRET-LNTLTRALD-RVLLWQ
FAMIPHYHSG---------ETRIAVWD-KFDWPEPYPK--YGMD---LDAWWVDPEREAEI-----------
----------------
>Paroceanicella/1-583 profunda
-------------------------------SDAVTISHGLSVFG---DLKYPEGFPHFDYVNPDAPKGGLH
S---TWA-----FGTFDSLNPFIL-------KGNSGQGLGLL------------FDSLMTGS---------A
DEPDSQYGLVASKVEYPADR----SWVIFDMRPEATFSDGSPLTADDVVFTFETLRDKG-TPSYAI-QFQDF
ESVEALDPHKVKFTFRAGASTRDLPLTAG-QMPILSRAYYDTH-------DFTESTMT-PPLGSGPYAVERA
DPGRSITFERRDDYWAKDLPVNVGQNNFDEIRFEYYGDYTAAFEAFK-GGGYNFREEY--LSKLWAESYH-F
PAIDKGWVKTEVIPD--ATPSGTQGFWFNMRREKFRDPRVREALAMVFNFEWSNKALFYDQYKRTASFWENS
-ELKAE---GMPTQAELKLLEPLR----ADLPEA------VFTEPAYVPPVSST-ERI---DRRVLRQAGKL
LDAAGWTVR-----------------------------------------------------------N-GL
RTNAKGEVLSLVFLNDS---PSMERIITPYVDNLRQLGVDASLRSI-DAAQEEQRRKS---FDYDITSQ---
--RYVMSMSPGDE-LRQMFGSQSAETPGSANLTGLSNPAVDTLIADIVNARDRDE-LNTAVHALD-RVLRAM
HLWVPNWYKG---------SHTIAYLD-VFGRP--DPLPAYSM--GELSLWWWDEARAAKL-----------
----------------
>Sabulicella/1-580 rubraurantiaca
-------------------------------------THALSLLG---EPSLPRDFPHWPWVNPDAPKGGEV
TLTGLG--------SFDSFNPFIL-------RGTPAIG-LSN-----------LYDSLLVESADE-------
--SSTEYCHLAEWVELPADR----RGVSFELREGARWHDGRPVTAEDVAWTFEALRTHG--RPFYRSYWADV
TEVVAESPRRVTFRFR-SDENRELALILG-QLQVLPKHWWE-------GRDFARPLLD-VPLGSGPYRLERF
EAGRSLTLRRVEDYWARDLNTRRGTNNFDAIRFEYFRDATVAFEAFK-AGQIDFRTEN--VARNWSTGY-DF
PAVRRDLVKRDTIRH--ELPTGMQAFVMNLRRPLFQDARVREALVHLFDFEWLNANIFFGLYARTNSYFSNS
ELAS----SGLPQGREKEILEGFR----GRIPE------SVLTTEFRLPTTDGS-GNN----RDGLRRALEL
MRQAGWTVR---------------------------------------------------------DRRLVN
AQG---QRFEFEILLNG---ATFERVALPFVQWLGRLGIEARVRTV-DPAQYQVRID---GFDYDMTMEV-L
GQ----SLSP-GNEQRDYFTCARAREPGSQNVIGICDPAIDELVEMVINAPDRQE-LIHRTRALD-RILLHH
HYVIPNWHL---------RAFWVAFWD-KFGRPERS--PRYGLGFP--DSWWVDPARERALAEARR------
----------------
>Alsobacter/1-581 metallidurans
---------------------------------------ATSLMG---EPRYKPGFAHFDYVNPQAPKGGTV
RLGRQG--------AFDNFNPVVAGV-----KGQLEAGVG------------LVYEQLTEQSLDE-------
--VSTAYGLLADGVKFPEDF----SWVTYRLRENARWQDGKPVTPEDVIFSFETYKET---SPQMAFYYSHV
VKAEKTGEREITFRFDQPG-NRELPQIVG-QLTVLPKHWWEGTAPDGRRRNVKETTLE-PPLGSGPYRLKAF
DAGRNAVYERVADYWGKDLPERIGTNNFDQVRFEYYRDSTVLFEALK-GDQYDFRLEN--SAKNWATGY-DF
PAVKDGRVVKEEFTE--RASGRMQAFAFNLRKPKFGDQRVRRAFNLAFDFEEMNKTIFYGLYSRIGSFFEGT
-ELAS---KGLPEGKEREILESVR----DKVPA------SVFTAAYANPVNGDA-TKV----RDNLREADRL
LKEAGWDIKG---------------------------------------------------------GKRVN
AK-G--EQLTAEFLA--YD-PTSERFVLFYKPSLERLGIGVSLRVV-DPSQYENRMR---DFDFDITTDL-W
AQ----SLSP-GNEQRDFWGSKAADRPGSRNTLGIRDPGVDALIDKVIFARDREE-LVAATKALD-RVLLAH
DYVVPQWFS---------PATRTARWN-RFGRPG--VMPRYASP-AFPTVWWWDAALAQK------------
----------------
>Sphingobium/1-588 lignivorans
----------------------------------VTTAHGYAVFGE--L-KYPADFKHLDYVNPDAPKGGIY
RYAQTG--------SFDTLNFFGL-------LGTPPFA-LLW-----------IYDTLMQRSLDE-------
--PASYYPLIAETISYPRDL----AWVEFRLDPRARWHDGKPITPEDVIFTVAKFKELV-SPTYRR-IGAAV
SRVEKTGPRSVRLYFVQK--GNPTMPTVVAAMPVVPRHVWQ-------GKDFTASTLE-RPVGSGPFRIGRM
SPGRWLEMERVKDYWAKDLPINKGKWNFDIIRHDFYRDVGVMNEVFL-SGQADLRFEG--SAARWDAQDQ-M
PAFKAKNLVRDVIRY--ENGAFYMGLMMNSRRPFLADRRVRKAITLAYDYEWVKRVLLAGHHGRLASFFANT
EFAAE----GLPGEDELALLAPFR----DQLPP------ELFT-QPPELPVAGQWGSR----RENLVQAAAL
LREAGYRIED---------------------------------------------------------GLLID
PRTR--QPVRLGLAAYSAL-MD--RQVSLFIENMRQLGITVDFRSY-DTAQFRHKIR---NFDFDLMINL--
-PSFPPLVTPG-LELMQFWSSRAADTPQSFNYMGVRSPVVDALVMKVG-TATDRATVVSAMRALD-RVLLWD
YYAIPFQHTYPAP----MGQVPITYWN-RFGRPAKDPTYNFP--FLTMDHWWIDKEKEARL-----------
----------------
>Halovulum/1-582 marinum
------------------------------RGQEAITSHGISAFG---ELKYGPEFPYFDYVNPDAPKGGRM
```

TFRGTGA-----SATFDSLNPFIL-------KGEPAQGLGLL------------YDSLLVGS---------A
DEPDSAYGLIAERLEYPEDR----SWVIFHMNPEATFSDGEPIEASDVVFTYNVLLEKG-HPAYKI-SYKDF
ETVEALDTYKVKFTFRQDAAKRELIQQAG-GISILPEHYYQDV-------DFAESTLT-PPVGSGGFLVEEA
KPGRSITYCRNPDYWAWDHPVNVGANNFDCYTYEYFADYTAAFEAFK-GGSYLLHEEF--FSKLWATGYD-F
PAIDEGWVVKESVPD--NRPSGTQGFWINMRRDKFQDPRVREAIGLMFNFEWSNQTLFYGIYQRTDSFWENS
-PMQAE---GLPEGAELELLQEFH----DELPES------IFSEPVYTPPVNQP-SRT---DRAAVRKASRL
LDEAGWTLQ---G-GV
RKNAQGQTLTLEILDDS---ASFERVFNPYVENLKRIGIDARLSLI-DAAQYQQRQED---FDYDMIPG---
--RLVMSLTPGLE-LRQIFGSDSAKEPGTANFSGVSDPVIDALLEKAVAAGSRED-LEVAVRALD-RVLRSM
HIWVPNWYKG---------EHNLAYWD-VFSKP--AEKPLYSR--GDA-YWWWDEDK---------------
----
>Plasticicumulans/1-560 lactativorans
---PRYPADFQHFDYVDPAAPKGGTL
TLGAIG--------GFDRLNPFVL-------KGTAAAGVSE-----------LACDTLGEQS---------R
DEPFSIYGLLARDIELEPDR----LAVTFTLDPRARFSDGSAVTAEDVKATFDALKGP-KAHPQYRVYWADV
TAAEVLDARHVRFRFA--RVNPELHLIIA-QMPVFAARWVEGR-------NFDDIVLE-APLCSGPYVVDKF
TLGRSITYRRNPEYWARDLPVRKGQYNFDRVTFEYYQDAGVALEGFK-AGEFDLAQVN--VAKQWVRDY-VG
PKFDSGELLKRELAH--HNNAGMQGFVFNLRRPLFRDVRVRRALALAFDFEWSNQNLFFGLYRRSSSYFANS
ELAAT----DVPSGAELALLEPLRSQ----LPPA------LFT-QPWRAPSTEA-PGS---LRDNLRAAQAL
LAEAGWTLQ--DEVLV
KD-G--QRLEFEALLEN---RAFERVFAAYAKNLQRLGVRMNYRTV-DGSLYQRRVD---IFDFDLVVHV-Y
AQ----SQSP-GNEQIGYWHSSMAAQEGSNNLMGLRDPVVDALVEKLVYAQDRAQ-LVAAAHALD-RVLLWG
EYVVPNWHSP---------VHRIAYRD-RFGMPKQLPLYY-QPLEWALSTWW--------------------
----
>Inquilinus/1-580 limosus
-----------------------------AEAASAGKTYALTELKL--PPKYPADFKHWDYVRPDAPVGGSL
RLAAFG--------TFDSLNSLIT-------RG-TPGAGLNL-----------IYDALIAANADE-------
--LTAYYAYVAHSIEHAEDN----SWMVFKMRPEAHFHDGTPMTAHDVVFTHETLRDKG-APRLRIRFYADV
DRIEALDDHTVRFSAKSL--QNPNLLMAIATFPILPKHWWQN-------RTFDDPVLD-PPLGSSTYKIKSV
DPGRSIVYERVPDFWGRSLPQNVGLGNFDEIGYDYYRDNNVMYEALK-AGAFDFIEVT--QSQEWATGFTNV
PAVKDGRLILEALPS--DEPANFAGFWFNTRRPQFRDVRVREALAQFYDFETARRTIHYNLFKRVDSYFPNT
DFAAT----GVPEGRELEILNQFK----GKIPD------KIFT-EPFRLPTTDGSGNI----RANLARARDL
FSQAGWTVQN---GTLVE
TATG--QPMAFEIMFTSQP-TE--KIVNAFIANLARGGIKA-TARLVDVPQYINRMD---NYDFDMSV----
-TALNIFYPPG-QELRGAWKSEAADEKGNENMTGVKDPVIDALVEIAVAASTWDEKVAAC-RALD-RYLLWN
WLTIPTFYDD---------THRLAYWK-MFGRPATRPKY----GVGFPDSWWFD------------------
----
>Alkanindiges/1-583 hydrocarboniclasticus
--------------------------------AAPITQNYIALHS---KPLYAS-ATAMPYANPKAPKGGML
SQSAVLA-------TFDNFNTLNG-------KGTPAEGIG------------LVYDTLMSPSLDE-------
--PGIRYPLLAERVTYDPDDS---SYVIYHINPKARFSNGTPVTAADVVFSFNMLLTKG--APGIKVYLAEV
DKVIALNKLAVRFNFKSKD-NIELPSIVG-EVPIYSQKDWQ-------GHDYERVSLR-IPVGSGPYLMDNF
VAGRSITYKRNPNYWAKDLPVNVGRYNFDRMRYLYFRNPDVAFEGFK-AGQYFFQEEN--KARTWSIGY-NF
PAFNQKYVVKKAISN--QNPVNLQAFVFNTRRPLFSDIRVRQALTYAYDYEWLNKALFYGHYDRIQSYFYNS
ELAA----TGTPSAAELKLLKPWL----DKLSP---IQRQGVLTNWQFPKSDAS-GFN----RNNLLKAREL
LLQAGYHYQH---GKLMD
KSGK---PFRFEILIRQ---DGLQRTILPFTRNLARLGIDATIRLV-DAPQYMERQR---TFDFDMITLA-T
SN----TISP-GNEQAQYWSSVSADQEGNYNLAGIKNPAIDAMIDLIIRAPNREQ-LITRTRVMD-RLLRAG
YYLIPTYSKS---------KYWVAYWD-MYGYTAQR--PKYDLG---VDYWWVDPAKEKRV-----------
----
>Antarcticimicrobium/1-611 sediminis
-------------ILGLALIFLVGFATLLSAQEVVTKSYGFSEFG---ELKYPEGFAHFDYVNPDAPRGGEL
SYAAQG--------TFDNFNPFTR-------QGRAGARSGEQ------------YESLLFPS---------Y
DEPAAYYGLLAESLEYPESQ----DWVIFNLRPEARFSDGTPVTAEDVVFSHNILLEQG-LQSYAEAVRRRI
PKAEALGPHRVKFYFAPDISRRALITQVG-GTPVFSKAWFEADP---DNRRIDKPRME-PGIGSGPYVLDSY
DINRRVVYKRNPDYWGDHVNVNVGRNNYETIRIEYFSDSIAALEAFK-AGEFTLRQEN--NSKSWASAYD-F
PAIDKGWVLREELED--GNVPDATGFVMNMDRPQFQDIRVREAVQLAFNFEWTNESLQYGLFRQRQSFWQNS
-PLEAK---GLPEGRERAVLEALG----DDLDPA------LLESEPVMAHSSRA-DRQT--DRGNLRKAMKL

LDAAGWAVG---DDGV
RRNAQGQTLRIEFLSDD---PVMDRIVMPFVDNLQVMGIDASYNRI-DNAQFTLRRRER---DFDMISA---
--GYRTSLQPSTG-LYQQYGSEAAAY-SVFNPAGIHGPDIEALIDNIVSAREWED-LSANTRALD-RVLRAR
RFMVPTWYLG---------KYWVAYWD-KYAYP--EILPPYAL--GIEDLWWVDADKAAALTSSG-------
----
>Meinhardsimonia/1-606 xiamenensis
------------------VMALLALSSGAARAQEIIKAHGISTFG---TLKYPADFEHLDYVNPEAPKGGEM
SIWAFG--------GFDSMHPYTT-------KGRAGALSTIF------------FESLLEGT---------A
DEIGASYGLLAESLEYPEDR----SWVIFNIRPEARFSDGTPLTAEDVVFSYELFRDKG-LPSFRAVLRTQV
ESAEVLGPHRVKFTFTPGIPTRDLPETVG-GLPIFSKAHYLAN-----NRDFEESSLE-PLLGSGPYVLDRM
EVGQTIVYRKDPDWWGADLPINRGRYNFETIRIEYYADYNAAFEGFK-GGSYTFRNEA--SSKIWATGYD-F
PAVQKGWVVKRELPD--GTIAPGQAWAINMRRPHLQDVRVREALGLMFNFEWSNEKLFYGLYERIHSFWENT
-ELAAS---GVPAPEEVAILKPLVD--EGLLPPS------ILTEEAVMAPVSSA-SRQL--DRGNLRKASAL
LDEAGWTVG---SDGM
RRNAAGELLTVEFLNDS---QTFDRVINPYVENLRRLGVNAVHQRV-DNAEATNRER---NYDFDLVTT---
--QFRMSYIPGAG-LKQYFGSETADS-SVFNLMGLKDPAVDRLIDLVIAAETQEE-LLHRVHALD-RVLRSI
RFWVPQWFKD---------VHTVAYYD-MYEHP--DPLPPYAL--GNLDFWWYNAEKAEKLKAAG-------
----
>Granulosicoccus/1-586 antarcticus
-----------------------------VSEQGVTTSWSMAEFGE--P-LYDENLTHWPYVNPDAPTGGTV
VLGDFG--------SFDSLNSYIL-------KGEWPRS-ISL-----------ASDTLMVGSGDE-------
--LAAAYGLLASTVEYPADK----SWIIFNLRPEARFDDGNPITAADIEFSFKTIREHG-RPFLKS-FYSEV
ESVEVLGDHQIKFSFNTT--GSMKPLMKVAGLSPLSVEYWKD-------KDISKTYLT-PGPSSSGYFIADV
DAGHSITYKRVENYWGKDLPVNKGLNTFDSLRYDYYRDLEVMLEAFK-AGEIDFRAEN--SSKRWATAYQ-T
EEVEKGEIVLDTPPD--NAPGAIQAFFFNLRRAPFDDHRVRQAINLLYDFETIKRTILYNQYERINSYFPNS
EYGAA----GAPSPDEVAVLEPFR----EQLDP------AVLT-QEFLSPVTDGSGRN----RREMRQALAL
FNEAGWKLSG--GKLMK
N--G--QPLKLELLLVQAD-GQ--RVAAPWIQNMQKAGIQT-SIRLVDSAQYQVRVD---DFDFDMIS----
-ARLNFFPPPG-PELRSYYGSAAADERGSANMAGIKNPVVDELIEQIIAADSLEKLQLTT-RALD-RVLLWN
HYVIPQFFNA---------EHRIAYWN-RFGKPDVLPKYISFGGSGFPTGWWLDTELDSKL-----------
----
>Vineibacter/1-576 terrae
-------------------------------------SHGIAIHG---EPGYPPDAKHLDYANPDALKGGSI
RLAAGG--------TFDSLNPFIL-------QGTAARG-VAG-----------IYEPLLETVYDE-------
--VSTEYGQLVETIETPPDR----AWVIFTLRANARWHDGKPVTSDDVVFSFNILKEKG--QPLYAVYWHDV
VAAEALDQRRVKFSFR-GGDNHELPVIIG-QLTVLPKHWWA-------SRAFDQASLE-IPLGSGPYRVESL
EAGRFIVMRRVEDYWGKDLWLSRGRYNFDTIRYDYYRDEQVAFEAFK-AGDVDYREEF--TSRLWATSY-DF
PAVKSGAVQKVELKH--ESTLPMQGIGFNLRRAMFTDRRFREAFVHLVDFEWFNKALSWNLFTRIDSYFFNS
ELAA----KGLPSKDELALLEPLR----SQLPP------ELFTKEFKLPVTDGS-GNN----REGMRRAIAL
LKEVGWEVR---SGKMTN
VKNG--QQLSFELLLGA---PRLERFALPFKEWCGRVGIDVRLRTV-DPVQYQKRMD---EFDYDATIVI-F
SQ----SLSP-GNEQREYWGSAAAKTKGSQNLIGIADPAIDKLIEAVIAAPDRPS-LVTRSRALD-RALLWN
YFIVPQYYS---------TTFWIAYWN-KFGRPEKT--AKYQPRGI--ETWWLDPEKEKTL-----------
----
>Aquaspirillum/1-570 serpens
---------------------------------------AVALGY---TPKYPPHFSHFDYVNPTAPQGGTL
TLPALG--------GFDTLNPFTL-------KGDKEAGIAM-----------LLFDSLAEKS---------W
DEPFTVYGLLAEDIALAEDG----LSVTFRLNAAARFSDGSAVEAADVKHSFDTLTQDPAAHPRFRAYWSDI
KQAVVLDKRHIRFDFK--RRNAELHMIIA-ELPVFSRRWGGQG-------QLGKQPLT-PPLASGPYLLTKY
QLGKNSEFKKNPHYWAKNHPVRRGMFNFDRIRFRYLLDDTVRLEAFK-AGEFDVVAEN--IAKTWARSY-RG
AAFDEKRILKHTFPN--QNPTGMQGFVMNQRRAVFADRRVRQALALAFDFDWANRQLFYGQYRRSQSYFSNS
ELAAQ----GTPSAAELALLNPLR----EHLSAA------VFG-PAVTLPASPD-AFA---LRNNLLQARQL
LFEAGWLLSP--EGKLV
DAQG--RPFRFEFLTFS---KTYERIVAPYQRNLARLGIELAIRTV-DSSIYQQKLN---QFDFDMTVAV-Y
GM----SLSP-GNELRDYFSTASADQLGSLNLAGVKHPALEHLLDHFVSFRDRNE-LVTAARALD-RVLRAE
YLLVPNWHLA---------THRVAWWD-KFAYPQQLPLYY-QAQEWMIHTWW--------------------
----
>Phaeovulum/1-591 veldkampii

```
-----------------------------VQAQNTIVSHGISTFG---DLKYAADFPHLDYVNPEAPKGGEI
SVWTAG--------GFDSFNPYTL-------KGRAAALASVV------------HESLLTST---------A
DEIGAAYGLLAESLEYPESR----DWVIFTLRPEATFSDGSPVTAEDVLFSYETMRDKG-LISFRAVLTQQV
AGAEVLDARRIKFTFTPDYPRRDVIQSVG-GLPVFSKADYIAK-----GRDLENSSDT-PFLGSGPYVFDTA
DMGRRVVWRRNPDYWGADLPINRGRSNFDRIRVEYFGDYEAAFEGFK-AGAYTFRTEA--SSLIWATRYN-F
PALDKGWVVKRTLPN--GQIGTGQAFSINLRRDRFQDPRVREALGLMFNFEWANETLFYGLYTRTNSFWDNS
-ELAAT---GTPGPDELALLEPLAD--Q--LPPG------VLTDPAVVAPTS-G-PRQL--DRGNLRKAAAL
LDAAGWPVA-----------------------------------------------------------DDGM
RRNPQGQTLRVEILNDS---QTFDRVINPYVENLRALGVDAVHTRV-DDAQFTNRER---SHDFDMITD---
--QLGQDYIPGSG-LQQYFGSGSVG--DVFNSMGLAHPAVDALIAQVEAAHTRDE-MTTAVRALD-RVLRAL
RFWVPQWHKA---------EHTVAYYD-MYEHP--EALPPYAL--GELDFWWYNAEKAEKLKAAG-------
----------------
>Geovibrio/1-591 thiophilus
--------------------------FFSQAEAGITKNHGFSLTGE---LKYGADFRHFDYVNPNAPKGGTL
RRAMYG--------TFDSFNPFAP-------KGLAIKATG------------YLYDSLLTSSSDE-------
--AVSYYGLIAETMEYPDDY----SWVVFNLRPEARWSDGTPLTAEDVVFSFEKIT-EA--SPFYSNYYNLI
TKAEAVGRHSVKFHFKKGETSRELPLIAG-QLSIIPKHFWQ-------TRDLSKSSLD-IPPVSGAYRIASY
EIGKRVTFERIKDYWGEKLPVNAGQNNFDAIVFEYFRDQTVAFEAFK-AGHFDFTAES--SGRRWYRGY-TG
KYFDMGLIRKEEIPH--KNPQGMKGIVFNTAVKPLDSVLVRKALNYAYDYDWINKNIYFDQDKRHDSYFSNS
ELAC----GSVPPAAVAALIKQVK----PDAG------DGLMKTPFKLPSTDGS-GNN----RENLKTAVQL
FEQAGYRIVN---------------------------------------------------------GKMTG
KD-G--RSLYLEISTSS---KTIEKELMTFKKALERIGIDFYIRYL-DSTQFVDKVR---SKDYMMIYTT-V
RQ----SESP-GNEQRNMWHSEAADEAGSRNYARIKDPAVDRLVNMIINAPDRKS-LVTYSKALD-RVLLNG
WYFIPAGYSD---------RYRIAYWD-KFGKPAKMP---EY-SFG-FGSWWIEPGKEKKIDSLIKR-----
----------------
>Chenggangzhangella/1-588 methanolivorans
--------------------------------------HGLSLLG---DVKYPADFKHFDYVNPDAPKGGTV
RLGALG--------TFDSLNAFVP-------KGNEAAGLN------------LLYDTLMTDALDE-------
--PATEYGLIAESVRYPADY----SSVTYRLRPEARFNDGSPVTAEDVVWSFETLKRI---DPMRSRYYHDV
AKAEITGEREVTFTFAGPG-NRELPQITG-QIPILPKKFWEGSDEQGRKRNIEEGTLD-KPLGSGPYRVKAF
APGRWISYERSPDYWAAKLPARVGIYNFDEMRFDYYRDSTVLVEAFK-GDQFDYREEN--SAKNWATAY-AF
PAVQQKKVLLEKFVD--SQSGRMQGYAFNIRRDKFKDPRVRRAFNLAFDFEEINKSIMFGQYVRIDSYFYGT
DLAS----KGLPEGREKEILESVR----DKIPA------EVFTSAYKNPVGGSP-EKA----RENLREAVRL
LKEAGWTVSR------------------------------------------------------EGGKAALK
NEKG--EAFKVEFIY--GD-PTGERLLSFYKPALERLGVEVTMRLL-DDSQYINRIR---SFDFDMVTVG-W
GQ----SLSP-GNEQRDMWGSSSADVSGSQNFVGIKDPGVDALIDKIIFAKDRDD-LTAAARALD-RVLLAH
NYVVPQFTS---------IEDRTARWD-RFSRPA--KLPPRGA--MFPTVWWWDAEKAKAVGGRS-------
----------------
>Methylocystis/1-586 rosea
---------------------------FGPLAQGEVESHGLSTFG---DLGEPADFKYFGYVNPKAPKGGTL
ALSPASS-------TYDSFNAYVL-------RGNPATGMTLV------------FDSLMNQS---------L
DERDAYYGLVAKKVRISPDK----LTYAFLLRKEARFHDGSPLTAHDAAFSLNILKSKG-HP-VISQMLRDL
ESAEAEADDVLVLRFAPDRTRDLPLSVVG--QPIFSRAYYKDR-------DFAATTME-PPLGSGPYKVGAF
EQGRYIAYHRMPDYWAKDLPITTGQANFDVVRFEYFGDSQVAFEAFK-AGAFTEREEN--IARVWATGYD-F
PAFKEGRVKRTTTPN--YNIPQIQGWFFNTRRKIFKDPRVREAIGYAFDYEWTSRNLMYDAYKRISSYFENS
-ELEAK---GLPSEQEKALLEPF----RADLPAE------VFGEPFVAPVSDGT---GQ--DRVLLKKANDL
FVAAGCT-R-----------------------------------------------------------KDGV
LCLPDGTPLEFEFLDNS---NVFERHTQPFIKNLKLLGVSAHIRVV-DAAQYKQRLEN---FDFDVVHD---
--VLMMSWNPGEE-LRAYFSSKTANVPGSRNLPGVSNPAIDSLVEKALQAQTREE-LVTCSRALD-RALRAQ
RYWVPHWYNP---------VHRFAYWD-LFGQP--ERPPKFDT--GVLWTWWWDEDKAKKI-----------
----------------
>Sulfuritalea/1-581 hydrogenivorans
--------------MLKRIAHVVAPLLCALVAGASFASHGLALGG---RPKYPAGFSHFDYVNPAAPRGGQL
TLAAMG--------SFDKLNPFTL-------KGVPPVNLTE-----------LVFETLVAQS---------D
DEPFSVYGLLAEDMALAQDE----MSITFRLNPKAKFSNGDPVTAEDVKHSWNMLISK-AASPLYRAMWADV
KGIVVVDPRTVRFEFK--RRNRELHMIVG-QLPVFSRKWGNG-------KPFDQIVTD-TPIASGPYLVDKA
ALGKTISYRRNPAWWGGDIPARRGMFNFEGVAYRYYKDELIRIEAFK-AGEFDFVHEN--MAKNWARSY-QG
AKFDRGELIRRELTH--LNPQGMQGYVFNLRRPLFQDVRVRKALTLALDFEWMNRQLFYNQYKRNYSYFTNS
```

EMAAT----GSPDAAELKLLEPVR----RHLEPE------AFG-PVPRPPTTVA-PRS---LRENLREAREL
FRAAGWEFR--DGALR
NARG--EVFEFEIPLSG---KSWERVVAPYARNLEKLGVSVKYRII-DSAILAKRTD---DFDFDMLLHW-F
LS----SQSP-GNEQFLRFASETADEKGSQNLIGLKNPGIDHLVDAILTADTREK-LVTACRALD-RALLAG
YYMIPQWHNT---------VHRVSYKK-HLGIPEKQPLY---------------------------------
----
>Oricola/1-584 cellulosilytica
--------------------------------------HATSLIG---EPKYAEGFERFDYVNPDAPKGGSL
RLSETG--------SFDTLNPILS-------KGEVASGLGA-----------FVYETLMKSSLDE-------
--VSAEYGLLAEALSYPDDF----SEVSYRLRADARWHDGEPVTAEDVVWSFEKLVEL---NPQRRFYYQHV
TGAEAVDERTVRFTFDEKD-NKELPHIVG-QVPVLPKHWWEGKDASGEQRDISGSTLE-PPLGSGPYRVSAM
NPGSTLTFTRVADYWGAELPVNAGQHNFETITYNYYRDRNVEFEAFK-ADEFDFWNEN--EAKRWATAY-DF
PAARDGSIVREELENPYRAQGVLVGFVPNLRKPMFQDARVRRALNLVFDFETLNKTIFYDQYERVDSFFYGT
PLRW----EGFPEGRELEILETVR----DQVPS------AVFTKEYTNPVSGGG-RQQ----RQNLREALRL
LLEAGYEQS---DGVMVH
KETG--QPLSFELLLN--G-PTIERVALPFAEDLRKIGVEMRVRSV-EPSQYVARAR---SRDFDMIYTG-W
AQ----SLSP-GNEQFDYFGSKAADSEASQNFAGIKDPAVDALIRRVVFAKDRDE-LIAATKALD-RVLMAN
QYVIPSYTS---------REARIAYWD-RFAHPE--PLPEYSIG--FPEIWWYEAEKAAAI-----------
----
>Arboricoccus/1-567 pini
--------------------------------------HGYALYD---DLKYGPDFSGFGYVNLKAPKGGRL
IDSALG--------SFDSFNPYI-------LRGRAADNVD------------LIFDTLTVQSQDE-------
--PFSRYGSVAQYIFMPPDR----SWVAFEVNAAARWHDGTPILASDIAFTFEILKTKG--SPAYQFYYRQV
AGVDILGERRVRFNFVPDGSNRELPLVVG-QLPVLSAAYWST-------RDFEANSLD-KPLGSGPYRIRDV
DPGRSVTYERIDDYWGADLPINRGRYNFAEIRSEYFRDLDVSLEAFK-AGQYDFRFEN--SAKRWVTGY-NG
PAVEEGKIAKLDLMI--PSSARMQGFAMNIRRAFFADPRVREAIGYCFDFEWSNKTLFFGQYERIRSYFHGH
LEFMA---TGLPGEAEREMLTPFK----DVLDP------RLFSEEFELPHTDGT-GNV----RDNLRHAFGL
LKDAGWSVEN---GSLKH
IQTG--QSMAFEILLS--Q-SDLERVALPFVQNLSRLGIKANIRTV-DPSQYQNRMN---QFDFDMTVAL-W
PQ----SSSP-GNEQAQFWSSKAADVEGSKNFVGIKNRAIDAICERIVQATRRDE-LEIAVRALD-RVLLWG
FYVVPHYTL---------GHYWLARWK-KIAFPA--TLPTQSPD--VFS-WW--------------------
----
>Enhydrobacter/1-588 aerosaccus
-------------------------------SSKIHVGHGIAMHG---EPKYGADPGPLDFLNPAAPKTGAV
KFGTLG--------TYDSLHPFTL-------KGVPAAG-LGS-----------LWETLCWHSPEE-------
--AFTAYGLIAETIEWPEDR----SWVAFTLRAQAKWHDGTAITPEDVVFSFDILKSKG--RPTYAAYYADV
IKAEKTGDAKVLFTFR-NDKNRELPLILG-QLPILPSTWWK-------GKDFEKVSLE-PALGSGPYKVDSV
DVGRSITYRRVPTWWARDLWFNRGRNNFETIRYDYYRDNTIIFEAFK-GGDTDIRREN--SGRNWMIGYKDL
PAIADGRIQRAEIAH--ENPAPMQGFVFNTRRDIFKHRNVREAIGLMYDFEWQNKNLSYGFYQRTRSYFGNC
ELEA----KGLPSPEELKILEPLR----GKIPD------EVFTAEYNPPKTDGS-GNI----REQVRKAIPL
LKEAGWEIK---DGKMTD
-KNG--RKLAFEILLND---AAFEKMALPVKQNLERLGIDMTIRTV-DTSQYQRRTD---NYDFDMVIDL-W
AE----SLSP-GNEQRDFWGSKAADIPGGRNTIGIKDPAIDQLVELIIAAPDRES-LVTRTRALD-RVLSWH
MFAIPQFYS---------GKALVAYWN-RFGRPSKT--AKYEPLAF--DTWWVDEAKDRALTRGEKK-----
----
>Bartonella/1-600 choladocola
-----------FCLFFALNSIA---------IADPEWRTSLSLGV---EPKYGDNFTHYDYVNVNAPKGGTF
NDSRTG--------TFNSFNPYIV-------TGTPAAGF-------SSRGGGEQYDTLMAPSTEE-------
--TSVNYPLIAEAAQYPDDF----SWVKFRLNKKARWHDGRPITVEDVVWSFNVLKSN---SPFFNNYYHSV
KKAEKTGENEVTFVFQEKG-NRELPYVMG-QMPVLPQHWWEGTDETGKKRDITAPTLE-IPLGSGPYEVESF
IAGKSITWKRVKNYWAADLPVNRGRFNFDKEHYSYFLDPNAAWEAFKKGNLVDWNLEN--RIQRWNQSY-DF
PAVKNGQVIRRSFAF--HGSGRMQGFFINTRRPQFADRRVRQALNLAFDFESLNKSLFFNDYHRISSYFDGL
DLAA----KGLPQGKELEILETVK----DEVPA------EVFTTPFSNPVYDKP-DSS----RRYLTEAMRL
LNEAGWHLEQ---NRLVN
KK-G--EVFTLEVMLS--D-MAFERATAPFIANLQRLGIDASMRVV-DASQYQNREA---NFDFDLV-IKVI
GQ----SDSP-GNEQLEYWGSKAADRQGSYNVAGIKNPAVDKLIERVIYAKDREE-LVAATRALD-RVLLWN
YYVIPQWYA---------EHLNIAYWD-KFGIPE--PQPEASGVD--IPSWWVKKDK---------------
----

>Oleiphilus/1-588 messinensis
-----------------------------VEQSGLTTSHGFALYG---ELKYPPDFKHFDYTNPNAPKGGEI
TLMGFG--------TFDTLNPYTL-------KGISPWGSPGFF----MYGIGELNESLLAGSSAYLKS---G
DEPFSAYGLIAKHLTYPADF----SWVEFELNSKAQFHDGHPIDADDVIYSYHILIS--EGHPIYRQSLEDI
ADVQKVNSQRVRFNFK--ARNKPSAILRAGEMPILPEHFWSGR-------EFKDSLTT-PPLLSGPYRISEA
KLGNRVTFQRVPNHWGKDHPVYKGRFNFDTVHFDFYRDQTIAFEAFK-SDEFDVFYDY--TAKNWATAY-TF
PALKQGKVVKAEIPH--DIPSPTQAIVFNTRRAPFDDIRVREAFSLMFDFEWTNTNLFHDAYRRTRSFYPNS
PFSAN----KVISPKVKALLISLG----LDEQSP------ILNQPLHHHETNGN--GN---IRPQQRQALAL
LKDAGWTFLD---GKLLN
AEM---QPLSFELLIRQ---KGLERVMLPYLKNLERLGIEGSLRLV-DSSQYKVRLD---QFDFDLTTHI-W
SQ----SPAP-GHEQKNYFHSTLKSVQGSQNYAGIANPIIDRLTERITQVKDYDE-LIVTMKALD-RMLLSE
HYVIPNWHID---------YHRVAYWN-KFGLPQQQAEFV-----LGFENWWY-------------------
----
>Thalassococcus/1-606 halodurans
----------------IASVFAAERLWAQEAQDGVIKSHGISSFG---ELKYPADFKHWDYVNPDAPKGGEY
S---TWA-----FGTFDSLTPYIL-------KGNAAAGATVF------------YDTLMTGN---------L
DEPDSMYGLVAESVEYPENR----EWAIFNLRPEAKFRDGSQVTAHDVVFSFEKLFNEG-QPSYRV-ALKDF
EKVEALDDFRVKYTFNPEGPLRELLMTAA-GLPIFSKAYYETR-------DFTESSLE-PPMGSGQYELLSV
DPGQSVAYKRRDDYWGKDLPVNVGQNNFDVIKFEYFADYTTAFEAFK-AGSYSYREES--LSKLWATSYD-F
PAIDKGWVKVETLPD--GRPSGTQGFWFNLRRDKFSDPRVREAIGLAFNFEWSNESLFYGLYDRTDSFWENS
GTLQAE---GMPSEAELALLEPLR----ADLPET------VFTEPAFVPDVQKA-EDLA--DRRALRKAGKL
LDDAGWEVG---ADGI
REKD-GEKLTIQVLNDS---ASFDRIINPMIENLRRLGIEADATRV-DAAESQEREKN---FDFDIVTQ---
--RFSMSSTPGDE-LRTIFGSETANTPGSANMAGIANPAVDALIEKIATAESREE-LTVATRALD-RVLRSM
HVWIPQWYKG---------AHNIAYLD-MFARPYTDTPPPFGM--GTSSIWWYDEEKAQALKEAG-------
----
>Gulbenkiania/1-575 indica
-----------------------------------RSAPAIALGY---VPRYQAGFSHFDYVNPDAPKGGQL
VLPATG--------SFDTLNPFTL-------KGDKEAGVSL-----------LTLDTLLAQS---------E
DEPFSLYGLLAEDVVLAPDG----LSVIFRLNPKARFVNGDPVTAADVVASFRTLTTDRTATPLYRVYWADV
AEAAALNSRTVRFTFK--RRNAELHLILG-QLPVFSRKWIPPGR------TLADVATT-PPIGSGPYVLESW
AAGRYARYRRNPAYWAVTLPVRRGMYNFDHITFRYYQDTTARLEAFK-AGEFDLIEEN--TAKDWARGY-NG
PRFRDGRILRRTLPH--ENSAGMQGFVFNLRRPLFADRRVRQALSLAFDFEWANRQLFYNQYRRSDSYFTNS
EMAAR----GLPTQEEMKLLAPLR----ARLDPA------VFG-EAVLPPDSAG-PYG---LRNNLKRARSL
LFEAGWRYEG--GRLV
DRNG--RPFVFEFLSFS---RSYERIVSVWQRNLARLGITLNVRVV-DPAIYQRRMD---RFEYDSTVVV-Y
GA----SQSP-GNEQLDFHSCEAARTPGSRNWAGLCDPAVESLLQHFLHFESRTV-LVAASRALD-RVLRAG
HYVVPNWYLP---------YHRVAWWN-RFGQPARTPRYYPIATYWALETWW--------------------
----
>Segnochrobactrum/1-598 spirostomi
-------------------------------AADVKWRHGTALVG---EPKYPADFKHFDYVNPTAPKGGLV
RLSAEG--------TFDTFNFVVP-------RGNLADGLG------------LLYDTLTTSSLDE-------
--PYSEYGLLADSLSYPPDF----SSVKYHLRPEAKWQDGTPVTPEDVVWSFEVLTKN---NPQQQFYYRHV
KSAAATGPNEVTFTFDQAG-NRELPQIVG-QLLVMPKHWWEGTDAKGRKRDITAGSLE-PPLASGPYKIKTM
VPGRSITYERDPNYWGRDLPVNVGTNNFDQIRYDYYRDDVAELEAFK-ADQFDWRVEA--SAKTWATAY-DF
PAVKDGKVVLEMFPT--RGSGVMVGFIPNLRRAEFQDPRVRRALNLALNFESMNRILFFGQYQRINSYFYGT
DLAA----AGLPTGRELEILNEVK----ASVPPGTIP-DSVFTTPYSNPTAPDV-DAE----RDNLKKALDL
LQAAGWELK---GRQLVN
VKTG--QPFTAEFLIN--G-PTFERVGLQFKQALSRIGINLTLRTV-DSSQYVNRVR---SHDFDLIYGG-W
PQ----SLSP-GNEQRGFFGSDSADEDGSQNYAGIKNAAVDKLIDKVIYAKDRDD-LVAATHALD-RVLLAN
DYVVPGWTL---------PASRVARWN-RFAHPD--PLPQYSTG--FPTIWWYDQALAAKIGRAPQ------
----
>Paracoccus/1-585 zeaxanthinifaciens
-------------------------------------SHGIGTFG---EPSLPADFDHLPYVNPDAPKGGEI
AESIPNS-----T-GFDNYNPFTF-------RGRAAVLSTVM------------LESILTGT---------A
DEVGAAYCLLCESIEYPESR----DWVIFHLRPEARFSDGTPLTANDVLFSYETLRTKG-LSSFRVVIAQQV
AGATVIDDHTIRFDFTPDYPRRDVIQSVG-GLPIFSAKDFEEN-----DRDLEQPMRT-PMIGSGPYVFGNA
DINRNVTWERDPDYWGADLPINRGRHNFDRIRFEYFADYDAAFEAFK-AGEYTFRREV--SSIIWATRYD-F

PSLQKGWVVKEELPD--GNIASGQAWVLNLRRPDWQDIRVREAIGLMFNFEWSNASLFYGLYDRVESFWDNS
-ELEAE---GPPTEAELALLEPLAA--D--LPEG------VLTEDAVSPPVS-G-ERQA--DRGNLRKAAAL
LDEAGWTVG---SDGM
RRNADGRVLSLEILNDS---QTFDRVINPFVQNLRRLGIDARNTRV-DNAEYENRKR---GHDFDMISD---
--HLGQDAIPGAN-LQQYFGSASVD--DVFNSMGLANPAVDSLIATIEAADTQED-LTTAAHALD-RTLRAL
RFWVPQWYNG---------QHLIAHYD-IFGRP--DTLPPFDL--GELDFWWYDQAKADALRDAG-------
----
>Frigidibacter/1-583 mobilis
-------------------------------------AHGVSTFG---DLKYPADFPHLDYVNPEAPKGGEI
SEWTMG--------GFDSFNPYTI-------KGRAAALSSAV------------HESLLTST---------A
DEIGSAYCLLCETLEYPEDR----SWVIFTLRPEAKFSDGTPVTAEDVRFSHDILLTQG-LSSFRAVISQKI
ETVEVLDDRRIRFTFKPDYPKRDLLQAAG-GLPVLSKADFEAK-----GRNLAESSMD-PFVGSGPYVFDSM
DVGRRVVWKRNPDYWGKDLPINIGRHNFDRIRIEYFGDYQAAFEGFK-AGAYTFRNEA--SSLIWATGYN-F
PALEKGWVVKRELED--GSIANGQAFVLNLRREKFADPRVREAIGLMFNFEWSNASLFYGLYERVESFWENS
-ELEAT---GTPSPEELALLEPLAA--D--LPPG------VLTDEAVRAPTS-G-PAQL--DRANLRKAAAL
LDEAGWPAG---ADGL
RRNAAGEVLRVEMLNDS---QTFDRVMNPYVENLRRLGIDAVLTRV-DDAQMTNRER---NHDFDMITK---
--HLGQGYVPGAG-LQQYFGSNSID--DSFNAMGLQSPAVDKLIATVEAADTRED-MTVAVRALD-RVLRAM
RFWVPQWYKA---------AHTVAYFD-MYEHP--DPLPPYAL--GEMDFWWYNAEKAETLKAAG-------
----
>Siculibacillus/1-576 lacustris
--------------------------------------HGTSLMG---QPKYPAGFAHFDYVDPKAPKGGVL
RLSETG--------TFDSFNPLIP-------KGNPAAGLS------------LVNQSLMTSALDE-------
--VATEYGEIAEAVAFPDDF----SSVTYRLNPKAKWHDGTPITADDVVWSFKTAVEL---SPTQAFYYKHV
KDAAVSGEREVTFTFDQTG-NRELPQIVG-QLTVLPRHWWEGTGPDGRKRSITETTLE-PPLGSGPYRIKSW
EPGRSVTYERVRDWWAADLPTAIGTNNFDEIRWEFFRDDTVELEAFK-GDGYDVRMEA--TAKNWATAY-DF
PARAEGRVVLEQIPT--RAQGVMVGFIPNLRRAKFQDPRMRLALSYTLDFEEMNRTLFFGQYERIVSYFHPT
ELAS----SGLPQGAELDLLHEAE----KAGPIP----AEVFTKPFANPVTADG-SAA----RANLKEALRL
FGEAGWVSK---GGKLVS
AATG--EPLVLEFLTD--G-PAFERVAVRWRESLAKIGVTLTVRIV-DSSQYVNRLR---SHDYDLIYSG-W
AQ----SMSP-GNEQQYYFGSASADSEGARNWAGIRNPAVDALIQKVIYAKDRAG-LIAATRALD-RVLLWN
HYVIPGWTA---------PYTRMARWN-RFSHPE--PLPLLSSG--FPTTWWFD------------------
----
>Arenibaculum/1-582 pallidiluteum
--------------------------------EKVTKAWAIAEWGE--P-LYGPDMPHFPYVRPDAPKGGTV
TLAAFG--------GFDSFNTMIP-------RGEWQRTNIGL-----------ISDSLMEGSADE-------
--IGVVYGAVAESVEYPEDY----SWAIFNLRPEARWHDGAPITAEDVKFAFDILKQHG-RPFLRA-IYDPV
ASAEILSPHRIRFAFSTK--GKRKPLILAAGMSPWPKHWWSQN-----GRDPSQTFLE-PPLGSGPYRLVTV
EPGRRLVYERVPDHWGADLPTNRGRNNFDRIIYDFYRDQTVMFEAFK-GNAYDFREET--SPLRWATGYD-F
PALAEGRVVKREIPV--QTPGGMSAFFINTRRPHLADPRVREAINHLFDFEWIQKNIFFGYYKRNTSYFIQS
EYGSS----GLPTGRELELLEPFR----DRLPA------RVFT-QPFEPPKTDGSGNI----RANQRRALQL
FKEAGWELKD---GRLVN
AQ-G--QPFTLEILTILPD-DE--RWTNAYVENLRKSGIEARHVRLPDTATWERRAD---QFDFDLFT----
-ATYTFFPPPG-TELVSRFGSAEADVQGSANMTGIKDPVVDAMLERIVTARDHETLVAAT-RALD-RVLLWG
WYVVPRWHKT---------ESWVAYKD-IFGRPDRAPKY----GVGFLDTWWVDPRKSA-------------
----
>Undibacter/1-601 mobilis
--------------------IGALSFGGVAVAQDKPWKHGLSLFG---DVKYPAGFKNFDYVNPAAPQGGIV
RLGAFG--------TFDNFNEVVAMV-----KGNLAGGIG------------LLSESLMTQAFDE-------
--VSTEYGLLADGVRYADDY----SWVTYRINAKARWHDGKPVTVDDVIFSFTTLKEN---RPDLAAYYNHV
VKAEKTGEREITFTFDAPG-NRELPHIVG-QLTILPKHWWEGTDKSGNKRDVTATTLE-PPLGSGPYRVKEF
VPGRTIVFEKVADYWGKDLNVSIGTNNFVQIRYEYFRDLTVSLEGFK-GDQIDWRTEN--SAKDWATAY-DF
PAVRDKKVVREEFPM--TSVGVMQAFAFNIRRDKFKDARLRRAFNFVFDFDEMNRQLFYGQYKRIDSYFYGT
-ELAC---SGVPQGLELDILNDVR----AEVPA------ELFTQPYANPPGGGP-EVL----RNNLRSALML
MREAGYEIKD---TKLVH
TKTG--ERLKVEFLL--QQ-PTFERVVLAYKASLERLGIEVSLRTV-DTSQYENRLR---QWDYDIIVSS-W
GQ----SLSP-GNEQREFWGSKAADRPGSRNTIGIKNPAVDKLIERVIYAKTREE-LVAATHALD-RVLLWN
NYVVPQWSY---------PFQRTARWD-RFGRPE--TLPKYAAS-AFPTIWWWDEAKAAK------------

----
>Tianweitania/1-604 sediminis
-------------------LFFLPFSASLHAQDAEEWHTTSSLIP---GENEDKPFERYSYVNPDAPKGGTL
NAVAPG--------TFDSFNPFVV-------RGTAAAGF-------SSFGG-IMYDTLMQQSLDE-------
--AGTSHGLLADAFRYPDDY----SSATYRLNPDARWHDGKPVTVEDVIWSFNVLKKN---SQLYNRYYANV
TEAVKVGEREVEFRFDQKG-NRELPHIMG-DLAVLPKHWWEGTDASGNKRDITQPTLE-PPLGSGPYRIQSF
RPGSEIIWERVEDYWGADLPVNIGRNNFDRKRYVYIQDDNAAWQAFTKGGFQDIRPEN--SSRRWHVDY-TF
PAFQAGDVVKRAFET--RSGEPFQGFFLNTRRPQFQDPRVREALTYAFDFESMNRTLFYNAYTRTDSYFEGG
ELAS----SGLPEGRELEILNEFR----GQIPE------RVFTEPFTLPTNETP-RDE----RANLRRASEL
LKEAGWTLRD--GRLVN
EA-G--EQFRMEFLGD--D-PTDERIATPYIQNLRRLGIDASLRVV-DASQYINRTR---SFDFDVISSVQ-
QQ----SQSP-GNEQRDFWSSAAADAPDSRNLSGIKDPVIDKLVERIIFAEDRED-LIAATKALD-RVLLWN
FYAVPQWHL---------PEIWVAYWN-KFGIPD--QQPAYIGVD--IDSWWIDEAKEQAL-----------
----
>Pseudogulbenkiania/1-592 subflava
-----------------MRILLLIGVVGALLARLAVGAPALALGY---APKYPPNFTHFDYVNPAAPKGGRL
VLPAQG--------SFDTLNPFTL-------KGDKEAGVGT-----------LVVETLMTQG---------E
EEPFTVYGLLADDIRLADDG----LSVSFHLNPRARFSNGDKVTAEDVKFSFDMLTRDPTASPSYRFYWADV
ARAVVLDPANVRFDFK--RRNAELHLIIA-QLPVFSRKWLPPGT------KLGDRVLQ-PPIGSGPYLLEQY
DEGKFSRFRRNPAYWAANEPVRRGMYNFDTILYRYYKDETARLEAFK-AGEFDLSVEN--VAKQWARGY-LG
SKFEDGRIVKKTLPH--GSSAGMQGFVFNLRRPLFRDKRVREALALAFDFEWTNRQLFYGQYQRSDSYFTNS
ELAAK----GLPGPDELALLNPLR----SKLDPE------VFG-PAVEPPQVTG-RYG---VRTNLRRAREL
FYQAGWRYEG--GRLV
NKQG--RPFEFEFLSYS---RTYERIVASWQKHLAKLGITLTVRVV-DPAIYQRRLN---DFDYDTTVVV-Y
GA----SQSP-GNEQLGYHGSEAARTPGSSNWAGLADPAVDALLQNFLHFKTRRD-LIAASRALD-RVLRAG
RYVVPNWHIP---------YHRAAWWN-RFGQHSRLPRYY-DATSWAIETWW--------------------
----
>Amaricoccus/1-583 solimangrovi
-----------------------------------IIRSHGISTFG---DLKYPPDFAHFDFVNPDAPKGGTM
VFRGTGA-----SKTFDSLNSFIL-------KGEPAQGLGLL------------YDSLLTGA---------P
DEPDASYGLIARGIEYPEDR----SWVIFDMRPEAKFSDGAPITAEDVVFTYDALMEKG-QPSYQI-TLRDI
ASVEALDPHRVKFTFRPGVPSRDLPAMVG-GLAILPKHYYDEV-------DFGESTMT-PPVGSGQYHVENA
QPGRSIRYCRNPDYWGKDLPVNIGSSNFDCYQYEYYGDDNAAFEAFK-VGDYQFHQEF--YSALWATGYN-F
PAMDEGWVKREEIPD--NTPSGTQGFWFNLRREKFQDPRVREAIGLMFNFEWTNATLFYGLYQRTDSFFENS
-PMQAE---GVAGGEELAVLEPFR----DRLPPE------IFTEPPYTPPVSGE-QQI---DRSALRRASKL
LDEAGWPVG---KDGL
RRDARGATLGVEFVDDA---PTFERIINPYVANLRRIGVDARFSRI-DAAQMQQRLKT---FDYDIIPT---
--RFVMSMSPSIE-LREIFGSAAANAQGTANLTGLADPVVDALIEQVIAAKSREE-LDARAQALD-RVLRSK
QIWVPNWYSG---------KFLIAYWD-IYGRP--DQPPPYSR--GDA-FWWFDQAKFDKL-----------
----
>Telmatospirillum/1-600 siberiense
-------------------------------ESRAGQGAHALALYG---TPKYPPDFKHFDYVNPDAPKGGLL
RLYQLG--------GFDTLNPFSGIGH-----PPLARGATAR----PIDLIGLTFDSLLARSEDE-------
--PASAYGLIAESFEWDRAN----RWLVFNLRPEARFHDGSAITADDVLFSFETLRNSG-PPG-LKASLQGV
TRGEKLADRKVRFIFGPGETR-ALPLVLG-SLPILSKSWWQ-------GRDFTRPLAE-PPLGSGPYAVQSV
DPGRSVVLVRAQDYWAKDLPVATGLYNFDRIRTDFFADSPAAFSAFK-NGAYDVRFEW--ESRKWATG-YVF
PALTRGLVNKLNIPN--RRTEPMRGFAFNLRNPLWRDPRTRRALILAFDFESLNHDLFYGLYRRAGSYFAKS
ELAAP---ELPSD-EETLLLEAFR----GQVPD------ELFAKPYSLPDNADH-AQR----QRNLAEAAHL
LSEAGWTMKKG---KLVD
GQGG--KPFVFEILVDD---PSWQPICKDFLSALHHLGIQASVHAV-EEAEFSRRRA---QAEFDMIVARWP
A-----SETP-GTEQRTLWGSAAADQPDGLNWAGIRSPAIDRTIDALAASPDRQT-LVTRTHALD-RLLLWG
DYVIPQWYMP---------EDHVAVWD-KFGIPAVT----PDQG-AEVLAWWVAPEKSKPAAKDPVKAR---
----
>Consotaella/1-574 salsifontis
---KYADSFPHYDYVNPDAPKGGTL
NQVAIG--------SFDSFNPFIV-------RGTAAAGMSAG---NGGFGGGLIYETLTEQSTDE-------
--PGVSYGLIAEAMKFPEDD----SWVKFRINPNARWHDGEPITADDVIWSFDTLREL---HPQWNAYYRNV
VKAEKTGEREVTFTFDQKG-NRELPHIMG-DLVVVPRHWWEGTDASGKKRDITQPTLE-PPLGSALYKIDSF

STGSSIIWARVPDAWSKDLPQNVGRHNFDRMKFVYLRDSNASWEAFKKGGLDDWRVEN--ISRRWAEGY-DF
PAAQNGQVIKKEIHS--PSVESMQAYVLNNRLPKFQDRRVRQALTLAFNFEEMNRTLFYNSYKRITSYFANS
ELAA----TGLPSEGELKFLEPLR----GQIPD------EVFTKEFTLPVYDDA-AAT----REHLRQALDL
FKQAGYELKG---TSLVN
AKTG--EPFTIEFLGD--D-PTDSRTLEPFARQLQRLGIKTNIRII-DTNQYVERMT---NFDFEVMAAKYY
PQ----SLSP-GNEQRDFWSSKAADSPGSRNYAGIKSPVIDKLVDTIVYADNRDD-LVAATRALD-RVMLWE
YYVVPQWYK---------DTIWLAYWN-KFGMPE--TAPEYAGID--PYSWW--------------------
----
>Profundibacter/1-595 amoris
----------------VALALAVLWATSVRAEQKIIKSHGISTFG---ELKYPADFPYFDYVNPDAPKGGEF
S---TWG-----FGTFDSLTPYIL-------KGNAATLSTAF------------FDTLMTGN---------L
DEPDAMYGLVAETIEYPEDR----SWAIFHMRPEAKFSDGTPVRAEDVVFSFEVLRDKG-SPTYKV-LFKDF
ENVEALDDLTVKFTFAADAIKRELPMTAA-GIPIFSKAYYATR-------DFAESTLE-PPLGSGAYELLSV
DAGRSVAYKRRDDYWARDLPVNVGQNNFDVIKVEYFADYTAAFEAFK-GGAYSYREEF--LSKLWATGYD-F
PAIQKGWVIKEELPD--GNPAGTQGFWFNLRRPQLQDPLVRQAISMAFNFEWSNKSLFYGIYTRTDSFWENS
-DMQAE---GMPSEAELALLEPLR----ADIPES------VFTEPAFVPAVSNP-DKVS--DRKLLRRAGKL
LDQAGWTVG---YDGF
RYNDKGQKLTIEILNDG---PSFERIINPFVENLKRLGVNAVYTRV-DAAQAQDREKN---FDFDVTTR---
--RYSMSETPGIE-LRGIFGTSTADLKGSNNISGVKNPAVDSLIKTIEGAKSREK-LTTAVKALD-RVLRAM
HIWVPQWYKP---------SHTIAYYD-MFERPYTDTPPKTSL--GELSIWWYN------------------
----
>Albimonas/1-584 donghaensis
-------------------------------ADGIIRSHGISTFG---ELKYPADFPHFDFVNPDAPKGGVW
SGRGTGA-----SNTYDSLNPFIL-------KGEPAQGLGAL------------FDTLITGS---------P
DEADASYGLVAERMEYPEDR----SWITFHMRPEARFSDGSPVTAADVVFTFEVLKTKG-APSYRL-RLRDV
ASAEALDELTVKFTFAEGVSTRDLPDLVG-GLPILSAAWWETR-------DFEASSLD-PILGSGPYRVDVA
DPGRRIIYARRDDYWAKDLAPNVGANNFDRYVFEYFKDYTAAFEAFK-AGAYLFHEEF--FSKLWATEYG-F
PAIQRGWVKKEQLPD--GRPSGTQGFWFNMRRPQFQDPRVREAMAMAFDFQWSNDTLFYGLYQRTESFFENS
-PMAAS---GTPSEAELALLEPLR----ADLPET------VFGE-AVRPPITDA-SGS---DRRVLRRGMKL
LDAAGWNVV---D-GM
RRNAAGETLRVEFLDDS---PTFQRIIGPYIENLKKMGVDASLRIV-DAAQYQQRQED---FDYDVIPG---
--RFVMSLTPGPE-LRQLFGSESADQRGTPNLTGLANPAVDALIARVIGAESREE-LDIAARALD-RALRAL
HIWTPNWYKG---------SYTIAYWD-VYGRP--EQQPPYSR--GDG-YWWWDEAKAEKL-----------
----
>Beijerinckia/1-583 mobilis
------------------------------QAGEAYESHGLSIFG---DLALPADFPHLPYAYPAAPKGGIF
IEQAGFN-------TFNTFNAFIL-------KGDPASGLGLL------------FDTLMTSS---------S
DEPDALYGLVAEKVAVSADR----TLYRFFLRKEARFHDGTPIKASDVAFSINLIKTKG-HP-ILRQGLRDL
ESVEAEADDIVRVRLRAGHSREAPLTVAS--QPIFSQAYYKTH-------NFEETTLE-PPLGSGAYKIGPF
EQGRFIAYARVENYWGKDLPINRGRHNFDTIRYEYFNDRKVAFEAFK-AGVFTYREEF--TSILWATGYD-F
AALKEGKVKRETIPD--LYPRGTQGWFFNTRRGKFKDPRIRRALGYAFDFEWTNANLMYNLYKRTVSYFQNS
-PMEAR---GLPSAAEIAYLDPF----RKDLPEA------VFGEAVLPPVSDGS---GQ--DRTLLREASEQ
LRQAGCT-R---KGNK
LLLPDGKPFEIEFLGFE---TSFQPHTAAYVKNLKLLGIDADYRVV-DAAQYKRRTDE---FDYDIVVE---
--RFGFGLTPGED-MRLIFGSESAAIPGSRNVSGIALPSVDALIEKALVVDTREN-LTEICRAID-RILRAH
YFWVPMWNNP---------NHLLAFWD-LFQRP--QRPPRYDV--GALTTWWFDPEQAKRI-----------
----
>Chelativorans/1-572 intermedius
---FERYDHVNPDAPKGGTL
NASVIG--------TFDSFNPFIV-------RGNPAAGL-------TFFGG-FLWETLMQQSPAE-------
--PGTSHPLIAAAFRYPDDY----SSATYRLNPAARWHDGTPITAEDVVWSLATLKEI---SPQHRHYFANV
EEAVALSEHEVEFRFDQAG-NRELPHIMG-DLPVLPRHWWEGTDARGNRRDITQPTLE-PPLGSGPYRIASF
RAGSEIVWERVKDYWGAALPVNIGRYNFDRRRYTYFLDDNAEFLAFTKGGTEDIRREV--STRRWSQNY-DF
PAVQAGDVIKREFTS--TGIEAMQAFVFNLRKPRFADRRVRRALTLAYNFEEQNRTHFFGLNRRFSSYFEKS
ELAA----TGLPEGRELEILEMYR----DQLPP------ELFSEEFTLPVFDSP-RAE----REHLREAVRL
FGEAGWEIRD---GRMVN
KETG--RQFTIEFLGI--A-PTSEIIVSGIIPNLRKIGIDARLRIV-DTSQYIQRVQ---NFDFDAT-TGRF
AQ----SMSP-GNEQRDYWSSAAADIPGSRNLAGIKDPVVDALVDRIIYAEDREE-LVAATRALD-RVLLWN

HYMVPQYYQ---------PTLRYAYWN-KFGIPE--KQPAYAGVD--IESWWVDPQRQATL-----------
----
>Rhodoplanes/1-591 serenus
----------------------------GAAAVEAPESHGLSAFG---DLKYPADFRHFDYVNPAAPKGGLL
SLIGSTRQFNQNFLTFNSLNSYIL-------RGDAALGMELT------------FTSLMTRA---------A
DEPDALYGLAARAVRVAPDG----LAYTFLLRPEARFHDGTRITAHDVVFSLTTLKTKG-HP-VIGQLMRDV
LAIEADDDTTITVTFAKGRARDVPLFVAA--LPIFSRAYYANR-------PFDETTLD-VPLGSGPYRVGRF
EPGRTIAYERVRDWWGAGLPAMVGQYNFDTIRYEFYRDRDVAFEGFT-GRSYLFREEF--TSRIWATRYD-F
PAVRDGRVKRDVLPD--ETPSGAQGWFLNTRRDKFKDRRLREALIVAFDFEWANASLMYGSYQRTHSVFQNS
-DMMAE---GKPEGAELALLERF----RGRIADE------VFGEPFVPPVSDGS---GQ--DRALLRRAVQL
LNEAGYP-V---KNGK
RVMPNGEPLTIEFLLDE---PAFQPHHMLYIKNLGLIGIEATLRMV-DPVQHRARIDA---FDFDAVIQ---
--RFAFSTTPGDS-LRSYFSSQAAALKGTQNLAGIADPVVDALIEEVIAANSRAD-LVVAARALD-RVIRAG
RYWVPHWYKP---------SHWLAYWD-VFARP--ATKPRYAR--GVLETWWTDPAKAAK------------
----
>Paenihalocynthiibacter/1-591 styelae
------------------------------KSEDVITSHGISTFG---NLALPADYTHLPYVNPEAPKGGEI
SIWGFG--------SFDSMNPYST-------KGRAGGLANIG------------LESLLTST---------A
DEQGASYGLLAESLEYPEDR----SWVIFNMRPEARFADGSTVTAEDVVFTYETFVTKG-LPSYRAVLSQQV
ASAEVLDTHRVKFTFHEGIPTRDLPQTVG-GLPVFSKAHYEAE-----GLDLEESSLT-PLLGSGSYELDEL
DVGQSITYRRRADYWGADLPMNIGRNNFDHIRVEYYADYQAAFEGFK-GGSYTFRNEA--SSRIWATGYD-F
PAMDRAWINKVSLPN--GDMGQAQSFNFNMRQEPFEDQRVREAIGMMFNFEWSNETLFYGLYERVNSFWENS
-DREAT---GLPSEAELALLEPLAE--H--LPDG------VLDQEAALAPAS-G-TRQL--DRRNLRAASRL
LDDAGWTVD---DRGM
RRNAAGELLTIEFLNDS---PSFDRILNPYLENLRALGVNAEYNRV-DNAQATDRER---KHDYDFITY---
--SYPMSWFPSDN-LKQFFGSETAMT-SVFNKSGIHSPAIDALIGHITRAQTEEE-LVTAVRALD-RVLRAE
KFWVPQWYKD---------SHTVAYYD-MYEHP--DPLPPFDL--GYLDFWWFNADRAAELEAAG-------
----
>Hansschlegelia/1-581 zhihuaiae
--------------------------------------HGLSLFG---DVKYPAGFKHFDYVNPDAPKGGTV
RLGAFG--------TFDSFNAFIP-------KGNPAAGTN------------LLYDTLMTEALDE-------
--PATEYGLIAESVRYPADY----SSVTYRLRPEAKWQDGKPVTVEDVIWSFETLKRI---NPLMSRYYHDV
AKAEQTGEGEVTFTFAGPG-NRELPQITG-QIQILPKHWWEGTDPQGRKRNIEEGALE-KPLGSGPYRVKDF
APGRWVSYERVPDYWGAKLPVRIGTYNFDEMRFDYFRDMTVLVEAFK-GDQFDYREEN--SAKNWATAY-QF
PAVAEKRVILEKFKD--SQSGRMQGYAFNLRRDKFKDPRVRRAFNLAFDFEEINKTIMFGQYVRIDSYFFGT
ELAS----KGLPEGREKEILESVK----DKVPP------EVFTTEYRNPVGGSP-EAA----RKNLREAVDL
LKQAGWTVSR--EGGKPALR
NAQG--EAFKVEFIY--QD-PSGERLLSFYKPALERLGVEVTMRLL-DDSQYVNRIR---SFDFDMVTVG-W
GQ----SLSP-GNEQRDQWGSQAADVPGSQNFVGIKDAGVDALIEKIIYARDREE-LTAATRALD-RVLLAH
NYVVPQFTS---------VEDRTARWD-RFSRPA--NLPPRGS--LFPTVWWWDAEKA--------------
----
>Pontivivens/1-586 insulae
--------------------------------QEIITAHGISSFG---DLKYGPDFTHFDYVNPDAPKGGFF
T---TWG-----FGSFDSMRPYIL-------NGNGARSVGIL------------YDTMLTGS---------A
DEPDALYGLLAESIEYPEDR----SWIIFNIRPEARFHDGSPVTAADAEASYWALYNDG-RPVYRLGIFRDV
AGVEVLDERRIKFSFTEGAPVRDMPGTVG-GLPIFPAAWLAEN-------DFAEASME-PVPGSGPYVLSDL
DEGRSITYSRVEDYWGADLPVNRGSNNFDEQRVEYFADSTAAFEGFK-AGAYTFRSEN--SSRQWATGYD-F
PAIEAGHVVTETLID--GTPTGTQGWWFNLRRPQFQDPRVREAIGMMFNFEWSNRALFYDLYNRTDSFWENS
-YLQAE---GMPTEEELAILEPLR----EHFPEE------VFTEEAFSPVVSSA-ERL---DRRTLRRATAL
MREAGYTIV---D-GK
LVDADGEQFRLEILNDS---PAFERIINPFIQNLERLGIEVVAPLI-DNAQAQEREKN---YDFDMTTR---
--RYAMSLTPGTE-LVGIFGSSSANELDTANVMGLANEGVDALIELIAQAENRDE-LNVRVRALD-RVLRSM
HLWVPQFYSG---------QHFIAYRD-VYERP--ETKPPFAL--GTG-TWWWNEERAEELRAAG-------
----
>Thermohalobaculum/1-592 xanthum
--------------------------FGARASEGIIRAHGVSAFG---DLKYPADFAHFDYVNPDAPRGGTF
STGGGPG-------TFDSLNPFIL-------KGNPATLIGLT------------FDSLMDGS---------A
DEPDSMYGLVASSIEYPEDR----TWCAFELRPEARFADGSPITAEDVAFSFNIRREKG-HPSFRL-QYAAV

TGVTVEGPHRVRFDFDRSAALRDMPMAVA-GLPVLSAKWWEGR-------DFAASSLT-PILGSGPYRIETA
DPGRVISFVLRDDYWGWDLPVNRGRWNYQRISFEYYRDRSASFEAFK-AGKFLFNEEF--WSKQWATGYD-F
PALKRGDVVRATLPD--NRPSGTQGYWFNTRRSKFLDPRVRKAIAICFDFEWSNKTLFFDLYTRTDSFFEGG
-PMEAS---GSPTPGETTLLEGLASKLPPDLPED------VFGTAYVPPVTDGS---GR--NRRELREAARL
LDEAGWKIV--DGK
RRNAQGEVLEIEFLDAS---SAFERITVPFIRNLERIGIVATNRVI-DPAQYRKRMED---FDFDITTD---
--RKVMSLTPGVE-LRDYFHSSSANSPGSDNTAGVDNPAVDALIETIERSNDRET-LTNAVRALD-RVLRAM
HIWVPQWSKA---------SHHIAYWD-IYDRP--EVKPAYSL--GNFDLWWVDPDKFAYL-----------
----

>Brucella/1-618 neotomae
----------FWVSVFAVAMLASAMPASRADNAEPQWRYSSSLLD---EPKYPADFKHYDYVNPDAPKGGTL
NLVAVG--------TFDNLNPYVV-------QGVSAAGL-------SDFGGGMLYDTLMADSQDQ-------
--GSTQYPLIASALQYPDDF----SWVKFKLNPDAKWHDGQPITVDDVIWSFNVLKKQ---SPMYNKYYSDV
ESAEKTGEHEVKFTFSRKG-NRELPQIMG-QLAILPKHWWTAKDARGKQRDITRPTLE-IPLGSSAYKIESM
KPGHSIIWARVEDYWGKDLPVNVGRNNFDHVAYEYYFNEDATWEAFKKGGQYDYRNEN--RAQRWAEQY-NF
PAVQRGDVVKASFPF--HAVGRMQGYFLNTRRDKFKDPKVREALTYAFDFESMNRLMFYNQYKRINSYFAGN
ELAL----SGPPTPAEQAILETVK----DALPA------DALTKEFKLPVYDTP-QAT----RENLRTALKL
FSQAGWTLKG---NTLVD
AK-G--NPFTIEFLGQ--D-PTDERIYNPFAASLRKIGINATVRIV-DAAQYQARVN---DFDYDVI-TAVI
AQ----TASP-GNEQRDMWGSKAADFKGSRNYAGIRNPAIDKLIDLVVYAKDHEE-LEAAAHALD-RALLWN
YYVIPQWYS---------DHINVAYWN-KFGMPE--KQPDYLGID--PYSWWIDPAKEAKLKTGG-------
----

>Inquilinus/1-579 limosus
-----------------------------VQAEAPAAGAGIAMHG---QPKYPPGFDHFAYANPDAPKGGEL
RQAVIG--------SFDSLNPFIVGG-------RRDQILS--------IVPPYHFAQLMARSYDE-------
--PFSLYPYVADRVEMPDDR----RSITFHINPKAVFHDGTPITADDVIFTAQALGTKG-LPT-SRALYDRI
ASVDRLDDRTVRFTFKEEASR-EAPLVVA-LQPVLSKAWYT-------AHPFDQPSLD-IPLGAGPYKISQV
DPGRSIVLERVKDWWGADLPAMRGQHNFDVMRYDFYLDDDVALEAFK-AGAYTLRREW--NAEKWVGGAYDF
PAAQDGRVTLLTLPH--YRPAGMMGFAMNSRKPLFADPRVRRAMILAFDFEWVNKTLLGGQYKRDDSFFANS
PLAAT---DT-PKGAELKLLEPFR----DQLPP------QLFTEPYTLPPSDGS-GRN----RENLRAAQAL
LAEAGWTIRDN---VLVD
G-SG--TPFRFEILLQD---KTYQRIALAYADQLRRLGVAASVRLV-DSAQYRNRTE---TYDFDMLLNKWL
V-----TLSP-GSEQMVYWGSRSADAPGTRNYAGVKSAAVDELIEAVTNARSQEE-LEAAVHALD-RVLLWG
NYVVPLYYLD---------RDYIAYWG-DLGRVTTVNPTYGTV----LEAWW--------------------
----

>Beggiatoa/1-572 alba
--AALGY---SPKYPTDFKHFDYVNPDAPKQGDI
TISGFG--------TFNSLNPFLL-------KGVEAVGTTN-----------LLFDTLMVKS---------E
DEPYSVYALLAKEMQLAADK----LSVTFSLNPNARFSDGSPVTAEDVKFSFDTLKGD-KAHPRYRIFWSDI
VKAEVINNLTVKFSFA--KENPELHLIVAYMIPIFSKTAVGEQ-------AFDSLVTT-PLIGSGAYTVTDF
KIGNYIIYQRNPQYWAKDLPTRRGMFNVDKITVKYYKDLSIAMEAFK-AKEFDFITVY--NSKEWARSY-VG
KAFETGEIIKEELPH--RNNAGMQGFVFNLRNPMFQDIRVRQAINLAFDFEWANTNLFYNQYNRCYSYFTNS
ELASPQ---ALPTDAELSLLQSLQAQYPKEFPDK------VLT-TVWQNVNTNP-PNS---LRSNLQKASQL
LTEAGWTLQ--EGILQ
KE-G--MRLSFEFLVAQ---DGFDRIYTPFARNLERLGIKLIYRKV-DLAVYQQLLE---AFNYDMIVTI-F
QQ----NQSP-ANELMNLWHSSSADKQGSNNWIGLKNPVVDALLYKIIYAPNRAE-LVTATHALD-RILLQG
EYVLPNWYTN---------VHRVAYWN-KFGKPAKSPLYY-QSIDWMLATWW--------------------
----

>Mongoliimonas/1-589 terrestris
-------------------MTVLALATGA-LPAFAEPSHGIAMHG---EPALAADFPHFPYVNPDAPKGGRM
ALGFPG--------TFDSLNPFIVKG-------NAPRGLT------DALYGNNVWDTLLMRSADE-------
--PFTLYGLLAATVEVPDDR----SFVEFRLRPEARFSDGTPLTVDDVIFTADLLKTKG-RPI-YRNRFGKV
ASIEKVGDTGVRFVFGDGADR-ELPLLIG-LTPILPAHAID-------PATFDQSSLT-PLIGSGPYTIASV
NAGAQVVLKRNPDYWAKDLPIKRGMDNFDEIRIEYFRDGNAYFEAFK-TGAFDVLIES--DPQRWKTG-YDF
PAATSGKIVREEIAS--GTPKGMAALVMNTRRPLFQDRKVREAMGLLFDFEWINRNLYEGAYSRTGSYFQGS
SLSAL---GVPAGEREIALLAPYP----DAVLP------SVMDGTDAPPVSDGS-GRD----RAKLRKALDL
FAEAGWTLKDG--VLT
NAEG--TPFRFEFMAKT---REEERLALAWQPSLKRLGIDMAIRST-DSSQYYDRQK---TYDFDMIQMLWT

-----ASLSP-GNEQNNRWSSESATTEGTFNYAGVAVPAADAMIDAMLAARERAD-FEAAVRALD-RVLISG
RYVVPLFHLP---------KDFVARWT-HIARPETT-ALTGIQ----PVTWW--------------------
----
>Starkeya/1-579 novella
--------------------------------------HGLSLLG---TPKYPADFKHFDYVNPDAPKAGLL
RLSVDG--------TFDSLNDIIP-------RGTPASGLQ------------LIYDTLMSPAYDE-------
--VATEYGLLAESVRYPADF----STVTYRLRPEAKWHDGQPVTAEDVVWSFEQLTKN---NPRQAYYYSHV
KKAEVTGEREVTFTFDQAG-NRELPQIVG-QIRVMPKHWWTGKDASGKQRDISQGTLE-VPLGSGPYKVKQV
VPGRSISFERVPDYWGAGLPVNIGTNNFAEQRYEYFRDNVVELEAFK-GDQYDFRVET--SAKDWATAY-DF
PAVKQGKVILEEFPD--RASGSMQAFIPNLRRTKFQDQRVRRALNYALDFDGMNRTLFFGQYKRTSSYFQNT
ELAS----SGLPTGLELEILNSVK----DKVPP------SVFTTPYANPPDGGE-DVR----RANLREAAKL
LREAGYQVK---GGKLVD
PQ-G--QPFTIEILLA--S-PAFERVALFYKPTLERLGITVNIRQV-DVSQYINRIR---ARDFDMIVTG-W
GQ----SLSP-GNEQRDFWGSAAADREGSSNYAGIKDSGIDALIERVIYAKDRAE-LVAATHALD-RVLLAH
DYVVPTWNY---------PNTRTARWN-RFGRPD--KLPEYSFG--FPDIWWWDAQKAAQ------------
----
>Pukyongiella/1-581 litopenaei
--------------------------------ETIIKSHGFSEFG---DLKYAEGFAHFDYVDPDAPRGGEL
SYAAQG--------TFDSFNPFTR-------QGRAGARAAEQ------------YESLLIPS---------Y
DEPASYYGLIAESLEYPESQ----DWVIFNLRPEARFSDGTPVTAEDVVFSHEILLDQG-LQSYAEAVRKRI
PKAEALGPHRVKFHFADGYPRRAMITQVG-GTPVFSKAWFEADP---DNRRLDKPRMV-PGIGSGPYVLDSY
DINRRIVYRRNPDYWGDHVNVNVGRNNFGTIRIEYFSDSIAAMEAFK-AGEFTFRQEN--NSKSWATAYN-F
PAITEGHVQKVELPD--GDVPNATGFVMNLDRPQFSDVRVREAVQLAFNFAWTNESLQYGLFQHRQSFWEGT
-DLEAR---NLPQGRELEVLKSLG----DALDPV------LLETAPPVLPEGRA-DRQT--DRKNLRRAMKL
LDEAGWMVG---DDGI
RRNTSGQQLRIEFLSDD---PALDRLASPFVDNLKAMGIAAAYNRI-DNAQFTLRRRER---DFDMISG---
--GYSMSLQPSTG-LYQQFGTEAAAF-SVFNPSGVHGPDIEPLIDNIVSARETEE-LVANVRALD-RVLRWK
RFIVPTWYLG---------KHWVAYWD-QYGYP--ANLPPYAL--GLEDLWWAD------------------
----
>Ruegeria/1-606 sediminis
------------------ILVLALAATQLRAEEKIIKSHGFSEFG---ELKYPEGFAHFDYVNPDAPRGGEL
SYAAQG--------TFDSFNPFTR-------QGRAGARSSDH------------YESLLYPS---------Y
DEPASYYGLLAESLEYPESQ----DWVIFNLRPEARFSDGTPLTAEDVVFSHEILLDQG-LKSYAEAVRKRI
PKAEVLDTHRVKFHFAPDIPRRALITQVG-GTPVFSKKWFEADP---ENRRIDKPRLD-PGIGSGPYVLDSY
DINRRIVYKRNPDYWGDHVNVNAGRNNYDTIRIEYFSDSIAAMEGFK-AGTFTLRQEN--NSKSWATAYD-F
PAIAKGHIVKAELID--GDVPPASGFVMNLDRPQFQDIRVRQAVQLAFNFEWTNESLQYGLFQQRHSFWEGT
-PLEAT---GLPEGRELEVLQGLG----EALDPA------VLTEEPVRAHESGA-GRQN--DRGNLRKAMKL
LDEAGWTVG---DDGI
RRNAEGEVLRIEFLSDQ---PTLDRIIQPFVDNLRAMGIDAIYNRV-DDAQFTLRRRDR---DFDMMVA---
--GYNMSLQPSTG-LYQQFGSEAASY-SVFNPAGVHGPDIEPLIDNIVAAKEGEE-LQANVRALD-RVLRAK
RFMVPTWYLG---------KYWVAYWD-MYEYP--ENLPPYAL--GVEDLWWINADKEAALKASG-------
----
>Enterovirga/1-583 rhinocerotis
------------------------------------ESHGLSSFG---DLTYPADFRHFGYVRPDAPKGGSF
SFQIASLTGNQNFDTFNTLNVYVL-------KGDGAAGMPLT------------FDSLMVRA---------L
DETDAMYGLVARSVRADAER----RVFRFRLRPEARFHDGSRLTARDVAFTVNLLREKG-HP-TLSQALRYV
AGAEAEAEDVVRIDFAPERARDLPLLVAG--LPIFSEAYWKTR-------DFEASTLE-APLGSGPYRVGRF
EAGRFIAFERVKDYWAADLPVNVGQNNFDELRYEYFRDRQVAFEAFK-GGVFTFREEF--TARIWATGYD-F
PAAKDGRVTRETVQD--RTPSGMQGWFFNQRRPKFADPRIREAIGLAFDFEAINRTIMYGAYARTASFFENS
-PYRAE---GKPTPEELALLEPL----RGKVPDE------VFGEPFSPPKSDGS---GQ--DRALLRRANTL
LTEAGCK-R---EGSV
LKLPSGEPLAFEFLDFQ---QALQPHTLPFIANLGLLGIQATSRVV-DAAQYQRRTED---FDFDVTTR---
--RYSHAATPGES-LRQVFGSRAAATRGSPNIAGISDPAIDTLIDAAVSAKSRPD-LVAACRALD-RVLRAG
RNWVPMWYKA---------SHTIAYWD-MFGHP--ETPP-YGL--AAPETWWHDSDKARRI-----------
----
>Granulibacter/1-568 bethesdensis
---------------------------------GVVRTYAVTTVG---APQLPADFKNFPYANPDAPKGGEI
VQAEIG--------SFDSLNPFIL-------RG-VAGP-VGA-----------VWDTLLVSTADE-------

--PETGYAHLARVIEIAADR----RSVAFELRPEARFHDGHKLTAEDVAWTFETLMKHG--RPQQRAYYADV
EKAEAQGPHRVVFHFR-TDQNRELPLIIG-QLSVLPKHWWE-------KRDFTQPLTE-PPLGSGPYQVGKV
DFGRSLTLERVKDWWAANLPTGRGLYNFDRMRTEFFRDPTIAFEAFK-AGQVDIRVEN--IARQWATGY-DF
PAVKEGRIKREQLPW--NLPFGMVGFGMNTRLSKFGDPRVREAIVCAFDFEWANKNLFYGRYQRLKSYYTFT
ECAS----SGLPSKEELALLEPFR----TQLPP------RLFTEPYTLPVTDGS-GNN----RDVLRHCLTL
LKEAGWTVR---EGKLVD
AGG---QQLSFEILLDN---PPFERVLLPYTQSLAKLGIDARIRSV-DPAQAQHLQE---AFNFDMALMQ-F
PE----SDSP-GNEQIGFWSSASAKEKGSNNLMGVSSPVVDALVAKVITAPDRAS-QITACRALD-RVLLWG
WYIMPNWTA---------ATMNVAYWN-RFAHVDKP--VRSGFAL---DSWW--------------------
----
>Curvibacter/1-579 delicatus
-----------------------------------SHAYAQFG---DIKYPAGFAHFDYVNPAAPKGGDI
TLVPPTRL-----SNFDKYNPFTL-------KGSAPPGLGA-----------LVFESLLTGT---------L
DEPTTAYGLLAQDVTVATDK----LSAVFTLNPQARFHNGEPVLAADVKHSFDQLTSL-AAAPQYRTIFGEI
KGVTVLGERVVRFDFR--RVNAELPLIAG-SLPVFSRKWGGGK-------PLDQIVTD-TPIGSGPYRIGKV
NFGKDISYERDPAYWARDLNVRRGLFNFDRITYKIYKDNTAQLEAFK-AGEFDYIQAF--IAREWARAF-TG
KAFDKGELIKKELKH--GNAGDFQGFLFNLRRDKFKDVRVREAIELAMDFEWLNRQLFYNAYTRVRGYFVGS
DFEAQ----GKPSTEELAVLAPLR----RQLPPA------VFTDDVPQPPFTSLDPASGHTLRDNLRRARDL
LAQAGWTYR---DGALR
NAKG--DVFSIEFLDNS---GSMGRVVTPYARNLEKLGIAVNYKVV-DFAILQKRLD---VFDFDVISNR-L
IG----SEAP-GTELLERFGSKAADTEGSMNVIGIKNPAVDALLDQVISAQTRSE-LVARLRALD-RVLRHG
HYAVPHWYGS---------VHRVAWRAGRFEQPAVTPRYYQP-ESWVTSVWW--------------------
----
>Halomonas/1-582 boliviensis
----------------------------------VETVHGLSLYDS---PELAADFPYFPHVNPHAPKGGTI
THTAVGS-------SFDSTNPFII-------RGTPVTGIS------------QIYDTLMASNPNE-------
--PFSLYGLLAKGVRLDPDR----EWIEFDLRPEARFQDGEPVTAYDVVFSLNLLREEG--NPFYASYYAGV
EEAIALNEHQVRFTFNDNESR-ELPLIIA-QLPILPRHYWE-------PREFTSPTLV-AHPGSGPYRISEV
DPGRRIVYQRDEDYWGKDLPVNIGRYNIDRIIYDYYRDRDIAWEAFK-AGLTDFRTDA--RAATWAIGY-NF
PAYEEGLVKRLTVPD--VNPSMMQAFVFNLRKEKFQDPRVREALSLTFDFPWLNTNIFYGTYQRTESFFQNS
EMEA----TGLPSEAELALLEPFR----DELIASHGSSDRLFTEP--LPIDEPI--EL----RERLRKALEL
LREAGYQVED--GMLVN
QDTG--RPLSLEVLLYD---SGLERVVQPMLRNMARLGVQTSLRIV-DINQYLNRQR---NYDYDIVISH-F
PQ----SNNP-GNEQRDFWTSAAAVAPQSRNRMALAHPAVDALVETIISADGREA-LDTATRALD-RVLRWG
FYVIPHYHSG---------ETRIAIWD-KFGYPEPFPA--YAMD---MDAWWVDTQREAAL-----------
----
>Aquitalea/1-584 magnusonii
------------------------VLLLLWAARGMAAPAQAMGY---TPKYAAGFTHFDYVEPQAPKGGQL
VLPAQG--------SFDTLNPFTL-------KGDKADGVQA-----------LLLDTLGVSS---------E
DEPYSCYGLLADDMQLAADK----LSVRFHLNPLARFANGRPVQAADVVASFNTLTRDPAATPLYRVYWADV
QQAVAVDAATVRFDFK--RRNSELHMTLC-QLPVFSRQWIAAGK------SLADVALQ-PPVGSGPYVLERY
DLGKNISFKRNPQYWAARLPVRRGMFNFDRIRYRYYQDETARLEAFK-AGEFDVSAEN--MAKQWARGY-VG
PKFDDGRIIKKALPH--QLSAGMQGFVFNLRRQQFADKRLRQAISLAFDFEWANRNLFYGQYRRSNSFFTNS
DLAAS----GLPGPDELKLLNPIK----DKLDPV------VFG-LPVEPPFTDG-RYG---IRRNLRQARQL
LFEAGWRYEG--GLLV
DHRG--RPLRIEFLTYS---KVYDRVASGWQQNLAKLGITLTVRLV-DPAIYQRRLN---DFDYDMTVVV-Y
GA----SNSP-GNEQLDYHSCQAAQTPGSQNWAGLCDPAVEALLPDFQQFADRRQ-LQAASRALD-RVLRAG
YYLLPNWYLP---------YYRMAWWN-RFGQPAKLPLYY-SPTMWAIETWW--------------------
----
>Donghicola/1-630 tyrosinivorans
------------LLGLGVLSVASYARAQEAEQKIIKTHAYSFWS---APKYPADFPHLDYVNPDAPRGGEI
SVSAEG--------TFDSMNPYSR-------KGRAAAGGGIQ------------YESLLEDT---------A
DTMEESYGLLAESLEYPEDM----SWVTFHIRPEARFSDGSPVTADDVVFSHNLMMEQS-LQSYREAVSKRV
IGVEKLDDLSVKFTFAPDIPRRALIDQVG-GAPVFSKKWFEES-----GARLDESRME-MGIGSGPYILEGY
DINRRVTYKRREDYWGYDLPINKGRHNFDRIRYEYFADSNAAMEGFK-AGAYTFRQEN--SSRSWATSYD-F
PNMRDGHVVKTELPN--GNVPAAAGFVFNLDKPKFQDIRVREAIGLAYNFEWTNAQVQYGLFRQRNSFFENS
-PVAAQ---GVPEGAELALLEKYK----DVLPEG------IQTAEPVMAHTSG--DSQL--DRRNLRKASKL
LEEAGWIAG---DDGI

RR-KDGQTLKLEILEDT---PTFDRIVLPYIENLKALGVEAVYNRV-DPAQYTNRRRDR---DYDMIYT---
--AYTMFERPSTG-LAQQFGSADAAY-SLFNPAGLANEGVDKLIDEIVASTTDEQ-IKPAVSALD-RVLRQE
RFLIPTWFND---------SYWVAYYD-MYEHP--AELPRFDM--GSMDWWWYNAEKGQALKDAGDSPPRTE
LCFDSLMASALDEPDA
>Candidatus/1-584 Paracaedibacter acanthamoebae
----------------------------------LTKAQGFSVFG---ELKYPDGFKHYDYVNPEAPKGGRL
TLSALG--------TFNSLNPFIVRGD-----YPAG--------------VGLTLSNLMSETQDR-------
--AGECYAFGAQSIEIAADR----NYVIFNINPKAQFDDGVKITAETVIWSFNILKDKG-SPL-FRTYYKNV
KKAEKLGTYQVKFYLDDTKNA-ELPLILG-QIYILPKHFYE-------KVDFGSTSLE-IPPSSGPYKIKSM
EPGRSITYERVKNWWGADLPSQLGANNFDEIHYEFFLDSNSQLEAFK-SGRIDIRAEN--SIKNWNTA-YNF
PAANKGCVIREELFH--DRTEPTYGFFFNIRHPKFHDMRVREALTIIYDFGWLNKRLFYSAYNRNLSYFPNS
CFAAH---DLPSE-MELEILTPLK----GQIPE------RVFTEKFGLPDPKIH-TEL----RTTMSRAIQL
FDQAGWHINEG---RMVH
KETN--TPFTFEILIDD---QSNEKICMHYVSTLERIGITAVVRSI-DKAAYTQRVE---NKSFDMIVDLIP
Q-----SNSL-GNEQRDFFGSSKADVPGSRNFAGINNPAIDEVIEKLVQSDSYLQ-LCHRARALD-RLLLWG
FYMIPAWHKG---------SLLVAYWD-KFGHPKVS----SKFNPFNIQTWWYDATKAQALLDKSE------
----
>Gemmobacter/1-609 aquaticus
----------------LLAFAIGFGAAQARAADGIITAHGISTFG---ELKYPADFKHLDYVNPDAPKGGEI
SEWAMG--------GFDSLNPFSV-------KGVGAALSTVM------------LESILTGT---------S
DEIGAAYCLLCETLEYPEDR----SWVIFNLRPEAKFSDGTPLTADDVLFSYETFLAKG-LTDFRTVLAKQV
EKVEVLDTHRIKFTFRADFPKRDLIQDVG-GLPVLSKAQYERD-----GLDLEQSTLK-PWIGSGPYMFDRM
ETGRTLVYRRNPDYWGADLPINRGRNNFDTIRIEYFADGAAAFEAFK-SGVYTFRNEN--SAKQWATGYD-F
PALTKGHAVKAELPN--GNKASGQAFLFNLRREKFQDPRVREAIGMMFNFEWSNQTLFYGQYARINSVWENS
-WLAAK---GAPSPEEAAILKPLVD--EGLLPAS------ILTDEAVMAPVS-S-ERQL--DRKNLRAASKL
LDDAGWPVG---ADGL
RRNAKGEVLRVEMLNDD---PAFDRVINPVIENLRALGIDALNTKV-DPAQMESRTRPP-AYDFDLITG---
--NARSNYISGSE-LKQYFGSETANV-SAFNLMGLQSPAVDRLIDTVLAAGSNDE-LTIATKALD-RVLRAE
RFWIPQWYKN---------THTVAYYD-MYAHP--EALPPYAL--GELDFWWYDADKAAALKSAG-------
----
>Pyruvatibacter/1-600 mobilis
--------------------FTGPAQAESLAPNGGEWRHGMSIFG---DLKYDAGFPHFDYADPAAPKGGEL
RLIPSEAYLNQGFLTFDTLNIFVL-------KGSGAHGMRLT------------FDTLMTRA---------W
DEPDAMYGLAAEAAAIAPDR----MSVAFRLRDGARFHDGSPVMVDDVVFTFDLLKEKG-HP-LLTTAIRDV
VSAEAAGDRTVVYRFQGDLVRDLPFTVA--ELPILSKAYYSDH-------DFSASSLE-PPLGSGPYRVDDV
QQGRSITHRLDPDYWGRDLPVNRGRFNFGTVRFEYFRDRTAAFEAFK-SGEYTFREEF--TSRFWATKYE-F
PAVTDGRVIKLTTPD--NRPSGTQGWFINTRKAKFADPRVRQALGYAFDFEWTNRLHFHGLYTRTQSYFENS
-DMKAE---GPPSEAELALLEPF----RGQVPDE------VFTEAWVPPVSNGS---GQ--DRRMLKRGRDL
LADAGWVVS---DG-K
ARNAAGEVLTIEFLSDT---PTFERVLQPFIKNLGLLGIDASIRQV-DAAQFEERLKD---FDFDIIIR---
--RFSMRETPGVE-LRAFMGSETADQPGSRNLAGIANPAVDALIDRVVHATTRQE-LVTATRALD-RVLRAN
HYWIPQWYKG---------EHNLAVWD-KFGWP--EVKPKYHR--GVIRLWWVDPEKEARL-----------
----
>Pontibrevibacter/1-603 nitratireducens
---------------LAVGLTLTTTSLRAERHEEIITAHGISSFG---ELKYGPDFEHFDYVNPDAPKGGYF
T---TWG-----LGTFDSLRPYIL-------LGNAASSASML------------YDSLMTGS---------A
DEPDALYGLVAETIEYPESR----EWIIFNIRPEARFHDGTPITAQDVADSYQALYEKG-RPTYRLSLFRDV
ESVEVLDERRLKYTFRPDAPLRDMPSMVG-GLPIFPSAWLAEN-------DFAESSME-PVPGSGPYKLQET
DGGRYILYERVEDYWGADLPVNVGRNNFDQQRIEYYADSTVAFEQFK-ALGYTFRAEN--SSRLWATAYD-F
PAIQNGWVMREELID--GTPTGTQGWWFNMRREKFQDPRVRQAIAMMFNFEWSNDSLFYDLYQRTDSFWENS
-YLQAE---GLPSEEELAVLEPVR----GMVPDS------VFTEPAYTPIVSEP-VQL---DRRVLRQAAAL
MREAGYSLQ---D-GV
LVNEHGEPFVIEFLNDS---RAFERIINPFIQNLERLGIRVDAPVI-DQAQAAEREKN---FEFDLTAR---
--RYRMGLTPGQE-LVGIFGSTSANEPDTSNIMGLQNEGVDHLIEQIAHAENREE-LNVRVRALD-RVLRSL
HIWIPQFYSG---------RHFIAYLD-VYSRP--EVQPPFSI--NTD-AWWWDEDKAEVLRAAG-------
----
>Puniceibacterium/1-588 confluentis
----------------------------------VIKAHGVSTFG---DLKYPANFKHWDYVNPDAPKGGEF

S---TWA-----FGTFDSLTPYIL-------KGNAASLSTVF------------YDSLLTGN---------L
DEPDSSYGLVAESLEYPENR----EWVIFHMRPEATFRDGSPVTAQDVVFSYNVLVEKG-RPSFKV-SLKDF
QTVEALDDHTVKFTFNPEGPLRELLMTAG-GLPIFSQAYYENV-------DFTESSLE-PPMGSGQYELLSV
DPGRSVAYKRRDDYWAKDLPVNVGQNNFDVIKVEYFADYTTAFEAFK-AGAYTFREEF--QSKIWATGYD-F
PAVKNGTVKIEELDD--GRPAGTQGFWFNLRREKFQDIRVREAIGLAFNFEWSNESLFYGAYERTDSFWENS
DTLQAT---GMPSDAELALLEPLR----ADLPES------VFTEEAYVPATSSP-NDLA--DRRALRTAGKL
LEEAGWIVG---NDGI
RYKD-GQKLSVSVLNDS---PSFDRIINPMIENLKRLGIEADATRV-DAAESQEREKT---FDYDIVTQ---
--RFSMSSTPGDE-LRQIFSSDSAEVPGSANIAGLANPAVDVLVDKIATATSRED-LDTAVHALD-RVLRAM
HVWIPQWYKG---------VHTIAYFD-QYDRPYTDTPPPFGM--GEASIWWYNAEKAKALRDAG-------
----
>Shinella/1-588 kummerowiae
-------------------------GTKAQETKEPVWRSGVSTIG---ELKRPNGFERFDYVNADAPKGGEL
KLSETG--------TYDTFNPILS-------KGEAATGVTS-----------LVFDTLLKSAEDE-------
--VTASYGLLAEGVSYPDDF----SSATFRLRPEAKWADGQPVTAEDVIFSFEKSKEH---NPLLSNYYRHV
ISAEKTGERDVTFRFDEKN-NRELPNILG-QFPIVPKHWWEGQDAKGNKRDISKSTLE-PVMGSGPYKIASF
QAGGSIRFELRDDYWGKDVNVNVGQNNFGTITYTFFGDKSVEFEAFR-GGNVDFYRDG--SASNWATRY-DF
PAVKDGRVVREEIENPLRATGIMQAFVPNLRREKFKDQKVREAMNYAFDFEDLNKTLAYNAYQRVDSYFWNT
ELAS----KGLPEGREKEILEELK----DKVPA------AVFTTPYTNPVNGDP-QKV----RDNLRKAIGL
FREAGYELK---GNKLVN
AKTG--EPFAFEILLS--S-PSFERTVAPFVASLKKIGIDARMRTV-DASQYTNRVR---SFDYDMIYGI-W
AQ----TLVP-GNEQSDYWGSASVDQQGSKNYAGISDPAIDELIRKIIFAPTRDE-LIATTRALD-RVLLAH
HYVVPLFYS---------KAQRIAYWN-HLVRPA--ELPYYGLG--FPDVWW--------------------
----
>Tranquillimonas/1-583 rosea
------------------------------QSETTTISHGVSAFG---ELKYPPDFPHFDYVNPDAPKGGEM
SFRGVGA-----SRTFDSLNPFIL-------AGEPAQGLERI------------HDTLMARA---------L
DEPDAVYGLLAESVEYPADR----GWVIFTLRPDAQFSDGHPVTAEDVVFTIETLKSEG-SPLYRI-QLEDV
TGAEALSERRVRIDFRDGAATRDLPMEVA-QVAILPAHYYGDV-------DFARSTLD-PPVGSGPYEIADV
RPGRSITYCRTPDYWGAEVPVNVGKDNFDCLRYEYFADHTAAFEALK-AGEYLLHEEY--FSAQWATGYD-F
PALNRGWVKQVELPD--QRPSGAQGFYMNMRRAKLQDVRVRQAIAALFNFEWTNQTLFYGLYERTDSFWENS
-ALEAT---GIPEGRELEILEEFR----GRLPPE------IFTEPPVSPPEGSL-SQI---DRSSVRRAGAL
LDEAGWKVG---EDGL
RRNDAGDVLRIEFVSDS---PSFERVVLPYVDNLRRVGIDARYVQV-DPAQMEERQKT---FDYDITGA---
--RLVSTLTPSTE-LRTFFGSQSADQPGTYNLSGISDPVVDALIARAVQADNREG-MVAAVHALD-RVLRSK
HLWVPNWYKG---------THWLAYWD-VFDRP--ETKPPYDR--GVD-FWWLDDEK---------------
----
>Parvibaculum/1-589 indicum
------------------------VKVAGAGP-----RHGMSFFG---GLKYAQDFPHFDYVNPDAPKGGTL
SQVGPTAAYNAGFTTFDTLNGYIL-------KGNAAQRLDLV------------FDTLMARA---------Y
DEPSAVYGLVAESAALSADG----NVLTFRLRKEARFHDGTPLTAEDAAFSLMLLKRQG-HP-LISQNLREM
KEAVATDSHTLTVTFTGKQTRDLPLFIGG-ELPIFSKAYYTAT-------TFDETTLE-PPLGSGPYRIGDF
KPGRFLTYEQVADYWARDLNVNVGQWNFDRLRFEYFRDRTAEFEAFK-TGAYTMREEF--TSRVWATEYN-F
PAVQDGRVKRLELPD--GTPSGAQGWFINTRRRKFADPRVRQALGLAFDFQWANKNLFYGLYQRTESYFENS
-PMKAQ---GLPSEAERALLAPW----KDELPAT------VFGEPVSPPASDGS---GQ--DRAMLRKAAQL
FVEAGWTIE---NG-R
RVNADGEALTVEFLQDD---PMWERICAGVIKNLRILGVDARFRLV-DSAQYQDRMKN---YDFDLIVQ---
--RYSMSLTPGIE-IRSYWSSAFADVAGGRNLSGIADPAIDALIEKVIAAATPEE-QVSGARALD-RVLRAG
HYWIPQWYKA---------SHHLAFWD-IYDRP--PQKPDYER--GVLRSWWTAPDKA--------------
----
>Sinorhizobium/1-582 arboris
--------------------------------------HATSSIG---EPKYKDGFARFDYVNPDAPKGGEL
RLSENG--------TFDSFNPILA-------KGEVATGVSS-----------LVFETLLKSAEDE-------
--ITTSYGLLAEAVSYPDDI----SSATFRLRAEAKWADGKPVTPEDVVFSFDMVKEH---NPLFSNYYRHV
VSAEKTAEREVTFRFDEKN-NHELPNILG-QFPILPKHWWEGQDAKGNKRDISRTTLE-PVMGSGPYKIASF
QAGGSIRFELRDDYWAKDLNVNVGRYNFRTINYAFFGDRSVQFEAFR-AGNVDFYQDN--SASHWATAY-DF
PAMKDGRVIREEIENPLRATGIMQAFVPNMRREKFKDQRVREALNYAFDFEDLNRSLAHNAFQRVDSYFWGT
ELAS----SGLPEGREKEILEELK----DTVPA------AVFTTPYRNPVNGDP-QKV----RENLRTALAL

FKEAGYELK---GSRLVN
AKTG--EPFSFEILLS--N-PSFERTVTPFVNSVRKIGIDARIRTV-DDSQYTNRVR---SFDYDMIYVV-W
AQ----TLVP-GNEQSDYWGSASVDQPGSRNYAGIADPAIDELIRRIVFAPNREE-LVATTRALD-RVLLAH
HYVVPLFYS---------KALRVAYWK-HLARPK--ELPYYGMD--FPDAWWSKNAAAK-------------
----
>Falsochrobactrum/1-586 ovis
--------------------------------------YSSSLIG---EPKYPANFKHYDYVNPDAPKGGSL
NQVAVG--------TFDSLNPYVI-------QGVAAAGL-------SEFGGGFLYDTLMSDALDQ-------
--ASTQYPLIAEAMQYPDDY----SWVKFKLNPKARWHDGQPITVDDVIWSFDVLKKQ---SPAYNKYYSDV
EKAEKTGENEVKFTFSQTG-NRELPQILG-QLAVLPKHWWEGTDAQGKKRDITRPTLE-IPLGSGAYKIERV
TPGRSIIWTRVDDYWGKDLPVHVGRNNFDRIAYEYFFNEDATWEAFKKGGQYDYRLEN--RAQRWAEQY-NF
PAVERGDIIQKSFPY--HAVGRMQGYFMNTRHDKFQDPRVRKALTYAFDFETMNRLMFFNQYNRIESYFSGN
ELQL----DGAPSPAEQEILETVK----DALPP------EALTEEFKLPVYDSP-QAT----REHLRTALKL
FEEAGWTLQG---NKLVD
AN-G--KQFTIEFLGR--D-PTDERIFNPFVQNLRRIGIDARVRIV-DAAQYQARVN---DFDYDII-TSVI
AQ----SSSP-GNEQRQMWGSAAADFKGSRNYSGIKNPAIDKLIDRVIYAKDREE-LVAATHALD-RALLWN
YYVIPQWYS---------DHINVAYWN-KFGMPE--NQPDYSGID--PFSWWVDPAKEANL-----------
----
>sulfur-oxidizing/1-591 endosymbiont of Gigantopelta aegis
-------------------LLLTVFICCVFISKSALSDYAIAYGY---TPKYPKNFPNFDYVNPGAPKGGRL
DLNGSR--------TFDRLNPFLL-------KGVAADGIDG-----------LVFETLMEQS---------L
DEPYTFYGLLADDIELADDG----LSITYHINPKARFSDNTPVTTEDVKFSFDTLMTNKHVHPAYKITLGNI
SQATIIDRLTIRFSFK--EKNAKLPMLLSSYLSVFPKHWVGET-------DFKDTAMR-IPIGSGPYTLDSY
STGKQLKFKRNPDYWAQDLGIRKGQFNFDEVVYKYFRDNTIALEALK-AGDYDFRHEY--NSKMWARDY-TG
RVFNSNEVLKDNIAH--QNSVGIQGFIMNTRKPLFADKRVREALVLAYDFEWANRMLFYNQYQRNNSYFSNT
ELAAK----GEISPQEKALLMPFEAI----LEPS------VFG-SAWIPPSTKP-PSS---LRQNLRKAIAL
LKAAGWEYK--DGALR
NDKG--QAFRFDLILAQ---KGFERIDAPYARNLKKLGISMEYRTV-DYALYKRKVD---NKDFDMIVSS-Y
PQ----SQVP-GNELEGRWHSKNADVKGSANYPGIKNKAIDVLLEKLSNTTDRKE-IIAITRSMD-RVLLSE
FYLVPHWYIS---------THRIAYWD-KFEFPKTLPKYF-SAEGWMLSTWWF-------------------
----
>Hoeflea/1-596 marina
----------------LIGTFFCLFVAVLGAQAQEQWRHAASVLG---EPKYEKGFDHFGYVNPDAPKGGQL
RLSEEG--------TFDTFNPILS-------KGDIANGMG------------LIFDTLLKSSDDE-------
--VSTAYGLLAESLSYPDDI----SSATFRLRAEARWADGQPVTPEDVIFSFESVKEY---NPLQMSYYSHV
KSAEKTGERDVTFRFDETN-NRELPLILG-QLLVVPKHWWEGEGPDGQKRDISQTTLE-PVMGSGPYRVASF
SAGSTIRYELRDDYWGKDLNVNVGQNNFGAISYTYFGDANVELEAFR-AGLIDYRQEN--SSSRWVTGY-DF
PAVANGSVKREEVENPLRAVGIMQALVPNMRREKFKDVRVRRALNYAFDFEQMNQTLAYGGLTRIDSYFWGT
ELAS----SGLPEGREKEILQELG----HPVPP------EVFTTPYTNPVGGDA-QKN----RANLGKALQL
FKEAGYELR---GTSLVN
AATG--EPFGFEVLLA--S-ASLERSVLPYVNNLKRIGINVTLRSP-DTSQYTYRIR---SFDYDMIWVV-W
AQ----GLIP-GNEQYNYWGSSSADQEGSRNYAGISDPAIDELIRRIVFVKSREE-LVATVHALD-RVLLAH
HYVMPLFYS---------KAYKIAYYK-SLTHPA--EFPYYSIG--FPDVWW--------------------
----
>Pleomorphomonas/1-607 koreensis
------------RLVAACLAALPLVLGAPALADEPAWRTATALGG---EPRYSQGFTHFDYVNPDAPKGGEA
RFGAEG--------SFDSTNVFLG-I-----KGTPTGAVA------------LAYETLFTASLDEL------
-DISASYPLVADAMRYPDDF----AWAEYRIDPAARWQDGQPVTVDDVVWSFDTLKEI---YPTFTSYYAHV
VKAEPAGERIVRFTFDAPG-NRELPHILS-QLYVLPKHWWQGTDAAGKPRNIRETTLE-PPLGSGPYKVTAV
DPGKRVVLSRDPAYWGAKLPVNAGANNFDRLSYEYYLDPTVMMEAFK-GDKYDFRAER--SAKMWATGY-DF
PARADGRVVTLTFPR--SATGVMQALALNLRLPKYQDPRIRRALNFTFDYETLRRTVFFDLYDRIDSYFFGT
-DLAS---KGLPGPDELALLEPLR----DKLPA------SVFTTPYANPVGGTP-EAV----RDNLRQAVAL
FAEAGWTIRD---GKMRN
GS-G--EPFRIEFLT--ND-QLNERYMSPYAKALARIGIDLDYRLV-DDAQYQNLMR---DFRFDMTTAI-W
AE----SLSP-GNEQREYWGSQAADRPGSRNTAGIKDAAIDALIDKVVFATDREA-LVTATHALD-RALLAG
DYVIPLFYS---------RNNFYAYWN-RFGHPA--DLPKYSV--GFPDIWWYDAAKAA-------------
----
>Aliiruegeria/1-611 haliotis

-----------ARLAMSGAALLAMTAFAQATPEGWINSHGITTFTGD-PLKYAADFEHLDYVNPDAPKGGEM
SVWGFG--------GYDSMNPYSV-------KGRAAGLATAP------------YETLLESV---------A
DEIGSNYGLLAETIEFPEDR----SEVIFTLREVATFSDGTPVTAEDVLFSYELFREKG-LPSFRAVLREQV
KEGEVLDERRVRYIFHEDQPKRDLIQTVG-SLPVFSKAHYEAN-----GLDLEENSMT-PFLGSGAYVPDQL
DVGKRATYKRNPDYWGKDLPINKGRHNFDSLRVEYFADYNVAFEGFK-GGSYHFRNEA--SSKNWATSYD-F
PAIKNGHVKKVELHD--GTIATGQSFVMNLRREQFQDPRVREAIGLLFNFEWSNEQLFYGLYARVNSFWENS
-ELAAT---GQPSPEELALLEPLKE--H--LPEG------VLDSEPVMAPTS-S-ARQL--DRKNLRKASNL
LDQAGWIVG---DDGM
RRK-DGKVLKVEFLNDS---QTFDRVINPYVENLRRAGIDAVHSRV-DQAEATNRER---NYDFDIATD---
--QFPMGYVPGAG-LKQYFGSETADS-SVFNSMGLKSPAVDALVDTIIGAQSKDE-LHVAVKALD-RVLRAE
RFWVPQWYKD---------KHTVAYFD-MYEHP--EPLPPYAL--GHLDFWWYNAEKAEKLKAAG-------
----
>Caballeronia/1-612 cordobensis
-------------RALLGFIACALAGSIASVASPAYAVHAIAQYG---EPKYPVGFRHFDYVNPDAPRDGTL
VLANPNRL-----TSFDKFNPFTL-------RGNPAPGLS------------LMFESLTTGS---------S
DEVSTAYGLLADDIAVAPDG----MSTTFHINPKARFSNGDPVTAEDVKFSFETLKSP-KAAPQFSVYFGQI
ARAVVVDPLTIRFEFK--IATREMPLLAG-GIPVFSRKWGMKPD--GTRIDFDKIAFQ-KPVGSGPYLIDRY
DNGRTISYRRNPDYWGAALPVRIGMFNFAHIDYKLYGDPTARLEAFK-AGEYDVLVEY--VARSWVRRD-IG
KRFDSGELIKREFPQ--HNGTGMQGFFMNTRRPLFKDVRVRQALDLALDFEWLNRQLFFNQYKRIDSFFVNT
DLQAK----GKPGEGELAILDPLK----KQLDPS------VFG-EMPRQPDTDP-PGS---LRANLIKAREL
LAQAGWTYR--DGALR
NAKG--EPFVFEILDDTGGGASMEPVAAAFGRNLQKLGITMNFRTV-DYALIQKRLD---AFDFDMTSVR-M
PD----VQVP-GTEQVSRFGSKSADEQGSDNLAGVKSPAVDAILQKVISAQTREQ-LVDATHALD-RVLMHG
YYVVPHWFSA---------THRVAYRN-TLGFPSTLPLYY-GAEEWVISTWWAKPAHAAK------------
----
>Phreatobacter/1-566 oligotrophus
---------------------------------------AIAMHG---EPAGPAALAHRRYVNPDAPKGGRL
VLGIQG--------TFDTLNPFVVRG-------LPVPGAR-----------SYVWETLMQRSYDE-------
--PFTLYPQVAAGLELPDDR----AWVIFHIDRRARFSDGRPVTAEDVRFSYELLRERG-RPN-HRTYFRKV
TRVDLIDSHTIRFDLAGAADR-ELPMILG-LMPVLPRHAVD-------PETFEETTLT-PPVGSGPYTIAEV
RAGESVTFRRDPSWWGADLPINRGLHNVDEVRFDFYRDGNTLFEAFK-KGLVDVRVET--DPGRWASG-YDF
PAVRDGRVVRDTIRQ--GTPKGIRGLVMNTRRAVFEDVRVREALGLLFDFEWTDRTLYADGFERSQSLFEGS
ELSAR---GRPADEAERRLLASVG----ASIRP------DILEGTYEVPVSDGT-GAD----RARLRQALTL
LGQVGYRLDGG---VLK-
GRDG--QPLAFEFLVAT---RDQERLALTFQRFLQRAGIRMSVRNV-DAVQYDRRLR---DFDFDMVDYRWW
NT----SLSP-GNEQAFYWGVSAAATPGSRNLPGIADPAVDRLVAAIVEARTRGD-LVTAARALD-RVVTGG
FYWVPLFHQP---------AQWIARWN-HVGTPAES-SLFGYL----VETWW--------------------
----
>Falsiruegeria/1-616 litorea
---------------LSIGLLLAATGLARAEDEKIIESHGYSFFG---DLSYPADFEHFDYVNPDAPKGGEI
AVSAPG--------TFDSMNPYSR-------KGRAGRYSWMV------------YESLLGEMP-ATGGGLPA
DQIGESYGLLAERLEYDEGK----TWVIFYMRPEARFADGSPVTAHDVVFSHNLFLEQG-LPSYAQAVKKRI
LSAEALDDHTVKFTFATGISRRSLIDQAG-SVPVFPKAWYEEN-----GARLDEPRLD-AAPGSGPYQLDSY
DVNRRIVYKRNPDYWGKDLPINKGRYNFDTIRVEYFADDTAGFEAFK-AGEYTFRSEG--NSKRWATEYD-F
PAVNKGWVKLEEIPD--LTPPTPTGFVFNLGRPALQDKRVREALSLAFNFEWTNESLQFGLFKQRHSFVENA
-PHMAQ---GAPEGAELELLKSLG----DLVPAD------MLTEPSVRAHESRA-KRLN--DRRNLRTAMKL
LDEAGWAVG---DDGM
RRNAAGEKLVLEIPYPSSSSPTLTAVIDGFAKNLVAMGVDARPDKV-DSAQYTLRNRDR---DYDIVFD---
--NYRAFLSAGTG-LMQMYGSGEADF-SLFNPAGLASPLVDAIIERSLVTTTKED-EAAALQALD-RALRYE
RIMVPVWYND---------KSWVAYWD-QYDHP--ENLPEYAV--GVLDFWWYDAEKAAKLKS---------
----
>Sediminimonas/1-590 qiaohouensis
--------------------------------QTIIKAHGVSTFG---ELKYEEGFDHFDYVNPDAPKGGEF
S---TWA-----FGNFDSLSPYIL-------KGNAAALSTVF------------FDTLMTGG---------L
DEPDAMYGLVAHTVEYPESR----EWVIFHMRPEAEFSDGTQVTAEDVVFSYEALRDKG-QPSYRV-LFRDF
KSVEALDKHRVKFTFNPDGALRELLLSAA-GLPIFSKAYYETR-------DFGESTLE-PPLGSGAYMLEEV
DAGNSVTYKRRDDYWAKDLNVNVGQNNFDRIKVEYFADYTTAFEAFK-GGAYAYREEF--QSLIWATAYN-F
PAIKQGHVKVETLPD--GRPAGTQGWWFNLRREKFSDPRVRKAISMAFNFQWSNESLFYGLYERTDSFWENS

-DLQAE---GMPSEAELALLEPLR----DDLPEE------VFTEPAFTPAKSKS-GDVA--DRRVLRRASRL
LDAAGWTVG---DRGI
RTNEAGEPLSVEILNDS---ASFERIINPYVENLKRLGVDVRYVRV-DSAQLQEREKK---FDFDITTR---
--RYAMSMTPGIE-LRGMFGSDAATAQGSNNIAGVSNPAVDSLIKTIENAESRED-LNVAVSALD-RVLRAM
HIWVPQWYKP---------SHNIAYFD-MFERPYDDTPPMMGL--GEISIWWYNPQKHQALIDAG-------
----
>Noviherbaspirillum/1-572 soli
-------------------------------------AHAFALYD---TPKYPAGFHHFDYVNPDAPKGGEL
YLANPDRR-----TSFDKFNPFSL-------KGVAAAGVSN-----------LMFETLAVSS---------S
DETATMYGLLAEDMELAADR----MAMTFRLNPKARFNNGDPVLAADVKHSFDTLMA--KGAPQFKSVFADI
KQCVVVDERTVRFDFR--QLNRELPLIAG-GMPVFSRKWGAR-------TSFDKIQLE-APIASGPYLIERY
DVGRAITYKRNPDYWGDAVPARRGMFNFGSIFYRFYKDDTARLEAFK-AGEFDVVVEY--SAKNWARAY-NG
PKFRSGEIIKRELTH--SNGAGMQGFVMNLRRGQFQDIRVRQALGLALDFEWMNRQLFYGQYKRIHSFFNNS
ELGAR----GMPSEDELKLLEPMR----AKLDPA------VFG-PAPLPPSTEP-PSS---LRANLMQAREL
LRQAGWEYR--DGALR
NAKG--EPLAFEIIDDQ---GALSRVISVYVRNLQKLGVQVHQRTA-DFALLQKRME---EFDFDMTTNR-F
PD----VTSP-GNEMYDMFGSKAADEKGSNNAWGLKDPAVDKLVDALVAANTRRE-LVAAARALD-RVLMHK
YIVVPHWYSS---------THRVAYRN-RFGIPATAPLYY-QADPYVISTWW--------------------
----
>Thioclava/1-609 atlantica
-----------LLLGGIVWTTLALGAHAQDQSGDTITSYGIATLG---SLKYPADFPHLDYVNPDAPKGGEI
SEWAPG--------GFDNYNPYSI-------QGRAAALASAP------------LESLMEGT---------A
DTVGELYCLLCKSLEYPKSK----DWVIFTLRDGIKFSDGTPLTAEDVVFSYEQLRDKG-LSSFRAVISQQV
KSAEVIDPTHVKFTFLPDYPRRDLIQSVA-TLPVFSKAQFEKD-----KIDLSQTSDE-PFIGSGPYMFDSV
KNNRSITWKRNPDYWGKDLPINKGRSNFDKIRIEYFGDYQSAFEGFK-SGTYTFRNEA--SSIIWATGYD-F
PAMQTGEIQKAELPN--GNIASGQAFVINLRRPKFDDVRVREALDLMFNFEWSNETLFYGLYDRVESVWENS
-ELQAK---GKPTPEEVKILEPLAK--D--LPEG------ILTDEAVIQPVS-G-KRQL--DRKNMRKAAKL
LEEAGWKVG---DDGK
RRNAKGEVLSVSILNDS---QTFDRVINPYVQNLQALGIDARMDRV-DDSEFENRRR---SHDFDMITT---
--HLGQDYIPGAD-LQQYFGSKNTD--DVFNAMGLKNPAIDKLISLVEHASTHEE-LVPRVHALD-RALRAL
RFWVPQWYKP---------TYTVAYWD-QYDHP--KTLPPYSL--GELDFWWYDAEKAKKLEASG-------
----
>Tranquillimonas/1-585 alkanivorans
--------------------------------QEVTVRHGISPFG---ELKYPPDFPHFEYVNPDAPQGGTL
SFRGTGA-----SRTFDSLNPFIL-------KGEPAQGLERL------------YDTLLAPS---------L
DEPGAAYGLIAETVEYPEDR----SWAIFNLRPEARFTDGESLTAEDVVFTFEALKEHG-MPWYRI-TLADV
ETVEALDPHRVRFTFREGAATRDLAAEVG-SIEILPAHYYAEV-------PFEESTLD-PPVGSGGFEVAEV
SPGRSITYCRTDEYWGAELPVNVGKDNFDCVVYEYFGDNTAAFEALK-VGEYLFHEEF--TSALWATAYD-F
PAVERGWIKRETLPD--GRPSGAQGFWFNLRREKFADPRVREAIALMFNFEWANQTLFHGLYERTDSFWENS
-ELEAE---GLPEGDELALLEEYR----DQLPEA------VFTEPAYSPPVSSL-SQT---DRRALREGSRL
LDAAGWTVG---EDGL
RRNAQGEVLRVEMVDDS---PAFERIALPFIGNLRALGIDATHTLI-DPAQMQERQEV---FDYDIAIG---
--RLVMQLSPSVE-LRSLFGSEGADNPGTLNLAGVADPVVDALIERIIQAESRGE-LVTRVRALD-RVLRAK
HIWVPNWYKG---------SHFLAYWD-VFGRP--EEKPPYDR--GDD-FWWWDEEKYQSL-----------
----
>Rubellimicrobium/1-586 rubrum
-------------------------------------SHGYNFFG---ELKYPEPDFVLDYVNPDAPKGGEI
AIAAEG--------TFDSFNPFTI-------AGGATAYGAYG------------VESILTST---------A
DDPTSLYCLICSSMEYPEDL----SWVIFTLRPEARFADGSPLTAEDVKFSHDLFMEQG-LESYRLAAGGMV
SNVEVLDPHRIRFTFADDAPMRDRIGLAG-GLSVLSKAWFEET-----GQRLDEPSDQ-PLLGSGPYMVENF
DFSRRILIGRNPDYWGKNLPINVGRNNFDRIRVEYFADSNVAFEAFK-AGEYTFRVEN--SSLLWATGYN-F
PALEQGWVFKGELPN--GGLPVAQSFVFNLRRETFQDPRVREALGLMFNFEWSNETLFYDLYTRTVGFWNES
-ELMAQ---GAPSEGEVALLQPLVD--EGLLDAS------ILTEEAVVPPVSSP-ERQA--DRGNIRQASQL
LDEAGWIVG---DDGV
RR-KDGQTLDVVILEDS---PTFDRVINPYVENLQRIGVNARLDRV-DPAQMTQRRD---ESDFDMTAW---
--GFQMSLEPSTG-LEQWFGSESAAE-SNRNLMGLQDPAVDRLIAIAVAADTRET-MVTAVNALD-RVLRAK
RFWVPQWYKA---------SYTVAYYD-MFEHP--ETLPPYDL--GYLDFWWYNADKAEALRTAG-------
----

```
>Prosthecomicrobium/1-584 hirschii
------------------------------------ETHGLSIFG---DLKYGPDFKAFDYVNPAAPKGGRL
VTVPSQWAYNQNPSTFNTLNTLIL-------KGDAPVGMERT------------FASLMTRA---------L
DEPDAVYGYVARSAAVRDGG----ATFVFRLRPEARFHDGSPITAEDVAFSLTTLKEKG-HP-SVSLTLRDM
RAVEVEAADTVVVRFAPTRSRGLPLIVAT--LPILSKAWYATR-------DFEQTTTE-AALGSGPYRVGRF
EAGRYIEYERVVDWWGRDLPVAAGHNNFDIVRYEMFRERTAAFEAFK-AGLYFLREEF--TSLVWATQYD-F
PALADGKVVRFELED--RSPSGAQGWYLNMRRPKFADRRVREALGLVFDFEWTNKNVFFGSYKRISSFFVNT
-EFEAA---GLPSAEELALLEPF----RGKVPDE------VFGEPWTAPVTDGT---GR--DRTLLRQAAKL
LAEAGWT-A-----------------------------------------------------------RDGT
LRNAKGESLTVEFLDSD---TSFQRVTGPYLENLKRIGVDASVRVV-DPSQYQKRVDE---FDFDVITR---
--RSSFPPTPDED-IRQAWHSESVDRKGSRNIGGIRDEAVDALILKALGAETRAE-LTVACRALD-RVLRAG
RYWIPQWHKA---------SHWIAMWD-LYDRP--SVKPRYAR--GIETTWWVNRDKAEKL-----------
----------------
>Boseongicola/1-602 aestuarii
-------------MSLRAATVFAALVFAVPALAENTVSHGYSAFG---DLKYGPDFSHFDYANPEAPKGGTM
SQRQLYG-----TPTFDSLNTFIIK------GDSAPEVGVHM------------YDSLMVRA---------Y
DEPDAYYGLVAETIEYPEDL----SWVAFNLRPEARFHDGEPVTASDIVFTVDALKTMG-HPYYRN-LLSDV
SLVEAEDKHRVRFDLGPGAG-AAFPGDLA-SLPVLPEHFYNAH-------PFDETWIV-APLGSGPYLLDRV
DPPRTVRFCRDPDYWARDLPVNVGRNNFDCFAYEYFADDTVGLEAFA-AGEYTMRVEY--RSASWATGYD-F
PAVQRGWVKQMIIPD--GRPANAQGIWFNLRNPVLQDRRVRKALELAFNFEWTNDTLFYGTYKRTDSFFENT
-DMQAS---GLPEGGELALLNEFR----DQLPDE------IFTDPAYVPYSGSP--QPR--DRSAMRQASAL
LDEAGWTVG-----------------------------------------------------------SDGI
RRNSDGNVLSLEFPEDS---RSLERVMVPFVQNLQALGIDTEFELI-EPSSLTERRQQ---FDFDLSAT---
--AWSVAVTPSAE-LRAFYGSKAASAEGSNNLTGLADPVVDALIERAVNAGSRDE-LVTAARALD-RVLRQK
LLWIGNWHLG---------AHRVAVWD-IFGIP--ETPAPYDFN-RNVDFWWFDQAK---------------
----------------
>Cucumibacter/1-579 marinus
-----------------------------------------SLVG---EAKYPEGFAHFDYVNPDAPKGGNV
RLSSLG--------SFDSFNTVPA-------KGETAPGLG------------LIYDTLLTPSMDE-------
--VSTYYVALAEAFTYPEDF----SSVTYRLRPDATWHDGVPITVDDIVWSFNKATEL---NPNLAAYYENV
SAVEITGDRDVTFSFDSTG-NRELPQIMG-QMPILPKHWWEANGPDGEPRDLGASTLE-PPLGSGPYRIKDF
VAGRTITYERVEDYWGEDLPVNIGHNNFDTITYEEFRDGTVMFEAFK-GDEFDWWSEN--RAQRWAQAY-DF
PAVNQGRIVRERFENPMRDEGVMVGFILNTKRPPFDDVKVREALNYAFDFETLQRDLFFGEYERVDSFFFPT
ELAS----SGLPEGEELEILESVR----DLIPE------KVFTTPFENPVNGSP-TNL----RANLRIALDL
LAEAGYTLD---------------------------------------------------------GNRLVD
ENGE--Q-LSFEVILS--G-PTIEPVALSWLDNLERIGIAGSVRSL-DSSQYVNRLI---SKDYDVVYYG-L
RQ----SLSP-GNEQRRFWGSQSADVDGTFNLAKLTDPGVDALIDKVIFADDRET-LVAATRALD-RVLLAH
HIVVPSYAA---------SDERIARWD-RFSHPD--TLPEFSIG--FPSVWWYDEEKAAKV-----------
----------------
>Labrys/1-593 okinawensis
---------------------------------------GLSLLG---EPKHKEAFDHFDYVNPDAPKGGRV
RLAISG--------TFDNFNIALMAINGVSVKGNIEAGVT------------NVYQNLMTSAQDE-------
--ASTEYGEIADAVRFPDDI----SSVTFRINPKARWNDGTKLTPEDVVWSFEVQKAN---NLQTAQYYHDV
TKAEVSAPDQVTFSFSTKG-NRELPQILG-QLYVMPKHWWLANGPDGKPRNVANTTLE-PPLGSGPYRLKSF
EAGRGAVYERVKDWWGADLPTAKGHYNFDEIRYDYYTNAQQLPELFK-SDSYDFRLEN--VIANWMTLY-NI
PAVTEGRIVKQDFPS--NTIGRMQAFAFNIRRPKFQDVRVRKAFNLVFDFETLNRTIFFNKYKRIGSYFAGL
DELSS---SGLPQGQERDILETIK----DKLPP------EVFTTPYANPVNGSD-EAQ----RANIRAAIGL
LKEAGWEFKD---------------------------------------------------------RKLTN
IGNG--EVMKVEILL--SS-DTFTKITLPYVENLKRLGIEASVRVV-DETQFNQREN---NRDFDMILFG-V
GQ----TQSP-GNEQRDYWGSAAADKPTSRNIIGIKDPAVDALADRIVYAKDRAE-LVATTKALD-RVLLAG
NYVVPMFYS---------DVWHTLRWN-RFGQPA--VLPSQSPTGGFPTVWWYDSALAAKTGAA--------
----------------
>Paenirhodobacter/1-584 enshiensis
---------------------------------------AIATLG---DLKYAPDFAALDYVDPAAPKGGEI
SEWAPG--------SFDNYNPYTL-------EGRPAALSSAP------------HETLLTPT---------S
DTVGEAYGLLCESLEYPPSK----DWVIFTLREGIRFSDGSPLTVEDVVFSFEQLRDKG-LSSFRAVVAQQI
ESCEALDARRVKFTFVAGYPRRDIIQSAG-GLPVLSKAQFLRD-----GIDLAKASDK-PLIGSGPYMFDSA
RGGRTVVWRRNPDYWGADLPVNRGRANFDRIRIEYYGDYQAAFEGFK-AGNYTFRNEA--SSQAWATGYD-F
```

PALNKGQVVKAELPN--GNVASGQCFAINMRRPQFQDIRVREALGLMFNFEWANKALFYGLYARINSIWENS
-ELAAQ---GKPTPEELEILTPLAH--E--LPEG------VLTDEAVMAPVS-G-ERQL--DRANMRRAAIL
LDAAGWTAG---GDGL
RRNPEGELLRVEILNDD---QTFDRVITPYVENLRALGVDARMARV-DDAQYQTRRG---NHDFDMITT---
--QLGQELIPGAN-LQQYFGTGGVK--DVFNAMGLSNPGIDALIRLVEEAQTHAE-LVARVRALD-RALRAL
RFWVPQWYKD---------KYTVAYYD-MFEHP--PALPAHAL--GELDFWWANADKGAKLKAVGAFG----
----
>Variibacter/1-595 gotjawalensis
-----------------------------LAAQEATETHGLSIFG---DLKYPADFKQLDFVNPQAPKGGIF
SEIKTTIAYNQNFLTFNTLNAYIL-------NGDGAQGMELT------------FATLMARA---------T
DEPDAIYGLAARAVRVSADK----LAYTFLLRPEAKFHDGTQLTAHDVVFSLTTLKEKG-HP-NIQQMLRDL
VEAKADDDASVTLRFAEKRARDVPLYAAT--LPIFSRAYYATK-------TFDSTSLD-IPLGSGAYRVGRF
EAGRYNEFERVKNWWGADLPIMRGQLNFDVVRYEYYRDREVAFEGFT-GKSYLFREEF--TSRIWATRYE-F
PAMKDGRVKREVLVD--ETPSGAQGWFINTRRERFQDRRVREALINAFDFEWTNKNLMYGSYNRTHSVFQNS
-DMMAT---GKPSAEEMALLEPF----RGKVPDD------VFGEPYLPPVSDGS---GA--DRVSLRKATQL
LNDAGWT-I---RDGK
RRNAKGEQLTLEFLIDE---PTFQPHHMPFIKNLGTIGIDATLRVV-DAVQFRRRLDD---FDFDATVQ---
--RFSFSTTPGDS-LRTYFSSQAAAIKGSRNLAGMADPAIDMLIEKIIAADTRPE-LVFAARALD-RVIRAN
RYWVPHWNKA---------SHWIAYWD-LYSRP--AAKPKYAR--GVMETWWYDRDKAAKTDRAG-------
----
>Formivibrio/1-587 citricus
-----------------------FVLVLLALAQHAQAGHALALGY---TPKYPASFTHFGYVNPDAPKGGEV
VLPNPDRR-----TSFDSFNPFVL-------KGTPVAGLSP-----------LMFESLAVAS---------L
DEPATVYGLLAEDMAVASDG----LSITFRLNPAARFNNGDPVLAADVKHSFDTLNSK-AAHPSYRTRLAEV
AAVEVTGERTVRYRFR--RPNPDLPLMVA-SLPVFSRKWGNS-------KPLDKIVLE-TPIASGPYRIERY
DLGRSIVYQRRSDYWAQQHPARRGTFNFSRIVYRFYKDDVARLEAFK-AGEFDFLVEY--TARNWARQY-HG
PKFRSGELLKKEFPH--SNTAGMQGFVLNIRKPQFADKRVRQALGLALDFQWMNRQLFYSQYTRIDSFYSNG
ELAAR----GTPSAAELKLLEPLR----SQLDPA------VFG-PAPVPPSTEA-PAS---LRDNLRRARDL
LAQAGWTYR--DGALR
NAQG--EPFVFEWLDDG---GSMSRVFAVFERNLNKLGITVKGRQV-DYALYQRRLD---EFDFDLISLR-F
PD----TQSP-GIELYDYYSSKAAKEKGSSNVIGLQEPAVDKLIDAVVKSRTRAE-RVTAVHALD-RVLRHG
YYIIPHWYSA---------SHRVAWKN-RLAWPQKLPLYY-EAGEWMLETWW--------------------
----
>Ramlibacter/1-574 rhizophilus
-------------------------------------AHAYAQFG---DVKYPPGFSHFSYVNPDAPKGGEI
RMVPPTRP-----TNFDKFNPFTL-------KGTAPYGITT-----------LMFDSLLTGN---------S
EEPTTAYGLLAEDVSVAPDG----LSATFRLNPKARFHNGKPVLAADVVHSFRTLTSK-QAAPQFRTIYAEV
KGVEALDERTVRFDFA--SPNRELPLIVG-GMPVFSRDW-------GQGKPFDQVVSE-VPIASGPYRIADP
RMGRDITYVRDPNYWAADLNVRRGHYNFDRITFKIYLDETSRFEGLK-AGEFDFMREF--ISRNWARQY-TG
KAFESGELAKRPFPN--QNPGDFQGYVFNLRHPKFQDVRVRKALGLAMDFDWMNRQLFYNLYKRVQGYFPNS
EFHAE----GLPKPDELAILEPLR----DKLRPE------VFG-PAYVSPSTKP-PHS---LRDNLRQAQKL
LAEAGWTYR--DGALR
NEKG--EPFTIEFLTDQ---PSLVRIVTPFEKALEKLGIQLIYRSV-DFSLAKQKMD---EFDFELTTTR-L
PG----STAP-GTELLERWGSEAAKTKGSSNIWGIADPAVDAILQKVVTAKTRPE-LSAAMRALD-RVLTHG
HYSIPQYYGS---------DFLVGFRPRRFELPATIPPYYDV-HNWAMSTWW--------------------
----
>Polymorphum/1-604 gilvum
---------------LVLAVCTSALAGPSRAADEPAWRHATALTG---EPKYGPDVPHFDYVDPDAPKGGLV
RLSATG--------GFDTFNVLL--P-----KGNPAPGLG------------LIYESLMESSYDEF------
-DISAQYGVIAEAMRYPDDF----SWVEYRLNPAARWHDGAPVTTADVLWSYEKAVEI---NPQQRFYYHNV
VKAEERPGGVVRFEFDASG-NRELPHIMG-QLLILPKHWWEGTNAAGVKRDIASGTLE-PPLGSGPYRIKDF
AANRQIAYERVPEYWGKDLPIRVGTNNFGEIRYVSFLDQAVELEAFK-GDQYDWREER--SASQWSKAY-DF
PAVRAGRVVLEEFPD--RGSGVMQAFVPNLRLEKFADPRVRRALNYAYDFETTNEIVSANLLKRIGSYFAGT
-DLAA---TGLPQGLELEILEEVR----NQVPP------EVFTQEYKNPVGGNP-QNV----RANLREAVKL
LQEAGYRLDG---RRMVD
AG-G--QQLTIEFIY--AD-KSAERSLLPYSENLKSIGIRADLRLI-DLPQYINRIR---SRDFEMATLV-W
GQ----SLSP-GNEQRDYWGSDSADRPQSRNYAGIRNPAVDKLIDRIVFARTREE-LVAATHALD-RVLLWN
HYVVPQFHS---------DVDRTARWN-RFGHPQ--EIPEFTH--GFPTIWWYDEALAAK------------

----
>Plasticicumulans/1-561 acidivorans
---PKYPTDYTHFEYVNPQAPKGGTF
VLPAIG--------SFDRLNPFLL-------KGTVADGVTE-----------LTCDTLTEQS---------R
DEPFSAYGLLASDIAVEADR----LSAVFTIRPEARFNDGTHVTAADVKATFDALMST-RAHPQYRVYWADI
VAAEVIDAQRVRFRFA--RVNPELHLIIG-QMPVFSKNWILGR-------NFDELILE-PPLCSGPYIVDAY
SLGRSINFRRNKQYWAQDLPVRRGQYNFDNIRFEYYRDPDVALEAFK-AGEFDYIQVN--IAKQWVRDF-TG
PKFDSGELRKRELEH--HNDAGMQAFVFNLRKPLFQDLRVRKAIALAFDYEWSNQNLFFGLYRRTNSYFANS
ELAADS---EAPRGEEREMLTYFRDS----LPEF------VLT-GRWEAPVNID-AAA---LRTHLREAQTL
LMDAGWTMQ--DGVLM
KE-G--TQLEFEMLLAS---RTFERVMAPFAKNLERLGIHMSYRTV-DASLYQQRVD---HFDFDMLVHV-F
SQ----SQSP-GNEQITYWHSSTADLPGSNNLIGLKNPIVDALVENLVYAQSRQQ-LVSAAHALD-RVLLAG
EYVIPNWYSP---------VFRVAWRD-RFDYPDKLPLYY-QPVEWALSTWW--------------------
----
>Hansschlegelia/1-589 beijingensis
--------------------------------QADGWRHGLSLLG---DVKYPADFKHFDYVNPDAPKGGSV
RLGAFG--------TFDSFNGFIP-------KGSEAAGLS------------LLYDTLLTESADE-------
--PATEYGLVAESVRYPADF----SSVTYRLRPEARFNDGSPITVEDVIWSFETLKRI---SPLRARYYHDV
VRAEQTGDREVTFTFSGPG-NRELPQITG-QFPILPKRWWEGVDAQGRKRNIEEGTLE-KPLGSGPYRIKDF
APGRWISYERAPDYWAAKLPVRVGTNNFDEMRWDYFRDTTVLLEAFK-GDQIDYRMEN--SAKNWATAY-DF
PAVRQKRVILEKFAD--SQSGRMQAYAFNIRRDKFKDPRVRRAFNLAFDFEEINKTIMFGQYVRIDSFFFGT
DLAAP---KAPPEGLEKEILESVR----DKVPP------EVFTTPYRNPVGGSP-EAA----RANLREAFDL
LKQAGWTVSRG---PGGKPALR
NAKG--ETFTVEFIY--GD-PSAERILSFYAPALERLGVQVTQRLL-DDSQYVNRVR---SFDFDVITMG-W
GQ----SLSP-GNEQRDQWGSQAADTPGSQNVVGIKDPGVDALIDRVIYAKSREE-LTAATHALD-RVLLAH
DYVVPQFTS---------LEDRTARWN-RFSRPD--KLPPRGS--MFPTVWWWDAEKA--------------
----
>Jhaorihella/1-585 thermophila
-------------------------------------SHGYSFYG---DLTYGPDFSHFSYVNPDAPKGGEI
SIAALG--------TFDSMNPYSR-------KGRRGQLSWIM------------YESLLGDG--------PA
DTYGEQYCLLAESVEYPVTK----DWVIFHMRPEARFSDGTPVTAHDVLFSHNLLLEQG-LPSYAVAVKRRI
PHAEALDDYTIRFDFTPGISRRSLIDQVG-SVPVWSRKWFEET-----GARLDESRLD-TPPGSGPYMVDTV
EVNRKIVYRRNPDYWGRDLPINRGRHNFDAIRVEYFGDDTAAFEAFK-AGVYTFRVEG--DSKKWATGYD-F
PKVRDGSVVKTELPD--GTPPTPTGFVFNLGREVLQDKRVREAIALAYNFEWTNESLQYGLFKQRASFTQDT
-PLMAT---GVPEGAELEFLKSLG----DLVPPE------MLTEPARMPHTSSP-KRVT--DRRNLRRAMKL
LDEAGWPVG---PDGK
RRNAAGQPLTITFLFNSASDGTLGAVVEGFMSNLRRMGIDARLEKV-DAAQYTLRERDR---DYDLIFD---
--AYAPFLGTGTG-LLQRYGSSEAEF-SLFNPAGLASPLVDAIINASLMAKSREE-EVTMLRALD-RALRYE
FFMIPVWYKA---------NYWVAYYD-QYEHP--DPLPPYDL--GFLDFWWFNAEKHAAL-----------
----
>Silicimonas/1-589 algicola
--------------------------------QDTTVSHGISAFG---ELKYPPDFAHFDYVNPSAPRGGTM
SFRGTLA-----SQTFDSLNKFIL-------AGEAAQGLELI------------YDQLMVRA---------W
DEPDALYGLLAETIEYPADR----SWVIFKLRADARFSDGMPVTADDVAWTIETLKTKA-NPLYRL-AIEDV
EGAEVLSPREVKVTFRAGAQTRDLISTVA-ELEVLPKHYYQAN-------DFERSTMV-PPVGSGPYVVSSA
DPGRRITYCRNPQYWGASLPVNRGTNNFDCYRYEYFSDSTAAFEALK-AGVYLFHEEN--FSALWATGYD-F
PSLTRGWVKRETLPD--GRSSGAQGFWFNLRNPKFQDRRVREALGMMFNFEWSNETLFYGLYERTDSFWENS
-HLQAA---GALEGEELAFLEAWR----DRLPET------VFSEPAFVPPVSQP-NKT---DRAMVRRANAL
LEEAGWVVG---SDGV
RRNEAGEVLSVEFLNDS---PAFERIILPFIENLKAVGIDATLNMV-DPAEYEERQER---FDYDIVSG---
--RYVLPVSPSVE-LNVLFGSNSANDPGSANLSGIADPVIDEIIQQVLAAEDRET-LDVRVRALD-RVLRDR
MIWIPNWFKG---------EHWVAYWD-VFDHP--ATKPPYTR--GVD-FWWWNEEKFRALKSAG-------
----
>Oxalicibacterium/1-570 solurbis
---------------------------------------AFSLYD---TPKYPANFTHFDYVNPDAPKGGDL
YLANPDRR-----TSFDKFNPFSL-------KGVAAAGVSN-----------LMFETLATGS---------A
DEVATMYGLLADDMTLAPDR----LSMTFHLNPKARFNNGDPVLAADVKYSFDTLMA--KGAPQFKVIYAEV
KGCTVVDERTVRFDFK--SRNHELPLLVG-DVPVFSRKWAQG-------TDFDKIQLT-PPIASGPYLIDRY

DTGRAITYRLNPDYWARDLPVRRGMFNFERIHYRFYKDDLARLEAFK-AGEFDVVVEY--SAKNWTRSY-QG
KPFEQGRIVKREFVH--ENTAGMQGFIMNLRRPQFQDVRVRRALGLALDFEWLNRQLFFNSYTRIYSFFNNS
PMAAT----GLPSADELALLEPLR----ARLDPA------VFG-PAPVPPTTTP-PHS---LRSNLLEAVEL
FRQAGWTYR---DGALR
NVKG--EPFTFEILDDQ---SAMSRIISVYARNLQKLGIQVTQRTA-DYALIQQRMD---DFDFDMTSLR-F
PD----INSP-GNEMFDMFGSKAADEKGSSNYWGLKDPAVDSLVASLVKADTRRQ-LTAAARALD-RVLLNK
AIVIPHWYSS---------THRIAYWN-RFGMPERLPLYY-RADPYVISTWW--------------------
----
>Salipiger/1-606 aestuarii
----------------ILALGLALSGPALAQQDDIITAHGVSSFG---ELKYPEGFDHFDYVNPDAPEGGEM
S---ISA-----FGTFDSLTPFIL-------KGNAAAMSSVF------------YDSLLTGT---------L
DEPDSMYGLLASSVEYPESR----EWIVFHMRPEAKFRDGSPVTADDVVFSFNTLVEKG-LPALRV-QFKDI
TNAEALDDRTVKFTFNPDGPLRELLMTAG-SLPVFSKAYYDTR-------DFAESSLE-PPMGSGQYDLLSV
DPGRSVAYKRRDDYWGKDLPVNVGQNNFDVLRVEYFADYTTAFEAFK-GGAYTYREY--QSKIWATGYD-F
PATQDGTIKVETLPD--GRPAGTQGFFFNLRRDKFADPRLREAIGLAFNFEWSNESLFYGLYDRTDSFWENS
PTLQAS---GMPSDAELALLEPIR----EHIPET------VFTEDAYVPPVSSP-DDLA--DRGSLRRAGRL
LNEAGWTVG--DDGY
RYKD-GQRLTIEMLNDS---PSFDRIINPYIDNLRRLGVEATANRV-DAAEAQERQKN---FDYDIVTL---
--RFAMSQTPGDE-LRSVFGSDTADVPGSANISGLKNDGVDQLIRIVADAQSRDE-MTVAVHALD-RVLRAL
HIWVPQFYKG---------VHNIAYLD-VYDRP-SDTPPAQGL--GNMSIWWWDDAKADALRAAGK------
----
>Lacisediminimonas/1-594 profundi
--------------MVRLLSTLLFLAGLMA-GTHAWGAHAFALYG---EPKYPAGFRNFDYVNPDAPRGGEL
FLANPDRR-----TSFDKFNPFSM-------KGVAAAGLSN-----------LMFETLATSS---------S
DETATMYGLLAQDMVLAPDR----MSMVFRLDPKARFNNGDPVLAEDVKYSYDTLLA--KGAPQFKSIFADI
KQCVVLDARTVRFEFK--SLNRELPLIVG-GVPVFSRKWAAG-------TPFDKIQLA-PPIASGPYLIDRY
DVGRSIVYRRNPAYWANEVPTRRGMFNFERIVYRFYKDDVARLEAFK-AGEFDVVVEY--SAKNWARAY-NG
PKFRSGEIVKRELKH--SNGAGMQGFMMNLRRPQFRDPRVRQALGLALDYEWMNRQLFYNQYKRIYSFFSNS
ELGAT----GMPGAGELKLLEPLR----AGLDPA------VFG-PAPVPPDTEP-PAS---LRANLLKAREL
FRQAGWEYR---DGALR
NASG--EVFGFEILDDQ---SALSRVISVYVRNLKKLGVQVTQRTA-DYALVQKRME---EFDYDMTTQR-L
PD----VTSP-GNEMFDIFGSQAADIKGSNNVWGLKDPAVDKLVAALVAAETREQ-LVSAARALD-RVLLHK
YIAVPHWFSP---------THRVAYRN-RFGIPDKAPLYY-QADPYVISNWW--------------------
----
>Sinisalibacter/1-595 lacisalsi
---------------------------TMAARSAETVTSHAFSYFG---TPKYGADFAHLDYVNPDAPKGGEI
ALWAMG--------TFDSFNPYSR-------KGVSGWGASIN------------IERLMTST---------A
DEIGTSYCLICETLEYPEDH----GWVTFTLRPEAAFADGSPVTAEDVVFTHELFMEQG-LPSYREGVSRII
EKIEALDARTVKFTFVEDSTKRDRIGQAG-ATPVMSKAWFEEN-----GARLDESRLE-MALGTGAYMLDSY
EVNQRIVYRRNPDYWATDLPIMKGRNNFGSIRVEYFADSDAAFEAFK-AGAYTFRIEN--SSKQWATGYD-F
DAAKNGEVVTAELPD--GQQASGQAFVFNLRREKFQDIRVREAISRMFNFEWSNDTLFYGLYARVNGFATNS
-YNEAT---GLPSPEELAILEPLAD----ILPEG------VLTEEVVMPPVSGA--RQL--DRAQLRAASAL
LAEAGWEVG--EDGL
RRNAAGEVLSVEFLEYS---PSFDRVIQPFVANLKSLGVDAKLNRV-DTAQYIERSR---SFDFDIMTD---
--HLSIGYEAGSG-LEQAFGSREAEY-SLFNPAGIAHPAVDAIIKVAANAESRDE-MVAGMKALD-RTLRAL
KFVVPQWFKD---------KHTVAYYD-MFEHP--GTIPPYDL--GYLDFWWFNEEKHAALKASG-------
----
>Youhaiella/1-595 tibetensis
-----------------------------AQAQHGPWMSAVSMMG---EPKYKDGFAHFDYVNPDAPKGGLV
RLSSEG--------GFDTFNPILP-------LGEPAVGMG------------LVYETLMTPSSDE-------
--VFTKYGLLAESIAFPPDY----SSVSFKLNPNAKWQDGEPVTAEDVVWSFNKLVEL---NPTRQQYYADV
KSAEITGDGEVTFTFDKTG-NRELPLIMS-EIPVMPKHWWEGKDASGKQRDIGASTLE-IPVGSGPYKIKSF
TAGRTLTFERDPNYWGANLPVNIGQDNFDQIRYEYFRDTDVEFEAFK-GDQFDWWDEN--RAKRWATSY-DF
PAVKDGRVIQEMFENNYRDSGVMVGFIYNLRQEKFKDERVREALNYAFDFEELNRTLFYGQYSRISSFFYGT
ELAAP---AGLPTGKELEILESVK----DKIPA------SVFTTPFANPVAGDS-TKL----RANLRHALQL
LGEAGYKLN---GNQLQD
ANGN--Q-LSFEILLN--G-PTIEPVASAFANNLRSIGVNANVRSV-DSPQFINRLR---TYDFDVVYSG-W
GQ----TLSP-GNEQRFFWGSEAAKQQGSQNYSGISDPGVDALIDKIVFATDRET-LVAATHALD-RVLLAH

NIVVPSYTL---------RASRIARWN-RFSHPE--NLPEFSIG--FPTVWWFDKDKAAQTGGA--------
----
>Sulfitobacter/1-607 algicola
----------------LFLIALFWMATALFAEEKIIKSHAYAILG---EPKYAADFEHFDYVNPDAPKGGEM
STWTQG--------TFDSFNPYSL-------KGRAGALSSAS------------IERLMMSP---------A
DESGVRYGLLAESLEYPESR----DWVIFNMRPEARFSDGTPVTADDIVFSHELLMEQA-LPSYREAISKLV
QKVEAIDTHRVKFTFMPDVPRDGLISQVS-TNPAFSRKWFEEN-----NMRLDESRLV-QNLGSGPYVLDTF
NVNRNIVYRRNPDYWGKDLPVNRGRHNFDKIRIEYFTDNNAALEAFK-GGVYAFRLEQ--SALTWATQYD-F
PAVQNGRVIKEEIAD--GNLPISEGFVFNLNRENLKDPRVREAISLMYNFEWTNESLQFGLYSQRHSFWENT
-DFAAT---GIPEGAELAFLRSLG----DLIDPA------ILTEPAVMAHTSG--ARQL--DRRNLRRATQL
LQDSGWEIG---DDGK
LRNGDGQAFNLEFLTDD---PEIERLASGFVDNLNGLGMNARLTRV-DPTQYTNRTRAR-PREYDMIFD---
--QYGTSLVPGLG-YQQQYGSEDAYV-SNFNPAGFADPGADAIIEKLIASKTPEE-FAVTTKALD-RVMRAE
RFIIPTWYKS---------NHWVAYYD-QYEYP--ENMPPYAL--GYLDFWWYNADKGQALTDAG-------
----
>Ahrensia/1-577 kielensis
---SLIA---TSKYADHFSHYDHANPDAPKGGTV
NLPALG--------TYDSFNPYII-------KGNPAAGFAS-----GGFGGGLLYDTLRAQSVDE-------
--PSTSHPLISESFTYPEDD----SFAIYRLNKAAKWHDGEPITPQDVVWTFDVLKKY---NQLYREYYKNV
VSAEAINETDIKFSFDEKN-NRELPNILG-DMPVLPQHWWEGTDANGNKRDISKSTLE-PPLGSGPYKIASF
DPGVSIRWERVKDYWAADLPVNKGRKNFDSYNYTYFLDSTPIWEAFKKGGIQDIHTET--SSQRWASEY-TF
PAFKEGKVKKRTFPT--TSPQPMQGFAMNMRRDKFKDVRVRKALSYLFDFESINRLQLSGLRTRTDSYFEGG
ELQS----SGIPEGRELSILQEFS----DQLPE------ALFTTPFENPVGGDR-AKN----RDNQRLAAQL
MREAGWEPRN---GTWTN
IETG--EPFSIEILGG--S-PVTNIIAGQFTQDMRKFGIDASIRIV-DASQYVSRLE---DFNFDMT-TVQL
AQ----SLSP-GNEQRDFWSSTAADKPDSRNMSGIKNPVVDALVDKIIFAENRDE-LIALTHALD-RVLLWN
YYYIPQWHS---------PEEWLAWWD-KFDFVD--KQPTYSGVD--TSSWW--------------------
----
>Zongyanglinia/1-611 huanghaiensis
-------------VMVMGLILALGFASFLQAEEKIITSHGFSEFG---DLKYPAGFAHFDYVNPDAPRGGQL
SYGAQG--------TFDSFNPYTR-------KGRGAARSTEH------------FESLLTPS---------Y
DEAASYYGLVAESLEYPESQ----DWVIFNMRPEAVFSDGSPVTADDVVFSHNLLLDQG-LKSYADAVRKRI
PKAEALGPHRVKFYFSPDFPKRAMISQVG-GTPIYSKAWFEADP---ENRRLDEPRLD-PGIGSGPYMLDSY
DINRRVVYKRNPDYWGDNINVNVGRHNYDSIRIEYFTDSVAAMEGFK-SGAFTLRQEN--NSKSWATAYD-F
PAMHAGNVVKGDFFD--GDVPGASGFVFNLDRPQFQDIQVRAAMQLAYNFEWTNESLQYGLFQQRHSFWQGS
-ELQAT---GVPEGRELEVLQSLG----DALDPA------ILTEEVITAHESSS-KRQN--DRRNIRKAMKL
LDAAGWAVG---DDGI
RRNADGQTLSVEFLSDQ---PTLDRIIQPYVDNLVVMGVDAKYNRI-DNAQFTARRRDR---DFDMMVA---
--GYRNSLQPSTG-LYQKFGEEAASY-SLFNPTGVHGPDIEPLIDNIVQAKTTEE-LHANVRALD-RVLLSK
RIMIPTWYLG---------KYWVAYWD-MYSFP--DNLPPYAL--GFDDLWWIDEDRAAALVTSG-------
----
>Seohaeicola/1-595 saemankumensis
----------------LVVLALAAMTVLARAEDKIITSHGFSEFG---DLKYPAGFAHFDYVNPDAPRGGEL
SYAAQG--------TFDSFNPYAK-------QGRAAAGSGDQ------------YESLLIPA---------Y
DEPASYYGLVAESLEYPESQ----DWVIFNMRPEARFSDGTPVTAEDVVFSHELLLEQG-LKSYAEAVRKRI
PKAEVLDTHRVKFHFAPDISRRALISQVG-GTPIFSKAWFMADP---ENRRLDEPRNE-PGIGSGPYVLDSY
DFNRRVVYLRNQDYWGDAINVNIGRNNYETMRIEYFTDSIAAMEAFK-AGEFTLRQEN--NSKNWATSYD-F
PAVAKGWVRLEELTD--GDVPSATGFIMNMDRPQFQDIRVREAIQLAFNFEWTNESLQYGLFQQRHSFWQGT
-HLEAT---GLPEGRELEVLQALG----DALDPA------VLTGEPVSAHSSNA-DRQS--DRRNIRRAMKL
LDEAGWKVG---DDGK
RRNAGGQTLKVEFLSDQ---PTLDRILLPFVDNMKRLGIDAKYERI-DPSQFTLRRRER---DFDMVYS---
--GYQTSLQPSTG-LYQQYGSEAAAY-SVFNPAGIHGPDIEPLIDNIVAATEADE-LQANVRALD-RVLRAK
RFLVPIWYLG---------KHWVAYWD-MYEYP--ENLPPYAL--GVEDLWW--------------------
----
>Cereibacter/1-595 sediminicola
------------------------------EDETIIRSHGISTFG---DLNYPADFTHLSYVNPDAPKGGEI
SEWAFG--------GFDSMNPYSV-------KGRAAALSSIM------------YESILTGT---------A
DEIGAAYCLLCETLEYPEDR----SWVIFNLRPEATFSDGTPVTAEDVVFSYETFLTKG-LTDFRTVFAQQV

ESAEALDTHRVRFTFKPGIPTRDLPQDVG-GLPVLSKAHYERE-----GLDLEEGGLK-PFLGSGPYVLDRM
NVGQTVVYRRNPDYWGNDLPINRGRGNFDRIRIEYYADYNAAFEGFK-GGSYTFRNEA--SSILWATGYD-F
PAVGAGHVTKVELPA--GAKATGQGWMMNLRRDKFQDPRVREALNLMFNFEWSNQTLFYGLYTRVDSFWENS
-YLEAE---GPPSEAEVALMRPLVD--EGLLPES------ILTEPPVSPAGS-G-DRQL--DRRNLRAASKL
LDDAGWAVG---ADGL
RRNAGGEVLRIEFLNDS---QTFDRVINPFVENLRALGIDALMTRV-DNAQMESRTRPP-SYDFDITTG---
--NARTNYISGSE-LKQYYGSETADV-SSFNMMGLKSPAVDRMIEMVLAAETSDE-LEVATKALD-RVLRLQ
RFWVPQWYKA---------SHTVAYYD-MYEHP--EELPPYAL--GELDFWWFNPDKAEALRAAG-------
----
>Rhodoligotrophos/1-572 appendicifer
---------------------------------AAAPAHGIAMHG---DLRYPADFRHFDYVNPDAPKGGRL
NLSAIG--------TFDSLNPFIIRG-------LAPTGLR-----------DYVFESLLTRSRDE-------
--AFSLYGLLAESVELAENG----RSVTFSLNPSATFSDGTPVTIDDIEFSWAVQREKG-RPN-SRIYYRKV
VAIERPAPGAITFVFEDATDR-ELPLILG-LMPILPKHRYG-------EGRFDAATLE-APVGSGPYATEAV
KPGAEITYRRREDYWGKDLPVNRGRYNFDAIRVLYFRDSNSAFESFK-KGLVDVQADS--DPTRWTTG-YDF
PAARRGEVVKEAFET--QLPAPTAAFVFNTRRPPFSDIRVREALLELFDFSWINKNLYYDLFERTDSFYSGS
ELSSA---DRPASPEERVLLAEYP----DAVLP------AIMDGRYRLPGGSIT-GRD----RDSQKRALEL
LSQAGYGIDRG---SLRR
IDSG--EPLSFELLTVT---RDQERLALAYSRSLAKIGVTATVRQV-DSSQFQKRIE---TYDFDMMPYTWY
N-----SLSP-GNEQAFYWGSFGADHPGTRNYMGAKAPAIDPLIGAMIAARTRAE-LVTATRALD-RVLRSG
IYVVPLFYAP---------AQWVGRWT-PIEHPDVT-PLYGYQ----IDSWW--------------------
----
>Deefgea/1-573 tanakiae
-------------------------------------SHAIALGA---EPKYPADFTAFAYVNPQAKKGGEI
VLPNPDRR-----TSFDSFNPFII-------KGTSAAGLGS-----------LMFESLGTMN---------Q
DETATMYGLLAKDIAVAADG----LSVTFKLNPKARFNNGDPVTAADVKHSFTTLNSK-LASPQYASQLIDL
IGVDVIDTQTIRFRFK--TPNRELPQIVG-TLPIFSKKWGAG-------LALDKIALQ-KPIASGPYQISDY
KLGKQISFQRRDDYWAKSHPARIGQYNFDRITYRYYQDDTAKLEAFK-AGDFDFISEY--SARNWARQY-QG
PKFRSGEIIKRELRH--HNSSGMQGFVMNLRRPLFADIRVRQALGLALDFEWMNRQLFYGQYQRLNSFYANG
ELAAQ----GKPSDAELVLLNPLK----KQLPAS------VFG-ELPAQPNTNP-PHS---LRDNLRQAREL
LKQAGWEYR--DGALR
NQKG--EPFEFEMLDDG---GAMGRIFPSKARNLAKLGITMNVRNI-DFALYQRRLE---AFDFDVIVLR-F
PD----VQSP-GQELLDYYGSKAADVKGSSNVIGLKDPAIDALIEHVLKAKDRQQ-QIIAVHALD-RALLAG
HYIIPHWYSA---------NHLVAWRD-RFEMPKTMPLYY-QAGDWMLATWW--------------------
----
>Dinoroseobacter/1-583 shibae
-------------------------------------SHGISTFG---DLKYPADFPHLDYVTPDAPKGGEM
SVWAFG--------SFDSMNPYTL-------KGRAAGLSNVF------------FESLLTGT---------A
DEIGAAYCLLCESLTYPEDR----SEVTFTLREGITFSDGTPLTAQDVVFSYEILLTKG-LPSFRAQLAQKV
ESAEALDDRTVRFVFKEGIPTRDLISDVG-ALPVFSEDFYTAN-----DRDFEETSLE-PLVGSGPYVLGRM
DVGQTVVYERNPAYWGADLPINIGRNNFDAIRIEYYADYNAAFEGFK-GGTYTFRNEA--SSKIWATGYD-F
PAVAEGSVIKAELPN--GSKATGQAWVFNLRRPQFQDPRVREALGMMFNFEWSNETLFYGIYDYVDSFWDNS
-ELEAT---GLPTEAEIAILQPLVD--EGLLAPE------ILTSEPFSFPRS-G-ARQL--DRGNLRAASAL
LDEAGWVVG---DDGL
RR-KNGQTLSVEFLNDS---QTFDRVINPYVENLKRLGVDARHTRI-DNAQMTLRERPP-EYDFDMVTT---
--FMATDYIPGSG-LRQYFGSETADT-STFNKMGLQSEAIDRLIEVVLAAQSQEE-LTTAVHALD-RALRAE
KFWVPQWNKN---------THTVAYYD-MYEHP--ETLPPFAL--GNLDFWWFNAEKAEAL-----------
----
>Bauldia/1-599 litoralis
------------LRLAAAVAASFGIGLVP-QLAAAEASHGVAMIG---EPALPADFTHLPYANPDAPKGGKI
TYGVVG--------TYDSLNSFIVQGG-----TTSSRGLR------DPVFGNLVFESLLSRSSDE-------
--AFTLYGMLAESVETPEDR----SWVEFTMNPDARFSDGEPVTVDDVIFSMEILRDKG-RPN-YRYYYGKI
DDYERVGERGIRFNIATAKDR-ELPLILG-LMPILPKHAID-------PETFDKSTLT-PPVGSGPYVITDV
GAPNYIVFKRNPDYWASDLPIQKGLYNYDEIRIDYYRDANAMFEAFK-KGLYQVNPEG--DPAQWNKG-YDF
PALNQGRVVKETFST--GTPKGMSGFVFNTRRPIFADIAVRRALAKLFDFGWVNTNLYYDAFDRAAGYFNDS
ELSSI---GIPASDREKELLAPFP----DAVVA------EVMDGTYRPASSDSS-GAD----RTVLREALSE
LQAAGYELKDG---TLVN
TASG--KPLAFEMLVTT---KEDERLALAYQRTLSRIGIKASIRNV-DASQYQLRRQ---TFDFDMIRNSWG

-----ASLSP-GNEQSFRWSQEAADQDGSFNYPGASEPAIDAMIEAMLAARTRDE-FVAAVRALD-RVLVSG
FYVVPLFYLP---------DQWMARWT-TVEHPEQT-SINGAK----LETWW--------------------
----
>Jeongeupia/1-590 chitinilytica
---------------------LVAAILLLCVTLPARADYAVALGY---VPKYQTGFTHFDYVNPKAPTGGEL
RLAAMG--------DFDKFNPFTL-------KGRSVAGLGGLG----DG---LVFESLTMQS---------Q
DEPLSAYGLLADDIRIAPDG----LSVTFHLNPQARFSNGRKVGADDVRFSFETLTSK-RASPMFRQYWADV
AGVDVLDAGTVRFRFR--QHNRELPLTIG-QLPVFAKEWGGG-------KPFDEWVQQ-TPIGSGPYAIESV
DLGKRIVYKRRADYWGWKLPVRLGTFNFDRISYRYYQDDTARLEAFK-AGEFDFTFEN--VARQWVRGY-TG
PAFRDGRIVKQTFAH--ENSAGMQGYAINARRPNYADVRVRRALVLAMDFEWMNRQLFYDQYTRSPSYFSNS
EIEAR----GKPGPGELHYLEPLR----GRIDPS------VFV-TATMPPTTTP-PDS---LRKNLLRARQL
LEDAGWTYR--DGALR
NRKG--EPFVIDMLLYQ---KAFERLVAPYARNLQKLGITLNYRVL-DLALSQKRLD---AFDYDMTVVT-F
GA----SQSP-GNELYGNFGSHSANEPGSNNAMGISDPAVDALIDAVVQAPDRAA-LVDAGRALD-RVLRAG
YYLVPNWHNR---------VHRVAFRA-GYAWPATLPKYY-GAEEWAIQTWW--------------------
----
>Hartmannibacter/1-573 diazotrophicus
---------------------------------------GIAMHG---DPALAAGFDHLPYADPKAPKGGRI
TYGFQG--------TFDSLNPFIVKG-------NAPRGIF------DSIIGNNVVESLMTRSADE-------
--PFTLYGLLAKTVEVPEDR----SWIEFHLDPLARFSDGKPVTIDDVLYSVDLLRDKG-RPY-YRSRFEKI
AKAEKVGADGVRFTFVEGTDR-ELPMLIA-LLPVLPKHATD-------PQTFEKSSMD-QLVGSGPYTIGEV
RPGSEIRLERNPDYWAKDLPVKRGFDNFDEIRVDYFRDENALFEAFK-GGDVDIRFDN--DPNSWARA-YDF
PAATDGRIVKDAVET--GVPKGMNAFVFNTRRKPFDDERVRKALILLFDFEWVNHNLYSDSYTRTASFFQGS
SLSAF---GTPASEAEKALLAPFP----DAVEP------DVMDGSYAPPKSDGS-GRD----RKNLRAALDL
LKSAGFALKGR---DLVD
SATG--QPFRFEFMAMS---KEQERLALAYQSTLRLVGIDMSVRTV-DPVQFWDRQK---TYDFDMMQMLWT
-----GTLSP-GREQLFRWSSEAAKTDGTFNTAGVSNPAVDAMIAAFLQAVGQDD-FENAVRAFD-RTLMSG
HYVLPLFHLK---------DDRIAHWA-RIKRPDVT-ALTGFQ----TPTWWFD------------------
----
>Noviherbaspirillum/1-594 aerium
--------------MRKLLLSILLGITIPY-SSGSHAAHAFSLYD---TPKYPEGFKNFDYVNPDAPRGGEL
YLANPDRR-----TSFDKFNPYSM-------KGVAAAGVSN-----------LMFETLATSS---------A
DEMATMYGLLAEDMQLAEDR----MSMTFRLNPKAKFNNGDPVTAADVKHSYDTLLA--KGAPQFKSVFADV
KECVVVDERTVRFDFK--ALNRELPLIVG-GVPVFSRKWGAK-------TSFDKIQLE-APIATGPYLIERY
DVGRSIIYKRDPNYWGNDVPARRGMFNFGRIIYRFYKDDVARLEAFK-AGEFDVVVEY--SAKNWARSY-IG
PKFTSGEIVKRELQH--SNAAGMQGFVMNLRRGQFSDIRVRQALGLALDYEWMNRQLFYNQYKRIYSYFNNS
ELAAS----GMPSEDELKLLEPLR----DMLDPK------VFG-PAPVQPRTDP-PAS---LRANLLQAREL
FRQAGWEYR--DGALR
NAKG--EPFTFEILDDQ---SALSRIISVYIRNLQKLGIQVNQRTA-DYALVQKRME---EFDFDMTSIR-F
PD----TTSP-GNEMFDTFGSKAADEKGSNNAWGLKDPAVDKLVANLVAAGSRTE-LIAAARALD-RVLLHK
YIVVPHWFSS---------THRVAYRS-RFGIPAKMPLYY-AADPYVISSWW--------------------
----
>Tabrizicola/1-599 rongguiensis
--------------------------TGGATAQTIITSHGISTFG---ELKYPADFKHLDYVNPDAPKGGEI
SEWAFG--------GFDSMNPYSV-------KGRAAALSSSL------------YESILVSP---------S
DEIGSSYCLLCETMEYPEDR----SWVIFNLRPDVRFSDGTPMTADDVVFTFQTFLQKG-LTDFRTIFAQQI
EGAEVLGPHRVKFTFKTGVPTRDLPADVG-GLPILSKADYVAN-----KRDLEESSLQ-PFLGTGPYILDSM
DVGKTITYRRNPNYWGEKQPFNVGQNNFDRIRIEYYADYNSAFEGFK-AGNYTFRNEA--SSIQWATGYD-F
PAVQSGDVIKVELPD--GNKANGQAFLFNLRRAKFQDPKVREAIGLMFNFEWSNATLFYDLYARINSVWENS
-WLAAE---GAPSPEEVAILKPLVD--QKLLPES------ILTQPAVMAPVS-G-ARQL--DRENLRKASDL
LDDAGWLVG---PDGM
RRNEKGETLAVEILNDS---QTFDRVINPFVENLRKLGVDAKMTHV-DDAQMESRTRPP-VYDFDLIVG---
--NARSNYISGSE-LKQYYGSQTADV-SSFNVMGLKDKAVDQLIDVVLAAKTNDE-LTVATKALD-RVLRNI
RFWIPQWYKD---------KHTVAYYN-MYEHP--EKLPPYAL--GELSIWWYNPEKAAALKASG-------
----
>Tardiphaga/1-581 robiniae
-------------------------------AQAPSEAHGISAFG---DLKYPADFPNFDYVNVKAPKGGAF
STVPSSRAFNQSFQTFNSLNAYVL-------KGDGAQGMGMT------------FAPLMVRA---------G

DEPDAMYGLVAKTVQISADG----LTYRFTLRPEARFHDGSKLTARDAAFSLTALKTKG-HP-IITQQMRDM
VKAEAVDDVTLVVTFAEKRGRDVPLFVAG--MPIFSQSYYDKR-------AFDETSLE-TPLGSGPYKVGRF
EVGRYIEFDRVKDWWAADLPVNRGSYNFDTVRFDFYRDRDVAFEGFT-GRNYLYREEF--TSRIWNTRYD-F
PAVKDGRVKQEMLPD--DTPSGAQGWFLNTRRAKFKDPRVREALGNAFDFEWTNKTIMYGAYVRTVSPFQNS
-DLMAT---GAPSPEELALLEPF----RDKVPAE------VFGAPYLPPTSDGS---GQ--DRALLRKAIQL
LNDAGCA-M---KDGK
RMTAQGEPFKIEFLLDE---PAFQAHHMPYIKNLGTLGIEASVRLV-DPVQFRARRDD---FDFDAAIE---
--RFSFSTIPGDS-LRSFFSMQSAATKGSNNLAGISDPVVDALMNEVIAADTRPK-LVFAARALD-RVIRAG
RYWVPQWYAA---------SHRVAYWD-VFGHP--ANLPKYIG-VGAPDLWW--------------------
----
>Extensimonas/1-592 vulgaris
-------------------MRFCLLFVLWMCAVPAWAAHAYALWG---QPRYPAGFAHFDYVNPAAPKGGEL
RLVSNSRA-----STFDKYNPFTI-------KGSAPAYLSE-----------LMFDTLLVGA---------L
DETATGYGLLAQDVDVAPDG----LSATFRLRPEARFHNGKPVLAQDVKYSFDTLVGP-HTSPAYKTLLIDV
AGAEVLDERTLRYHFK--KPNRELPLIVG-GLPVFSRDWGAG-------KPFDQVVMD-TPIGSGPYRIGPV
RFGKDITYVRDPNYWARDLNVRRGTANFDRIQVKIYKDNTARLEALK-AGEFDLMRFF--SAADWARRV-NG
KRFATGELVKGEFPH--KLPSGFQSYVLNTRRPLLQDRRVREALGLALDFEWMNRQLFYGAYQRVRGLFGNT
ACETH----GLPSAPELALMEPWR----DAIPPA------AFG-PMYEPPRTDD-GHS---LRANLRRAQAL
LQEAGWVVQ--GGVLR
NAAG--QALELEYLDSG---EGSMRVVAPWIRNLEKLGITLRFRAV-DFALYQQRLQ---KFDFDITSLA-F
QG----THNP-GQEFADLFGSKAADTEDSGNFAGVKNPAVDAMIEAMVAAKTQAE-LLPACHALD-RIIMHS
HYLIPQWSAS---------THRMAYNAWRLARPAQVPPYAPG-ETWVIDTWW--------------------
----
>Ascidiaceihabitans/1-612 donghaensis
-------------------LSLIVLAASMVRAQDTVKTHGYSFYG---DLTYPEDYPHFDYVNPDAPKGGEI
SIGTLG--------TFDSMHPYTR-------KGRAGALSSVM------------YESLLGAG---TNADAPA
DVYAEYYGLLAKGLEYDEGR----NWVIFYMRPEARFSDGSPVTAHDIAFSHNLLLDEG-LKSYADAVRKRI
PKVEVIDDHTIKFYFTEGISRRSLIDQVG-FVPAWSKKWYEET-----GASLNESRLE-VSPGSGPYMIEDV
DVNRRVVYKRNPDYWGNDLPFNVGRNNFDRIRIEYFGDDTARFEGFK-AGEFTMRIES--DSKRWATGYD-F
SKIDDGHIVKKELPD--GAPATPSGIVFNLGREVMQDRRVRQALALAYNFEWTNESLQYGLFQQRASFSQDT
-EIMAK---GTPEGAELAFLQSLG----DLVPAD------MLSEPVAIPHTSKA-GRLL--DRRNARTAMRL
LDDAGWAVG---DDGM
RRNADGQPLSVNFLFNSSSPSTLSAAMENYVNNVRKLGIDITFEKV-DSAQYTSRERDR---DYDLVYD---
--SYAAFLGAGTG-LMQRYGSETAAF-SLFNPAGLASPLVDAIIEASLQSETREE-EVAVLRALD-RVLRHE
FFMIPLWYKA---------NHWVSYYN-MFEHP--ENMPAFDL--GYLDFWWYNADKASALKAAG-------
----
>Jeongeupia/1-576 naejangsanensis
-----------------------------------RADYAVALGY---APKYQTGFTHFDYVNPKAPTGGEL
RLAAMG--------DFDKLNPFTL-------KGRSVAGLGGLG----DG---LVFESLTMQS---------Q
DEPLSAYGLLADDIRIAPDG----LSVTFHLNPKARFSNGRKVGADDVRFSFETLTSK-RASPMFRQYWGDV
AGVDVLDAETVRFRFK--QRNRELPLTIG-QLPVFAKEWGGG-------KPFDEWVQQ-TPIGSGPYAIESV
DLGKRVVYKRRADYWGWKLPVRLGTFNFDRISYRYYQDDTARLEAFK-AGEFDFTFEN--VARQWVRGY-TG
PAFRDGRIVKQTFAH--ENSAGMQGYAINARRPSYTDVRVRRALVLAMDFEWMNRQLFYDQYTRSPSYFSNS
EIEAK----GKPGPGELHYLEPLR----GRIDPS------VFV-TATMPPTTTP-PDS---LRKNLLRARQL
LEDAGWTYR--DGALR
NKKG--EPFVIDMLLYQ---KAFERLVAPYARNLQKLGITLNYRVL-DLALAQKRLD---AFDYDMTVVT-F
GA----SQSP-GNELYGNFGSRSANEPGSNNAMGISDPAVDALIDAVVQAPDRAA-LVDAGRALD-RVLRAG
YYLVPNWHNR---------VHRVAFRA-GYAWPATLPKYY-GAEEWAIQTWW--------------------
----
>Chitinibacter/1-589 fontanus
---------------------KLLASFLCIYHAHVWAAHAISLGD---TVKYPQGFTAFAYVNPQAKKAGEL
ILPNPDRR-----SSFDSFNPFII-------KGQPAAGLGA-----------LMFESLGTMS---------L
DETATMYGLLASDIALAADG----KAVTFKLNPKARFNNGDPVTAQDVKHSFDTLNSK-LAAPQYASQLSDV
AGIEILDAGTVRYRFK--SANRELPQIVG-TLPVFSHKWGAG-------KALDQIALE-APITSGPYRIASY
QFGRSISYQRRLDYWGSTIPTRRGQYNFDRVTYRYYQDETARLEAFK-AGEFDFITEY--SARNWARQY-QG
PKFRAGEIIKRELRH--HNSSGMQGYVLNIRKPQFADLRVRQALGLALDFEWLNRQLFYSQYTRLNSFYANG
ELAAS----GKPSEAELALLKPLA----KFLPPE------VFG-QLPAQPNTNP-PNS---LRNNLRQAREL
LKQAGWEYR--DGALR

NAKG--EAFTFEILDDG---GSMARVFPPLARNLAKLGITATMRNI-DFALYQRRLD---SFDFDVTVLR-F
PD----VQSP-GQELLDYYGSKAADVKGSSNVIGIKNPAIDALIGHVLAAPNRAA-QITAVHALD-RALLTG
HYIIPHWYSA---------THRVAWRD-RFAMPKVMPLYY-QAGDWMLATWW--------------------
----
>Afipia/1-585 massiliensis
------------------------------------ETHGMSAFG---DLKYPADFKNFDYVNVGAPKGGVF
STVPSSRAYNQSFHTFNSLNSFIL-------RGDGAVGMGQT------------FASLMVRS---------G
DEPDAMYGLVARSVRISPDK----LTYRFTLRPEARFHDGTKITAKDVAFSLTILKEKG-HP-IIQQQLRDM
TKVEANDDATLTVTFAAQRGRDVPLYTAG--LPIFSKAYYDKK-------PFEESTLD-IPLGSGPYKVGRF
EVGRYIEYDRVKDWWAKDLPVSRGAFNFDAVRYEFYRDRDVAFEGFT-GRNYLYREEF--TARIWSTRYD-F
PAIKDGRVKQEKLPD--DTPSGAQGWFLNTRRAQFKDPRVREAMIQAFDFEWTNKTIMYSAYARTQSPFQNS
-DLMAE---GPPSPEELKLLEPF----RGQVPDE------VFGAPFVPPVSDGS---GQ--DRSLLRKASQL
LQDAGYP-I---KDGK
RRLPNGDAFRVEFLLEE---PAFQAHHAPYIKNLGVLGIDASVRLV-DPVQFRSRVDD---FDFDVAIQ---
--RFSMSSTPGDA-MRTFFSSQVADIKGSQNLSGIKNPAIDALIERIIAADNRDE-LRIACRAFD-RVFRAG
RYWVPQWYSA---------SHRIAYWD-VFAHP--PNIPRYANSAGAPDLWWFDEAKAKK------------
----
>Ensifer/1-581 sesbaniae
--------------------------------------HGLSLVG---ELKYKPNFPHYDYVNPNAPKGGDV
RLSNTG--------TFDTFNPILD-------RGEVAVGLA------------LVFDTLMKSADDE-------
--ISTAYGLLAEGVSFPADI----SSATFRLRQEAKWADGKPVTPEDVIFSFDKTKEL---NPLYSNYYKHV
KSAEKTGERDVTFHFDEKN-NHELPHILG-QFLILPKHWWEGQGPDGKPRDINKTTLE-PVMGSGPYKIASF
SAGATIRYELRDDYWGKSLNVNVGHNNFGSISYTYFGDRDVEFEAFR-SGNSDYWQEN--QASRWATGY-DF
PAVKDGRIKRESLPNPLRATGIMQALVPNMRRVPFNDERVRQALNYAFDFEEMNRTVFYNQYTRVDSYFWNT
ELAS----SGLPEGDELSILNEVK----DKVPP------EVFTTPYKNPVGGTP-QAG----RDNLRQAIAL
LMEAGYELK---GNRMVE
AKSG--RPFSFEILLS--S-ATLERVALPYAQNLKKIGIDARVRTV-DPSQYTNRTR---AFDYDMTWIV-W
GE----TLSP-GNEQLDQWGSRAAKEPGSRNYAGIADAGVDALIEKVIFAKDRKA-LVAATKALD-RVLLAH
NFVVPLYYK---------KDMTIAYWD-KFERPA--ELPTYGIG--FPDIWWSKTAAAK-------------
----
>Tropicibacter/1-595 naphthalenivorans
-----------------------------AQDENVTTAHGYSYFG---NLDYPADYTHFNFVNPDAPKGGAL
VLGATG--------TFDSLNPYTV-------KGRSGALTTLQ------------YDSLIESV---------E
DSVGQYYCLLCESLEYPESR----DWVIFHMRQDAKFWDGSPVTAHDVVYSHKLFTTQG-LPSYAAAVSKMI
TGAEALDDYTVKFTFNPEETKRSRIETAG-STPVFQKAWFEADP---ENRRLDQPRLE-VAPGSGPYRLDSF
EVNRRIVYKRVEDYWGADLPYQTGRNNFDEIRLEYFADQTAAFEAFK-AGEYTFTTES--DPKQWATGYD-F
PKVQNGIVVKEELPD--GSPPNPTGFVFNLGKEPLQDKRVREALALAFNFEWTNESLLFGLYSPRSSFVQGT
-HIEAT---DVPTGAELAFLQSLG----DVVPSD------VYDTPVYQFHESNA-SRLP--DRRNLRQASNL
LSNAGWEVG---DDGV
RRNAQGNTLSLTFLIPSNIDSSVEGMHETFIQNLQQIGVDASVEKV-DPSQYTNRRRDR---DYDVLYST--
--RYGTFLSTGGG-LSQMYGSEEAAF-SVFNPAGLASPLVDAIIAESFNAENQDE-TDVALKALD-RALRYE
FIMIPTGYIA---------DHWVAYYD-MYEHP--ETIPPYDL--GYLSFWWANPEKQANL-----------
----
>Cohaesibacter/1-622 gelatinilyticus
--------------------------IPAQAVEFDTWLHGTSLMG---DVKYPKGFKHFDYVNPDAPKGGIA
RQAVAG--------GFDNFNAIIP-------KGDPAAGLG------------NIYDTLMASSYDE-------
--ISTEYGLVAEEMLIGPNY----SYVKFRLRPEARWHDGKPITPEDVIWSFEKLTSL---SPQQRFYYSHV
VGAEITGENEVTFRFDEEG-NRELPHIVG-QLLILPKHWWTGTDAKGEKRDISRVTLE-PPLGSGAYKIGEF
SPGKFMILDRVDDYWGKDLAINIGSNNFEQLRYEYFRDNTVMFEAFK-SDAYDFRVEN--TARVWAKSY-DF
PAIEDGRVIKEEFPD--RASGVTIGFFPNMRREKFKDPRVRQALNYLFNFEEMNRTLFFNQYKRAGSYFFGT
EFAA----SGKPDAAELALLEPLK----DQVPA------IVFEELPDQPKVESR-EDI----RSNLKKALEL
FKEAGWEPKTEIDKSKMPTGLAGFWHSITSTLGLSS----------------------------DPTRSVMR
NEKG--EAFEIEYLVS--T-PSFERIALRLQASMERVGIKMTIRVV-DSAQYVNRLR---NRDYDFIYGG-F
AQ----SLSL-GNEQKGYFGSASADREGSRNFGGIKNPAVDKLVDAIIFADNRKS-LVTAARALD-RVLQAN
HYMVPGWTN---------DMTRTARWN-RFGHPK--DLPILTVG--FPTIWWWDEAKAKSI-----------
----
>Rubrimonas/1-572 cliftonensis
--------------------------------EGVIRTHGGSLVG---KLKYPEGFAHFDYVNPDAPKGGVA

RQATAS--------AFDSFNPFIV-------RGDPPTGIG------------VVFESLMTPSLDE-------
--GSTHYGLLAEWEEHPADD----AWVSFRLREEARWHDGRPITVDDVIWSFETLVARG--APLYRFYYKNV
VSARDLGDRVVRFDFDQTG-NRELPHIMG-QLTVLPKHWWE------DGRDFEAGGLE-PPLGSGPYRVGAF
ESGRFVEYERVPDYWGAGLPVNVGANNFDRLRYEIFLDASAQLDAFR-AGQIDYREEF--SAQNWAERY-DF
AAVRDGRVKRDEVTL--DGLKRVQFFAMNLRRPKFQHIDTRRALSIAFDFEWTNKTIYFDQYARPSSYFQGG
DDLQP---SAPPEGLELAMLEPFR----GQVPD------EVFGAPYAPPQTDGS-GRN----RRGLRDAARL
LESAGWRPGP---DGVLQ
DAQG--RPFEIEFLSA--Q-DTQGRVIGPYIDNLATLGIRATFRVV-DGPQYVRRVSQDPDFDFDMIIFG-V
NN----SESP-GNEQREYWGSEAADRMGSRNVSGVKDPAVDALIERIVFAEDRET-LAAASRALD-RVLTHS
RYSVLQLYT---------PFERLAWWD-VFGRPE--PLPSRSLG------F---------------------
----
>Rhodoferax/1-592 sediminis
-------------------MRGLLFCLMLLSATASWASHALSIWG---DVQYRAGFPYFDDVNPDAPKGGEM
VLVSDQRS-----STFDKYNPFSL-------RGSAPAYIDT-----------LMFDTLLTGS---------P
SEFGVGYCLLAQDVELAPDR----MSVVFKLNPSARFNNGDPVTAADVKYSFDSLTGK-YAAPQYRSLFADV
KSPQVLDAGTIRFEFK--RFNRELPLTVG-GLPVFSHKWGAG-------KPFDQITTE-PPIASGPYKIGPV
VFGRDITYVRDPTYWARELNVRRGQYNFDRITVKIYRDNTAKLEAFK-AGEFDLIQEF--ISRDWVRAY-TG
KRFDSGELVKRLLPD--NSPHGFQVHVFNLRRPKFQDIRVRKAINLAFDFEWMNRQLFYGLYNRLKGYFGGS
EYEAL----GTPGADELALLEPFK----NQLPPA------VFG-PVAYPPSTDP-PRS---LRGNLREAKAL
LHEAGWDYR--DGALR
NAQG--EAFVIDYVDSN---ESGIRTIAPWMRNLEKLGIQLNFRAV-DFSIYQQRLR---TFDFDMTGIR-M
AG----ITVP-GNEMFDLFGSRAAKIEESNNLWGLQSPVVDALLEKMVTAQRRSD-ADAAGKALD-RVLIHG
WYSVPQWYSA---------SYRVAYDSRKFVQPSRLPKYFTP-EAWAMSTWW--------------------
----
>Primorskyibacter/1-608 aestuariivivens
---------------LVGGLVLAAGLASAAIGEEVTESHGYSYFG---NLDYPADFPHFDFVNPDAPKGGEI
SLGASG--------TFDSLNPYSR-------KGRSGVLTGVQ------------YDQLIETA---------E
DTVGQYYGILAHKLEYPESR----DWVIFHMRPEARFWDGTQVTAHDVVYSHKLFITQG-LPSYAQAVSRMV
TSAEALDDHTVKFTFNPEESKRSRIETVG-STPVFSKAWFEAD----ESRRLDEPRLE-VAVGSGPYKLDSY
EVNRQIIYKRRDDYWGEDLPYNKGRYNYDTIRVEYFADQTASFEAFK-AGEYTIRIES--DPKMWASSYD-F
PKVNDGTVVQMELPD--GSPPNPTGFVFNLATPQLQDKSVREAIALAFNFEWTNESLLFGLYSQRSSFVEGT
-HLEAK---DVPTGKELEFLKSLG----DVVPPE------IFTDPVRMSHTSGA-ERLN--DRRNLRQAAKM
LEEAGWVVG---DDGV
RRNAKGEELTLDMLFPSNIQSSVEAMHETFAQNLALIGVKANLEKV-DPSQYSERRRER---QFDILYST--
--RYSTFLSTGGG-LSQMYGSKEAAF-SLFNPAGLASPLVDAIIEASFVTDNQVD-QDTALMALD-RALRHA
FFIVPTGYIA---------EHWVAAYD-MYGHP--ETLPPFAL--GYLDFWWIDPDREAAL-----------
----
>Fuscibacter/1-594 oryzae
--------------------------------QKIITAHGISTFG---PLKYPADFKHLDYVNPDAPKGGEI
SEAELG--------TFDSLNPYSF-------TGTATGVATVL------------YESILGAT---------A
DEVGAAYCLMCETMEYPEDR----SWVIFNLRKDVKFSDGTPLTADDVLFSYNLFREKG-IAEFRAVFNDKV
QSAEVLDPYRIKFTFTPGTPFRDMPASVG-SLTVFSKADYEKN-----NRDLEASSLK-PFLGTGPYVLDSL
KPGQQIILKRNPDYWAANHPLNIGQNNFDRIRVEFFGDDSAAFEGFK-SGVYTFRQET--TVKQWATGYD-F
PGVKNGTVIKAELPN--GNLPPASGFLFNLRREKFQDVRVREAIGLMFNFEWSNQTLFYGLYARVNSVWENS
-SMAAK---GAPGPDEVAILQPLVD--QGLLPAS------ILTDEAAMSPVSTP-ERSL--DRKNLRKASAL
LEQAGWVVG---DDGM
RRNAKGETLKVEFLTAS---PQLERVISPYVENLRAAGVDASLNII-DPAQLQFRADPP-NFDFDVLPT---
--SMVNSQEPGGE-MKQSYASVTANN-STRNRMGLANPAVDKLIDNVEPAKTREE-LDSATRALD-RVLRSL
HLMVPRWYNP---------NYFVAYFD-QYDHP--TTLPPYAL--GETSFWWYDAAKADKLRAAG-------
----
>Falsochrobactrum/1-581 ovis
--------------------------------------HGISAFG---NLKYPKDFGYFDYVNPDAPRGGVF
RFAPSSWLWNQNTNTFNTLNSFSF-------KGDSPPRLELT------------FDALMAAA---------F
DEPDSLYGLIAESVTISPDR----QTFLFKLRKAARFHDGSPVQASDIVFTYRIFQKKG-HP-HIRQSLSAL
ELIEE-DEGDVRMRFSEGQ-PKAVFDAVT--LPILSENYFDDK-------NFESTATE-PILGSGAYRVGNF
VLGQFIEYDRVDDYWAKDLPINRGQNNFDRIRIEFYRDRQPEFEAFK-KGAIDFRAEY--VAKNWATGYE-F
PAIKQNKVIKRTFAR--EKRPLMQAWALNQRREHFRDPRVREAIGLCFDFEWTNKNLFYGAYERSQSCFNGS
-DFQAV---DLPTAQEHKILERY----KDRLPPE------IYGPAPIMAVSDGS---GR--DRKQFSRAIQL

LKESGFI-R---EKGN
FHDETGQKLSLEILSNS---ESFSRIYNPFVQRLRAIGIDASLRLV-DASQYQSRVED---FDFDMVGM---
--AMQFGATPTKDSLAGMFGSKAANMPGSYNLPGTSDPIIDALIEDVGTVTTRDE-LVATIRVLD-RYLRIR
REWIPNWTVA---------NHRVAYWD-RFGFK--EPKPDYGF--PVETLWWLDKEKAETI-----------
----
>Oharaeibacter/1-593 diazotrophicus
---------------------------FGRALAAAGRRHGLSIFG---DLKYPADFTRFAYANPDAPKGGRL
PFTVPNWYFNQDPQGFDTLNGFVI-------KGSAAPRVELC------------FDALMVRA---------L
DEPDAVYGLLAEAVEVSEDG----TTYTFHLRPDARFHDGSALTAEDVAFSYNLLKAKG-HP-NLADGLREM
ANAEAIDAGIVELSFTGRQSLSAPLEATG--FPVFSKAWWDGR-------DFGAATLE-PILGSGPYKVGRF
DQHAFVEYERVPDWWAKDLPVSRGQYNFDVVRVEFYKERQAAFEAFK-KGETLLREEF--VSKTWATEYD-F
PAIRDGRVKREEIAA--EANPSMQGWFFNLRRGKFADPRTREAIGLAFDFEWTNKNLFFGAYARQTSFFQKS
-DFAAD---GPPSPAEAVLLESF----RADLPAG------VFGPAVSPPVADGS---GR--DRTLLRRAAEL
LAAAGWT-R---DGEV
LRDRAGAALTLEFLIES---TVYERVLTPYVRNLRSIGVDASVRLV-DSSQYQRRTQD---FDFDVIGR---
--AFSMSPTPIES-LDGFFHSRLANQPGSYNVSGIAVPAIDALIARAGQVKHRGE-LVDVVRAID-RVLRAL
HVWVPNWYSS---------THRVAVWD-VFGRP--AEKPAYGF--PVESTWWIDADKAAAL-----------
----
>Heliomarina/1-589 baculiformis
-------------------------------------SHGYSFYG---DLKYPADFEHYDYVNPDAPKGGEL
SYAALG--------TFDSMNPYTV-------KGRRGELSWIM------------YESLLGDG--------PA
DAYGEAYGLLAESLEYPATK----DWVIFHMRPEAKFSDGTPVTAEDVMFSHNLLLEQG-LPSYAAAVKKRI
PRVEVLDEHTIKFYFTPGISRRSLIDQVG-GVPVWSKKWFEES-----GARLDESRME-TSPGSGPYMIDRI
DVNRRIVYKRNPDYWGKDLAINQGRHNFDSIRVEYFGDDTASFEAFK-AGEYTFRVEG--DSKKWATSYN-F
PKIQAGQVVKAELPD--GTPPTPSGIVFNLRREVMQDKNLREALSLAFNFEWTNESLQYNLFEQRASFSQDT
-PLMAT---GAPEGAELEFLKSLG----DVVPAE------MLTEEARMPHSSDG-SRLT--DRRNLRRAMKL
LDEAGWPVG---SDGV
RRNADGKTLSLTLLFNTAAEGTLGAILENYVNNVQAMGIDITLEKV-DPAQYTLREREF---DYDLVFD---
--SYRPFLGTGTG-LMQMYGSSEAAF-SVFNPAGLASPMVDAIIEASLMAENAEE-EETSLRALD-RALRFE
FFMIPVWYKA---------DYWVSYYD-QYGYP--EPLPPYDL--GYLDFWWYDAEKAEALKTAG-------
----
>Futiania/1-565 mangrovii
--------------------------------------HAIAMHG---EPALPPDFAHFPYADPAAPKGGRL
VLGQLG--------GFDSLNPLIVKG-------QAAAGIR-----------EYVYESLLARSFDE-------
--PFTLYASVAKAVEVPDDR----GSITFHLNENAHFSDGEPLTAEDVLFTWALLKEKG-RPN-HRFYYGKV
AEATAPDAHTVRFVFEAGSDR-EMPLIMG-LMPILPAHATD-------AETFGETTLS-APVGSGPYTVAEV
DTNRSISFARDPDWWGRDLPANRGRFNFDEIRIDYYRDANALFEGFK-AGLVDLRIEQ--DPAQWASA-YDF
PAMRDGRMVREQIAH--GRPSGMLGLVFNTRREIFADPRARKALAGLFDFEWVNRTLYAGGYVRTRSYFDNT
PLAAK---DAPTP-KEQALLVPYA----ETLDP------DVMAEGFAPPDGSGP-GAL----RERARAAVGL
LQEAGFRFEGR---TLLT
PS-G--SPAQFEILLAE---PDWERLALAWADALKRIGIAVQVRTV-DSAQYQLRRQ---EYDFDMIVNDWG
-----MSLSP-GNEQSFYWSSQAAGTPGTRNYPGVRSEAVDAMIAALLAARTRED-FEAAVRALD-RALLAG
WYVLPLFHLD---------ADLVAHAG-RLERPETT-SLYGMM----LDTWW--------------------
----
>Starkeya/1-560 koreensis
--G---APQYPAGFAHYRSVNPDAPKGGRL
TQAAVG--------GFDSLNPFIVRG-------TAPPFIR-----------WNLVESLMARSPDE-------
--AFTCYGLLAQTVETDDAR----SYVAFNLDPRARFADGVPVTAEDVLFSFELLRSRG-RPN-HRSYYGKV
AKAEVTGPLSIRFTFG-VVDR-ELPLILA-LMPVLAKHAVN-------PETFEQTSFT-APLGSGPYAVAEV
KPGESVILNRRADYWGAALPVNRGNYNFDSLRYDFYRDVNSQFEAFK-RGLSDIRFET--DPGRWKTG-YDI
PAVRDGRIVTEQIAT--GTPKPYSALIFNMRRPLFADVRVRQAMVELFDFEWANENLYFGLYRRNGSFYDDS
ELSSL---GRPADARERALLSGFP----DAVLP------QVMDGTWRVPVADGS-GRD----RERLRRALDL
FGEAGWQLKGG---ALVS
KAGG--ERFAPELIVGT---KDQERLALAYQNMLRRAGVTLNIRLV-DNVQFEARKQ---TYDYDMVPYIWD
Q-----SLSP-GNEQAFYFGSAAADTPGTRNFMGLKSPAADAMIEALLAARERGD-FVSAVRALD-RVLISA
RFSLPLFYTP---------GQWVARWR-KVERPQTL-ALQGTL----AESWW--------------------
----
>Simplicispira/1-578 suum

-------------------------------------AHAYALWG---EPLYPAGFDHFAYVNPQAPKGGEL
RLVSNQRT-----STFDKYNPFTM-------RGAAPAYLSE-----------LMFDSLLTGS---------L
DETATGYGLLADDVQVAADG----LSATFHLHPEARFHNGDPVLAQDVAWTFETLVGP-FTSPGYKTLLIDV
AGVDVVNDRTVRFRFN--KPNRELPLTVG-GLPIFSRAWGMEN---GKHKPFDQVVTD-IPIGSGPYKIGPV
RFGKDITYVRDPNYWARDLKVRRGQNNFDRIEVKIYKDNTAKLEALK-AGEFDLMRFF--SAGDWARRV-NG
KKFDTGELVKGEFAH--KLPSGFQSYVLNTRRPFLQDERVREALGLAIDYEWMNRQLFYGAYQRVHGLFGNT
ACETH----GSPSPDELALMEPWR----KSIPPA------AFG-PMPQPPRTDG-EHS---LRDNLRRAKAL
LNDAGWAVK--DGALR
NAQG--EAMVLEYMDSN---EGGVRTVSPWMRNLEKLGITLKFRSV-DFALYQERLQ---KFDFDITSIA-Y
QG----TNNP-GQEFADLFGSKAADTEDSGNFAGVKNPAVDAMIRAMTSAKTQQQ-LLPACHALE-RIIVAG
HYLIPQWSAG---------THRMAYNAWRLAKPDVVPPYSPG-ETWVIDTWW--------------------
----
>Andreprevotia/1-592 chitinilytica
------------------MRRILVACLLMTFAHVALADHAIALGY---TPKYPAGFTHFDYVNPDAPKGGEL
LMANPDRR-----TSFDTFNPFIL-------KGTAPVGVGE-----------LMFESLATGS---------L
DEPATVYGLLADDMSVAADG----LSATFHINPKARFNNGDAVTAKDVKYSFDMLISK-QAAPQYAAMLADV
KAVTVLDRLMIRFTFK--RKNPELPQIVA-TLPVFSDKWVGN-------KPFDKLGLT-PPISSGPYRIEHF
DAGRSITFKRNANYWGRDLPTRRGMFNFDRMTWRFYKDEVVKLEAFK-AGEFDFNVES--IARQWMRQY-NG
PKFRSGELIKHEFMH--HNTAGFQGFILNTRRPQFADWRVRKALGLALDFDWLNKQLFYDLYVRMDSLYSNS
ELAAT----GLPTPGELKLLEPLR----KQLNPA------VFG-PVPMPPATQP-PDS---LRANLLQARDL
LAQAGWTYR--DGALR
NAKG--EPFSIEYLDDS---GSMMKVLAPWSRNLQKLGITVRPRTA-DFALYQKRLD---GFDFDVTTLH-Y
GD----TQTP-GNELYDLFGSKAAKTEGSSNVIGVADPAVDRLIDAIVASETRAE-RVTAVRALD-RVLRNG
YYVIPHWYSR---------THRIAYRN-TLRFPATLPQYY-PAAGWIVSTWW--------------------
----
>Delftia/1-578 tsuruhatensis
-------------------------------------SHAYALWG---EPRYAEGFSHFDYVNAKAPKGGEL
RLVSNLRI-----STFDKYNPFTI-------KGSSPAYLAE-----------LMFDTLLWPS---------L
DETATGYGLLAEDVQAAPDG----LSATFRLRPQARFHNGKPVLAADVKFSYDTLVGP-HALPGYRSLFFDV
AGVDVLDERTVRFRFK--QPNRELPLVVG-AIPVFSRDWGMEN---GKPKPFDQVVTD-TPIGSGPYRIGPV
RFGKDITYVRDPDYWGRDLPARVGTANFDRISVKIYRDNTAKLEALK-AGEFDLMRFY--SAGDWARRL-YG
KRFDRGELVKGEFSN--KLPSGFQSYVLNTRRPLLQDVRVRRALDLALDYEWMNRQMFYGSYQRVRGLFGNT
ACETH----GEPSPEELALMEPLR----KSIPPE------AFG-PMAEPPRTDG-NNS---LRANLRQARQL
LADAGWTIQ--DGVLR
NARG--QAMELEYLDSS---ESGIRQVSPWIRNLEKLGIRLRFRSA-DFALYQQRVE---KFDYDIISIA-Y
QG----TNNP-GQEFADLFGSRAADTDGSGNFPGVKNSAVDALIRRMTEAKTEAQ-LLPACHALE-RVIAHG
HYLIPQWSAG---------THRMVYDSWRLVRPAVVPAYAKG-ESWAMDTWW--------------------
----
>Abyssibius/1-583 alkaniclasticus
--------------------------FGTARAQDTTIAHAISKFG---DIRYPADFENFDYVNPDAPKGGTF
STWAYG--------SFDTLNPYSV-------NGVAASGANIM------------IERLMTGT---------Q
DEPDTVYGLLAETVEYPADH----SWAIFTLREGIRFSDGTPVTAADVVFSHNLFVTQG-IPDFAAAVKAGI
PNVEALDDRRVKFYFAEPSETTDWIQQAA-ATPVLKASQFEDR-------ELTDTTLE-PYIGTGAYMFDDM
DTGRWIQYRRNPNYWGADLPINAGQNNFDVIRYQYYSDPTAAFEGFK-AGEYLFRSEN--SSAAWANDYD-F
PALTNGWVIKTELPD--GDIGQAQGFAFNLYRPQFADPRVRQAIGMAFNFEWSNRTLFYGLYKRVDSFWENS
-VLEAQ---GDIPDDERAILEPLA----ADLPPE------VFSEPAYSPPVSSD--NQA--DRAVLRAAGRL
LDEAGWTVG---SDGK
RRNADGVVLEAEFLNYS---PLFDRIINPYIENLERLGVVATMRRV-DTAQFVERSRNR---DFDIVID---
--TFANSLTPGLG-TAQTYHSDNATG-ETRNTTGLANPAIDALVEMLPNAKTRDE-LNLITRALD-RALRAM
HIWVPQWYKD---------VHTVAFWD-TFGMP-DQALSPYAL--NPAGTWWWD------------------
----
>Gellertiella/1-598 hungarica
---------------FILSALLAAGGFSAVVAEDGGFVHGLSSSGE--PPKYQPGFPRFDYVNPDAPKGGAV
TQAVVG--------TFDTLNPLLA-------KGDAASGSD------------LVFETLLKNALDE-------
--VTTAYGLLAEGVSVPADI----SSATFRLRPEARFADGTPVTPEDVIFSFEKGKEL---NPQLAVYYAHV
VKAEKTGERDVTFRFDMTG-NKELPNILG-QFPVVPKHWWEAKDAKGNQRDISRTTLE-PVIGSGPYRIAEV
QPGASIRYERRDDYWGKNLNVNVGQNNFATMKYVYFGDNDVAFEAFR-SGQIDYWLEL--TALRWAKSY-DF
PAVQDGRVKREQVENPYRNAGVLVGFIPNLRRDLFKDQRVRKALNYAFDFEELNRTIFFGQYQRINSYFYGT

ELAA----KGLPEGRELEILNEVK----DLVPP------EVFTTPYENPVGGKP-DLF----RNNMKTAIGL
FREAGYEIR---GGKMVE
TKTG--TPVSLEILLD--G-KTLERVALPFAQNLKKIGIAASVRVV-DPAQYTNRVR---SFDFDVIYSG-W
AQ----SLHP-GNEQAEFWGSQAATREGSKNYGGISDPGIDQLVKKVIAAGTQDE-LKAATHALD-RVLLAH
DFVVPTYTL---------RASRIAYWS-DLTRPE--KLPEYGVG--FPDIWW--------------------
----
>Pseudopuniceibacterium/1-590 antarcticum
----------------------------------IITSHGYSYFG---ELKYPADFEHLAFVNPDAPKGGEI
VIQESG--------TFDSMNPYTV-------KGRAGAQATIM------------YETLLGSS---------A
DDYNGSYCLLCESLEYDEGQ----TWVIFHMRKNVKFSDGTPLTAQDVVFSHNLLIEQG-LPSYSAGVKKRI
LSAEALDDYTVKFTFATGISRRSLIDQVG-GTSVFSKKWYEET-----GARLDEPRFE-ISPGSGPYMLDSY
DVNRRIVYKRNPEYWGADLPLNKGRYNFDTIRIEYFGDESAAFEAFK-AGEYTFRIES--SSRQWATQYN-F
PGVQNGHVIKTELPD--GTPPNNVGFVFNLGQEKFKDKRVREAIALAYNFEWTNESLQYGLFKHRESYFQDT
-PLQAI---GLPEGEDLAFLDGLG----DIVPEV------LKTEPPVGIHDSSP-ERLN--DRGNLRKAANL
LEEAGWIVG---DDGL
RRNAAGDVLFIDFPINSSSASALEPVLDNYALNLKAIGIRPDFEKV-DPSQYTNRTRKG---QYDMVYG---
--GYPAFLGTGTG-LMQMYGTSEVGF-SSFNPAALASDLVDTIILASLDTTSQEE-QDASLRALD-RALRYE
FLMVPTWYKS---------GYWVAYYD-MFEHP--D-IPPYDL--GYLDFWWYNADKAAQLKAAG-------
----
>Serpentinimonas/1-583 raichei
-------------------------------------AHGYAQFG---ELALPPGFAHFPYVNPNAPKGGEI
TLVSPTRI-----TNFDKFNPFTL-------RGTAPPGLFG-----------LLFETLLTPS---------L
NEPAAAYGLLADDVQVAPDG----RSVTFRLHPQARFHNGDPVLAEDVRHSFETLIGP-QAAPQFRSFFSEV
TGVRVLGERLLRFDFA--RANPELPLVIG-SLPVFSRQWGVVD---GVRLPFDQIVMQ-HPIASGPYRIGRM
SFGRDITFERDPTWWAQNLNVRRGMFNFDRITYKIYRDPTAQVQAFL-AGEFDYMQSF--ISREWARAL-TG
RPFDRGEIIKRELAH--ANAGDFQGFLFNTRRPQFQDPRVREALALTLDFEWMNRQLFFNSFERMRGFFVAS
DFEAR----GLPGPDELALLEPLR----AQLPPQ------VFTQPVPLPSVTRLELDSGQTLRDHLRRARDL
LAEAGWRYR--DGALR
NAQG--QAFTLEFLDSG---GGMGRVVTPMQNNLRRLGIESSYRVI-DFALLQQRLD---RFDFDVISTR-W
LG----SEAP-GAELLDRFGSAAADTEGSSNLIGVRDPAVDALLHRAVAARTRPE-LVTTLRALD-RVLRHQ
HLVIPHWFGS---------THRIAYRAGRFEQPAVLPRYFQP-EGWILATWW--------------------
----
>Polynucleobacter/1-585 cosmopolitanus
-------------------------------------AHGFAQYG---DLQYPPGFKHFSYVNPNAPKGGTI
FLPNPDRR-----TSFDKFNPFSM-------KGVAAPGVAQ-----------LMFESLLIGS---------A
DEVASSYGLLADDVSLAKDE----LSVTFRLNPLARFNDNSPVLAKDIKYSFDTLMSK-LASPQFRTVYADV
KQAVIISERVIRFDFH--RKNTELPLLVG-SMPIFSEKWGKDAQ--GAGKPFNQFTFE-KPIASGPYLIESY
DIGKNIVFKKNPEYWGAELNVRVGFFNFEKVSYRLYKDDTARIEAFK-AGEFDTLVEY--RAKNWAKSY-VG
PKFRDGTLKKQHFQH--RNGAGMQGFVMNTRHPIFKDQRVRQALTLALDFEWMNRQLFYGQYHRIDSYFSNS
ELGASFEAGSLPSAQERKLLEPLQKAYPQHFPKN------ALG-PMLLPVTTEE-PSS---LRQNLRKAREL
LAQAGWVYK--DGALR
NAQG--QAFHFEFMDDG---GAMSRLVSAYARNLEKLGMKVDIRTT-DYALYQKRLE---EFDFQMTSMK-F
PD----SQSP-GNELWDRYGSQSATTKGSDNILGVQSPVVDALISSIVKAQTRKD-LITATRALD-RVLTHS
YYIVPHWYSA---------THRVAYKS-DMGFSIP-PNYF-AAESWILSTWW--------------------
----
>Herminiimonas/1-606 contaminans
--------------MAKLLPSFLLISCLSLPGSAAFAAHAFSLYD---TPKYPPGFTHFDFVNPDAPKGGEL
FLANPDRR-----TSFDKFNPFSM-------KGVSAAGLTT-----------LMFESLTTNSSGSEAI---T
DEVATMYGLLAEDMELAPDR----MAMTFRLNPKARFNNGDPVLAADVKHSFDTLVA--KGAPQVKSLLENV
KQCVVLNERTVRFDFK--TLNREMPLIVG-SVPVFSRKWAAG-------TDFDKIQLT-APITTGPYLIDRY
DTGRSISYKLNPNYWGKDIPTRKGMYNFNRINYRFYNDNVARLEAFK-AGEFDLVVEN--SAKNWARSY-IG
PKFNNGSIIKRELAH--SNGAGMQGFLMNLRRPQFQDVRVRQALGLALDYEWMNRQLFFGSYKRIYSFFNNS
PMAAT----GLPSAEELVYLEPLR----KKLDPA------VFG-AAPIPPDTNP-PGS---LRANLLKAVEL
FRQAGWEYR--DGALR
NAKG--EVFAFEIMDDQ---SAMTRVIAVYVRNLQKLGIQVTQRTA-DYALLQKRME---EFDYDMTSIR-F
PD----VSTP-GNEMFDMFGSKAADEKGSSNFWGLKDPAVDRLVEGLVAADSRSQ-LQGAARALD-RVLLNK
YIVIPHWYSS---------THRIAYWN-RFGMPGKLPLYY-QPDPYVISTWWQDKAK---------------
----

>Hansschlegelia/1-579 quercus
--------------------------TAQVREDGLTPRHGVAMHG---EPTLPVGFRNFDYARPDAPKGGRL
VNGWLG--------GFDTLNPYAYKG-------HQAQGLK-----------GFVYESLMARNYDE-------
--PFSLYGLVAESVELPDER----DFIVFHIDERARFSDGEPVTAEDVVFTFELLRDRG-HPT-FRSNYRRV
KAATIEGERSVRFDLSGADDR-ELPLILG-LMPVLAAHATD-------AATFESTTFK-PLLGTGPYIISAV
DPGRSITFRRDPDYWGAELAVNRGMNNFDEIRFDYYRDDNVMFEAFK-KGLVDVRPEG--DPTRWATG-YDT
PAVRRGEVALESFTS--GLPWGMNAFVFNTRKPIFSDPRVREALGRLFDFEWIDRNLFAGATVRTQSYYEGS
ELSSH---GRPADARERALLAPFP----SVVRE------DVMEGRWSAPSTDGS-GRD----REQARQALDA
LAEAGWAVKDG---ELRR
TSDG--EPFRFEIMVST---KDHERLSLAFARGLERIGVRATVRLA-DSVQAFRRLQ---TYDFDMVVYNWV
S-----SLSP-GTEQKKYWSVAAADTPGERNYMGVREPAVDAMIAALLAARERPD-FVSAVRALD-RVLLSG
FYVVPLFHVK---------EQWIARWK-TIERPEKT-SLYGPT----PETWW--------------------
----
>Segnochrobactrum/1-582 spirostomi
--------------------------------------HGLSVFG---DLKYSPDFKAFGYVDPTAPKGGRM
IFMPPYWVYNQNPQTFNTLNSFVL-------KGDAPPRMELC------------FATLMERA---------L
DEPDAVYGAAAESVEILDGG----NTVRFRLRDGLTFHDGSPLTADDVVFSVTILKEKG-HP-QITELTREV
VSVTADDSKTVTMRFTGRQTRQLPQSLAQ--LPIFSKAWYATR-------DFGAATLE-PPLGSGPYKVGRV
EAGRVIEYERVADWWGRDLPVHRGTFNFDVIRVLFYRDRQVSFEALK-KGDVTFREEF--VARTWETGYD-F
PAARDGRVKKAALPD--QRPAGAQGYFLNMRRPAFADPRTREAIGLCFDFEWTNKTLFYGLYQRTQSFFQNS
-PMMAE---GEPSAAELALLEPH----RAALPKE------VFGPAITAPVSDGS---GQ--DRKLLRRAAEL
LEAAGWK-R---QGGN
LVDASGAPFTIEFLEND---GATSRIVQPFIRNLKLIGITAAERII-DPVQYQRRQDT---FDFDVVGA---
--RYSLSPTPGEE-VRTFWTSAAARQDGSYNLAGIADPVVDALVATMINAPTRED-MTNAGRALD-RVLRAI
RPWVPNWFRA---------TYTVAYWD-LFGGP--PAPPAYGW--PPETLWWFDAARAAAI-----------
----
>Hartmannibacter/1-584 diazotrophicus
-------------------------------------GHGLSFFG---DLKYPAGFARFDYVNPDAPKGGEV
ATQVASWAYNQNPSTFNTLNSYVL-------QGDGAYGMSLT------------FASLMGGT---------L
DTIDNLYGFVAEEVEVSGDG----RTYRFFLNKAATFHDGSPLTAADVIFSIETLKTDG-HP-SIALQLQHV
EEAVAEDDRTLRIVYAEGTPGSIPLTVAG-GVPIFSAAWWKGR-------DFKATLSE-PPLGSGPYKVKDF
TFGRTIRFERVKDYWAVNHPTMIGSTNFDIVRYEFFRDRNTAFEALK-KGTTTFREDH--SSKSWATAYD-F
PAFKNGEVVRDIVPD--GSPSGAQGWFMNMRRPKFQNPLVRQALAMAFDFEWANANLMYDTYKRTTSFFENS
-DMKAT---GEPSPEELALLEPL----KAEIPPE------AFGPAILPPVTDGS---GR--DRKVLRAVQDL
LKKAGCT-R---EGDR
WLLPGGEPLTFEILDDD---NVFEPIVGSYFSTLKLIGIPANFRLV-DAAQANARVRD---FEFDMTPF---
--RSSMPVYPDNY-FKLIFGSEAADQQGSRNLAGIRNKGVDSLIGTIIAAKTRPD-FVNACRALD-RVLRAI
YPWVPHWYSG---------THKLAYWD-VYEHP--ETLPPYGV--GLLDIWWLNKDKAQKL-----------
----
>Sulfitobacter/1-596 sabulilitoris
-------------------------------------SHGYSFYG---DLSYPPDFPHFDYVNPNAPKGGEI
AFSALG--------TFDSMNPYSR-------KGRAGTLSWMI------------YESLLGEMP-AEGGSAPA
DAYGEAYGLLAERVEYPASK----EWVIFHMRPEARFSDGTPVTAHDVLFSHNLLLDEG-LKSYGDAVRKRI
PNAEVIDDHTIKFFFAEGISRRSLIDQVG-GVPVYSKAWYEKT-----GYGLDESRLD-VSPGSGPYMIDSV
DVNRRIVYKRNPDYWARDLPINVGRHNFDTIRVEYFGDENAAFEAFK-AGEYTFRTEG--NSRQWATAYD-F
PKVNEGLIVKKELPD--GSPPTPSGIVFNLGREVLRDKRVRQAVALGFNFEWTNESLQFGLFKPRASFTQDT
-KLMAE---GVPQGAELAFLKSLG----DVVPAE------MLTEPAVVPHESNA-DRLL--DRRNLRRAMKL
LDEAGWPVG---DDGL
RRDAQGQPLRLTFLLNSAGSSTLRAVVENFLSNLEAMGIQVTLEAV-DASQYTSRERDR---DYDMVYD---
--SYAAFLGTGTG-LAQRYGSEAAEY-SLFNPAGLASPMVDAIIEASLLAQSAEE-EDTTLRALD-RALRYE
FFMIPTWYND---------SFWVAYYD-QYEHP--QVLPPYAL--GFLDFWWYNQDKAEALRAAG-------
----
>Albibacillus/1-591 kandeliae
------------------------------------ETYGYNFYN---ELSYPADFKQFAYVNPEAPKGGEI
SIGVLG--------TFDSMNPYTI-------KGRAGAWSSSM------------YESLLGEG--------PA
DVYGEQYGLLAERVEYPEDM----SWVIFHMRPEATFSDGTPVTAEDVLFSHNLLLEEG-LASYAAAVKARI
PNTEVIDEHKIKFYFTEGISRRSLIDQAG-GVSVWSKKWFEDT-----GARLDQSRLE-TSPGSAPYVLDSY
DVNRRIVYKRNPDYWGWHLPVNVGRHNFDRIRFEYFGDDTAAFEAFK-AGEYTFRQEG--DSKKWATQYS-F

```
PKVQNGQVVKTELPD--GTPPADSGIVFNLRRPIMQDKNLREALTLAYNFEWTNESLQYGLFEHRVSFMQDT
-PVMAE---GVPEGEELEFLKSLG----DVVPAD------LLTEPARMPPTSSA-ERLN--DRRNLRQAAKL
LEDSGWVVSQ----------------------------------------------------------NDGK
RRNAEGQLLELTFIFNSSNEGTLSAVVENYIKNLEIIGVTPKLEKV-DAAQYTSRERAF---DYDLIFD---
--SYIPFLGVGTG-LMQVYGSESAAV-STFNPAGLASPLVDTIIEQALAAQSRQE-EETRLKALD-RALRYE
MIMIPAWYRG---------SYWVAYYD-QYEHP--DPLPQYDL--GYLDFWWFNQEKADKLKAAG-------
----------------
>Polaromonas/1-592 glacialis
-----------------------VLVCVAAFAPQSRAAHAYAQFG---DIKYPAGFTHFDYVNPAAPKGGEI
RMVPPTRP-----TNFDKFNPFTL-------RGTAPYGLGI-----------LLIESLLTGN---------S
EEPTTAYGLLADDVAVAPDQ----LSATFRLNDKARFHNGAPVLAADVLHSWTQLTSK-LAAPQYRTIYAEV
KGVTVVSERVVRFDFL--TPNPELPLVVG-GMPVFSRDWGVVN---GQPKPFDKIVSD-IPIGSGPYKIASP
AMGRDITYVRDPAYWGADLPSRKGQFNFDRISFKIYLDETSRFEGLK-AGEFDFLREF--ISRNWARQY-TG
KHFTSGELVKRAFEN--RNPGDFQGYVFNLRNPKFQDARVRRAIGLAMDFEWMNRQLFYGLYKRVNGYFPNS
EFHAE----GLPQADELALLEPLR----AKLKPE------VFG-PVPVSPSTTP-PGS---LRENLRQAQAL
LREAGWTYR----------------------------------------------------------DGALR
NSKG--EAFTIEFLNDQ---PSLVRIVGPFQKALEKLGITMTYRIV-DFSLGKQKMD---AFDFEISTLR-L
PG----STAP-GGELLELFGSKAAATPGSSNVWGIADPAVDALLQKVVTARTRPE-LGAAMRALD-RVLTHG
YYSVPQYYGD---------AFLIGYRPRPFVLPPTIPPYYQP-DTWAMSTWW--------------------
----------------
>Sansalvadorimonas/1-585 verongulae
-------------FIAVVFLWTLAHFLQAEGDNSVTFSHAISLYGT--P-QYGADMKHFDFVNPDAPKGGTL
NVAQIG--------SFDTFNAYGP-------NGKVPFG-LTY-----------TNETLMMRGWDE-------
--PLSKYGAVAEKVESPEDN----SWVAFHINPKAHFHDGKAITADDVVFSFTTITEKG-SLFWRQ-FYQDV
DTVEATSPQRVLFTFKHN--QNRELPLLLGQLPVLASHWWQ-------NRDFSETTLD-IPIGSGPYKIHRF
QPGRFVEYKRDPNYWAKDIPTNIGRYNFDIVRYDFFRDNHITVEAIN-SGQLNWQLES--DPRFWEKGYS-Q
RALDKGKLVKSTWVN--HNPQ-TDSLVFNTRKPLFSDVRVREAIATLFDIDWVLSNLLNNTSERATSLFAGS
ELAAA----GLPNSEELALLTPLK----DHLPQ------RVFT-HQWPPFTG---MKK----REKLKHALNL
FQQAGFLLVN---------------------------------------------------------GKIMQ
AD-G--QPVIVEMLLGDPS-LE--RLMQGQVQRLAEAGIEMRLRTV-ESARYLKHIR---ELDFDIIL----
-HSFRHTPSPG-QEQMSFWGSEGSDQPGTLNFAGVNNPAIDNLVHRIPSAKTWPE-LVTLVHALD-RSLLWD
FKVVPLFYNS---------SWRVIHSS-NIKHPERLPKFS-----LERATWW--------------------
----------------
>Ketogulonicigenium/1-612 vulgare
----------VIVLAAFGFLGAALWSGAARAQEETITSHGFTTFGDLSELTYPADYQHLNYVNPDAPKGGTI
SVWAQG--------TFDSFNPYAR-------EGLSGAMATIG------------YESLLEAT---------A
DTVSDAYCLLCETLEYPESQ----DWVIFHMRPEARFSDGTPLTAHDVVFSHNLLMEQG-LPSYREAVAPLI
TNVEALDDHTVRFTFAPDVPRKGLIVQAG-GSPVFSQAWYEST-----GARLDQTQVE-TSPGSGPYVRAAF
DFNNYITYRRNPDYWGADLPINQGRHNFDEIRVVYFADANVAFEAFK-AGEYTFRQEN--SSIVWATGYD-F
PALNNGYVVKDELGN--GNLPPAVGFVLNLRRPEFQDIRVRQAIALMYNFTWTNDTLQYGLFQQRNSFWQGS
-PLEAH---GTPEGLELEYLESVA----DLIDPA------ILTEPVTMPHTAG--ANQL--DRANLRRALAL
LTEAGWESG-----------------------------------------------------------ADGR
LM-KDGQPLTLELLGYS---PTFDRIVTPFIENLNTLGIAANYNRI-DPSQYTVRTRAN---DFDMVYD---
--GYTTGLEEGLG-LAQRFGSY-AVD-DVFNPAGYSSPAVDRLIEIAVDAQNYDE-MSAAVRGID-RIMRRA
LFVIPSWYNA---------NFWVSYYD-MFEHP--EEMPPYAL--GQLDFWWYNAEKADGLRAAG-------
----------------
>Pseudopuniceibacterium/1-581 sediminis
--------------------------------EKIIKSHGYSYFG---ELKYPADFDHLDFVNVDAPKGGDI
AIQASG--------TFDSLNPFTV-------KGRAGALASTM------------YESLLGEA---------A
DDYNGSYCLLCESLEYDEGK----TWVIFHMRKDAKFSDGTPLTAHDVVFSHNLFIEQG-LPSYSAGVKKRV
LGAEALDDYTVKFTFATDISRRSLIDQVG-ATTVFSKKWFEET-----GARLDEPRFE-TSPGSGPYMLDSY
DVNRRITYKRNPDYWGKDLPLNKGRYNFDTIRVEYFADESAAFEAFK-AGEYTFRIET--SSRQWATQYD-F
PAIQAGHVVKAELPD--GIPPNNVGFVFNLGREKFKDKRVREAIALAYNFEWTNESLQYGLFKHRESYFQDT
-PLQAN---GVPTGEDLAFLEGLG----DLVPDA------LKSEPPVGIHVSSP-ERLN--DRGNLRKAMKL
LDDAGWAVG-----------------------------------------------------------DDGI
RRNAQGEVLSIDFPINSASASSLEPVLDNYALNLKAIGIRPDFEKV-DPSQYTNRSRNR---EYDMVYD---
--GYRAGLGTGTG-LMQMYGSSEAAF-SVFNPAGLASDLVDQIILASLETQSQEE-QDASLRALD-RALRYE
MLMVPTWYKA---------GYWVAYYD-MFEHP--D-IPPYDL--GYLDFWWYN------------------
```

```
----------------
>Paroceanicella/1-551 profunda
--------------------------------------------G---EPALPEGFDHLPYANPDAPQGGRI
VMGELG--------GFDSLNPFILKG-------NAPWGLR-----------SHVFESLMGRSIDE-------
--PFTLYGLLAATIETDDAR----SWVEFTLRPEARFSDGNPVTVADVMFSMQVLAEKG-LPG-FASSWAKV
AKMEQTGPRSIRFTFS-EEDR-ELPLILG-LRPILEKADWE-------GHDFAESSMR-SPVGSGPYVIADA
KPGRSITFRRNRDYWGDGLGFNRGRNNLDEIRYDFFRDSNALFEAFR-GGLTSVQREG--DASRWQNA-YDF
PAVRDGRVVLSTIPN--GRPTGMHGFVFNTRRAVFSDIRVREALTLAFNFDWINARLLDGAFRRIPSYFANS
PLGID---GPAGPGER-ALLGSFA----EELPA------DIFAP-EEPENGDAG-GRN----RGALRKARKL
LEEAGWHIGSG---------------------------------------------------------GVME
TADG--TPLSFEIMLR--A-ADDEAVASIYVDALKSLGIDARARLV-DDSQYNARRT---TYDYDMMVNTWA
L-----SLSP-GNEQEFYWGSAGRDMEGTRNYMGVASKAVDALIPDMLAARSMED-FTSAVRALD-RVLTKG
RYVIPFWYTP---------ESWIAHEA-TLHYPQTT-PLYG-------------------------------
----------------
>Azohydromonas/1-579 lata
----------------------------GAAAEGGRWVHALATFG---EPKYPENFTSFEYVRPDAPKGGVL
KLRNPDRR-----TSFDKFNPFTV-------RGNAPAGVSI-----------YMFESLCTGA---------D
DEPQTMYGLLAERFIVAPDK----SSITFRLDPRARFWNGDPVTAEDVKFSFERLTSK-QASPALVQTYAPA
GPVTVLDERTVRFGIK--DRSLDTVFLVG-GLPVFSRKWGLGAD--GKVKPFDQVVGE-YPITTGAYVIDRV
DMGRRIEFKRNPDYWAKEHPTRRGWFNFDRVVYRMYKDGSVAFEAFK-TGEYDIHKEY--SSRAWARQH-KG
PKWDDGRIVKNDFET--ATGQGLQSHQFNTRRELLSDRRVREAIALAYDFERINR---FGLLKRAYSVFNNS
EFAAE----GLPSAGELKLLEPYR----KALPPE------VFG-PAFRPPSTAGGAAD---LRRNLLKARAL
LEDAGWKLDG---------------------------------------------------------DGLLR
NAQG--RALELEYLNPG---ETGGR-ISDWQKNCEKLGITLKERNV-DFALYRNRLE---NYDFDVITIV-E
GK----FTLPNAASYITSYGSQAADEKGNGNFRGIKSPALDHILAVMSKVQTLAE-LRDCCRAMD-RIVMWN
HWQVPDVYVS---------AERASYWN-RFGIPAKQPLYFGIDT----------------------------
----------------
>Hansschlegelia/1-567 beijingensis
--------------------------------------------G---EPALPPGFERLPYARADAPKGGRL
TNGWLG--------AFDTLNPYAYKG-------VPAQGLK-----------GFVYESLLARNYDE-------
--PFALYGLVAESVELPDDR----DFIVFHIDPRARFSDGEAVGAEDVLFTFELLRERG-QPA-FRSNYRRV
RNAAVIDQRTIRFDLP-TDDR-ELAMILG-LMPVLASHATD-------VGRFEQTTFT-PLVGTGPYGVSAI
DPGRSITYRRDPNYWGRDLPVNRGVNNFDEIRYDYYRDANAMLEAFK-RGLVDIRIEA--DPVRWTTG-YDI
PAVRRGDVVLESFTS--GLPWGMNAFVFNTRKPIFSDVRVREALGFLFDYEWVDRNMFGGATVRTQSYYEGS
ELSSH---GRPADARERELLARFP----GVVRD------DILEGRWSAPSGDGS-GRD----REQLRKALDL
LAAAGWSLKGG---------------------------------------------------------ALAR
DSDG--APFRFEIMVST---KEDERLALAFARGLERIGARADVRLA-DSIQAFRRLQ---TYDFDMIVYNWV
S-----SLSP-GTEQKKYWSAAAADTPGERNYMGVKEPAVDAMIDAMLAARERPD-FVSAVRALD-RILLSG
FYVVPLFHVR---------EQWIARWK-AIQRPDVT-PLYGPV----LETWWRATSPAG-------------
----------------
>Palleronia/1-581 sediminis
----------------------------------IITAQAFSNLG---AIKYEPGFEHLDYVNPDAPKGGEF
STWGRG--------TFDSMNPYSR-------RGRSPALSVLP------------YERIMTNT---------A
DDPYASYCLLCETLEYPETQ----DWVIFNLREDITFSDGTPMTAEDVAFTYELFMEEG-FPSYRIAVKRMI
PSVDVLGPHRIRFNFNPDVPRKGLISQAG-AQIVFQKKWFEEN-----GAKLDESRLT-QPPGTGPYRIAEV
VAPTRIVAERVPDYWGADLPINVGRWNYDTIRQEYFADTSAALEAFK-AGAYTFRQEA--SSLTWATQYD-F
PAVEDGTVVTETLPD--GSLPVASGFVFNLRKDKFQDVRVRRALGLMYNFEWTNETLQYGLFERRQSFWQNT
-DLEAK---GVPEGLELDYLNQVA----ELIEPS------ILTEEVFTASDGG--ARQL--DRGDLRRASAL
LEEAGWITG-----------------------------------------------------------DDGL
RR-KDGEVLSVEFLESN---PALDRIILPYIDNLKRLGVDARYNRV-DDSQYTDRVRNH---DFDMVFD---
--SYTNGLEEANG-IAQRYGCD-DRD-DVFNPAGFCDPAVDALIERLQVAQTLEE-MQAAVRAID-RILRAA
YFMVPVYYNA---------AYWVAYYD-MYEHP--ENMPPYAL--GEQDFWWIDAEKAEAL-----------
----------------
>Brucella/1-612 melitensis YejA2
MTFLCMNRRHFMGLSGSAALVACLPGQAFADQPTGKALHGLSAFG---DVKYGPDFSHFDYANPDAPKGGCF
TLAPLGWIWNQNTLTFNTLT---L-------KGDAPPRMELT------------FDSLMVSA---------L
DEPDSVYGLIAESVTLSKDR----KTCTFKLRKEARFHDGSPIEASDVAFTYKTFKEKG-HP-TLRQSLAGL
QKVEATAKHEVQMHFAAGRGPNAILDAVT--LPIISEKWFKGR-------NFEATSME-PVLGSGAYKVGNF
```

SAGHFIEYDRVDDYWAKDLPVQKGSNHFDRIRIEFFHDRQPAFEAFK-KGSIDFCEEN--VAKNWATAYD-F
PAIHQKKVIRRTFPR--EKRPLMQAWAVNQRRERFRDPRVREAIGLCFDFEWSNKNLFYEAYTRSQSCFNGS
-DFQAN---GLPSPDEMKILARY----RGKLPEA------VFGEAVLVPVSDGT---GR--DRKQFTQAIKL
MEEAGFE-R---RHGH
FHDHDGSNFALEILCNS---EAVTRIYNPFAQKLRAIGIDTSLRLV-DASQYQARLQD---FQFDMVGV---
--ALQFSATPTKESLAGIFGSDSAKIPGSHNLPGTNDPLIDALIEDAGKAESREE-LVATLRVLD-RYLRIR
RDWIPNWTAA---------NHFVAYWD-RFGFK--EPKPDYGF--PVETLWWIKA-----------------
----
>Brucella/1-622 melitensis YejA1
-------MKAFWVSAFAVAMLASAMPASRADNAEPQWRYSSSLLD---EPKYPADFKHYDYVNPDAPKGGTL
NLVAVG--------TFDNLNPYVV-------QGVSAAGL-------SDFGGGMLYDTLMADSQDQ-------
--GSTQYPLIASALQYPDDF----SWVKFKLNPDAKWHDGQPITVDDVIWSFNVLKKQ---SPMYNQYYSDV
ESAEKTGEHEVKFTFSRKG-NRELPQIMG-QLAILPKHWWTAKDARGKQRDITRPTLE-IPLGSSAYKIESM
KPGHSIIWARVEDYWGKDLPVNVGRNNFDHVAYEYYFNEDATWEAFKKGGQYDYRNEN--RAQRWAEQY-NF
PAVQRGDVVKASFPF--HAVGRMQGYFLNTRRDKFKDPKVREALTYAFDFESMNRLMFYNQYKRINSYFAGN
ELAL----SGPPTPAEQAILETVK----DALPA------DALTKEFKLPVYDTP-QAT----RENLRTALKL
FSQAGWTLKG---NTLVD
AK-G--NPFTIEFLGQ--D-PTDERIYNPFAASLRKIGINATVRIV-DAAQYQARVN---DFDYDVI-TAVI
AQ----TASP-GNEQRDMWGSKAADFKGSRNYAGIRNPAIDKLIDLVVYAKDHEE-LEAAAHALD-RALLWN
YYVIPQWYS---------DHINVAYWN-KFGMPE--KQPDYLGID--PYSWWIDPAKEAKLKTGGA------
----
>Xaviernesmea/1-602 oryzae
-------------------AQSQAG-----EASAPAWRHGLTTVG---TLKYPEGLKRFDYVNDKAPKGGEV
RLNLMG--------SFDSTNPLLA-------KGETAVGLAGPVADASGLAAGLVFETLMKPSMDE-------
--ISSSYGLIAESVAFPEDI----AWAKFRLRPEARWADGTPITPEDVIFSFEMTKTL---NPQSEFYYKHV
VKAEKTAEREVTFTFDETG-NKELPHILS-TLVIVPKLWWQGQGADGKPRDISRTTLE-APMGSGPYRLVSL
TPGSTLRYERNPDYWGKDLPVNVGQNNFDAITFTYFADLDVAFESFR-SGNADYRWEN--SARRWATAY-DF
PAVKDGRILRESLENENRNSGVMVGFIYNMRRAPFDNPKLREALNYAFDFEELKRTIFFGQYDRIDSFFFRT
ELAS----SGLPEGKELEILNSVK----DQIPP------EVFTKPYVNAVGGDP-AKM----RDNLRQAIAL
LKEAGFELK---NNRMVE
VKTG--KPLKLEMLLD--G-NTIERVALPFAQNLKRIGIDASVRSV-DSAQFTNRWR---GRDYDMLYLG-W
AQ----SLNP-GNEQTEYWGSQSATREGSQNFSGISNPGVDALIRKVIFARDRDE-LIAATHALD-RVLLAH
HIIVPSYAS---------KDSNIAYAK-RLKHPD--PLPEYGVG--FPAIWWSD------------------
----
>Schlegelella/1-569 aquatica
---FG---DIKYPPGFAHFDYVNPAAPKGGSI
VLVPPTRV-----SNFDKYNPFTL-------KGIAPPGLDG-----------LVFESLLTRN---------F
DEPTTAYGLLAEDVEVAADR----RSVTFRLRRIARFHNGDPVLAQDVKHSFEMLTSK-QAAPQLRSYFSDI
ERVRTVDDRTVIFEFK--RVNAELPLIAG-DMPVFSRKWGAGK-------PFDEIVTD-IPIGSGPYKIGKV
NFGRDITYVRDPQYWARDLNVRRGMFNFDRITFQIYKDNTAQLEGFK-AGEFDYIQAF--IAREWARQY-TG
PKFASGELVKRELPH--GNAGDFQGFIFNTRREKFKDPRVREAIGLAMDFEWMNRQLFYNAYTRVRGYFPSS
DFEAK----GLPGPDELKLLDPLR----PHLDPA------VFTQEVPQPPSTRP-PGS---LRANLRRAKQL
LAEAGWTYR--DGALR
NPQG--EALTLEFLDES---ASMGRVVTPYIGNLAKLGIQASYRVV-DFALLQKRLD---VFDFDLISLR-L
RG----SEAP-GSELLDRFGSQAADTEGSSNLIGVKSRAVDALIEHVMSATTRAE-LVASLRALD-RVLRFG
HYVVPHWYSS---------TYRIAYRAGRFEQPRTIPRHYPP-EGWVLSTWW--------------------
----
>Shimia/1-592 gijangensis
-----------------------------AEEEKIIKSHGYSYFG---DLTYSANYTHFDYVNPEAPKGGEI
ALWAPG--------TFDSMNPYSR-------KGRAGRYSWMM------------YESLLGDAP--TGGGMPA
DVYGESYGLLAERLEYDEGK----TWVIFYMRPEAVFSDGTPVTAHDVVFSHNLLINQG-LPSYGQAVKKRV
LTAEALDDHTVKFTFATEMSRRSLIDQVG-GVPVYSKAWYDET-----GARLDEPRLE-TSPGSGPYMIDSY
DVNRRIIYKRNPDYWGWHLPINKGRHNFDRIRIEYFADDSAAFEAFK-AGEYTFRQEG--NSKQWATGYE-F
PGIEKGWVKKAELPD--GTPPSPTGIVFNLGKEPLKDRRVREAISLGYNFEWTNESLQFGLFKQRHSFVQGA
-PHEAM---GLPEGKELALFDSLG----DLVPEG------ILNEPVVSAHESSP-KREV--DRRNLRKAMGL
LDEAGWDVG---DDGM
RRNTNGQLLKLNFPIPTSSSATLGAFVEVFVQNLRKMGIDATVEKV-DPAQFTLRERDR---DYDLVFD---
--SYVAFLDTGTG-LHQRYGSEEAAF-SLFNPAGLASPLVDRIIDISLETETRED-RDVALMALD-RVLRWE

```
RFMIPVWYND---------STWVAYFD-QYEYP--DPLPRYAL--GELDFWWFN------------------
----------------
>Faunimonas/1-593 pinastri
------------------ITLWLGLCLVAGRAAWAEPTYGIAMHG---EPALPADFTHFPYANPDAPKGGSI
TYAGVG--------SFDSLNPFILQG-------TAPRGLW------DMTWGNNIWESLLVRSKAE-------
--PFTMYGLIASTVDVAPDL----STVDFQLRPEAKFSDGVPITPDDVIFSFDLLREKG-RERPYRETYKLF
DRAEKLGDHGVRFVLKPGTNR-ELPLLIG-LMPILPKHAVD-------AATFDRSSLK-IPIGSGPYTIAAV
DPGKSVTLKRDPNYWARNLPAKRGFDNFDQVRVEYYRDASAYFEAFK-KGLFDINPEN--DPARWAIA-YDF
PAVHEGRVVLDTFPI--QIPAGMNGFFFNTRRPVFEDARVRQALTYFLDFEWLNRNIYYGKYERTGSYFEGS
ELSAI---GRPATDAERRLLAPFP----DAVVP------DVMNGTYRPPESDGS-GHD----RDNMRKGVEL
LEQAGYGLKNG---------------------------------------------------------LMVN
LKTG--EPLAFEIMTNT---REQDRLALAFSRTLALAGIRVRIHDV-DSAQYERRVK---NFDYDMIQILVP
-----ASLSP-GTEQAGRWSSAAADAPGSFSYSGVKSPAVDAMINAMLAAHGQGD-FVSAVRSLD-RVLISG
FYVVPLFHPP---------GQWMGRWA-RVEHPEQA-SLWGPE----PTTWW--------------------
----------------
>Falsirhodobacter/1-572 deserti
----------------------------------ILRSHGISTFG---DLKYPPDFPHLDYVNPDAPKGGEI
SVWAQG--------TFDSFNPYAR-------VGRAPAYSVLP------------YESLLADV---------D
DEVSASYGLVARELEYPETQ----DWVIFHMRPEARFSDGTPVTAHDVAFSHNLLLEQG-LPSYAQSVKVSI
PVVEVIDDHTVRFVFGEGVPKKNLISQAG-GTPIWSKAWYEKT-----GARLDESRFE-TSPGSGPYMIDSW
DVNRRITLKRNPDYWGRDLPIMKGRANFDSIRVEFFADGSAAFEAFK-AGQYTFRQEN--SSVIWATGYD-F
PAVNRGDVVREELPN--ASLPAATGFVFNLRRPQLQDRRVRQALALMYNFTWTNDTLQYGLFQQRESFWQNS
-DLQAK---GIPEGRELELLQEVA----DQIPAA------ILTEEVTVPHTSG--DRQL--DRRNLRTALAL
MQDAGWMPN-----------------------------------------------------------ETGQ
LM-KDGQTLKVEFLTQD---PQMDRFVIPYIDNLKALGVDAGYRRV-DPAQYTSRQRSF---DWDMIYD---
--GYTNGPEEGTG-MGQRFGSE-DKD-REFNPAGYGSAALDHLITRVIEAQTHED-MAAAVRAAD-RLLRYE
MIMVPTWYLG---------KFWVAYWA-QFGRP--DPLPPYAL--GQLDFWW--------------------
----------------
>Lautropia/1-603 mirabilis
-------------------LVLGAAALAGWPGREVLAAHGMAWGG---EPRYPESFAHFDYVRPDAPRGGLV
RLAGMG--------TFDSLNPFTL-------RGIAAAGTG--------S---LMFESLAVAS---------W
DEPFSVYGLLAEDMILAEDR----LSVMFKLRAEARFNDGSPVLAEDVRHSFETLVGP-KGHPLFRQYFGDV
ERLEVMDNRLLRFFFK--RTNPELHLILAKDLPVFSRRWGQG-------KAFDAMPHE-RPITSGPYVVDSM
DLGKRIAFKRVEGYWADALPVRRGTFNFERVVFKYFRDEVARLEAFK-AGEFDWLFEN--SARNWTRGH-VG
ARYRSGEIVKRNFPH--SNVSGMQGYALNTRRPLFKDVRVREALALAFDFDWLNRHYFQGQYTRTRSYFANS
AMAAS----GKPDAEEMRFLKSLS----SPLDPA------VFG-ELPELPDNPD-ADA---LRRNLKRAQQL
LNEAGWQVDT---------------------------------------------------------DGVLK
NAQG--QAFRFEVLGDA---PAFERLATPWIHNLARLGIRVRQRMV-DPALYQKRVS---TFDYDVVTTV-Y
PM----SSTP-GNELLQMLGSAAANDARSGNYSGVADPAVDEIIERILQVRSRDE-LQLATRALD-RVLRHG
WYMVPQFHSS---------SYRVAFDW-RLRHPGVLPLFY-DPQSWLLETWWRDDEARPVPVAEQ-------
----------------
>Thioclava/1-590 indica
--------------------LALGFSPIFAQNASAESQDAIAMYG---APALAPGFDHLPYANPDAPKGGEY
RFGVAG--------SFDSLNPWILNG-------RPAQGIA-----------NYVAESLMGRSMDE-------
--PFTLYGVLAQSIKTDEAR----SWVEFTLRPEAKFSDGNPVTIADVMWSYETLGTQG-HPR-YQNAWFKI
AKMEQTGPRSVKFTFK-QPDR-ELALLMG-MRPVLEKAQWE-------GRDFANTTNL-APIGSGPYVIEKV
DQGKYITLKRNPDYWGRNLGFNKGRNNFDTLRYDYFGDGGVVFEAFK-GGATDSYTED--SAADWARN-YDF
PAVTSGDVVKSEIPN--GRPSGIKGLVMNTRNPIFADWRVRQALIDAFNFEFINKTINGGAEPRITSYFSNS
ELGMS---HDAASGKVLDLLTPFK----ADLPP------GVIEG-YSLPQSNGS-TRN----RKNMRAAAKL
LNEAGWKVDDS---------------------------------------------------------GVLR
-KDG--QPFTFEIVLQQGA-AQQQSIVDIYTQSLKRLGIDAKVTVI-DDAQYTQRTN---AYDFDMAWYWRA
L-----SLSP-GNEQMLYWGHQGVTEQGTRNWMGMNSPAAEAMIDTMLNARSHED-FVAATRALD-RILTAG
RYVIPIWYAP---------KDRIAHIK-ALHYPDKT-PLYGAWPGFAPDVWWYD------------------
----------------
>Lichenihabitans/1-570 psoromatis
---------------------------------------SIAMHG---APALPADFSHLPYTNPEALKGGKL
AMCYLG--------TFDSLNPFNLKAG------STAQGLN-----------TNVFETLMTRSLDE-------
--PFTLYGLIARSIDTDAAR----DEVTFHLDPQARFSDGSAVTADDVIFTFNLLKQKG-RPQ-QRAAFSLI
```

KSVDSPDPLTVHFDLRGLNDR-EMPMTLG-LMPVLPKNHVD-------PKAFDDTSLA-PPIATGPYRVASV
VPGQRLTLQKNPDYWAKDKPIRRGMFNVDTVTIDYYRDANGMFEAFK-AGLCDYRLET--DPTRWTTG-YDI
PAVRDGRIIKETVHS--GLPKGMEGFAFNTRRPIFADVLVREALGDMFDFEWIDAVLFNGLYRRTDSFFADS
DLAST---GRPADAAERSLLAPFP----QAVRP------DILAGTWRQMTTDGS-GRD----RRPAQAALAL
LKQAGYDIHDG---LLVS
RA-G--EPFSFEIMVTN---RSQERLALAYSESLARIGVVAHVRSV-DEVQYQRRRQ---AFDFDMMFGTWT
-----ASPSP-GAEQRSRWGSASAAQEASFNLAGARSPAIDAMIAAMLSATSQDD-FVAAVRAYD-RVLLSG
FYIIPLYHSA---------DQWVAYSA-MLRHPEHL-PLFGLSNTSPIEYWW--------------------
----
>Cupriavidus/1-586 alkaliphilus
--------------------------------------AHGFALHG---DLKYQPNFSHFEYANPNAPVGGTL
TLANPDRR-----TSFDKFNPFTL-------KGTSAPGLNA-----------LMFESLLISS---------A
DESASAYGLLAEDVTVAPDE----LSVTFAIRPQARFSNGDPVLASDVKYSYDMLMSK-ASSPGYRSMCTDV
KAVVVTGERTVRFDFK--QRNRELPLIVG-SLPVFSRKW-------TAKVPFEKLTFE-PPVTSGPYLIERF
DAGRGIVFQRDPKYWGKDLAVRRGTFNFARVVYRLYKDETARLEAFK-AGEFDAIVEY--KAKNWAKSY-QG
TRFRNGELLKTEFPH--RNGAGMQGYVMNLRKPVFQDVRVRQALILALDFEWLNRQLFYGAYKRLDSWFSNS
ELSASATFDGRPGPGELQLLEPLR----AQLPPE------VFG-PDVVQPSTAP-PRS---LRDNLRLARRL
LAQAGWTYT---DGALR
NAKG--EPLVFEFLDDG---GAMSRVITTYVRNLEKLGIQVHQRTT-DFALYQKRLE---DFDFDMVSIR-F
PD----SQSP-GNELRDRFSSEAAGTPGSDNLFGLKSPVVDKLVDNVLRADTRQE-LVTAGRALD-RVLMHG
YYIVPNWYSA---------SHRVAYRK-TLAYPERLPYFY-TAEGWILSHWWRTDVQAQAR-----------
----
>Martelella/1-578 mediterranea
--------------------------------------HGIAMHG---EPALPADYLHFSYVNPDVKKGGAI
TYGVVG--------SFDALNPFVLKGM-----RSSARGLW------DEVLGNLVIEPLMQRSRDE-------
--PFTLYGLLAESAEWDDDR----SYVQFNMNPDAKWSDGEPVTADDVIFTFNLLKDKG-RPP-YNRRLNLV
DKMEKVGDNSVKFTFNADANR-ETPLIFA-LMPVLPQHAID-------PETFDTDTMT-TPVGSGPYAITKV
EPGQRIVYERRDDYWGKDTPSMVGFANYDTVTIDYYLQDSTLFEAFK-KGAVDVYLDD--DPSHWARA-YDF
PAASEGKVVKDEFHP--KTPTGMQGFVFNTRRPKFEDARVRKALSDVFDFEWVNKTLFNNAYQRTQSYWQNS
ALGAY---GNPASEAERELLGDAI----DVIDP------AILAGDYAMPVTDGS-GAD----RKVLAAAVKL
LGEAGYRIANG---KMTG
TD-G--KPLAFEIMTTN---EGQEKLALAYQHTLNLIGVALSIRTV-DDAQYQKRLN---TFDYDMIVLLAP
GFWYQSSLSP-GAEQIWRWDSRSRDVEGTFNFAGVASADVDRMINDMLEARSTDH-FTDSVRAFD-RLLVNG
HYMLPLYHRE---------GQWVARWA-TIEHPDET-PIYGYQ----LPTWW--------------------
----
>Sinorhizobium/1-597 sojae
---------------ILSGLVMLLASGLAAHQAAAEPVHAIAMHG---EPALPADFKHFPYVNPDVKKGGKI
AYGVVG--------TFDSLNPFILKSM-----RTTARGMW------DPAFGNLVYESLMQRSQDE-------
--PFTMYGLLAETVEWDEDR----TFIQFNLNPKARWADGRPVTAEDVIFTFELLRDKG-RAP-FSNRLSKV
EKLEKVGERSVRFSFTEEADR-EFPLLLA-LSPVLPKHAVN-------VETFDRTTLE-PPLGSGPYRVGEV
RPGERIVYRRNPDYWGKELPSKIGVDNYDEISVEYFLQENTLFEAFK-KGEVDVYPEG--SATKWARA-YVF
PAVQSGNVVKESFKP--KTPSGMLGFVFNSRRPMFDNIKLRQGLALVFDFEWVNKNLFDGAYTRTQSYWQNS
SLSFL---GVAADDRELELMGDVR----ERINP------AILDGTYRLPVTDGS-GRD----RNVLREAVTL
LREAGYVIKDG---KMVD
EKTG--TPLTFEIMSQN---AGQEKIALAYQRFLAPLGIVATVRTV-DDSQYQLRSQ---SFDYDVIIKSYP
-----SSLSP-GVEQVGRWGSQSRDRPGSENFAGVANKDVDKLINNILQAQTLED-FTAAVRAHD-RLLVNN
SYVVPLYHLD---------EQWIARWK-HIRRPAEV-PLYGYQ----LPTWW--------------------
----
>Cognatiyoonia/1-588 koreensis
---------------------------------ETIIETHAYSTFG---TYKYDADFSHLDYVNPEAPKGGEI
SIWFQG--------TFDSFNPYATG------KGRSGVLATIG------------YERILTST---------A
DDASASYCLLCTTMEYPENE----DWVVFNLRQDITFSDGTPLTAHDLVFSHKLLLEQG-TPSYADYVSPRI
ESAEALDDYTVKFVFADGYPRTDMITLVG-STPGWSKKWYEET-----GARLDESRLE-ISPGSGPYILDSF
DINRQIIYKRNPDYWGNDLPINIGRNNFDSIRVEYFSDSNAAFEAFK-TGEYTFRQES--SSLVWATGYD-F
PALENGWVQKVELPD--GSLPSASGFTFNLQRDKFADRRVRQALALMYNFTWTNDTLQYGLFQQRESFWQNS
-DLEAR---GVPEGRELELLQSVS----DLIDPE------VLTEEVTVPHTSG--DSQL--DRRNLRRALAL
MEEAGWVAD---DAGQ
LR-KDGEIFDLEFLASS---PTLDRIINPYIENLKRLGINASYNRI-DPAQFTNRERSF---DWDMTLN---

```
--SYSNGLEESIG-LSQRYGTE-AVG-DVFNPASFGSEAVDKLIEEVVNAETYED-MAAGVRAID-RLMRRE
LFMIPVWYLG---------NHWVAYYD-MYEYP--DELPPYSL--GHIDFWWYNAEKAEALRAAG-------
----------------
>Roseinatronobacter/1-607 thiooxidans
--------------------------VFAEGDDGLIRAHGYSFYG---DLTYPEGFEHFSYVNPDAPKGGSI
SISAQG--------TFDSMNPFTR-------RGRAGLLSSAI------------YESLLESSEPMGGGLVPA
NVYGEAYGLLAHTVEYPETK----EWVIFHMRPEARFSDGTPVTAHDVVFSHNLFLEQG-LPSYAQAVSARV
TDAEALDDHTVKFTFAEGISRRSLIDQVG-GTPVFSQAWYEET-----GARLDESRME-ISPGSGPYMLDSF
EVNRRITYRRNPEYWANDLPQNRGRHNFDEIRIEYFADGAAAFEAFK-TGEYTFRAEN--NSRQWATGYD-F
PALNRGHVVRGELPT--GFPPTPTGFVFNLGREFLQDKRVREAISLAFNFEWTNDTLQYGLFQQRASFSQGT
-RFQAD---GPPEGLELELLQSLG----DLVPDE------VLSEPARMPHASDP-ARLT--DRRNIRRASAL
LSEAGWDVD-----------------------------------------------------------SAGQ
LRNADGARMTIEIPVSSAGSATMDSIIETFTQNLQGLGIDARFQRI-DPAQHTDRRRNR---DYDMIFD---
--QYTAFMDTGTG-LHQRFGS-EAAD-DVFNPAGLRSELVDKVIDLSLDAPDPET-RDAALMALD-RVLRYE
HFMIPGWFND---------TVWIAHWD-IYQRP--EETPPFST--GEMDFWWFDADRAAELRAAG-------
----------------
>Wenxinia/1-592 saemankumensis
--------------------------------EAVTESHGYTNFG---ELRYPADFEHLDYVNPDAPKGGEI
ATWAQG--------TFDSFNNYAR-------EGVPVALNDIY------------YESILTGT---------A
DDVYGSYCYLCTTLEYPESR----DWVIFNLRDDVTFSDGTPMTAADVEFSFNLFLEQG-IAEYRAVVESYI
ESVEVIDDHTIRFDFADEAPVRDRVGFAG-GTPVFSKTWFETN-----GIRLDESQDA-PFLGTGPYVLGSF
DYNRQVVIERDPDWWGADLPINRGRYNFDTIRVEYFADGSAAFEGFA-AGEYTFRAEN--SSKDWATSYT-F
PAVENGWVVQETIPS--GAIGSGQAFVFNLDHPQWQDIRVRQAVEMMFNFEWSNETLFYGLYDRTTGFWQNS
-DLAAS---GTPSPEEVAILQPLVD--EGLLPES------ILTDEVVMPTVNGAGQNQP--DRAVFRAAGAL
LAEAGWEVG-----------------------------------------------------------PAGM
LQ-KDGQPLQMTILQVS---PLFDRIVNPFIENLRQLGIDARLDRV-DYAQYVERTR---TGDYDLVNS---
--SPGQSFEPGSG-LKQWFDSSTAGD-SSRNLMNLRDPAIDRLLPIVIEAQTLEE-MVPAVHALD-RVLRAT
RFWVPQWNKG---------ETWVAYYD-MFRHP--ETLPPYAT--GELDWWWYDAEAAQALRDAG-------
----------------
>Angulomicrobium/1-565 tetraedrale
--------------------------------------HGLAMHG---RPLYPPDFTHYASVNPQAPQGGRL
VQGVVG--------SFDSLNPFIVRG-------TVPPIIG-----------WNVVEALMARSPDE-------
--AFTCYGLIARTVETDEAR----RFVAFTLDPRARFSDGTPVTAEDVLFSFNLLRAKG-RPN-HRTYYGKV
ARAEVTGPLSIRFDFA-GEDR-ELPLILA-LMPVLARHATD-------EVRFDQTSFT-PPLGSGPYTAVEV
KPGESVTLARNPAYWGRDLPVNHGNFNVDTLRYDFYRDVNGQFEAFK-RGLYDIRFET--DPGRWKTG-YDI
PAVRDGRILTEEVGN--GRPKAYSALLFNMRREIFADVRVRAAMVELFDAEWANQNLYFGLFRRNGSFYDAS
ELSAL---GRPAEPRERELLAPFT----DAVLP------AVMDGTWRPPASDGS-GRD----RGQLKRALDL
FTEAGWQLRRG---------------------------------------------------------TLMD
PRG---RPFAPEVLVGT---KDQERLALAYQSMLRRAGVGLNIRLV-DNVQFESRKR---SYDYDMVPYIWS
Q-----SLSP-GNEQAFYFGSEAADTPGTRNFMGLKSPAADAMIAALLAERERAG-FVAAVRALD-RVLISA
RFSLPLFYTP---------GDWIARWR-RIERPKDV-SLNGTL----AESWW--------------------
----------------
>Acidovorax/1-592 radicis
-----------------------LTFLLMGCAVPAWAAHGYALWG---EPRYPSGFAHFAYVNPDAPKGGEL
RLVSNLRV-----STFDKYNPFTI-------KGNAPAYLSS-----------LMFDSLLAGS---------M
DETATGYGLLAEDVEVAPDG----LSATFRLRPEARFHNGKPVLAQDVKHSYDTLVGP-FTSPAYKTLLIEV
AGVDVINDRTVRYRFK--LPNRELPLTVG-GLPVFSRDWGVEN---GKAKPFDQVVMD-IPIGSGPYKIGPV
RFGKDITYVRDPQYWARDLNVRKGTANFDNILVKIYKDNTARLEALK-AGEFDLMRFF--SAGDWARRV-TG
KKFDTGELVKGEFVH--KLPSGFQSYVLNTRRPLLQDARVREALGLAMDYEWMNRQMFYGAYQRVNGLFGNT
ACETK----GSPSPEELALMEPYR----KDIPAS------AFG-PMTVAPRTDG-DSS---LRANLRRAQAL
LKDAGWEVR----------------------------------------------------------DGTLR
NAKG--DAMVLEYMDSN---EGGVRTITPWMRNLEKIGITLRFRTV-DFALYQQRLQ---KFDFDITSIA-Y
QG----TNNP-GQEFADMFGSQAADVEDSGNFVGVKNPAVDAMIKAMTSAKTEAQ-LLPACHALE-RIIAHS
HYLIPQWTAG---------THRMAYNAWRLARPATVPPYASG-EGWVIDTWW--------------------
----------------
>Ottowia/1-593 testudinis
-------------------MRVLLGFLLASAASTAWAAHGYALWG---DMKYPPGFSHFDYVNPGAPKGGEL
IMVSNLRY-----STFDKYNPFTM-------KGSPPAYLGN-----------VLFDTLLTGA---------M
```

DETATGYGLLAEDVQVADDR----LSVVFRLRPQARFHNGDAVRAEDVKHSYDTLVSP-QASPAYRTMLREV
QGAEVIDARTVRFRFH--QPNRELPLTVG-GLPVFSRKWGAG-------KPFDQVVVD-TPIGSGPYRIGPV
QFGRDITYVRDPQYWARDLNVARGTSNFERITVKIYRDNTAKLEALK-AGEFDLMTVY--SAGDWARRI-NG
RRFDTGELVKKEFAH--KLPAGFQSYVLNTRRPMLQDVRVREALGLAIDYEWMNRQMFYGGYPRVRDLFGNT
ACEAT----GLPGADELALLAPWR----GKIPDA------VFG-PMYEPPRTEGPGQS---LRANLRHAQQL
LREAGWTYR--DGALR
DAKG--QPMVLEYLDSR---EGGVRTVGPWIQNLEKLGIKLRFSST-DFALYQQRLD---KFDYDIISLN-I
PG----THNP-GQELTELFGSKQADVEGSASYAGVKSPAVDALANAIVGARSLPQ-LLPACRALD-RVIMHS
HYHIPQWTLS---------SHRVVYNSQKLAYHPPMPPYARA-EEWVMSTWW--------------------
----
>Ahrensia/1-586 marina
--------------------------------------HGLSAFG---DLKYPKGFTHFDYASPDAPTGGTF
NFQPTYWYHNQNTQTFNTLNSFVR-------KGDAPPRMEMC------------FDSLVTSA---------H
DEPDAVYCHLAEYVVISEDR----NSFTFKIRDIARWHDGTPITANDVAFSYRTLKEQG-NP-ELSLPLASL
IEARIDGPMTVTLVFDGSQAAQDILSIAG--FPIISEAWYTEN-------TFDESTLT-PPLASGPYKVGRM
NPGSFIEYERVDNYWAKDLGTAKGLNHFQTIRIDFFKERQAGFEAFK-KGDITFREEY--TAKSWATEYN-F
PAIKDGRVIKALFDS--EKRARMQAWCLNLRRERFLDKRVRDAIAQCFDFEWTNNNIFYGAYERSHSLFGGS
-QYEAN---GKPDASEKELLDQLGS--EHDLPDG------IYEEAYLQPKSDGS---GR--DRNKLRGASKL
LAEAGWKPG---ENGY
LRNDSGETLDLEFLIYA---SVFERIYGPFIETMRAVGINTSLRLV-DPAQYQARTSS---FDFDMAGL---
--AIGWTPTPTASGLEVVFGSKAADTEGSRNLPGLQNSLVDAIIYEIGAANSRER-HETAMRVLD-RVLRLT
REWIPNWSSA---------NHRVAYWD-IFGFK--EPKPDYFW--PVEALWWIDKEKAEKL-----------
----
>Chachezhania/1-575 antarctica
--------------------------------------HGIALYG---EPALPEGFSHLPYANPDAPKGGSV
VFGNAG--------SFDSLNPFIAKG-------RVPWQMS-----------FWGYESLMGRSWDE-------
--PFSLYGLIAESIETPEDR----AWVEFTLRPEARFSDGSPVTVEDVIWSFETLGSEG-HPR-YLAFASKI
ESIEPTGPRSLRITFN-EDNR-ELAMIAG-LRPILQKAQWE-------GKSFSEGLIENIPIGTGPYVVSDY
DIGRSVTFRRNPDYWAADLPLVRGAANFDELRIEFFGDDAVLFEAFK-AGEISAVREF--NAEKWETQ-YTF
PAIQNGDIVKSEIPH--RKPSGMTGFAMNTRRPPFDDWRVREAMLLAFNFEFINDTMTGGAQWRITSYFSGS
ELAMR---HGPADDEVRALLEPFA----DTLLP------DALDA-YDLPVGDGT-ARN----RGNLRRATRL
LAQAGWTVEN--GRLV
DANG--QPFTFTVLLRQAD-QTMARVLDIYTQALEKLGITVRIDQV-DDAQYTEREA---SLDFDMIPFRRA
L-----SLSP-GNEQRFYWGSDVADSPGTRNLPGIESPAVDAMIDRILSVKTPES-FETSVRALD-RVLTTG
RYIIPIWNYE---------VGRIAHVK-QMEYSNNI-PIYGDGPYFMPDFWWWNNN----------------
----
>Bartonella/1-572 apis
--G---SPALGANFDHFPYANPDAPKGGKI
TYGVVG--------TFDGLNPFVIRSF-----RTTARGLFA-----DEQFGGLVYESLMVRTRDE-------
--PFTLYGLIADNVILNDDR----TEITFHLNPKARFSDGKAITADDVLFTVDLLKNKG-RPP-FDRYMKRI
EHIEKLDEQTVKMEFPHSKDR-EFPFILASSMPVLPKHAID-------VQNFEKNGLT-PIPGSGPYIISHI
DAGERIIYERDPNYWGKDLPVNRGLNNFNTVQIEYFRNDNTRFEAFK-KGILDVFLEEPANPNRWRLS-YNF
PAVRHGDVIKESFKK--GTPADMIGFVFNTRKPIFKDRKVRQALSLVFDFEWVNHNLFNDVYSRTEGFWCGS
TLSAV---GRPASDKEKQLLSPYP----DAVLP------AVMDGTWHNVKTDGS-GID----RASAEKAWQL
LREAGYKLQNR---QAIG
PD-G--QPLHFEIMTQS---LEEEKIALAYQRSLVRIGVGVDVRTV-DDTQYQNRLT---SFDYDMIVGKLK
-----ASLSP-GNEQLNRWGSSSRDLEGSFNFAGTSDPAIDAMISALLDARSNED-FTAAVRALD-RVLISG
SYYLPLYHLP---------DQWVAHWS-RIEYPAYT-PLYGYR----LPAWWH-------------------
----
>Aquimixticola/1-588 soesokkakensis
-----------------------------------ITSSYFSTWG---EVKYDAPFAHLDYVNPEAPKGGTI
TISANG--------TFDSLMPYATL------SGTPGALSSIT------------SETLMVGT---------A
DDVSGQYCLLCTTIEYPQNQ----DYVIFHLRDDVTFSDGTPMTAEDVAFSFNLLLEQA-TPSFREAVSKRV
DKVEATGPLTVRYDFNPDVPRKALISQAG-NTPVFSKAWFEET-----GTRLDEASYN-VPLGTGPYMLGDY
KPGEWIEYRRNPDYWGITQPLKVGTENFDTIRVEYFADTSAAFEAFK-AGDITFRQEN--SSLNWATAYD-F
PALDRGDVIKEELAD--GSLPPATGFVFNIREDKFDDPRIRDAIALMYNFSWTNDNLQYGLFAQRESFWENP
-ELKAV---GLPQGRELEILESVK----DDLPEA------IFTEEVRMPHSSG--DRAL--DRGNLRKALAL
FAEAGYEPD---DTGM

```
LRNADGMTFDLEFLSGY---QGYDRIINPYIENLKALGVNAKYTRV-DPNQYQARTQNF---DYDMIID---
--GYNNGLEEGTG-LMQRYGSS-GTE-DIFNPSGFASPATDKIIDTIIQAQTYDE-MAAGVRALD-RVMRYE
FFIVPTWFNN---------KFLVAYYA-MFEHPPEAQMPPYAL--GYLDFWWYNADKAAELKAAG-------
----------------
>Quisquiliibacterium/1-573 transsilvanicum
-------------------------------------------------PKYPPGFTHFDYVNPEAPRGGTL
NLNGFG--------SFDKLNPFTL-------KGMAAAGLG--------T---LMFETLAEAS---------E
DEPFSMYGLLADDMDFAADG----LSITFRLHPAARFSDGSLVLAEDVRHSFETLIGR-QAHPRFRQYFADV
ARVRVLDERRVRFEFA--RRNHELHMILGLQLPVFSRNWGAG-------KPFDQLVQE-PPLASGPYLIDKA
DWGRSITYRRNPGYWGEGLAVRRGSFNFERVTWKYFKDETARLEGFK-AGEFDWVAEN--SARNWARGH-TG
RRYASGEIVKREFPH--SNAAGMQGFALNTRRALFADVRVRRALALAMDFEWMNRQIFYGQYVRSPSYFTNS
EMQAD----GMPGPDELAVLEALR----DRLSPE------VFG-AAPVPPVTTP-PSS---LRENLRQALGL
LAEAGWKVAD---------------------------------------------------------DGRLR
NAAG--APFEFEILSYS---RALERVAVPWARNLEKLGIAARLRTT-DPALYQKRAD---EFDFDVMVGL-L
PA----SQTP-GNELVERFTSAAAKERGSDNVTGVSDPAVDALVQRLLASRSRAE-LVAVARALD-RVLRHG
WYMVPHFHAP---------THRVAYRK-HLAHPERLPRYY-GAESWLLKTWW----VRPGASANGH------
----------------
>Mangrovicella/1-591 endophytica
---------------------------------ADTELHGLSAFG---DLKYSAGFAHFDYASPQAPSGGRL
NLSVPSWILNQSPLTFDTLNTFVL-------RGNAPPRIELL------------YDTLMVSS---------L
DEPDALYGALAESVSMSADR----NRFTFRLRPEARFSTGAPVTADDVAFSYETLKKDG-HP-SLQVMLAEV
TSVTASDERTAVLTFSGRQNVAQALGTFG--VPIIPRAFFAGR-------GLDSVSRE-AVPGSGQYRIGRA
EFGTFIEYEKRDDYWAKDLPFVRGLNHFQTIRIDFFRDRQPALEAFK-KGLITVREEF--TTKDWATQYD-F
PAVTSGAVVRQEFPD--ETRPHFQCWALNQRRERFTDARVRHAINLCFDFEWTNANLLFGQRVHSQSPFEGS
-DFVAT---GEPSEAERALLEPL----RGKVPDE------VFGPVWTQPVTDAS---GA--DRVKLREAAGL
FAAAGWA-R-----------------------------------------------------------QGRQ
LVDAKGEPFRLEYMTNG---QEQGRVYGKFIDTLRLIGVDASIRLV-DAAQYQERVNR---FDFDMILA---
--AFSFAPTPTQDSLAIFFGSAARDNPGAYNYPGMASEAVDALIAKAGAAQSREE-LTTVMKALD-RLLRWR
LDWLPNITSD---------VHRVAYWN-MFGFK--EQKPEYGF--PIESLWWYDAQKAAAIGKA--------
----------------
>Pararhizobium/1-580 mangrovi
-------------------------------ARADEPVNAIAMHG---KPALPADYRHLPYANPDAPEGGSI
TYGVVG--------SFDSLNPFILKSM-----RTTARGIW------DPEFGNLVYESLMQRSMDE-------
--PFSLYGLLAETVQMDDAR----TYIRFNLNPKAHWSDGKPVTADDVVFSFRLLAKKG-RPP-YSNRLNDV
AKITKVGARSVRFDLKKSAGR-ELPLILA-MSPILPKHAID-------PGTFGRTTLE-KPVGSGPYKVGDV
EPGRSIVYKRDPNYWGKDLPVKRGFDNFGTVTVDYFLSQNASFEAFK-KGIFDVFPES--DPIRWRRS-FNF
PAVRDGRVVKDAFRA--ETPSGMYGFVLNTRRPVFKDRRVRHALALALDFQWINRNLFGDAYARTQDYWQGS
SLSCF---GVPASAYEKKLLAPFP----DAVTK------PVMEGTYKLPVTDGS-GRD----RTVLREAYDL
LTAAGYHIDDG---------------------------------------------------------RMVD
RN-G--RPLAFEVMTQN---AGQEKIALAYQRTLASLGIAMSVRTV-DDTQYQQRTL---TFDYDVIVKNYP
-----SSLSP-GSEQIGRWASSSANVEGSFNYAGTKSPAIDATIEAMLDARSQHD-FRDAVRALD-RVLISG
YYVVPLYHVD---------QQWIARWG-RIRHPDTT-PLYGYQ----LETWW--------------------
----------------
>Rhodosalinus/1-600 halophilus
----------------------------------IVVSHGYNFFG---ELSYPPDFEHFDYVNPDAPKGGEI
SLWAPG--------TFDSMNPYSR-------QGRAGRYSWMM------------YESLLGEMP-ASGAGLPA
DQIGESYGLLAWKVEWDPGK----TWVIFHMRPEARFSDGTPVTAHDVVFSHNLLLEQG-LPSYAQAVSRRV
LTAEALDDHTVKFTFAEGISRRSLIDQVG-GVPVWSKAWYEET-----GARLDEPRLD-PAVGSGPYVLESL
DVNRNIVYRRNPDYWGWHLPINQGRHNFDRIRIEYFADDNAAFEAFK-AGVYTFRAEG--NSRTWATGYD-F
PAVDNGWVQLDELPD--GTPPTPTGIVFNLGKELLQDKRVREAIALAYNFEWTNASLQYGLFEQRHSFVQDT
-PLQAE---GPPEGAELEFFQSLEG---VEIPEA------VLEEPARRAHSSSE-ERLN--DRRNLRRAMRL
LDEAGWQVG-----------------------------------------------------------DDGV
RRNGEGEVLSLDFPISSSSSATLESVVETFAQNLELMGIDINVQRI-DPSQYTLRSRER---DYDLIFD---
--NYRAFLQAGTG-LMQMYGSREAEF-SLFNPAGLASPLVDAVIEAALQTESREE-EAAALMALD-RVLRHE
FFMVPVWYND---------SHWVAYWD-QYAYP--EELPPYAL--GTLDFWWYDAEGAAELRAAG-------
----------------
>Limimaricola/1-594 hongkongensis
------------------------------EAEATTTSHGFANFG---ELKYPADFAHLDYVNPDAPKGGEI
```

```
SQWAMG--------TFDSFNSFAR-------QGVSAALNTLP------------LESIMTST---------A
DDPYGLYCYLCSTITYPESL----DWLILDLRDDVTFSDGTPMTAEDVKFSAELFLEQG-ITEYRTRVGSLI
DEIEVLGPHEVKFSFAPEAPRRDVVGLAG-GTVVFSKAWFEET-----GARLDESRDA-PFMGTGPYLLESA
DINRRVVYGRNENFWGADIPFNVGRNNFDTIRIEYFADSAAAFEGFK-SGEYTFRAEN--SSKDWATGYD-F
PAAQKGWVVTEEIPD--GNIGTGQAFVFNLDDPTWQDPRVREAIGLMFNFEWSNESLFYGLYERVESFWENS
-DLEAV---GTPSEAERDLLAPLVE--EGLLPES------ILTDEARRAPVLDAGANGP--DRATYRRAGKL
LEEAGWSVG-----------------------------------------------------------DDGL
RR-KDGAVLELVFLQRS---PQFDRIVNPFIENLARLGVRGVLERV-DTSQYVERTR---SGDFDLVNH---
--SFTMGFEPGGE-LEQWFASKTADD-SSRNLMRLRSTAVDRLIQEVLDAGALDE-LTTATHALD-RVLRAE
MIWVPQWYKD---------VHTVAYYD-QFRHP--DPIPPFAR--GELDFWWYDAEAAQDLRAAG-------
----------------
>Vandammella/1-593 animalimorsus
--------------------------ALGAGIAQAEPSHAYALWG---QPQYPAGFAHFDYANPAAPKGGEL
RLVSNLRT-----STFDKYNPFTL-------RGSAPAYLQA-----------MLFESLLTGA---------L
DETSTGYGLLAESVDVAADR----LSATFRIRAQARFHNGKPVLAEDVRHSFETLISA-QAAPGYATLLEKV
ARAEVLDARTVRFHFK--SPDRELPLVVG-GLPIFSRDWGLDAN--GQRKPFDQIITD-TPIGSGPYRIGPV
VFGRDITYVRDPDYWGRELAVNAGAHNFERITIRIYKDATARLEALK-AGEFDLMQFH--SAGDWARRV-KG
RRFDSGELVKAEFEH--RNPTGFQSYFLNLRRPHLQDVRVRRALALAMDYEWMNRRLFYGSYRRVRGLFGNT
QCAAE----GLPSAAELALLEPWR----SQIPPE------AFG-PMPEPPRTDHSEHG---LRENLRQAREL
LAQAGWTLQ----------------------------------------------------------GGTLR
NAQG--QPLVLEVMDSN---EAGIRTVAPWQRNLQKIGIELRFRVV-DFALMLQRMD---AFDYDITSLN-I
QG----THNP-GQEYAQFFGSAAADQNGSANYSGLKSPAVDALIARMVQAGTQAD-YQAACRALE-RVVTAE
NVMVPAWYAS---------NFRVVYNAQRLAFQAPMPPYVQVLENWAIAYWWH-------------------
----------------
>Paramesorhizobium/1-589 deserti
-----------------------------------KPLHGLSAFG---ELKYPADFTHFDYASPEAPKGGTF
TFGPPNWVFNQSPLTFNTLNSFSN-------KGDAPPRMELC------------FDSLMASA---------L
DEPDAIYGLVAETVTISGDR----NSYVFKLRPEARFSDGTPLTAEDAAFSYQTLKEKG-HP-ELLLALTEL
DEAVAEDAQTLRLTFSGKHSARAILDAAG--MPILSKAWYGTR-------DFSASTLE-PPPGSGPYKVGRF
SPAQFIEYERNKDYWARELPVSRGFYHFDRIRIEFYRDRQPAFEAFK-KGALDWRSES--VAKTWVTEYD-F
PAIREKKVVKRTFPR--EKRPSMQAWALNQRRERFHDPRVREAIALCFDFEWTNKNLFYGVYRRSQSCFGGS
-DFEAR---GKPSPDELAILERY----RDRVPEA------IFGEVVTMPVSDGS---GR--DRKLFRRAIEL
MTEAGFK-R-----------------------------------------------------------DGGR
FTDKDGRPFDLEILSDT---EAFTRIYNPFMQNLRAIGINASLRLV-DPVQYQARTLN---FDFDMMGM---
--AVSMTATPTQDSLGTMFSSKSAGIPGTRNYPGTADPVIDELIQEAGKASSRDE-LVPILQVLD-RLLRLR
RDWIPNWTSA---------NHLVAYWD-RFGFK--EPKPDYGF--PVETLWWVDEEKARAIGKA--------
----------------
>Lutibaculum/1-566 baratangense
-------------------------------------SHGIAMHG---DVKEPPDFTHFGYANPDAPKGGWI
GISVVG--------SFDTLNPFTVTG-------QEARNLS-----------GYVFQPLLARSLGE-------
--PFALYPLIAESVTMPEDR----SWIEFTIDARARFSDGEPVTVDDVIASWRLLRDRG-RPN-HRSYYALV
DSARATGERSVRFSFSPEAER-EMPLIMG-LMPVLPSHVYN-------VETFDKLGLS-PPVGTGPYVVSNV
ARGRSITLTRQKDWWGNDLPATSGQYNFDTIRYEYFRDQETALEAFR-KGIIDFREEE--DPSRWALS-YDF
PAARRGEIVKEALPN--GAPRGMSAFVFNTRRAPFGDIRVREGLSLLFPGAWVNRAYYRDLYERTDSFFDGS
ELSAE---GRPATDHERELLAPWI----DEVPQ------RVLEGDLGGQRRGAD---D----RRALREALRL
LKAGGFELRDG---------------------------------------------------------RLVN
AETG--ERMSFEILVAT---REHERLALAFSRALDRAGIEARVRSV-DSAQYQRRKQ---SYDFDVTHHFWF
S-----SLSP-GNEQRFYWGSAAADQPGTRNYMGVTSPGIDATIDALLAAHDRDD-FVTAVRALD-RLLVSG
HYVVPLFHRP---------SHWVAYQE-RFAHPETP-PLYGYA----LETWW--------------------
----------------
>Aliishimia/1-594 ponticola
-------------------------------------SHGYSFYG---DLTYPADFPHFDFVNPDAPKGGEI
SFARLG--------TFDSMNPYTR-------RGRAPVLSTVM------------YESLLGEG---VGTSGAA
DTYGEGYCLLCERLEYPADK----SWVIFYMRPEARFSDGTPVTAHDVLFSHNLLLEQG-LPSYAAAVKKRI
PKAEVIDDYTIKFYFADGISRRSLIDQVG-GVPVWSKKWYEET-----GARLDEPRLE-ISPGSGPYMLDTF
EVNRRVVYKRNPDYWGWDLPNNKGRYNFDSIRVEYFSDDTAAFEAFK-AGEYTFRGES--DPVKWVQSYD-F
PKVRNGAVKKEAIPD--GTPPEPTGIVFNLQRETLKDKRVREALALAYNFEWTNESLQYGLYAQRYSFTQGS
-RLEAQ---GAPEGAEREFLLSLG----DLVPEE------MLNEEPRTAHSSNA-ERLQ--DRRNLRRALKL
```

```
LDEAGWTVD------------------------------------------------------------DKG
RRVKDGKPMELTFLFNATFPPTLVAVIDNYIHNLRAMGIDVTYEKV-DSSQYTVRQRDK---DFDLIYSN--
--QYRSFLGTGTG-LHQQYGSEDAII-SLFNPASLQSELVDTIIDASLNASSREE-EDMTLRALD-RALRYE
FFMIPLWYNP---------DTWVAYYD-QYDYP--DPLPTYAL--GHLDFWWFDAARNEELKAAG-------
----------------
>Sinorhizobium/1-610 meliloti
----------MGSSHHHHHHSSGLVPRGSHMEEQPVWHHATSSIG---EPKYKDGFARFDYVNPDAPKGGEL
RLSESG--------TFDSFNPILA-------KGEVATGVSS-----------LVFETLLKSAEDE-------
--ITTSYGLLAEGISYPDDI----SSATFRLRAEAKWADGKPVTPEDVVFSFDMVKEH---NPLFSNYYRHV
ISAEKTGERDVTFRFDEKN-NHELPNILG-QFPILPKHWWEGQDAKGSKRDISRTTLE-PVMGSGPYKIASF
QAGGSIRFELRDDYWGKDLNVNVGRYNFRTINYAFFSDRSVQFEAFR-AGNVDFYQDN--SASHWATAY-DF
PAMKDGRVIREEIENPLRATGIMQAFVPNMRREKFKDQRVRQALNYAFDFEDLNRSLAHNAFQRVDSYFWGT
ELAS----SGLPEGREKEILEELK----DKVPA------AVFTTPYKNPVNGDP-QKV----RDNLRKALAL
FKEAGYELK---------------------------------------------------------GSRLVN
AKTG--EPFSFEILLS--N-PTFERTVTPFVNSVRKIGIDARIRTV-DDSQYTNRVR---SYDYDMIYGI-W
AQ----TLVP-GNEQSDYWGSASVNQPGSRNYAGIADPAIDELIRRIVFAPNREE-LVATTRALD-RVLLAH
HYVVPLFYS---------KALRVAYWN-HLARPK--ELPYYGMD--FPDAWWSKNTAAK-------------
----------------
```
